# Synergistic solvent-catalyst paradigm for sustainable aerobic allylic C–H functionalization

**DOI:** 10.1093/nsr/nwaf196

**Published:** 2025-05-16

**Authors:** Rui Wang, Long Zhang, Sanzhong Luo

**Affiliations:** Center of Basic Molecular Science, Department of Chemistry, Tsinghua University, Beijing 100084, China; Department of Chemical Engineering, Tsinghua University, Beijing 100084, China; Center of Basic Molecular Science, Department of Chemistry, Tsinghua University, Beijing 100084, China; Center of Basic Molecular Science, Department of Chemistry, Tsinghua University, Beijing 100084, China

**Keywords:** green chemistry, sustainable synthesis, C–H activation, synergistic catalysis, solvent revolution, amination and alkylation

## Abstract

Achieving sustainable catalytic transformations requires synergistic optimization of solvent systems, catalytic motifs and energy inputs. Herein, we report a synergistic Pd/hydroquinone catalytic system that enables aerobic allylic C–H functions under ambient conditions (room temperature to 50°C, air) with high turnover frequency (TOF), using ethanol/water as a green medium. This strategy achieves unparalleled synthetic efficiency and demonstrates remarkable versatility across two pivotal transformations (alkylation and amination) involving over 90 products (up to 96% yield). It also delivers exceptional stereocontrol (up to 93% *ee* for quaternary stereocenters) and enables advanced allylic transformations within a green framework through additional synergistic catalysis. By integrating solvent engineering with cooperative catalysis, we have developed a scalable platform for the synthesis of allylic functionalized molecules with pharmaceutical interests, demonstrating how molecule-level innovation can drive sustainable industrial transformation.

## Teaser

This study has developed a solvent-catalyst synergistic paradigm for aerobic C–H allylic functionalization reactions, illustrating a sustainable blueprint that integrates atom economy with pharmaceutical relevance.

## INTRODUCTION

**Figure 1.**
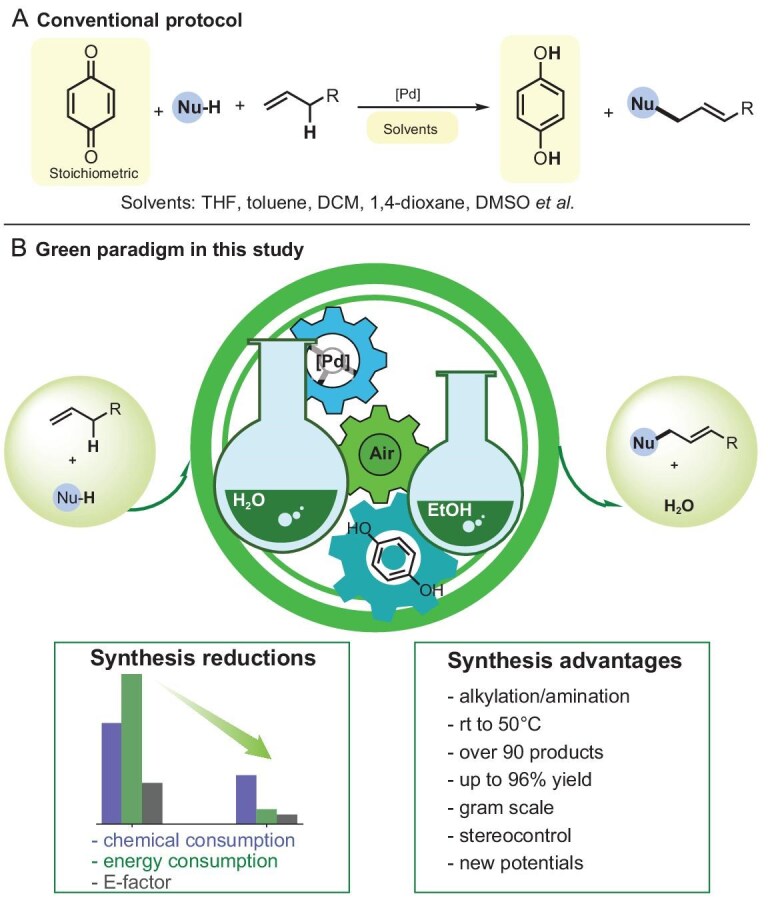
Synergistic solvent-catalyst paradigm for aerobic allylic C–H functionalization.

In the global pursuit of sustainable and environmentally benign chemical processes, palladium-catalyzed allylic C–H functionalization has emerged as an efficacious transformative strategy in synthetic chemistry, enabling direct construction of C–C and C–X bonds with inherent atom economy [1–11]. While palladium-catalyzed allylic chemistry has seen remarkable advances, its industrial adoption remains hampered by reliance on stoichiometric benzoquinone oxidants and hazardous solvents—critical barriers to achieving carbon-neutral chemical production as outlined in China's 14th Five-Year Plan [12] (Fig. 1A). Against this backdrop, the development of green and efficient catalytic systems is not only a scientific imperative but also a strategic necessity for the fine chemical industry [13–16].

Pioneering studies by Stahl *et al.* [17–21], Yu *et al.* [22–24], Báckvall *et al.* [25–27] and others [28–35] have established the viability of aerobic palladium catalysis. Building on these advances, our previous work demonstrated that molecular oxygen (O_2_) from ambient air serves as an ideal green oxidant for aerobic allylic alkylation, leveraging its sustainability advantages—abundance, low cost and environmentally benign byproduct (H_2_O). However, critical challenges persist, including low reaction rates and reliance on hazardous solvents [36], which hinder broader industrial adoption. Moreover, a generalizable methodology for aerobic allylic C–H activation remains elusive. For instance, while recent breakthroughs by White *et al.* [37], Jiang *et al.* [38,39] and Gong *et al.* [40] have advanced allylic amination chemistry, these Pd-catalyzed platforms still necessitate stoichiometric benzoquinone oxidants. Our current research represents an effective approach to overcoming these limitations by integrating solvent engineering with cooperative catalysis.

Water and ethanol, as universal, safe and sustainable solvents, are often overlooked in organic chemistry due to solubility limitations and sensitivity of reactants (hydrolysis or alcoholysis) [41–43]. Additionally, the extra reducing and nucleophilic properties of ethanol make it a cautious choice and rarely examined as a solvent in organic reactions [43–46]. Our findings, which began with the observation that undried dichloromethane (DCM) improved reaction rates in aerobic allylic C–H activation [36], led us to investigate the use of water (the most ideal green solvent) [13,15]. While ethanol, compliant with the *CHEM21 Solvent Guide* [47] and Chinese standards HG/T 5970–2021 and GB 31604.60–2024, serves as a highly viable alternative solvent for enhancing the conversion of specific substrates. Our work leverages these green solvents to achieve broad-spectrum allylic C–H activation, including alkylation, amination and dehydrogenation, with significant benefits for green chemistry. This approach further enables stereoselectivity in alkylation, direct formation of a *Z*-type allylic compound [48–50] and sequential bis-allylic amination of primary amines, offering a sustainable blueprint for fine chemical synthesis. Compared with conventional oxidative allylic C–H functionalizations (e.g. amination and asymmetric alkylation) that yield partially overlapping products, our method significantly reduces the E-factor (environmental factor) and reaction energy consumption (Figs S17 and S18). Preliminary mechanistic studies reveal that the green solvents, in conjunction with air, play a crucial role in enhancing overall catalytic efficiency. Specifically, the green solvents help maintain the activity of hydroquinone (or quinone, the core catalyst for aerobic reactions) and may also enhance the efficiency of palladium catalysis through different pathways to the target products, with this effect being particularly notable in ethanol. This research provides a sustainable and efficient strategy for allylic transformations, emphasizing the importance of both synthetic innovation and green chemistry principles (Fig. 1B).

## RESULTS AND DISCUSSION

### Condition optimizations

**Figure 2.**
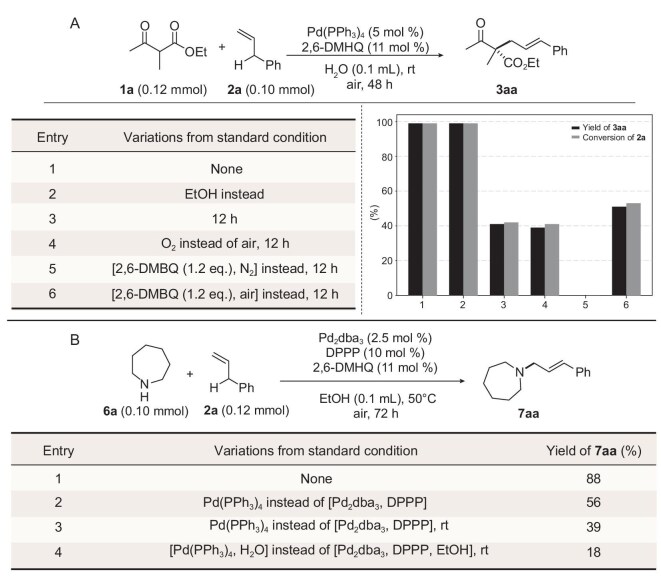
Control experiments. (A) Alkylation: **1a** (0.12 mmol), **2a** (0.10 mmol), Pd(PPh_3_)_4_ (5 mol %), 2,6-DMHQ (11 mol %), H_2_O (0.1 mL), rt, 48 h, air. (B) Amination: **6a** (0.10 mmol), **2a** (0.12 mmol), Pd_2_dba_3_ (2.5 mol %), 2,6-DMHQ (11 mol %), EtOH (0.1 mL), 50°C, 72 h, air. The yield was determined by ^1^H NMR with CH_2_Br_2_ as internal standard.

We have systematically optimized reaction conditions for allylic C–H alkylation and amination, using ethanol or water as green solvents under ambient air conditions. For the alkylation reaction, employing Pd(PPh_3_)_4_ (5 mol%) and 2,6-DMHQ (11 mol%) on water or in ethanol at room temperature (rt) achieved a remarkable yield of >99% for **3aa** within 48 hours (Fig. 2A, entries 1 and 2). This significantly outperformed reactions conducted in a variety of commonly used organic solvents, which showed only trace amounts of product at rt (for detailed solvent and condition screening, including benzoquinones, palladium sources and ligands, see Figs S1–S4). We found that the yield of the target product **3aa** was comparable in air and oxygen under our operational conditions (Fig. 2A, entries 3 and 4; for the detailed procedure, see Supplementary data). Consequently, air was chosen as the more versatile oxygen source for substrate investigation, while oxygen was employed for gram-scale reactions to facilitate operational ease. Additionally, stoichiometric utilization of 2,6-DMBQ revealed that oxidative allylic C–H alkylation was ineffective at rt under N_2_, while **3aa** was achieved under ambient air conditions (Fig. 2A, entries 5 and 6).

For the amination reaction, using Pd₂(dba)₃ in combination with the DPPP ligand in ethanol at 50°C delivered the optimal yields (Fig. 2B, entry 1, 88% for compound **7aa**). This protocol effectively overcame the reaction rate limitations observed with the alkylation conditions used in migration processes (Fig. 2B, entries 3 and 4) and the heating process in EtOH (Fig. 2B, entry 2). Detailed screening of various conditions can be found in Figs S5–S8.

Our ‘green solvent–air’ system also enabled other various transformations, including fine stereocontrol for quaternary centers, sequential dehydrogenation to form conjugated dienes, etherification and more functions, all with high efficiency (Figs S9–S12).

### Scope of alkylation

**Figure 3.**
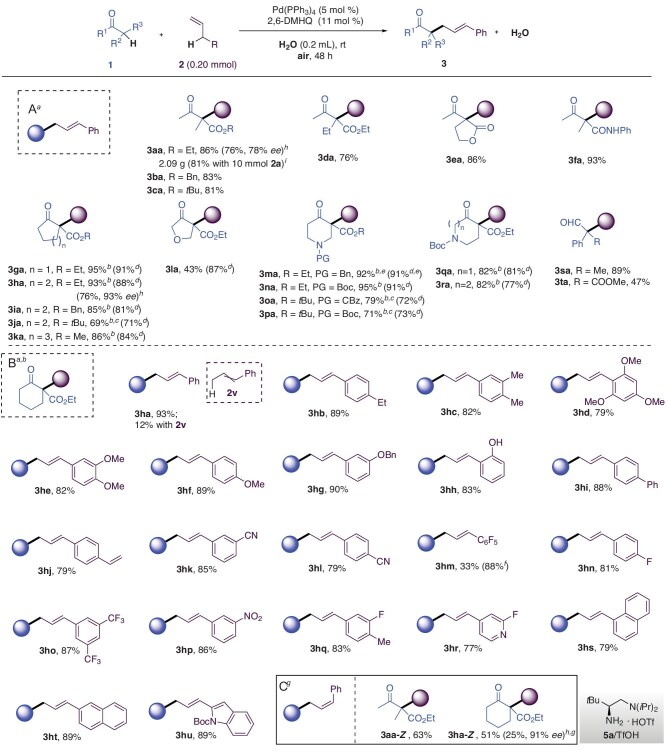
Alkylation scope: (A) with respect to the β-ketocarbonyl and α-branched aldehydes substrates; (B) with respect to the allyl arenes substrates; (C) *Z*-retentive allylic substitution. *^a^*Reaction conditions: **1** (0.24 mmol), **2** (0.20 mmol), Pd(PPh_3_)_4_ (5 mol %), 2,6-DMHQ (11 mol %), H_2_O (0.4 mL), rt, 48 h, air; isolated yield. *^b^*Pd(PPh_3_)_4_ (8 mol %), 2,6-DMHQ (18 mol %) used; isolated yield. *^c^*Another 18 mol % 2,6-DMHQ added after 24 h. *^d^*EtOH (0.4 mL) instead of H_2_O as solvent. *^e^***1** (0.20 mmol), **2a** (0.24 mmol) used. *^f^*At 50°C. *^g^*Additional Ir(ppy)_3_ (2.5 mol %) added, 30 W blue LED. *^h^*Reaction conditions: **1** (0.15 mmol), **2a** (0.10 mmol), **5a**/TfOH (12 mol %), Pd(PPh_3_)_4_ (10 mol %), 2,6-DMHQ (22 mol %), H_2_O (0.15 mL), rt, 48 h, air; isolated yield; the *ee* was determined by HPLC. *^i^*Gram scale: **1a** (12 mmol), **2a** (10 mmol), Pd(PPh_3_)_4_ (5 mol %), 2,6-DMHQ (11 mol %), EtOH (10 mL), rt, 48 h, O_2_; isolated yield.

The protocol demonstrated broad substrate compatibility (Fig. 3). *β*-Ketocarbonyl compounds (esters, amides, lactones) and *α*-branched aldehydes reacted efficiently with allyl arenes to form all-carbon quaternary centers (**3aa**–**3ta**, 76%–95% yield). For instance, the incremental addition of 2,6-DMHQ to bulky substrates (e.g. *t*-butyl esters forming **3ja, 3oa** and **3pa**) was necessary to maintain reactivity on water, while the reaction proceeded robustly in ethanol. The synthesis of **3la** was more efficient in ethanol despite complete substrate conversion on water.

Stereocontrol was achieved *via* a chiral primary amine **5a**/TfOH co-catalytic system, yielding **3aa** in 76% yield with 78% *ee* and **3ha** in 76% yield with 93% *ee* (H_2_O was crucial for maintaining enantioselectivity, outperforming EtOH, see Fig. S9).

The compatibility of various allyl arenes **2** with ethyl 2-cyclohexanone-1-carboxylate **1h** was assessed under on-water conditions using the Pd-hydroquinone synergistic catalyst (Fig. 3B). Allyl arenes with electron-donating (e.g. methyl, ethyl, methoxy) to strong electron-withdrawing substitutions (e.g. –CN, –F, –CF_3_, –NO_2_) showed good to excellent reactivity (forming **3hb**–**3hl** and **3hn**–**3hq**, 79%–93% yield). Allyl pentafluorobenzene gave **3hm** in good yield at 50°C. 4-Allyl-2-fluoropyridine without protection on pyridine nitrogen afforded product **3hr** in acceptable 77% yield. 2-Allylnaphthalene served as a good substrate to offer **3ht** in 89% yield, while 1-allylnaphthalene with higher steric resistance showed limited reactivity to offer **3hs**. The reaction with an indole-derived substrate also proceeded well to offer **3hu** in 89% yield. Internal alkenes such as 1-propenylbenzene **2v** was examined, showing rather poor reactivity.

When irradiated by a 30 W blue LED with Ir(ppy)_3_ (2.5 mol %), the reaction led to the formation of *Z*-retentive allylic substitution **3aa-*Z*** and **3ha-*Z*** (Fig. 3C), albeit with relatively lower yields due to the side reaction of 2,6-DMHQ. While without irradiation, it turned back to *E*-retentive allylic substitution **3aa** and **3ha** mediated by the palladium catalyst. Under photo catalysis with the synergistic Ir(ppy)_3_ (2.5 mol %), the asymmetric catalysis with our chiral primary amine **5a**/TfOH co-catalytic system produced *Z*-retentive allylic substitution **3ha-*Z*** in 25% yield, still maintaining high enantioselectivity with 91% *ee*.

To evaluate the practicality of the method, a gram-scale alkylation to construct all-carbon quaternary product **3aa** was performed under optimized conditions in ethanol with O_2_ as the sole oxidant, yielding the desired allylic products in 81% yield.

### Scope of amination

**Figure 4.**
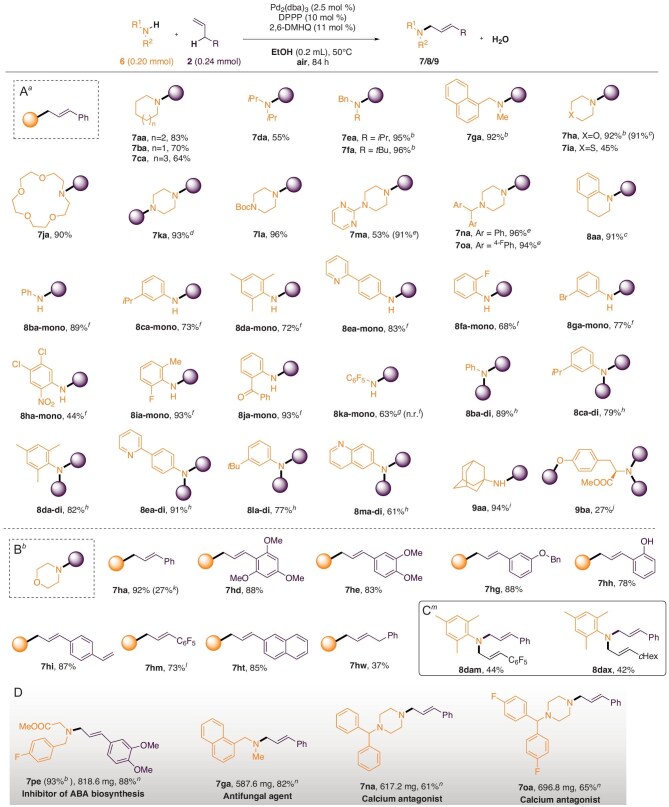
Amination scope: (A) with respect to the amine substrates; (B) with respect to the allylic substrates; (C) bis-allylic amination; (D) gram-scale synthesis. *^a^*Reaction conditions: **6** (0.20 mmol), **2** (0.24 mmol), Pd_2_(dba)_3_ (2.5 mol %), DPPP (10 mol %), 2,6-DMHQ (11 mol %), EtOH (0.2 mL), 50°C, 84 h, air; isolated yield. *^b^*72 h. *^c^*Pd(PPh_3_)_4_ (5 mol %), rt, 72 h. *^d^***6k** (0.10 mmol), **2** (0.24 mmol), Pd_2_(dba)_3_ (10 mol %), DPPP (40 mol %), 2,6-DMHQ (44 mol %), EtOH (0.2 mL), 50°C, 84 h, air; *^e^*Pd_2_(dba)_3_ (5 mol %), DPPP (20 mol %), 2,6-DMHQ (22 mol %) used. *^f^***6’** (0.24 mmol), **2a** (0.20 mmol), Pd_2_(dba)_3_ (2.5 mol %), DPPP (10 mol %), 2,6-DMHQ (11 mol %), EtOH (0.2 mL), 50°C, 72 h, air. *^g^***6’** (0.24 mmol), **2a** (0.20 mmol), Pd(PPh_3_)_4_ (5 mol %), 2,6-DMHQ (11 mol %), EtOH (0.2 mL), 50°C, 72 h, air; the yield was determined by ^1^H NMR with CH_2_Br_2_.*^h^***6’** (0.20 mmol), **2a** (0.50 mmol), Pd_2_(dba)_3_ (2.5 mol %), DPPP (10 mol %), 2,6-DMHQ (11 mol %), EtOH (0.2 mL), 50°C, 96 h, air. *^i^*Pd_2_(dba)_3_ (5 mol %), DPPP (20 mol %), 2,6-DMHQ (22 mol %), EtOH (0.4 mL) used. *^j^***2a** (5 equiv.), Pd(PPh_3_)_4_ (5 mol %), 2,6-DMHQ (11 mol %), H_2_O (0.1 mL), O_2_ used; rt. *^k^*With **2v**. *^l^*96 h. *^m^***6d’** (0.20 mmol), **2a** (0.20 mmol), **2m** or **2x** (0.50 mmol), Pd(PPh_3_)_4_ (5 mol %), 2,6-DMHQ (11 mol %), EtOH (0.2 mL), rt, 48 h, air; then 50°C, 72 h, O_2_. *^n^*Gram scale: **6** (2.5 mmol), **2** (5 mmol), Pd_2_(dba)_3_ (2.5 mol %), DPPP (10 mol %), 2,6-DMHQ (11 mol %), EtOH (2.0 mL), 50°C, 84 h, O_2_; isolated yield.

Under optimized amination conditions, the substrate scope was examined (Fig. 4). A wide range of amines, including aliphatic primary amines, secondary amines and anilines, underwent the reaction efficiently (Fig. 4A). Cyclic alkyl amines provided products **7aa**–**7ca** in fine to good yields. Chain secondary alkyl amines afforded **7da**–**7ga** in considerable to excellent yields. Oxocyclic amines yielded **7ha** and **7ja** in >90% yield, while thiomorpholine gave **7ia** in a moderate yield. Notably, morpholine exhibited special reactivity under optimized conditions. Secondary amines such as piperazine derivatives could be converted to the desired products **7ka**–**7oa** in high yields, mainly with augmenting of the Pd-hydroquinone synergistic catalyst. The reduced activity of piperazine derivatives may be due to the increased alkalinity of the substrates.

Secondary anilines were effectively utilized under ‘room-temperature-on-water’ amination conditions to provide good results, such as **8aa**. Aromatic primary amines could offer mono- and di-allylation products in fine to excellent yields with exclusive chemoselectivity under separate optimized conditions. With aromatic primary amines **6** (0.24 mmol) bearing different electronic/site substituents and allyl benzene **2a** (0.20 mmol), mono-allylation products **8ba**-mono–**8ja**-mono were obtained in up to 93% yield within 72 hours. The yield of **8ha**-mono was low, likely due to the poor solubility of the amine substrate. The bromination substrate intimated derivative potentiality was transformed into **8ga**-mono in fine yield; **8ka**-mono was synthesized on water in fine yield, but the reaction did not proceed in ethanol, likely due to the lower nucleophilicity and concentration of pentafluoroaniline. With aromatic primary amines **6** (0.20 mmol) bearing electron-donating substituents and allyl benzene **2a** (0.50 mmol), di-allylation products **8-di** were obtained in up to 91% yield within 96 hours.

Alkyl primary amines could also be converted to desired products in good yield using a similar protocol, such as **9aa**. Considerable tri-allylation **9ba** was achieved with a tyrosine derivative on water under an oxygen atmosphere.

The compatibility of various allylic substrates **2** with morpholine **1h** was also evaluated (Fig. 4B). Allyl arenes bearing electron-donating substituents provided **7hd/7he/7hg** in >80% yield. A free hydroxyl substituted 2-allylphenol afforded **7hh** in 78% yield. Olefin-substituted allyl arenes also furnished desired **7hi** in 87% yield with the other double bond remaining intact. Pentafluoroallylbenzene bearing strong electron-withdrawing substituents showed relatively low reactivity but still provided **7hm** in 73% yield within 96 hours. 2-Allylnaphthalene served as a good substrate to offer **7ht** in 85% yield. Alkyl alkene 4-phenyl-1-butene provided **7hw** in 37% yield, while 1-propenylbenzene **2v** offered **7ha** in 27% yield.

Based on the different reactivity of allylic substrates, a sequential bis-allylic C–H amination with primary amines was also achieved using the developed synergistic catalysis; **8dam** and **8dax** were obtained in satisfactory yields under one-pot conditions (**8da–di** <20%, the sum of all other allylic products <10%, Fig. 4C, Fig. S12).

Gram-scale amination to form FDA-certified biologically active allylic amines **7pe** (inhibitor of ABA biosynthesis), **7ga** (antifungal agent), **7na** (calcium antagonist) and **7oa** (calcium antagonist) was performed under optimized conditions in ethanol with O_2_ as the sole oxidant, yielding the desired products in good to excellent yields.

### Mechanism studies

**Figure 5.**
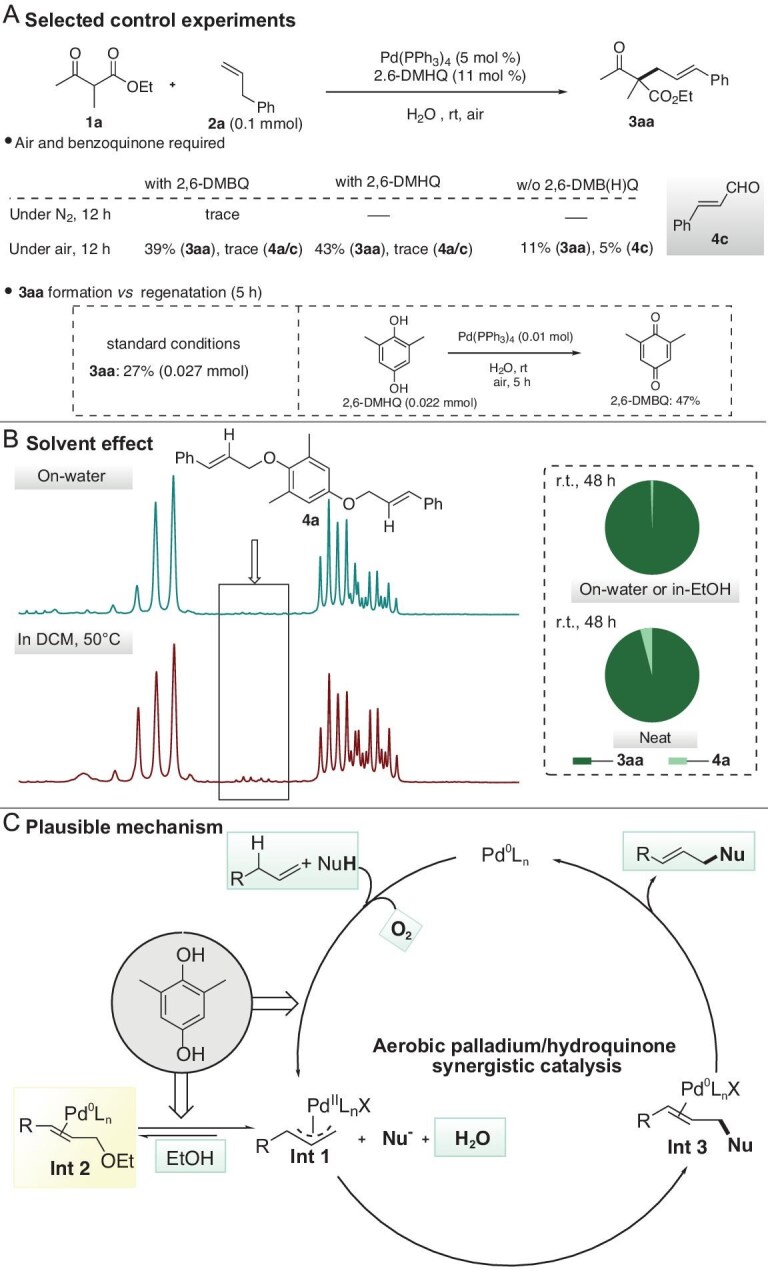
Mechanism studies. (A) control experiments; (B) solvent effects; (C) proposed catalytic cycle.

We explored the mechanism underlying our ‘green solvent-empowered’ aerobic allylic C–H functionalization facilitated by synergistic Pd-hydroquinone catalysis. Control experiments revealed that the alkylation reaction was completely suppressed at rt when either (hydro)quinone or oxygen was absent (Fig. 2A, entries 5 and 6, and Fig. 5A; Figs S4, S13 and S14; Fig. S7 for amination). Hydroquinone (2,6-DMHQ) demonstrated similar and even slightly better performance when compared with its quinone (2,6-DMBQ) precursor (Fig. 5A, details in Figs S4 and S7). In a separate experiment, it was found that the regeneration of 2,6-DMBQ from its hydroquinone counterpart 2,6-DMHQ was much slower than the formation of **3aa** under identical aerobic Pd catalysis (Fig. 5A). These critical findings collectively suggest that the current catalytic system operates through a mechanism distinctive from both the conventional quinone–regeneration-driven C–H activation processes [17,25] and the direct quinone mediated C–H activation process as previously reported by Gong and White [15,34,51–54].

A further increase in the loading of hydroquinone 2,6-DMHQ led to only minor improvement on activity along with the accumulation of side-adducts such as **4a** (Fig. S4, Appendix B). Under aerobic conditions, minor yet notable background reactivity was observed in the absence of quinone/hydroquinone cocatalyts (Fig. 5A). Thereby we arrived at a key conclusion: (hydro)quinone likely does not follow typical regeneration pathways but instead synergizes with air to facilitate allylic C–H functionalization during the palladium cycle (Fig. 5A). Recent studies have shown that stoichiometric reductive species combined with O_2_ can boost the oxygen reduction reaction (ORR) and the efficiency of cascade oxidation of substrates [55], which aligns with our observations.

In conventional organic solvents such as DCM, the synergistic Pd/(hydro)quinone showed rather low and even no activity (Fig. S3). In these instances, hydroquinone loses its catalytic activity upon etherification *via* electrophilic attack on the π-allylpalladium intermediate, leading to etherification byproducts as experimentally observed [45]. In stark contrast, reactions conducted in H_2_O (except those involving bulky nucleophiles requiring additional 2,6-DMHQ) show no detectable etherification byproducts **4a** by NMR analysis (Fig. 5B). Although ethanol-mediated reactions consistently generate trace etherification products, arising from ethanol as nucleophile, these do not impede the thermodynamically favored alkylation/amination ratio (Fig. S3, entry 15), which is consistent with the reversible allylic etherification processes reported in recent oxidative allylic functionalization studies [45]. On the other hand, the reaction under neat conditions, though equally fast, was unavoidably accompanied with a considerable amount of side products such as **4a** (Fig. 5B). In addition, the solid nature of specific substrates also limited the applicability of the neat conditions. These results highlight the beneficial features of water and ethanol as green solvents.

We attribute this solvent-driven enhancement to two key mechanisms: (1) water or ethanol can largely suppress the nucleophilic attack of 2,6-DMHQ on π-allylpalladium species through solvation, thereby maintaining the activity of the (hydro)quinone co-catalyst (Fig. 5C); (2) the protic and polar nature may facilitate the palladium catalytic cycles. In ethanol, these paths include oxidative C–H activation (Fig. 5C, formation of **Int1**) and reductive functionalization of etherification byproducts (Fig. 5C, from **Int2** to **Int1**). In the control experiment in the absence of nucleophiles, effective etherification was observed and the obtained allylic ethyl ether underwent smooth alkylation when nucleophiles were supplied (Fig. S14). With water, the on-water nature may facilitate the overall process by hydrophobic concentration effects as well as the known interphase O–H effect.

In summary, we have preliminarily proposed a plausible mechanism for the reaction (Fig. 5C), involving synergistic palladium/hydroquinone catalysis, solvent and air working together to achieve the target product.

## CONCLUSION

By integrating solvent engineering with cooperative Pd/hydroquinone catalysis, we have established a sustainable platform for allylic C–H functionalization that achieves unparalleled efficiency while eliminating wasteful oxidants and toxic reagents. This system aligns with the general trends in sustainable chemistry and directly addresses the strategic goals of China's green chemistry agenda. Mechanistic studies have revealed the key roles of palladium activity in a novel parallel-mechanism paradigm, where we have utilized green solvents to maximize the turnover number (TON) and turnover frequency (TOF) for specific allylic product synthesis and highlight the potential for further innovation. This work bridges molecular innovation with industrial decarbonization and provides a blueprint for green pharmaceutical synthesis, aligning with current sustainable chemistry efforts.

## METHODS

Full experimental details and characterization of the compounds are given in the Supplementary data.

## Supplementary Material

nwaf196_Supplementary_File
