# Supplementary material for: Synergistic solvent-catalyst paradigm for sustainable aerobic allylic C–H functionalization
Source: Natl Sci Rev. 2025 May 16;12(8):nwaf196. doi: 10.1093/nsr/nwaf196 (PMC12236312; doi:10.1093/nsr/nwaf196)
Supplement: nwaf196_Supplementary_File [file nwaf196_supplementary_file.pdf]

Supplementary Data for

Synergistic Solvent-Catalyst Paradigm for Sustainable Aerobic Allylic C–H Functionalization

Rui Wang (王瑞)[1,2], Long Zhang (张龙)[1] and Sanzhong Luo (罗三中)[1]*

[1] Center of Basic Molecular Science, Department of Chemistry, Tsinghua University, Beijing 100084, China

[2] Department of Chemical Engineering, Tsinghua University, Beijing 100084, China

*Corresponding author. E-mail: luosz@tsinghua.edu.cn

Table of Contents

1. General consideration 3

2. Compound catalog 4

3. Details of experiments 8

    3.1 Optimization of alkylation 8

    3.2 Optimization of amination 10

    3.3 Other allylic C–H functionalizations........ 13

    3.4 Detailed reaction procedure within green paradigm 16

    3.5 Characterization of alkylation products 19

    3.6 Characterization of amination products 36

4. Supplementary mechanism study........ 53

    4.1 Supplementary control experiments........ 53

    4.2 The K_H/K_D experiment........ 53

    4.3 Proposed mechanism........ 54

5. Comparison with conventional protocal 55

6. NMR spectra 56

7. HPLC Spectra 152

8. References........ 155

1. General consideration

All reagents were purchased from commercial sources and used as received, unless otherwise noted. Silica gel was used for column chromatography. Analytical thin layer chromatography (TLC) was visualized with UV light, or stained with $KMnO_4$, DNP and phosphomolybdic acid hydrate solutions. ^{1}H, and ^{13}C NMR spectra were measured on Bruker AV 400 M (400 MHz for ^{1}H NMR; 101 MHz for ^{13}C NMR). Chemical shifts of ^{1}H NMR spectra were recorded relative to TMS (δ 0.00) or residual protonated solvents ($CDCl_3$: δ 7.26). Chemical shifts of ^{13}C NMR spectra were recorded relative to solvent resonance ($CDCl_3$: δ 77.16). ^{13}C NMR spectra were obtained at 101 MHz using a proton-decoupled pulse sequence and were tabulated by the observed peak. The following abbreviations were used to express the multiplicities: s = singlet; d = doublet; t = triplet; q = quartet; m = multiplet; td = triplet of doublet; dt = doublet of triplet; dd = doublet of doublet; br = broad; ABq = AB quartet. GC analysis was performed on a Shimadzu GC-2030 instrument equipped with an FID detector using argon as the carrier gas. High resolution mass spectra were obtained using electrospray ionization (ESI) on Thermo-Fisher Scientific Q-Exactive spectrometer. The enantiomeric excesses were determined by HPLC analysis on Chiral Daicel Chiralpak OJ-H. Optical rotation were measured on a commercial polarimeter and reported as follows: $[\alpha]_D^{25}$ (c = g/100 mL, solvent).

2. Compound catalog

Table 1. Product catalog (part 1).

Click buttons for quick positioning: NMR

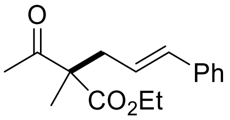

3aa

DETAILS NMR HPLC CATALOG

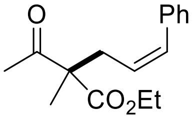

3aa-*Z*

DETAILS NMR CATALOG

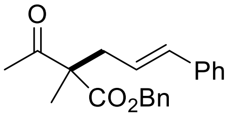

3ba

DETAILS NMR CATALOG

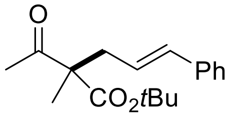

3ca

DETAILS NMR CATALOG

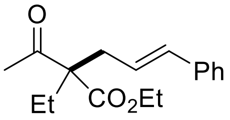

3da

DETAILS NMR CATALOG

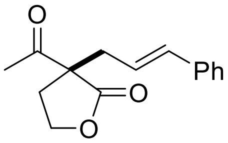

3ea

DETAILS NMR CATALOG

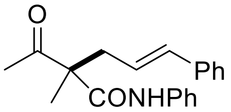

3fa

DETAILS NMR CATALOG

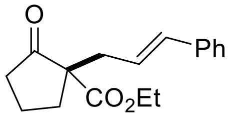

3ga

DETAILS NMR CATALOG

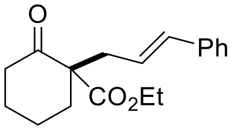

3ha

DETAILS NMR HPLC E-Fact CATALOG

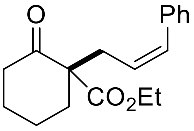

3ha-*Z*

DETAILS NMR HPLC CATALOG

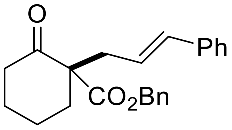

3ia

DETAILS NMR CATALOG

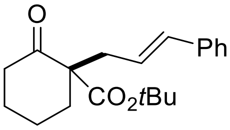

3ja

DETAILS NMR CATALOG

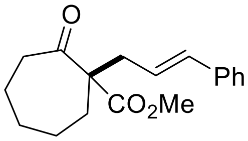

3ka

DETAILS NMR CATALOG

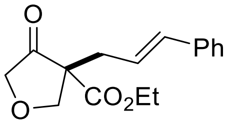

3la

DETAILS NMR CATALOG

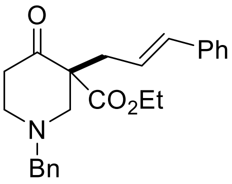

3ma

DETAILS NMR CATALOG

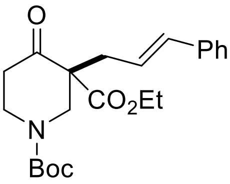

3na

DETAILS NMR CATALOG

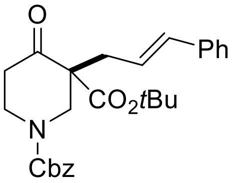

3oa

DETAILS NMR CATALOG

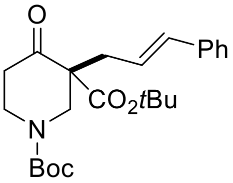

3pa

DETAILS NMR CATALOG

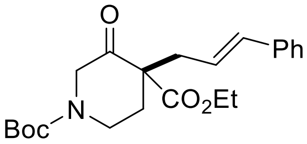

3qa

DETAILS NMR CATALOG

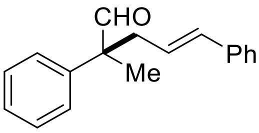

3sa

DETAILS NMR CATALOG

Table 2. Product catalog (part 2).

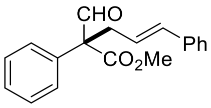 **3ta** DETAILS NMR CATALOG	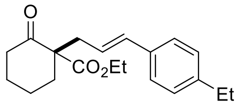 **3hb** DETAILS NMR CATALOG	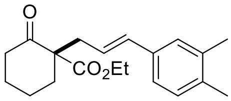 **3hc** DETAILS NMR CATALOG	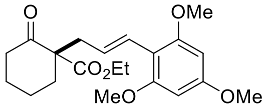 **3hd** DETAILS NMR CATALOG
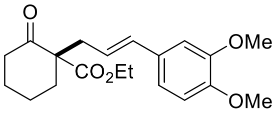 **3he** DETAILS NMR CATALOG	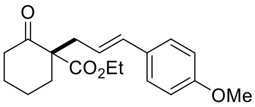 **3hf** DETAILS NMR CATALOG	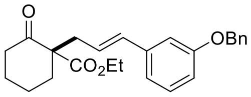 **3hg** DETAILS NMR CATALOG	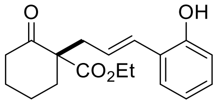 **3hh** DETAILS NMR CATALOG
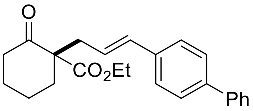 **3hi** DETAILS NMR CATALOG	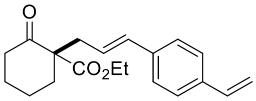 **3hj** DETAILS NMR CATALOG	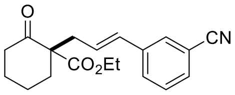 **3hk** DETAILS NMR CATALOG	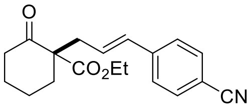 **3hl** DETAILS NMR CATALOG
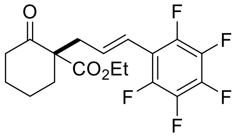 **3hm** DETAILS NMR CATALOG	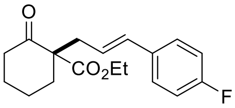 **3hn** DETAILS NMR CATALOG	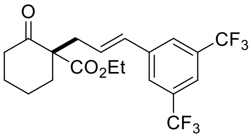 **3ho** DETAILS NMR CATALOG	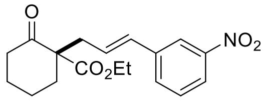 **3hp** DETAILS NMR CATALOG
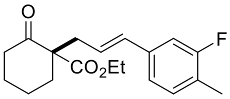 **3hq** DETAILS NMR CATALOG	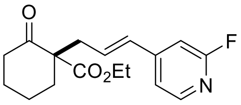 **3hr** DETAILS NMR CATALOG	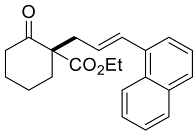 **3hs** DETAILS NMR CATALOG	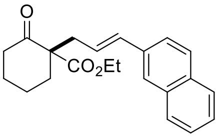 **3ht** DETAILS NMR CATALOG
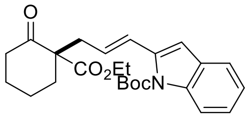 **3hu** DETAILS NMR CATALOG	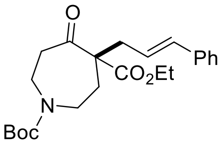 **3ra** DETAILS NMR CATALOG		

Table 3. Product catalog (part 3). CATALOG

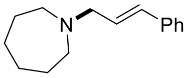

7aa

DETAILS NMR

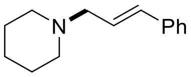

7ba

DETAILS NMR

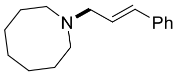

7ca

DETAILS NMR

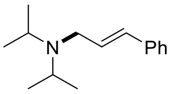

7da

DETAILS NMR

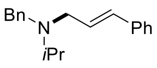

7ea

DETAILS NMR

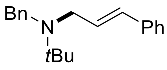

7fa

DETAILS NMR

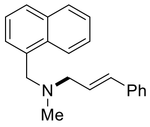

7ga

DETAILS NMR

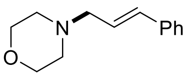

7ha

DETAILS NMR E-Fact

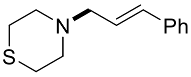

7ia

DETAILS NMR

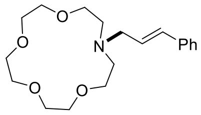

7ja

DETAILS NMR

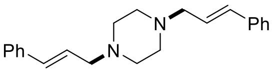

7ka

DETAILS NMR

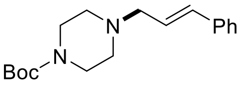

7la

DETAILS NMR

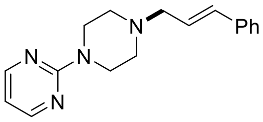

7ma

DETAILS NMR

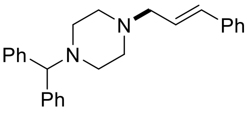

7na

DETAILS NMR

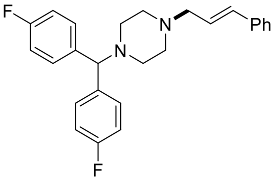

7oa

DETAILS NMR

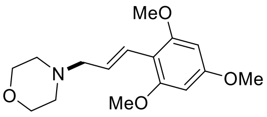

7hd

DETAILS NMR

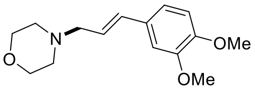

7he

DETAILS NMR

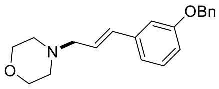

7hg

DETAILS NMR

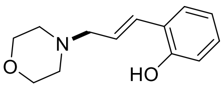

7hh

DETAILS NMR

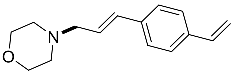

7hi

DETAILS NMR

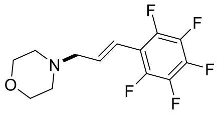

7hm

DETAILS NMR

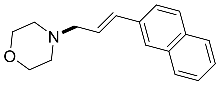

7ht

DETAILS NMR

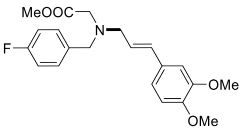

7pe

DETAILS NMR

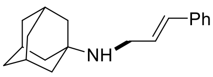

9aa

DETAILS NMR

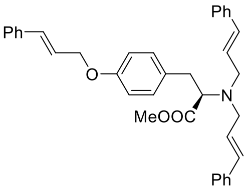

9ba

DETAILS NMR

Table 4. Product catalog (part 4). CATALOG

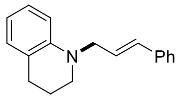

8aa

DETAILS NMR

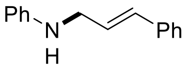

8ba-mono

DETAILS NMR

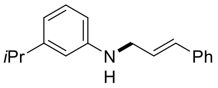

8ca-mono

DETAILS NMR

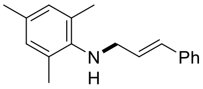

8da-mono

DETAILS NMR

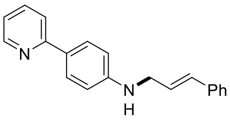

8ea-mono

DETAILS NMR

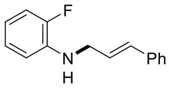

8fa-mono

DETAILS NMR

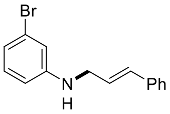

8ga-mono

DETAILS NMR

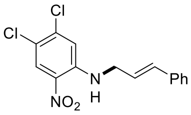

8ha-mono

DETAILS NMR

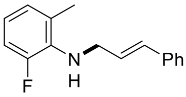

8ia-mono

DETAILS NMR

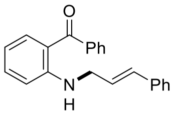

8ja-mono

DETAILS NMR

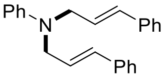

8ba-di

DETAILS NMR

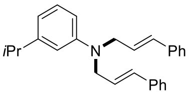

8ca-di

DETAILS NMR

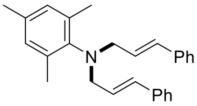

8da-di

DETAILS NMR

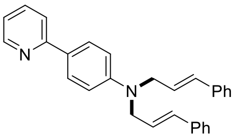

8ea-di

DETAILS NMR

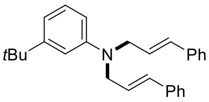

8la-di

DETAILS NMR

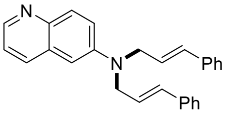

8ma-di

DETAILS NMR

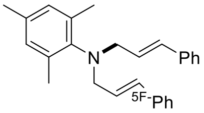

8dam

DETAILS NMR

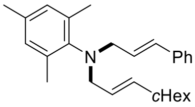

8dax

DETAILS NMR

3. Details of experiments

3.1 Optimization of alkylation

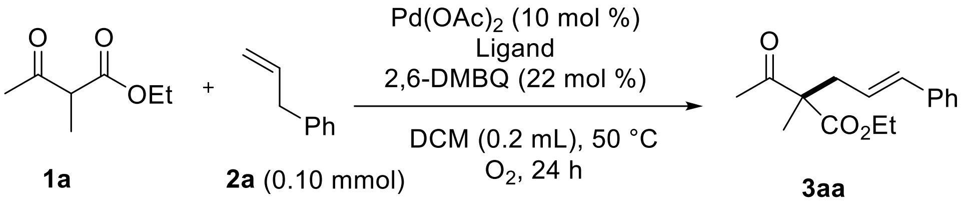

Entry	Ligand	Yield (%)[a]
1	PPh_3 (30 mol %)	57
2	$AsPh_3$ (30 mol %)	65
3	DPPF (15 mol %)	Trace
4	BINAP (15 mol %)	Trace
5	DPPP (15 mol %)	Trace
6	Xantphos (15 mol %)	Trace
7	PPh_3 (40 mol %)	77 (88[b])
8	$AsPh_3$ (40 mol %)	85
9[c]	PPh_3 (40 mol %)	4

Figure 1 Initial alkylation in DCM.

a) Reaction conditions: **1a** (0.12 mmol), **2a** (0.1 mmol), $Pd(OAc)_2$ (10 mol %), Ligand (X mol %), 2,6-DMBQ (22 mol %), DCM (0.2 mL). Prior to the initiation of the reaction and during its course, gas replacement is performed by connecting an oxygen balloon for a certain period. The reaction mixture is then sealed and heated at 50 °C for 24 hours. NMR yield using CH_2Br_2 as an internal standard. b) 36 h, replacement with oxygen is carried out twice; isolated yield. c) Without DMBQ.

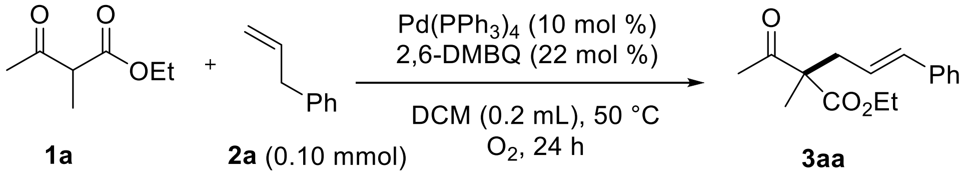

Entry	Conditions	Yield (%)[a]
1	standard conditions	73
2	add $AsPh_3$ (30 mol %)	81
3	add PPh_3 (30 mol %)	79
4	add *m*-nitrobenzoic acid (10 mol %)	55
5	add adipic acid (10 mol %)	49
6	without 2,6-DMBQ (22 mol %)	10
7	reaction time: 36 h	86[b]

Figure 2 Alkylation in DCM with $Pd(PPh_3)_4$

a) Reaction conditions: **1a** (0.12 mmol), **2a** (0.1 mmol), $Pd(PPh_3)_4$ (10 mol %), 2,6-DMBQ (22 mol %), DCM (0.2 mL). Prior to the initiation of the reaction and during its course, gas replacement is performed by connecting an oxygen balloon for a certain period. The reaction mixture is then sealed and heated at 50 °C for 24 hours. NMR yield using CH_2Br_2 as an internal

standard. b) 36 h, replacement with oxygen is carried out twice; isolated yield.

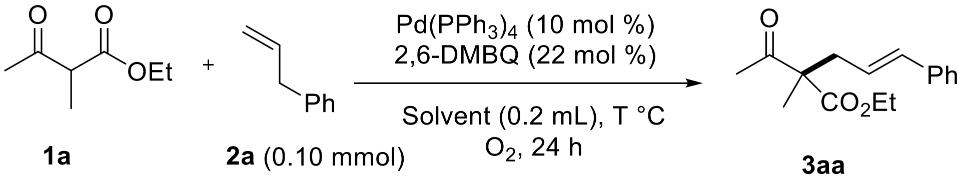

Entry	Solvent	T (℃)	Yield (%)[a]
1	H_2O	50	>99
2	EtOH	50	>99
3	MeOH	50	>99
4	$CHCl_3$	50	13
5	CH_3CN	50	26
6	THF	50	N.R.
7	DCE	50	trace
8	1,4-Dioxane	50	N.R.
9	Toluene	50	N.R.
10[c]	Neat	rt	91[bg] (92[d])
11	H_2O	rt	>99
12	EtOH	rt	>99
13	MeOH	rt	>99
14	DCM	rt	trace
15[c]	EtOH	rt	71[b] (>99[d])
16[c,e]	H_2O	rt	78 (>99[d])
**17[c,e]**	**H_2O**	**rt**	**>99[d] (93[d,f])**

Figure 3 Screening of solvents

a) Reaction conditions: **1a** (0.12 mmol), **2a** (0.1 mmol), $Pd(PPh_3)_4$ (10 mol %), 2,6-DMBQ (22 mol %), DCM (0.2 mL). Prior to the initiation of the reaction and during its course, gas replacement is performed by connecting an oxygen balloon for a certain period. The reaction mixture is then sealed and heated at 50 ℃ for 24 hours. NMR yield using CH_2Br_2 as an internal standard. b) The conversion rate of the allylic substrate is close to 100%. c) $Pd(PPh_3)_4$ (5 mol %) and 2,6-DMBQ (11 mol %). d) The reaction time is 48 hours, during which oxygen gas replacement is carried out three times.e) The reaction is conducted without an oxygen balloon and the sealed vial is opened every 2-3 hours to replace the air. f) 0.2 mmol scale; isolated yield.g) **4a**: 4%.

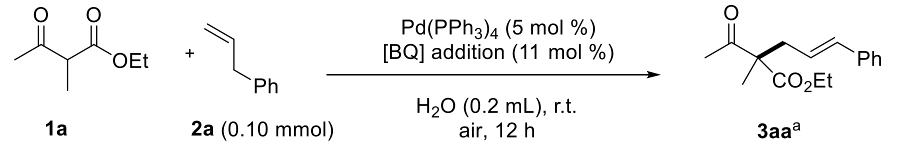

Pd(PPh3)4 (5 mol %)
[BQ] addition (11 mol %)
H2O (0.2 mL), r.t.
air, 12 h
OEt
Ph
CO2Et
1a
2a (0.10 mmol)
3aa[a]

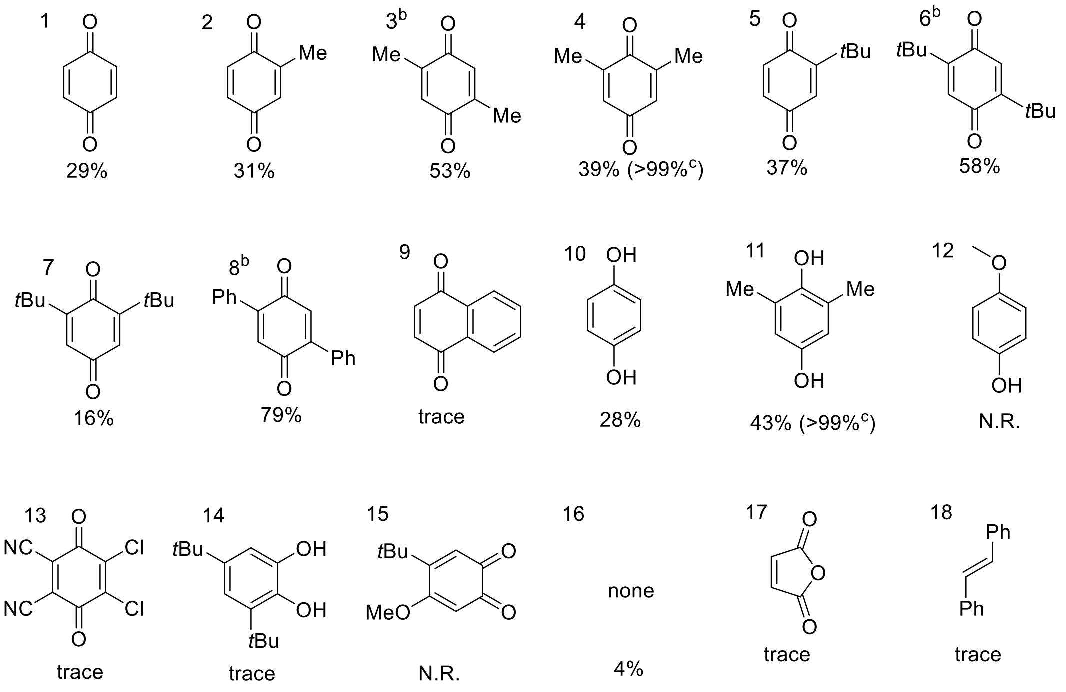

1
29%
2
Me
31%
3[b]
Me
Me
53%
4
Me
Me
39% (>99%[c])
5
tBu
37%
6[b]
tBu
tBu
58%
7
tBu
tBu
16%
8[b]
Ph
Ph
79%
9
trace
10
OH
OH
28%
11
Me
Me
OH
OH
43% (>99%[c])
12
OH
N.R.
13
NC
NC
Cl
Cl
trace
14
tBu
OH
OH
tBu
trace
15
tBu
MeO
N.R.
16
none
4%
17
trace
18
Ph
Ph
trace

Appendix A:

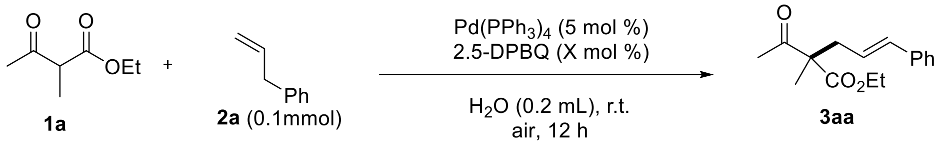

Pd(PPh3)4 (5 mol %)
2.5-DPBQ (X mol %)
H2O (0.2 mL), r.t.
air, 12 h
OEt
Ph
CO2Et
1a
2a (0.1mmol)
3aa

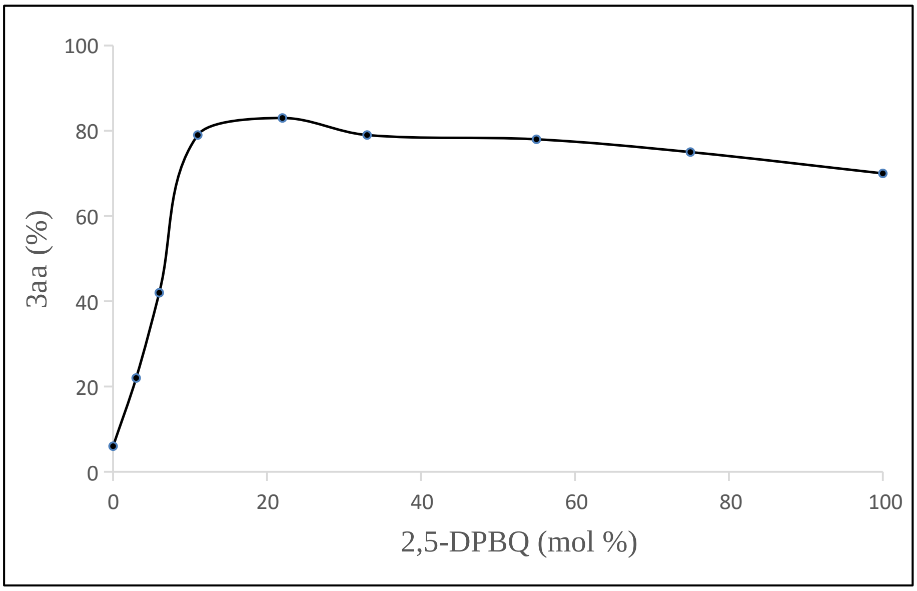

3aa (%)
100
80
60
40
20
0
0
20
40
60
80
100
2,5-DPBQ (mol %)

Appendix B:

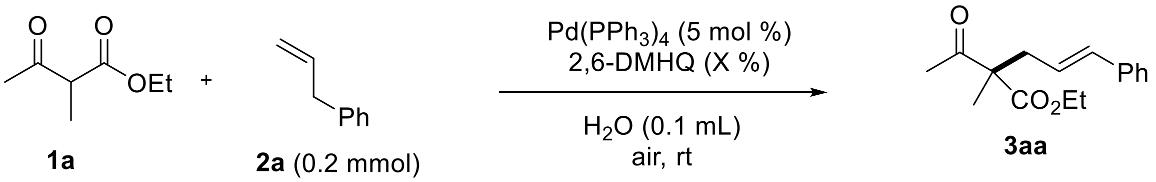

1st parallel experiment (12 h):

2,6-DMHQ: 0, **3aa**: 4%;
2,6-DMHQ: 6, **3aa**: 17%;
2,6-DMHQ: 11, **3aa**: 39%;
2,6-DMHQ: 50, **3aa**: 49%; (**4a** appears)

2nd parallel experiment (9 h):

2,6-DMHQ: 11, **3aa**: 37%;
2,6-DMHQ: 22, **3aa**: 43%;
2,6-DMHQ: 33, **3aa**: 33%;

Appendix C:

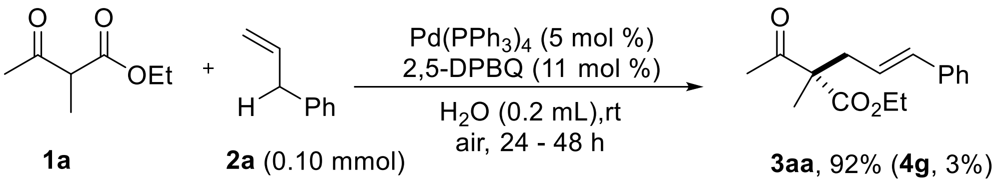

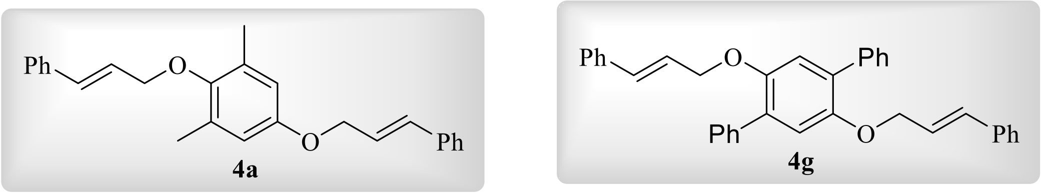

Figure 4 Screening of benzoquinones and back-bonded π materials.

a) Reaction conditions: **1a** (0.12 mmol), **2a** (0.1 mmol), $Pd(PPh_3)_4$ (5 mol %), [BQ] (11 mol %), H_2O (0.1 mL). The reaction mixture is exposed to air for a period before the reaction starts, then sealed and allowed to react at room temperature for 12 hours. The yield is determined by NMR using CH_2Br_2 as an internal standard. b) Limited substrate applicability c) The reaction time is 48 hours, during which oxygen gas replacement is carried out three times; same result in ethanol (0.1 mL).

3.2 Optimization of amination

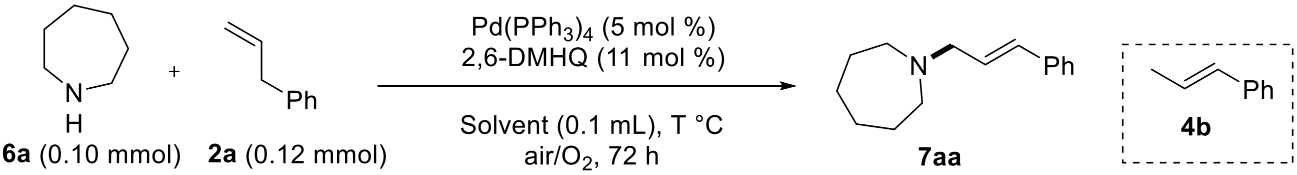

Entry	Solvent	T (℃)	Yield (**7aa**, %)[a]
1	H_2O	rt	18[b]
2	EtOH	rt	39
3[c]	EtOH	50	56

Figure 5 Initial amination trial.

a) Reaction conditions: **6a** (0.10 mmol), **2a** (0.12 mmol), $Pd(PPh_3)_4$ (5 mol %), 2,6-DMHQ (11 mol %). Air replacement is carried out for a period before the reaction starts and midway (every 3 hours). The reaction mixture is placed in a closed vessel with 0.1 mL of solvent at a certain temperature for 72 hours. The yield is determined by ^1H NMR using CH_2Br_2 as an internal standard. b) Byproduct **4b**: 6% yield. c) Oxygen replacement is carried out for a period by connecting an oxygen balloon (every 18 hours) after cooling,.

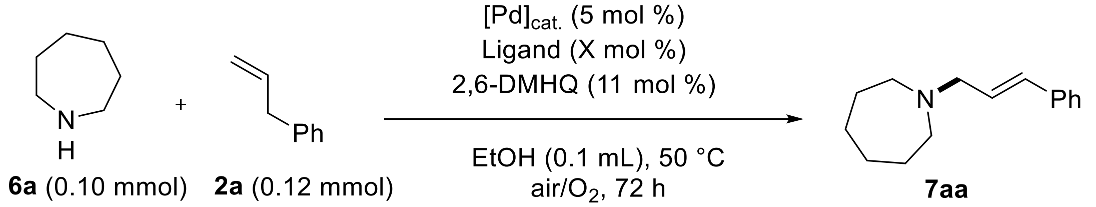

Entry	$[Pd]_{cat.}$	Ligand (X mol %)	Yield (**7aa**, %)
1	$Pd(PPh_3)_4$ (5 mol %)	none	56
2	$Pd(PPh_3)_4$ (5 mol %)	PPh_3 (10 mol %)	45
3	$Pd_2(dba)_3$ (2.5 mol %)	PPh_3 (20 mol %)	52
4	$Pd_2(dba)_3$ (2.5 mol %)	DPPF (10 mol %)	73
**5**	**$Pd_2(dba)_3$ (2.5 mol %)**	**DPPP (10 mol %)**	**78**
6	Pd $(dba)_2$ (5 mol %)	DPPF (10 mol %)	69
7	Pd $(dba)_2$ (5 mol %)	DPPP (10 mol %)	71
8	$Pd_2(dba)_3$ (2.5 mol %)	DPEP (10 mol %)	47
9	$Pd_2(dba)_3$ (2.5 mol %)	Xantphos (10 mol %)	41
10	$Pd_2(dba)_3$ (2.5 mol %)	R-Binap (10 mol %)	35
11	$Pd_2(dba)_3$ (2.5 mol %)	$AsPh_3$ (20 mol %)	trace
12	Pd/C (5 mol %)	DPPP (10 mol %)	trace
13	$Pd(OAc)_2$ (5 mol %)	DPPP (10 mol %)	trace
14	$Pd(TFA)_2$ (5 mol %)	DPPP (10 mol %)	trace
15	$[PdCl(allyl)]_2$ (2.5 mol %)	DPPP (10 mol %)	trace
**16[b]**	**$Pd_2(dba)_3$ (2.5 mol %)**	**DPPP (10 mol %)**	**89 (83[c])**
17[d]	$Pd_2(dba)_3$ (2.5 mol %)	DPPP (10 mol %)	76
18[e]	$Pd_2(dba)_3$ (2.5 mol %)	DPPP (10 mol %)	51
**19[f]**	**$Pd_2(dba)_3$ (2.5 mol %)**	**DPPP (10 mol %)**	**88[c]**

Figure 6 Screening of [Pd]/Ligand.

a) Reaction conditions: **6a** (0.10 mmol), **2a** (0.12 mmol), [Pd]cat./Ligand, 2,6-DMHQ (11 mol %). Before the reaction starts and midway after cooling, oxygen replacement is carried out for a period by connecting an oxygen balloon (every 18 hours). The reaction mixture is placed in a closed vessel with 0.1 mL of EtOH and heated at 50 °C for 72 hours. The yield is determined by NMR using CH_2Br_2 as an internal standard. b) The reaction is conducted on a **6a** (0.2 mmol) scale for 84 hours. c) Isolated yield. d) Reaction time: 96 hours. e) at 80 °C. f) Before the reaction starts and midway after cooling, air replacement is carried out for a period (every 3-8 hours).

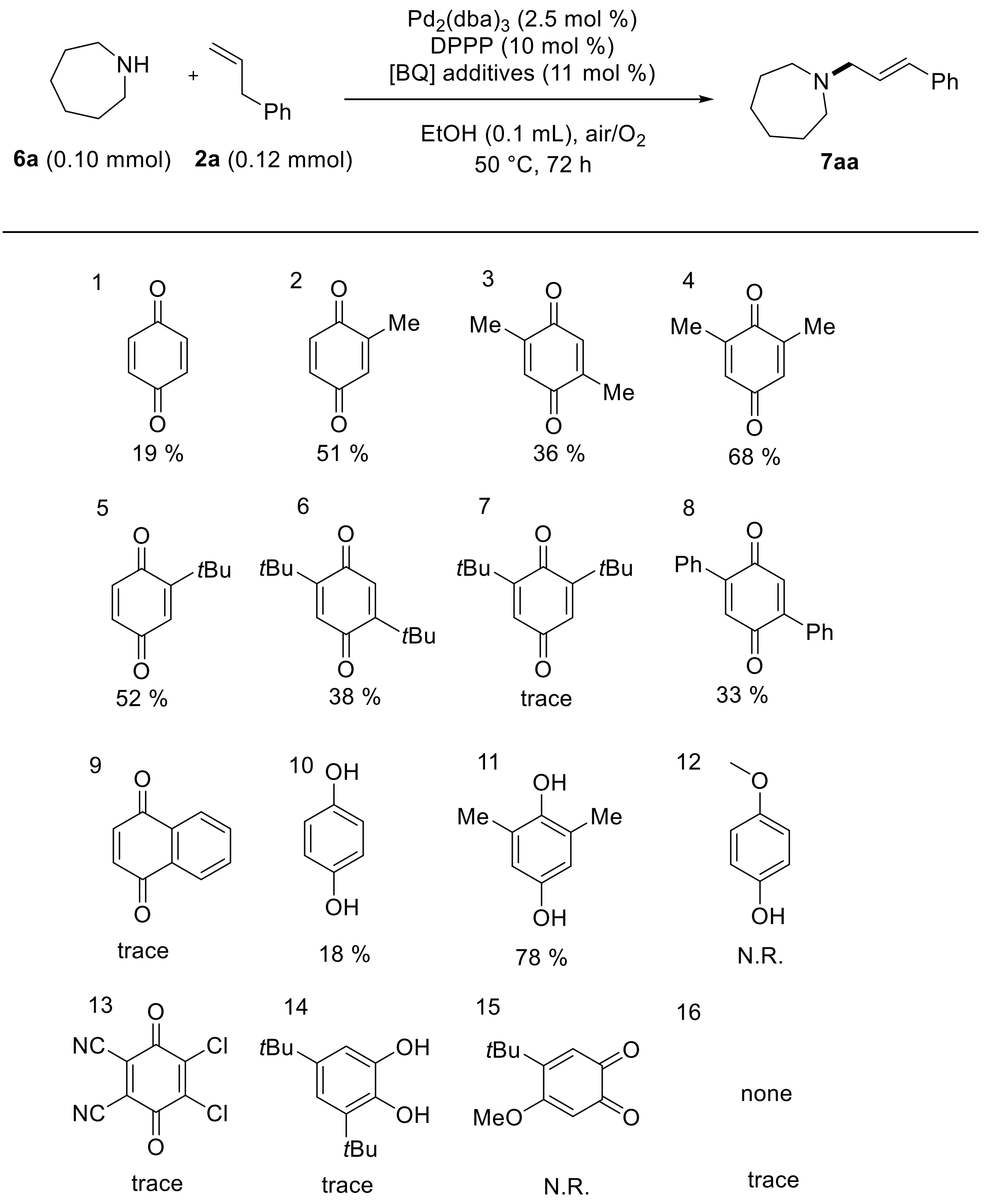

Figure 7 Screening of benzoquinones.

a) Reaction conditions: **6a** (0.10 mmol), **2a** (0.12 mmol), $Pd_2(dba)_3$ (2.5 mol %), DPPP (10 mol %), BQ addition (11 mol %). Before the reaction starts and midway after cooling, oxygen replacement is carried out for a period by connecting an oxygen balloon (every 18 hours). The reaction mixture is placed in a closed vessel with 0.1 mL of EtOH and heated at 50 °C for 72 hours. The yield is determined by NMR using CH_2Br_2 as an internal standard.

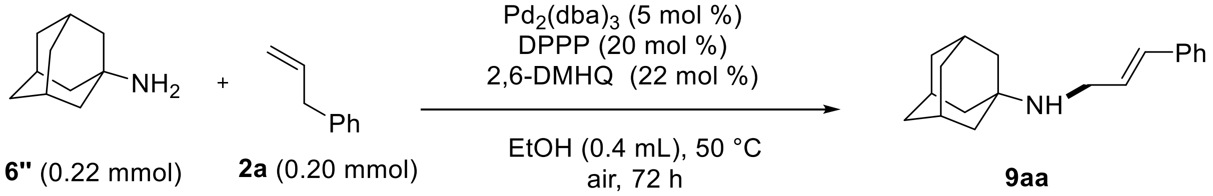

Entry	Conditions	Yield (%)[a]
1	standard conditions	~100 (94[b])
2	half of $Pd_2(dba)_3$/DPPP/DMHQ were used	38
3	EtOH (0.2 mL) was used	82

Figure 8 Alkyl primary amine as nucleophile.

a) Reaction conditions: **6"** (0.22 mmol), **2a** (0.20 mmol), $Pd_2(dba)_3$ (5 mol %), DPPP (10 mol %), 2,6-DMHQ (22 mol %). Before the reaction starts and midway after cooling, gas replacement is carried out for a period (every 5-6 hours). The reaction mixture is placed in a closed vessel with 0.4 mL of EtOH and heated at 50 °C for 72 hours. The yield is determined by NMR using CH_2Br_2 as an internal standard. b) Isolated yield.

3.3 Other allylic C–H functionalizations

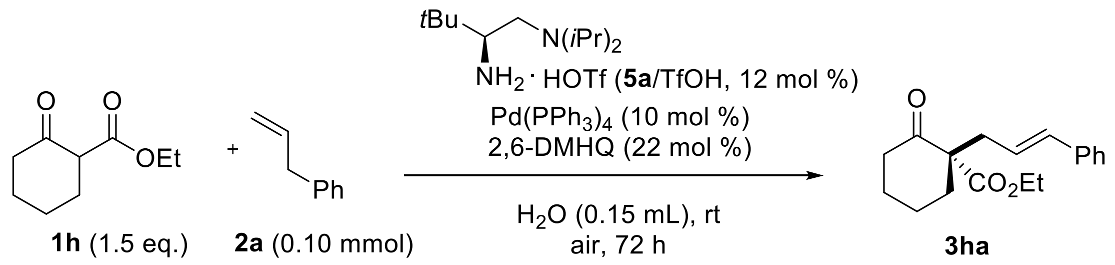

Entry	conditions	Results of **3**[a]
1	Standard	**3ha**: 83% (76%[b]), 93% *ee*
2	50% EtOH instead of H_2O	**3ha**: 79% (69%[b]), 87% *ee*
3	95% EtOH instead of H_2O	**3ha**: 85% (75%[b]), 80% *ee*
4	EtOH instead of H_2O	**3ha**: 92% (86%[b]), 74% *ee*
5	**1a** instead of **1g**	**3aa**: 88% (76%[b]), 78% *ee*
6	add Ir(ppy) (1 mol %), 30W Blue LED	**3ha-*Z***: 25%[b], 91% *ee*

Figure 9 Screening of asymmetric alkylation.

[a]Reaction conditions: **1h** (0.15 mmol), **2a** (0.10 mmol), **5a**/TfOH (12 mol %), $Pd(PPh_3)_4$ (10 mol %), 2,6-DMHQ (22 mol %), H_2O (0.15 mL),rt, 48 h, air; the yield was determined by ^1H NMR with CH_2Br_2 as internal standard; the *ee* was determined by HPLC. [b]Isolated yield.

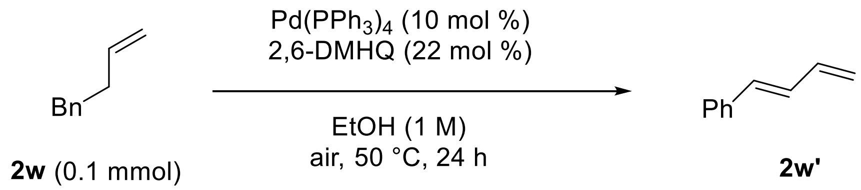

Entry	variation from standard condition	Yield of **2w'** (%)[a]
1	none	72 (92[b])

2	H_2O instead of EtOH	67
3	DCM instead of EtOH	22
4	rt instead of 50 °C	n.r.
5	2,6-DMBQ instead of 2,6-DMHQ	61

Figure 10 Screening of *α,β*-dehydrogenation.

[a]Reaction conditions: **2w** (0.1 mmol), $Pd(PPh_3)_4$ (10 mol %), 2,6-DMHQ (22 mol %), EtOH (0.1 mL), 50 °C, 24 h, air; the yield was determined by GC with tridecane as internal standard [b]48 h.

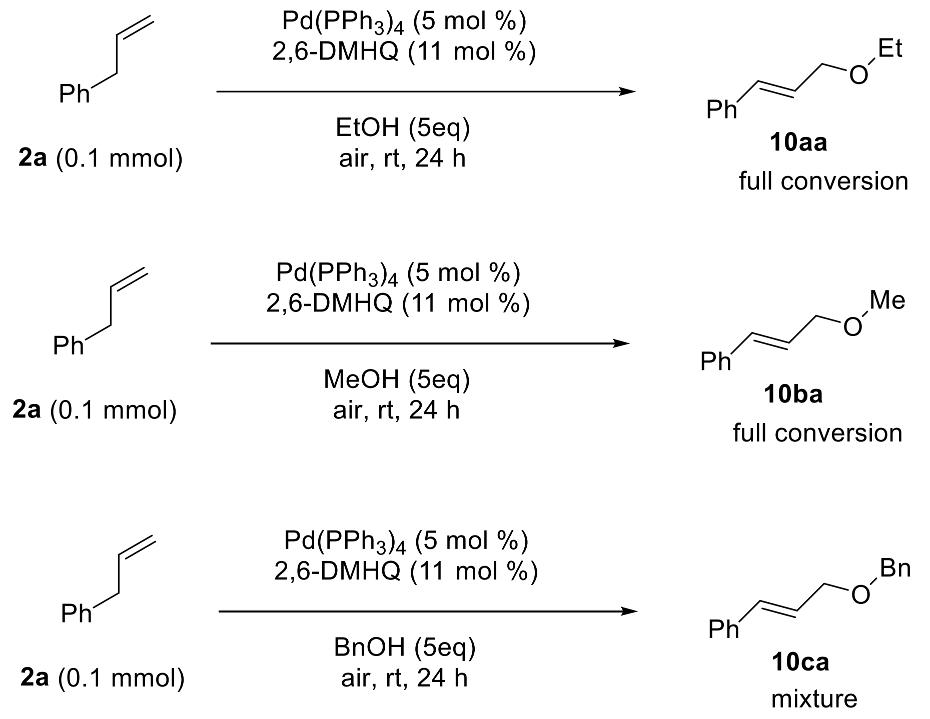

Figure 11 Allylic Etherification.

Reaction conditions: **2a** (0.1 mmol), ROH(0.5 mmol), $Pd(PPh_3)_4$ (5 mol %), 2,6-DMHQ (11 mol %), 50 °C, 24 h, air; the yield was determined by NMR with CH_2Br_2 as internal standard .

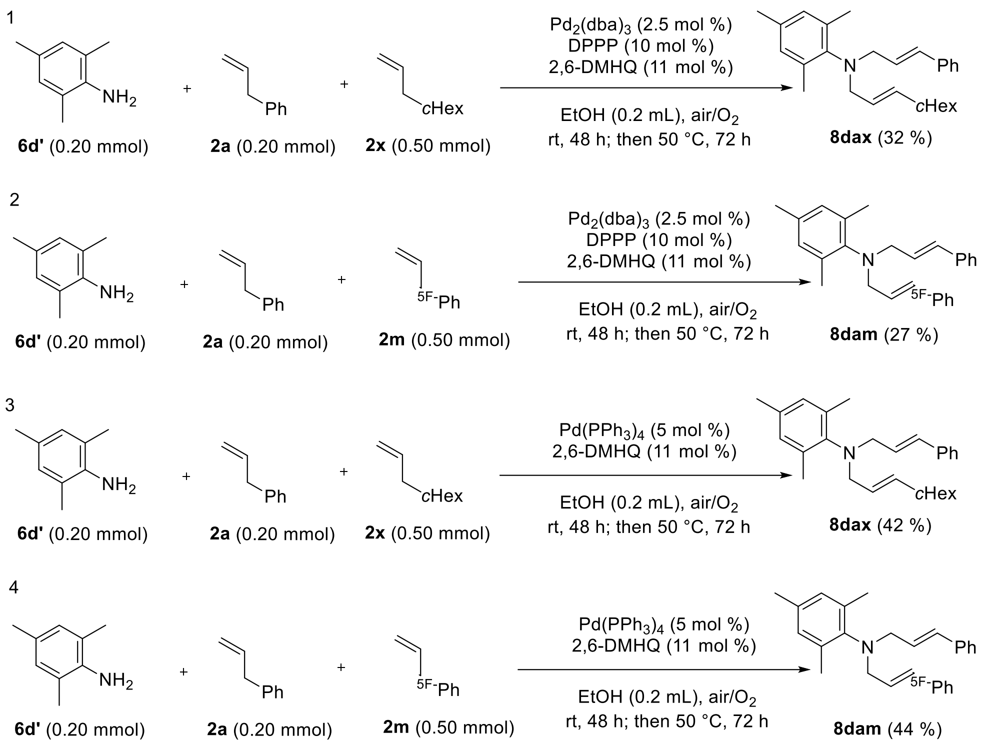

Figure 12 Sequential bis-allylic C–H amination.

3.4 Detailed Reaction Procedure within Green Paradigm

Procedure 1-1:On-water alkylation

Reaction Setup

Materials Preparation:

Reagents: 2,6-DMHQ, $Pd(PPh_3)_4$, nucleophilic reagent **1** , allylic substrate **2**.

Solvent: Water.

Equipment: 10 mL Schlenk tube, magnetic stirrer.

Procedure:

Step 1-1: In a glovebox, add 2,6-DMHQ, $Pd(PPh_3)_4$, nucleophilic reagent **1** and allylic substrate **2** sequentially into a 10 mL Schlenk tube equipped with a magnetic stirrer.

Step 1-2: Remove the Schlenk tube from the glovebox and add 0.2 mL of H_2O.

Step 1-3: Briefly expose the tube to air for several seconds to allow the reaction to proceed under aerobic conditions.

Step 1-4: Seal the tube and stir at room temperature for 48 hours. During this period, briefly expose the tube to air every 3-4 hours to maintain the aerobic environment.

Work-up and Purification

Step 2-1: Drying (not required): After the reaction, add anhydrous sodium sulfate to dry the mixture.

Step 2-2: Sampling and Separation: Sample the mixture multiple times with small amounts of EA.

Step 2-3: Use thin-layer chromatography (TLC) or column chromatography for **3** separation.

Note:

1. For Z- retentive productions, Ir(ppy)3 was added in step 1-1. Step1-4 was stir and Irradiated by a 30W Blue LED.
2. For Stereocontrol productions, **5a**/TfOH was added in step 1-1

Procedure 1-2: In-ethanol alkylation

Reaction Setup

Materials Preparation:

Reagents: 2,6-DMHQ, $Pd(PPh_3)_4$, nucleophilic reagent **1** , allylic substrate **2**.

Solvent: EtOH.

Equipment: 10 mL Schlenk tube, magnetic stirrer.

Procedure:

Step 1-1: In a glovebox, add 2,6-DMHQ, $Pd(PPh_3)_4$, nucleophilic reagent **1** and allylic substrate **2** sequentially into a 10 mL Schlenk tube equipped with a magnetic stirrer.

Step 1-2: Remove the Schlenk tube from the glovebox and add 0.2 mL of EtOH.

Step 1-3: Briefly expose the tube to air for several seconds to allow the reaction to proceed under aerobic conditions.

Step 1-4: Seal the tube and stir at room temperature for 48 hours. During this period, briefly expose the tube to air every 3-4 hours to maintain the aerobic environment.

Work-up and Purification

Step 2-1: 8 mL of ethyl acetate was added to dilute the mixture, which was then filtered through a thin layer of silica gel.

Step 2-2: The reaction tube and the silica gel layer were washed with ethyl acetate (8 mL × 4).

Step 2-3: After the organic solvent was rotary-evaporated off, the crude product was subjected to thin-layer

chromatography (TLC) or column chromatography for **3** separation.

Procedure 1-3: Gram scale alkylation

Reaction Setup

Materials Preparation:

Reagents: 2,6-DMHQ, $Pd(PPh_3)_4$, nucleophilic reagent **1a** , allylic substrate **2a**.

Solvent: EtOH.

Equipment: 50 mL round-bottom flask, magnetic stirrer.

Procedure:

Step 1-1: In a glovebox, add 2,6-DMHQ, $Pd(PPh_3)_4$, nucleophilic reagent **1a** and allylic substrate **2a** sequentially into a 50 mL round-bottom flask with a magnetic stirrer.

Step 1-2: Remove the round-bottom flask from the glovebox and add 10 mL of EtOH.

Step 1-3: Seal the flask with an oxygen balloon and stir at room temperature for 48 hours.

Work-up and Purification

Step 2-1: Remove the ethanol by rotary evaporation

Step 2-2: The crude product was subjected to column chromatography for **3aa** separation.

Procedure 2-1: General in-ethanol amination

Reaction Setup

Materials Preparation:

Reagents: 2,6-DMHQ, $Pd_2(dba)_3$, DPPP, allylic substrate **2**, nucleophilic reagent **6/6'/6"**.

Solvent: EtOH.

Equipment: 10 mL Schlenk tube, magnetic stirrer.

Procedure:

Step 1-1: In a glovebox, add 2,6-DMHQ, $Pd_2(dba)_3$, DPPP, allylic substrate **2** and nucleophilic reagent **6/6'/6"** sequentially into a 10 mL Schlenk tube equipped with a magnetic stirrer.

Step 1-2: Remove the Schlenk tube from the glovebox and add 0.2 mL of EtOH.

Step 1-3: Briefly expose the tube to air for several seconds to allow the reaction to proceed under aerobic conditions.

Step 1-4: Seal the tube and place it in a 50°C oil bath for the appropriate timeframe. During this period, briefly expose the tube to air every 4-8 hours to maintain the aerobic environment.

Work-up and Purification

Step 2-1: 8 mL of ethyl acetate was added to dilute the mixture which was then filtered through a thin layer of silica gel.

Step 2-2: The reaction tube and the silica gel layer were washed with ethyl acetate (8 mL × 4).

Step 2-3: After the organic solvent was rotary-evaporated off, the crude product was subjected to thin-layer chromatography (TLC) or column chromatography (triethylamine alkalization) for separation, yielding the desired product **7/8/9**.

Procedure 2-2: Sequential bis-allylic amination

Reaction Setup

Materials Preparation:

Reagents: 2,6-DMHQ, $Pd(PPh_3)_4$, allylic substrate **2a** and **2x** (or **2m**), nucleophilic reagent **6d'**.

Solvent: EtOH.

Equipment: 10 mL Schlenk tube, magnetic stirrer.

Procedure:

Step 1-1: In a glovebox, add 2,6-DMHQ, $Pd(PPh_3)_4$, allylic substrate **2a**, **2x** (or **2m**) and nucleophilic reagent **6d’** sequentially into a 10 mL Schlenk tube equipped with a magnetic stirrer.

Step 1-2: Remove the Schlenk tube from the glovebox and add 0.2 mL of EtOH.

Step 1-3: Briefly expose the tube to air for several seconds to allow the reaction to proceed under aerobic conditions.

Step 1-4: Seal the tube and stir at room temperature for 48 hours, then place it in a 50°C oil bath for 72 hours. During this period, cool the tube every 12 hours and then connect an oxygen balloon for several seconds to maintain the aerobic environment.

Work-up and Purification

Step 2-1: 8 mL of ethyl acetate was added to dilute the mixture which was then filtered through a thin layer of silica gel.

Step 2-2: The reaction tube and the silica gel layer were washed with ethyl acetate (8 mL × 4).

Step 2-3: After the organic solvent was rotary-evaporated off, the crude product was subjected to thin-layer chromatography (TLC) or column chromatography (triethylamine alkalization) for separation, yielding the desired product **8dax** (or **8dam**).

Procedure 2-3: General gram-scale amination

Reaction Setup

Materials Preparation:

Reagents: 2,6-DMHQ, $Pd_2(dba)_3$, DPPP, allylic substrate **2**, nucleophilic reagent **6**.

Solvent: EtOH.

Equipment: 38 mL Schlenk tube, magnetic stirrer.

Procedure:

Step 1-1: In a glovebox, add 2,6-DMHQ, $Pd_2(dba)_3$, DPPP, allylic substrate **2** and nucleophilic reagent **6** sequentially into a 38 mL Schlenk tube equipped with a magnetic stirrer.

Step 1-2: Remove the Schlenk tube from the glovebox and add 2 mL of EtOH.

Step 1-3: Connect an oxygen balloon and exchange the gas for several seconds.

Step 1-4: Seal the tube and place it in a 50°C oil bath for 84 hours. During this period, cool the tube every 12 hours and then connect an oxygen balloon for several seconds to maintain the aerobic environment.

Work-up and Purification

Step 2-1: 18 mL of ethyl acetate was added to dilute the mixture, which was then filtered through a thin layer of silica gel.

Step 2-2: The reaction tube and the silica gel layer were washed with ethyl acetate (28 mL × 4).

Step 2-3: After the organic solvent was rotary-evaporated off, the crude product was subjected to column chromatography (triethylamine alkalization) for separation, yielding the desired product **7**.

Procedure 2-4: Supplementary on-water amination

Reaction Setup

Materials Preparation:

Reagents: 2,6-DMHQ, $Pd(PPh_3)_4$, allylic substrate **2**, nucleophilic reagent **6/6’/6”**.

Solvent: H_2O.

Equipment: 10 mL Schlenk tube, magnetic stirrer.

Procedure:

Step 1-1: In a glovebox, add 2,6-DMHQ, $Pd(PPh_3)_4$, allylic substrate **2** and nucleophilic reagent **6/6'/6"** sequentially into a 10 mL Schlenk tube equipped with a magnetic stirrer.

Step 1-2: Remove the Schlenk tube from the glovebox and add 0.2 mL of H_2O.

Step 1-3: Briefly expose the tube to air for several seconds to allow the reaction to proceed under aerobic conditions.

Step 1-4: Seal the tube and stir at room temperature for 48 hours. During this period, briefly expose the tube to air every 3-4 hours to maintain the aerobic environment.

Work-up and Purification

Step 2-1: Drying (not required): After the reaction, add anhydrous sodium sulfate to dry the mixture.

Step 2-2: Sampling and Separation: Sample the mixture multiple times with small amounts of EA.

Step 2-3: The crude product was subjected to thin-layer chromatography (TLC) or column chromatography (triethylamine alkalization) for separation, yielding the desired product **7/8/9**.

3.5 **Characterization of alkylation products**

Ethyl (*E*)-2-acetyl-2-methyl-5-phenylpent-4-enoate **(3aa)**

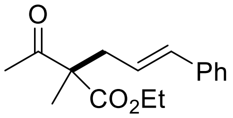

3aa

Links NMR CATALOG HPLC DETAILS

Following procedure 1-1, the reaction of substrate **2a** (0.2 mmol) for 48 hours afforded **3aa** in a yield of 44.8 mg (86%). Following procedure 1-1 (note 2), the reaction of substrate **2a** (0.1 mmol) for 48 hours afforded **3aa** in a yield of 19.8 mg (76%) with 78% *ee*; $[\alpha]_D^{25}$= -25.9 (c=0.20, $CHCl_3$). Following procedure 1-3, the reaction of substrate **2a** (10 mmol) was performed for 48 hours, yielding 2.06 g of product (81%).

^{1}H NMR (400 MHz, $CDCl_3$) δ 7.32-7.20 (m, 5H), 6.43 (d, *J* = 15.7 Hz, 1H), 6.05 (dt, *J* = 15.3, 7.5 Hz, 1H), 4.20 (q, *J* = 7.0 Hz, 2H), 2.79 (dd, *J* = 14.1, 7.3 Hz, 1H), 2.65 (dd, *J* = 14.1, 7.7 Hz, 1H), 2.18 (s, 3H), 1.38 (s, 3H), 1.26 (t, *J* = 7.1 Hz, 3H).

^{13}C NMR (101 MHz, $CDCl_3$) δ 205.2, 172.6, 137.2, 134.0, 128.6, 127.5, 126.3, 124.4, 61.5, 59.9, 38.7, 26.5, 19.3, 14.2.

HPLC analysis: Daicel Chiralpak OJ, iPrOH/hexane = 2%, flow rate = 1.0 mL/min, λ = 250 nm, 28.7 min (major), 42.6 min (minor).

HRMS (m/z, ESI-TOF) calcd for $C_{16}H_{21}O_3^+$ [M+H$^+$]: 261.1485, found 261.1485.

Ethyl (*Z*)-2-acetyl-2-methyl-5-phenylpent-4-enoate **(3aa-*Z*)**

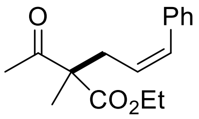

3aa-*Z*

Links NMR CATALOG DETAILS

Following procedure 1-1 (note 1), the reaction of substrate **2a** (0.2 mmol) for 48 hours afforded **3aa**-*Z* in a yield

of 32.7 mg (63%).

^{1}H NMR (400 MHz, $CDCl_3$) δ 7.36-7.27 (m, 3H), 7.26-7.20 (m, 2H), 6.56 (d, *J* = 11.7 Hz, 1H), 5.49 (dt, *J* = 11.9, 7.2 Hz, 1H), 2.88 (ddd, *J* = 23.5, 15.6, 8.3 Hz, 2H), 2.08 (s, 3H), 1.33 (s, 3H), 1.20 (t, *J* = 7.1 Hz, 3H).

^{13}C NMR (101 MHz, $CDCl_3$) δ 205.1, 172.6, 137.0, 132.4, 128.9, 128.4, 127.0, 126.0, 61.5, 59.7, 33.4, 26.2, 19.0, 14.1.

NOE (Nuclear Overhauser Effect) spectra exhibit significant peaks at 6.60-6.57 ppm and 5.53-5.49 ppm, indicating that the protons are in close spatial proximity. This spatial relationship is consistent with a *Z*-configuration (cis) for the alkene. The mutual correspondence of these two sets of peaks further supports the *Z*-configuration, as the protons are positioned on the same side of the double bond, leading to the observed NOE effects.

^{1}H-^{1}H COSY provides additional evidence for this configuration, showing cross-peaks between the protons at 6.60-6.57 ppm and 5.53-5.49 ppm, which indicates through-bond connectivity.

HRMS (m/z, ESI-TOF) calcd for $C_{16}H_{21}O_3^+$ [M+H$^+$]: 261.1485, found 261.1488.

Benzyl (*E*)-2-acetyl-2-methyl-5-phenylpent-4-enoate **(3ba)**

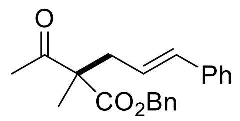

3ba

Links NMR DETAILS CATALOG

Following procedure 1-1, the reaction of substrate **2a** (0.2 mmol) for 48 hours afforded **3ba** in a yield of 53.3 mg (83%).

^{1}H NMR (400 MHz, $CDCl_3$) δ 7.33 (brs, 5H), 7.30-7.18 (m, 5H), 6.39 (d, *J* = 15.7 Hz, 1H), 5.98 (dt, *J* = 15.4, 7.5 Hz, 1H), 5.24-5.13 (m, 2H), 2.81 (dd, *J* = 14.1, 7.2 Hz, 1H), 2.64 (dd, *J* = 14.1, 7.8 Hz, 1H), 2.11 (s, 3H), 1.39 (s, 3H).

^{13}C NMR (101 MHz, $CDCl_3$) δ 205.1, 172.4, 137.1, 135.4, 134.2, 128.8, 128.6, 127.5, 126.4, 124.2, 67.3, 60.1, 38.8, 26.5, 19.3.

HRMS (m/z, ESI-TOF) calcd for $C_{21}H_{23}O_3^+$ [M+H$^+$]: 323.1642, found 323.1641.

Tert-butyl (*E*)-2-acetyl-2-methyl-5-phenylpent-4-enoate **(3ca)**

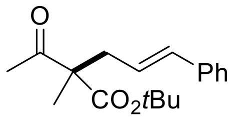

3ca

Links NMR CATALOG DETAILS

Following procedure 1-1, the reaction of substrate **2a** (0.2 mmol) for 48 hours afforded **3ca** in a yield of 46.6 mg (81%).

^{1}H NMR (400 MHz, $CDCl_3$) δ 7.32-7.28 (m, 4H), 7.22-7.19 (m, 1H), 6.43 (d, *J* = 15.7 Hz, 1H), 6.06 (dt, *J* = 15.4, 7.5 Hz, 1H), 2.75 (dd, *J* = 14.1, 7.4 Hz, 1H), 2.61 (dd, *J* = 14.1, 7.7 Hz, 1H), 2.18 (s, 3H), 1.45 (s, 9H), 1.34 (s, 3H).

^{13}C NMR (101 MHz, $CDCl_3$) δ 205.6, 171.7, 137.3, 133.9, 128.7, 127.5, 126.3, 124.7, 82.1, 60.5, 38.8, 28.0, 26.4, 19.3.

HRMS (m/z, ESI-TOF) calcd for $C_{18}H_{25}O_3^+$ [M+H$^+$]: 289.1798, found 289.1800.

Ethyl (*E*)-2-acetyl-2-ethyl-5-phenylpent-4-enoate **(3da)**

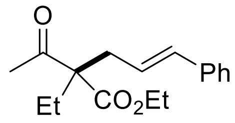

3da

Links NMR CATALOG DETAILS

Following procedure 1-1, the reaction of substrate **2a** (0.2 mmol) for 48 hours afforded **3da** in a yield of 42.0 mg (76%).

^{1}H NMR (400 MHz, $CDCl_3$) δ 7.34-7.27 (m, 4H), 7.23-7.18 (m, 1H), 6.43 (d, *J* = 15.7 Hz, 1H), 6.04-5.92 (m, 1H), 4.21 (q, *J* = 7.1 Hz, 2H), 2.75 (qdd, *J* = 14.6, 7.6, 2 Hz, 1H), 2.16 (s, 3H), 2.06-1.87 (m, 2H), 1.26 (t, *J* = 7.1 Hz, 3H), 0.83 (t, *J* = 6.8 Hz, 3H).

^{13}C NMR (101 MHz, $CDCl_3$) δ 205.0, 172.2, 137.2, 133.7, 128.6, 127.5, 126.3, 124.3, 64.2, 61.4, 34.8, 27.0, 24.8, 14.3, 8.3.

HRMS (m/z, ESI-TOF) calcd for $C_{17}H_{23}O_3^+$ [M+H$^+$]: 275.1642, found 275.1640.

3-acetyl-3-cinnamyldihydrofuran-2(3*H*)-one **(3ea)**

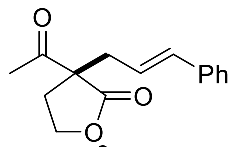

3ea

Links NMR CATALOG DETAILS

Following procedure 1-1, the reaction of substrate **2a** (0.2 mmol) for 48 hours afforded **3ea** in a yield of 42.1 mg (86%)

^{1}H NMR (400 MHz, $CDCl_3$) δ 7.35-7.28 (m, 4H), 7.25-7.21 (m, 1H), 6.49 (d, *J* = 15.7 Hz, 1H), 6.18-6.06 (m, 1H), 4.61 (d, *J* = 9.8 Hz, 1H), 4.28-4.18 (m, 3H), 4.05 (dd, *J* = 45.8, 17.2 Hz, 2H), 2.88 (ddd, *J* = 14.1, 6.8, 1.2 Hz, 1H), 2.63 (ddd, *J* = 14.1, 8.2, 1.0 Hz, 1H), 1.28 (t, *J* = 7.1 Hz, 3H).

^{13}C NMR (101 MHz, $CDCl_3$) δ 210.0, 168.7, 136.8, 135.0, 128.7, 127.8, 126.4, 123.2, 74.0, 71.1, 62.3, 59.8, 35.4, 14.3.

HRMS (m/z, ESI-TOF) calcd for $C_{15}H_{17}O_3^+$ [M+H$^+$]: 245.1172, found 245.1175.

(*E*)-2-acetyl-2-methyl-*N*,5-diphenylpent-4-enamide **(3fa)**

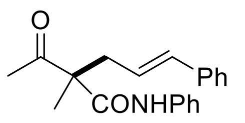

3fa

Links NMR CATALOG DETAILS

Following procedure 1-1, the reaction of substrate **2a** (0.2 mmol) for 48 hours afforded **3fa** in a yield of 57.3 mg (93%).

^{1}H NMR (400 MHz, $CDCl_3$) δ 8.10 (s, 1H), 7.51-7.48 (m, 2H), 7.35-7.26 (m, 7H), 7.24-7.19 (m, 1H), 7.15-7.10

(m, 1H), 6.49 (d, J = 15.7 Hz, 1H), 6.15-6.03 (m, 1H), 2.92-2.80 (m, 2H), 2.31 (s, 3H), 1.55 (s, 3H).
^{13}C NMR (101 MHz, $CDCl_3$) δ 210.0, 169.0, 137.6, 136.9, 134.6, 129.2, 128.7, 127.7, 126.4, 124.8, 124.0, 120.3, 60.5, 40.6, 27.4, 19.9.
HRMS (m/z, ESI-TOF) calcd for $C_{20}H_{22}NO_2^+$ [M+H$^+$]: 308.1645, found 308.1646.

Ethyl 1-cinnamyl-2-oxocyclopentane-1-carboxylate **(3ga)**

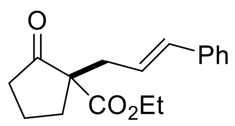

3ga

Links NMR CATALOG DETAILS

Following procedure 1-1, the reaction of substrate **2a** (0.2 mmol) for 48 hours afforded **3ga** in a yield of 52.8 mg (97%). Following procedure 1-2, the reaction of substrate **2a** (0.2 mmol) for 48 hours afforded **3ga** in a yield of 49.9 mg (91%).
^{1}H NMR (400 MHz, $CDCl_3$) δ 7.35-7.26 (m, 4H), 7.23-7.19 (m, 1H), 6.45 (d, J = 15.8 Hz, 1H), 6.16-6.03 (m, 1H), 4.23-4.14 (m, 2H), 2.82 (ddd, J = 13.9, 7.4, 1.2 Hz, 1H), 2.56-2.40 (m, 3H), 2.31-2.20 (m, 1H), 2.10-1.98 (m, 2H), 1.98-1.87 (m, 1H), 1.26 (t, J = 7.1 Hz, 3H).
^{13}C NMR (101 MHz, $CDCl_3$) δ 214.8, 171.1, 137.2, 134.2, 128.7, 127.6, 126.3, 124.7, 61.6, 60.4, 38.3, 37.2, 32.4, 19.7, 14.3.
HRMS (m/z, ESI-TOF) calcd for $C_{17}H_{21}O_3^+$ [M+H$^+$]: 273.1485, found 273.1487.

Ethyl 1-cinnamyl-2-oxocyclohexane-1-carboxylate **(3ha)**

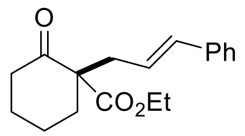

3ha

Links NMR CATALOG DETAILS HPLC E-Fact

Following procedure 1-1, the reaction of substrate **2a** (0.2 mmol) for 48 hours afforded **3ha** in a yield of 53.4 mg (93%); the reaction of substrate **2v** (0.2 mmol) for 48 hours afforded **3ha** in a yield of 6.9 mg (12%). Following procedure 1-1, the reaction of substrate **2a** (0.2 mmol) for 48 hours afforded **3ha** in a yield of 53.4 mg (93%). Following procedure 1-2 (note 2), the reaction of substrate **2a** (0.1 mmol) for 48 hours afforded **3ha** in a yield of 21.6 mg (76%) with 93% *ee*; $[\alpha]_D{}^{25}$= -56.1 (c=0.30, $CHCl_3$).
^{1}H NMR (400 MHz, $CDCl_3$) δ 7.35-7.28 (m, 4H), 7.24-7.19 (m, 1H), 6.40 (d, J = 15.8 Hz, 1H), 6.25-6.13 (m, 1H), 4.19 (q, J = 7.1 Hz, 2H), 2.77 (ddd, J = 13.8, 6.9, 1.2 Hz, 1H), 2.58-2.48 (m, 4H), 2.07-2.01 (m, 1H), 1.83-1.64 (m, 3H), 1.57-1.51 (m, 1H), 1.24 (t, J = 7.1 Hz, 1H).
^{13}C NMR (101 MHz, $CDCl_3$) δ 207.8, 171.7, 137.4, 133.4, 128.6, 127.4, 126.3, 125.3, 61.5, 61.5, 41.3, 38.8, 36.2, 27.7, 22.7, 14.3.
HPLC analysis: Daicel Chiralpak OJ, iPrOH/hexane = 2%, flow rate = 1.0 mL/min, λ = 254 nm, 27.2 min (minor), 48.9 min (major).
HRMS (m/z, ESI-TOF) calcd for $C_{17}H_{21}O_3^+$ [M+H$^+$]: 287.1639, found 287.1641.

Ethyl (*Z*)-2-oxo-1-(3-phenylallyl)cyclohexane-1-carboxylate **(3ha-*Z*)**

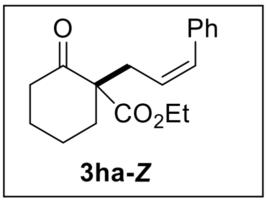

Links NMR CATALOG DETAILS HPLC

Following procedure 1-1 (note 1), the reaction of substrate **2a** (0.2 mmol) for 48 hours afforded **3ha-*Z*** in a yield of 29.0 mg (51%). Following procedure 1-1 (note 1 and note 2), the reaction of substrate **2a** (0.1 mmol) for 48 hours afforded **3ha-*Z*** in a yield of 7.2 mg (25%) with 91% *ee*; $[\alpha]_D^{25}$= -53.2 (c=0.20, $CHCl_3$).

^{1}H NMR (400 MHz, $CDCl_3$) δ 7.38-7.29 (m, 3H), 7.25-7.16 (m, 2H), 6.52 (d, *J* = 11.7 Hz, 1H), 5.60 (dt, *J* = 11.9, 7.3 Hz, 1H), 4.22-4.09 (m, 2H), 2.91 (dd, *J* = 15.1, 6.5 Hz, 1H), 2.67 (dd, *J* = 15.2, 7.7 Hz, 1H), 2.54-2.38 (m, 3H), 2.01-1.98 (m, 2H), 1.73-1.56 (m, 4H), 1.18 (t, *J* = 7.0 Hz, 3H).

^{13}C NMR (101 MHz, $CDCl_3$) δ 207.6, 171.6, 137.2, 131.7, 128.9, 128.3, 126.9, 126.7, 61.5, 61.0, 41.2, 35.8, 33.3, 27.7, 22.6, 14.2.

NOE (Nuclear Overhauser Effect) spectra exhibit significant peaks at 6.56-6.53 ppm and 5.64-5.61 ppm, indicating that the protons are in close spatial proximity. This spatial relationship is consistent with a *Z*-configuration (cis) for the alkene. The mutual correspondence of these two sets of peaks further supports the *Z*-configuration, as the protons are positioned on the same side of the double bond, leading to the observed NOE effects.

^{1}H-^{1}H COSY provides additional evidence for this configuration, showing cross-peaks between the protons at 6.56-6.53 ppm and 5.64-5.61 ppm, which indicates through-bond connectivity.

HPLC analysis: Daicel Chiralpak OJ, iPrOH/hexane = 2%, flow rate = 1.0 mL/min, λ = 251 nm, 15.2 min (minor), 18.7 min (major).

HRMS (m/z, ESI-TOF) calcd for $C_{17}H_{21}O_3^+$ [M+H$^+$]: 287.1639, found 287.1635.

Benzyl 1-cinnamyl-2-oxocyclohexane-1-carboxylate **(3ia)**

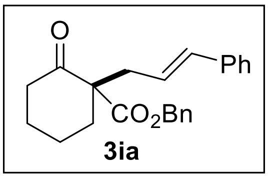

Links NMR CATALOG DETAILS

Following procedure 1-1, the reaction of substrate **2a** (0.2 mmol) for 48 hours afforded **3ia** in a yield of 61.3 mg (85%). Following procedure 1-2, the reaction of substrate **2a** (0.2 mmol) for 48 hours afforded **3ia** in a yield of 58.3 mg (81%).

^{1}H NMR (400 MHz, $CDCl_3$) δ 7.36-7.24 (m, 9H), 7.21-7.16 (m, 1H), 6.32 (d, *J* = 15.8 Hz, 1H), 6.13-6.03 (m, 1H), 5.13 (q, *J* = 12.2 Hz, 2H), 2.76 (dd, *J* = 13.9, 7.0 Hz, 1H), 2.59-2.40 (m, 4H), 2.00-1.99 (m, 1H), 1.74-1.64 (m, 3H), 1.53-1.49 (m, 1H).

^{13}C NMR (101 MHz, $CDCl_3$) δ 207.5, 171.6, 137.3, 135.4, 133.5, 128.7, 128.6, 127.4, 126.4, 125.1, 67.2, 61.6, 41.3, 38.7, 36.2, 27.7, 22.6.

HRMS (m/z, ESI-TOF) calcd for $C_{23}H_{25}O_3^+$ [M+H$^+$]: 349.1798, found 349.1800.

Tert-butyl 1-cinnamyl-2-oxocyclohexane-1-carboxylate **(3ja)**

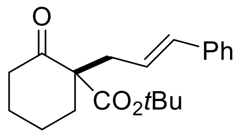

3ja

Links NMR CATALOG DETAILS

Following procedure 1-1, the reaction of substrate **2a** (0.2 mmol) for 48 hours afforded **3ja** in a yield of 43.4 mg (69%). Following procedure 1-2, the reaction of substrate **2a** (0.2 mmol) for 48 hours afforded **3ja** in a yield of 44.3 mg (71%).

^{1}H NMR (400 MHz, $CDCl_3$) δ 7.33-7.25 (m, 4H), 7,20-7.17 (m, 1H), 6.37 (d, J = 15.8 Hz, 1H), 6.25-6.12 (m, 1H), 2.76-2.67 (m, 1H), 2.56-2.39 (m, 4H), 2.08-1.99 (m, 1H), 1.79-1.44 (m, 4H), 1.41 (s, 9H).

^{13}C NMR (101 MHz, $CDCl_3$) δ 208.0, 170.8, 137.5, 133.2, 128.6, 127.3, 126.3, 125.6, 82.2, 61.9, 41.4, 39.0, 36.5, 28.1, 27.8, 22.8.

HRMS (m/z, ESI-TOF) calcd for $C_{20}H_{27}O_3^+$ [M+H$^+$]: 315.1955, found 315.1957.

Methyl 1-cinnamyl-2-oxocycloheptane-1-carboxylate **(3ka)**

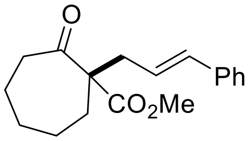

3ka

Links NMR DETAILS CATALOG

Following procedure 1-1, the reaction of substrate **2a** (0.2 mmol) for 48 hours afforded **3ka** in a yield of 49.3 mg (86%). Following procedure 1-2, the reaction of substrate **2a** (0.2 mmol) for 48 hours afforded **3ka** in a yield of 48.1 mg (84%).

^{1}H NMR (400 MHz, $CDCl_3$) δ 7.34-7.26 (m, 4H), 7.23-7.18 (m, 1H), 6.41 (d, J = 15.8 Hz, 1H), 6.21-6.06 (m, 1H), 3.71 (s, 3H), 2.93-2.84 (m, 1H), 2.71-2.65 (m, 1H), 2.56-2.45 (m, 2H), 2.22-2.11 (m, 1H), 1.91-1.82 (m, 1H), 1.81-1.61 (m, 5H), 1.48-1.41 (m, 1H).

^{13}C NMR (101 MHz, $CDCl_3$) δ 209.3, 172.7, 137.4, 133.7, 128.6, 127.4, 126.3, 125.4, 63.5, 52.4, 42.4, 39.2, 32.6, 30.1, 25.7, 24.9.

HRMS (m/z, ESI-TOF) calcd for $C_{18}H_{22}NaO_3^+$ [M+Na$^+$]: 309.1461, found 309.1464.

Ethyl 3-cinnamyl-4-oxotetrahydrofuran-3-carboxylate **(3la)**

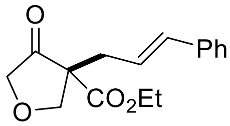

3la

Links NMR CATALOG DETAILS

Following procedure 1-1, the reaction of substrate **2a** (0.2 mmol) for 48 hours afforded **3la** in a yield of 23.6 mg (43%). Following procedure 1-2, the reaction of substrate **2a** (0.2 mmol) for 48 hours afforded **3la** in a yield of 47.3 mg (87%).

^{1}H NMR (400 MHz, $CDCl_3$) δ 7.35-7.28 (m, 4H), 7.25-7.21 (m, 1H), 6.49 (d, *J* = 15.7 Hz, 1H), 6.18-6.06 (m, 1H), 4.61 (d, *J* = 9.8 Hz, 1H), 4.28-4.18 (m, 3H), 4.05 (dd, *J* = 45.8, 17.2 Hz, 2H), 2.88 (ddd, *J* = 14.1, 6.8, 1.2 Hz, 1H), 2.63 (ddd, *J* = 14.1, 8.2, 1.0 Hz, 1H), 1.28 (t, *J* = 7.1 Hz, 3H).
^{13}C NMR (101 MHz, $CDCl_3$) δ 210.0, 168.7, 136.8, 135.0, 128.7, 127.8, 126.4, 123.2, 74.0, 71.1, 62.3, 59.8, 35.4, 14.3.
HRMS (m/z, ESI-TOF) calcd for $C_{16}H_{19}O_4^+$ [M+H$^+$]: 275.1278, found 275.1277.

Ethyl 1-benzyl-3-cinnamyl-4-oxopiperidine-3-carboxylate **(3ma)**

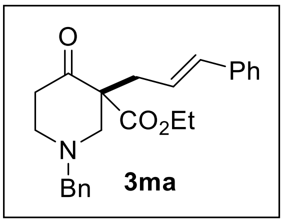

Links NMR CATALOG DETAILS

Following procedure 1-1, the reaction of substrate **1m** (0.2 mmol) for 48 hours afforded **3ma** in a yield of 69.3 mg (92%). Following procedure 1-2, the reaction of substrate **1m** (0.2 mmol) for 48 hours afforded **3ma** in a yield of 68.6 mg (91%).
^{1}H NMR (400 MHz, $CDCl_3$) δ 7.33-7.24 (m, 9H), 7.21-7.17 (m, 1H), 6.35 (d, *J* = 15.8 Hz, 1H), 6.22-6.13 (m, 1H), 4.25-4.09 (m, 21H), 3.59 (d, *J* = 1.3 Hz, 2H), 3.44 (dd, *J* = 11.6, 2.6 Hz, 1H), 3.04-2.97 (m, 1H), 2.89 (ddd, *J* = 15.2, 12.1, 6.6 Hz, 1H), 2.67 (dd, *J* = 13.3, 6.3 Hz, 1H), 2.53-2.38 (m, 3H), 2.32 (d, *J* = 11.6 Hz, 1H), 1.20 (t, *J* = 7.1 Hz, 3H).
^{13}C NMR (101 MHz, $CDCl_3$) δ 206.2, 171.3, 138.1, 137.4, 133.4, 129.0, 128.6, 128.4, 127.5, 127.4, 126.4, 125.1, 62.0, 61.8, 61.5, 61.1, 53.6, 40.7, 36.0, 14.3.
HRMS (m/z, ESI-TOF) calcd for $C_{24}H_{27}NNaO_3^+$ [M+Na$^+$]: 400.1883, found 400.1879.

1-(*tert*-butyl) 3-ethyl-3-cinnamyl-4-oxopiperidine-1,3-dicarboxylate **(3na)**

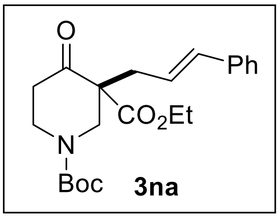

Links NMR CATALOG DETAILS

Following procedure 1-1, the reaction of substrate **2a** (0.2 mmol) for 48 hours afforded **3na** in a yield of 73.3 mg (95%). Following procedure 1-2, the reaction of substrate **2a** (0.2 mmol) for 48 hours afforded **3na** in a yield of 70.4 mg (91%).
^{1}H NMR (400 MHz, $CDCl_3$) δ 7.33-7.25 (m,4H), 7.22-7.19 (m, 1H), 6.43 (d, *J* = 15.8 Hz, 1H), 6.25-6.12 (m, 1H), 4.57 (brs, 1H), 4.17 (dd, *J* = 14.2, 7.1 Hz, 3H), 3.35-3.25 (m, 1H), 3.19 (d, *J* = 13.7 Hz, 1H), 2.80-2.64 (m, 2H), 2.60 (dd, *J* = 13.8, 7.7 Hz, 1H), 2.50-2.46 (m, 1H), 1.48 (s, 9H), 1.23 (t, *J* = 7.1 Hz, 3H).
^{13}C NMR (101 MHz, $CDCl_3$) δ 204.9, 137.1, 134.3, 128.6, 127.6, 126.4, 124.1, 80.7, 61.9, 61.6, 40.1, 35.7, 28.4, 14.3.
HRMS (m/z, ESI-TOF) calcd for $C_{22}H_{30}NO_5^+$ [M+H$^+$]: 388.2118, found 388.2119.

1-benzyl 3-(*tert*-butyl)-3-cinnamyl-4-oxopiperidine-1,3-dicarboxylate **(3oa)**

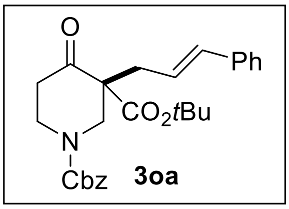

Links NMR CATALOG DETAILS

Following procedure 1-1, the reaction of substrate **2a** (0.2 mmol) for 48 hours afforded **3oa** in a yield of 71.0 mg (79%). Following procedure 1-2, the reaction of substrate **2a** (0.2 mmol) for 48 hours afforded **3oa** in a yield of 64.5 mg (72%).

^{1}H NMR (400 MHz, $CDCl_3$) δ 7.38-7.27 (m,9H), 7.23-7.17 (m, 1H), 6.42 (d, *J* = 15.8 Hz, 1H), 6.25-6.12 (m, 1H), 5.17 (q, *J* = 12.5 Hz, 2H), 4.65 (brs, 1H), 4.24 (brs, 1H), 3.39-3.28 (m, 1H), 3.14 (brs, 1H), 2.84–2.65 (m, 2H), 2.59-2.44 (m, 2H), 1.37 (s, 9H).

^{13}C NMR (101 MHz, $CDCl_3$) δ 205.2, 137.2, 134.0, 128.6, 127.5, 126.3, 124.4, 82.8, 80.6, 62.0, 40.2, 35.8, 28.5, 28.0.

HRMS (m/z, ESI-TOF) calcd for $C_{27}H_{31}NNaO_5^+$ [M+Na$^+$]: 472.2094, found 472.2091.

Di-*tert*-butyl 3-cinnamyl-4-oxopiperidine-1,3-dicarboxylate **(3pa)**

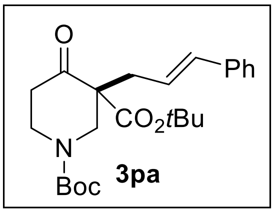

Links NMR CATALOG DETAILS

Following procedure 1-1, the reaction of substrate **2a** (0.2 mmol) for 48 hours afforded **3pa** in a yield of 59.0 mg (71%). Following procedure 1-2, the reaction of substrate **2a** (0.2 mmol) for 48 hours afforded **3pa** in a yield of 60.6 mg (73%).

^{1}H NMR (400 MHz, $CDCl_3$) δ 7.34-7.25 (m, 4H), 7.22-7.18 (m, 1H), 6.42 (d, J = 15.8 Hz, 1H), 6.27-6.15 (m, 1H), 4.67-4.55 (m, 1H), 4.25-4.07 (m, 1H), 3.32-3.16 (m, 1H), 3.06 (brs, 1H), 2.80-2.62 (m, 2H), 2.57-2.41 (m, 2H), 1.48 (s, 9H), 1.41 (s, 9H).

^{13}C NMR (101 MHz, $CDCl_3$) δ 205.2, 137.2, 134.0, 128.6, 127.5, 126.3, 124.4, 82.8, 80.6, 62.0, 40.2, 35.8, 28.5, 28.0.

HRMS (m/z, ESI-TOF) calcd for $C_{24}H_{33}NNaO_5^+$ [M+Na$^+$]: 438.2251, found 438.2246.

1-(*tert*-butyl) 4-ethyl 4-cinnamyl-3-oxopiperidine-1,4-dicarboxylate **(3qa)**

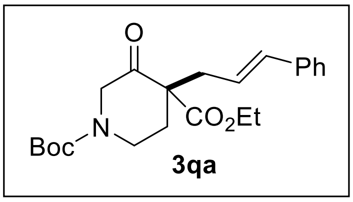

Links NMR CATALOG DETAILS

Following procedure 1-1, the reaction of substrate **2a** (0.2 mmol) for 48 hours afforded **3qa** in a yield of 63.6 mg

(82%). Following procedure 1-2, the reaction of substrate **2a** (0.2 mmol) for 48 hours afforded **3qa** in a yield of 62.6 mg (81%).

^{1}H NMR (400 MHz, $CDCl_3$) δ 7.35-7.28 (m,4H), 7.23-7.19 (m, 1H), 6.41 (d, *J* = 15.8 Hz, 1H), 6.22-6.10 (m, 1H), 4.48-4.22 (m, 1H), 4.14 (dt, *J* = 12.8, 6.5 Hz, 2H), 3.96-3.42 (m, 4H), 2.72 (dd, *J* = 13.8, 7.1 Hz, 1H), 2.66-2.50 (m, 2H), 1.45 (s, 9H), 1.21 (t, *J* = 7.1 Hz, 3H).

^{13}C NMR (101 MHz, $CDCl_3$) δ 170.1, 137.1, 134.2, 128.7, 127.6, 126.4, 124.2, 80.8, 61.9, 58.3, 37.4, 30.5, 28.4, 14.3.

HRMS (m/z, ESI-TOF) calcd for $C_{22}H_{30}NO_5^+$ [M+H$^+$]: 388.2118, found 388.2117.

1-(*tert*-butyl) 4-ethyl 4-cinnamyl-5-oxoazepane-1,4-dicarboxylate **(3ra)**

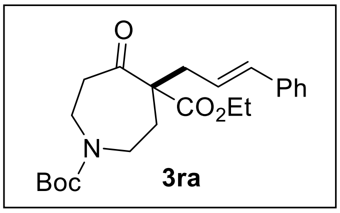

3ra

Links NMR CATALOG DETAILS

Following procedure 1-1, the reaction of substrate **2a** (0.2 mmol) for 48 hours afforded **3ra** in a yield of 65.7 mg (82%). Following procedure 1-2, the reaction of substrate **2a** (0.2 mmol) for 48 hours afforded **3ra** in a yield of 61.8 mg (77%).

^{1}H NMR (400 MHz, $CDCl_3$) δ 7.31-7.28 (m, 4H), 7.22-7.19 (m, 1H), 6.39 (d, *J* = 15.7 Hz, 3H), 6.15-5.98 (m, 1H), 4.30-4.17 (m, 3H), 3.75-3.70 (m, 3H), 3.52-3.36 (m, 2H), 2.92-2.82 (m, 2H), 2.60-2.50 (m, 1H), 2.22 (d, *J* = 14.9 Hz, 1H), 1.44 (s, 9H), 1.24 (t, *J* = 7.1 Hz, 3H).

^{13}C NMR (101 MHz, $CDCl_3$) δ 206.1, 171.6, 154.6, 137.2, 133.9, 128.6, 127.5, 126.3, 124.7, 80.3, 63.0, 61.7, 61.6, 59.0, 40.4, 34.2, 28.6, 28.5, 14.3.

HRMS (m/z, ESI-TOF) calcd for $C_{23}H_{32}NO_5^+$ [M+H$^+$]: 402.2275, found 402.2274.

(*E*)-2-methyl-2,5-diphenylpent-4-enal **(3sa)**

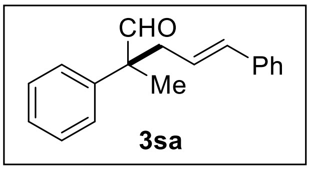

3sa

Links NMR CATALOG DETAILS

Following procedure 1-1, the reaction of substrate **2a** (0.2 mmol) for 48 hours afforded **3sa** in a yield of 44.7 mg (89%).

^{1}H NMR (400 MHz, $CDCl_3$) δ 9.57 (s, 1H), 7.44-7.26 (m, 10H), 6.40 (d, *J* = 15.8 Hz, 1H), 5.98-5.89 (m, 1H), 2.88-2.73 (m, 2H), 1.49 (s, 3H).

^{13}C NMR (101 MHz, $CDCl_3$) δ 202.1, 139.6, 137.4, 133.7, 129.1, 128.6, 127.6, 127.4, 127.3, 126.3, 125.1, 54.3, 40.1, 19.1.

HRMS (m/z, ESI-TOF) calcd for $C_{18}H_{19}O^+$ [M+H$^+$]: 251.1430, found 251.1434.

Methyl (*E*)-2-formyl-2,5-diphenylpent-4-enoate **(3ta)**

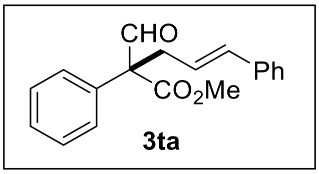

Links NMR CATALOG DETAILS

Following procedure 1-1, the reaction of substrate **2a** (0.2 mmol) for 48 hours afforded **3ta** in a yield of 27.8 mg (47%).

^{1}H NMR (400 MHz, $CDCl_3$) δ 9.93 (s, 1H), 7.45-7.33 (m, 4H), 7.27-7.19 (m, 4H), 6.43 (d, *J* = 15.8 Hz, 1H), 6.17-6.06 (m, 1H), 3.79 (s, 3H), 3.27 (ddd, *J* = 13.8, 6.5, 1.1 Hz, 1H), 3.01 (dd, *J* = 13.8, 8.2 Hz, 1H).

^{13}C NMR (101 MHz, $CDCl_3$) δ 196.4, 171.3, 135.1, 134.4, 130.2, 129.3, 129.0, 128.6, 127.5, 127.4, 126.3, 124.3, 66.6, 52.9, 36.2.

HRMS (m/z, ESI-TOF) calcd for $C_{19}H_{19}O_3^+$ [M+H$^+$]: 295.1329, found 295.1324.

Ethyl (*E*)-1-(3-(4-ethylphenyl)allyl)-2-oxocyclohexane-1-carboxylate **(3hb)**

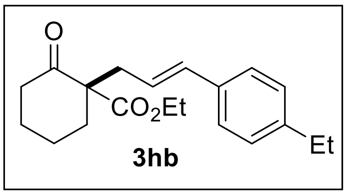

Links NMR CATALOG DETAILS

Following procedure 1-1, the reaction of substrate **2b** (0.2 mmol) for 48 hours afforded **3hb** in a yield of 55.9 mg (89%).

^{1}H NMR (400 MHz, $CDCl_3$) δ 7.24 (d, *J* = 7.9 Hz, 2H), 7.11 (d, *J* = 7.9 Hz, 2H), 6.35 (d, *J* = 15.8 Hz, 1H), 6.15-6.05 (m, 1H), 4.17 (q, *J* = 7.1 Hz, 2H), 2.72 (dd, *J* = 13.9, 7.0 Hz, 1H), 2.66-2.59 (m, 2H), 2.55-2.45 (m, 4H), 2.04-2.00 (m, 1H), 1.81-1.47 (m, 4H), 1.23-1.19 (m, 6H).

^{13}C NMR (101 MHz, $CDCl_3$) δ 207.8, 171.7, 143.5, 134.9, 133.2, 128.1, 126.3, 124.2, 77.2, 61.5, 61.4, 41.3, 38.8, 36.1, 28.7, 27.7, 22.7, 15.7, 14.4.

HRMS (m/z, ESI-TOF) calcd for $C_{20}H_{27}O_3^+$ [M+H$^+$]: 315.1955, found 315.1956.

Ethyl (*E*)-1-(3-(3,4-dimethylphenyl)allyl)-2-oxocyclohexane-1-carboxylate **(3hc)**

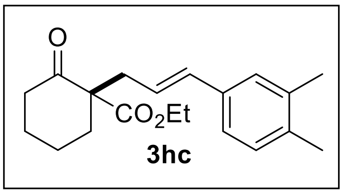

Links NMR CATALOG DETAILS

Following procedure 1-1, the reaction of substrate **2c**(0.2 mmol) for 48 hours afforded **3hc** in a yield of 51.5 mg (82%).

^{1}H NMR (400 MHz, $CDCl_3$) δ 7.10 (s, 1H), 7.07-7.01 (m, 2H), 6.31 (d, *J* = 15.8 Hz, 1H), 6.16-6.04 (m, 1H), 4.17 (q, *J* = 7.1 Hz, 2H), 2.71 (dd, *J* = 13.8, 7.0 Hz, 1H), 2.56-2.44 (m, 4H), 2.23 (s, 3H), 2.22 (s, 3H), 2.07-1.99 (m, 1H), 1.80-1.47 (m, 4H), 1.22 (t, *J* = 7.1 Hz, 3H).

^{13}C NMR (101 MHz, $CDCl_3$) δ 207.9, 171.8, 136.7, 135.8, 135.0, 133.3, 129.8, 127.5, 124.0, 123.8, 61.5, 61.4, 41.3, 38.8, 36.1, 27.7, 22.7, 19.9, 19.6, 14.3.

HRMS (m/z, ESI-TOF) calcd for $C_{20}H_{27}O_3^+$ [M+H$^+$]: 315.1955, found 315.1958.

Ethyl (*E*)-1-(3-(2,4,6-trimethoxyphenyl)allyl)-2-oxocyclohexane-1-carboxylate **(3hd)**

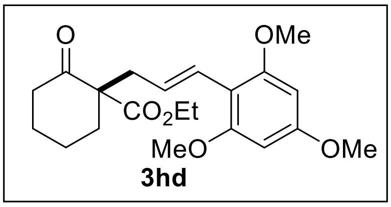

Links NMR CATALOG DETAILS

Following procedure 1-1, the reaction of substrate **2d** (0.2 mmol) for 48 hours afforded **3hd** in a yield of 59.4 mg (79%).

^{1}H NMR (400 MHz, $CDCl_3$) δ 6.54 (s, 1H), 6.29 (d, J = 15.7 Hz, 1H), 6.14-6.04 (m, 1H), 4.21-4.16 (m, 2H), 3.86 (s, 6H), 3.83 (s, 3H), 2.71 (dd, J = 13.9, 6.9 Hz, 1H), 2.57-2.46 (m, 4H), 2.07-2.00 (m, 1H), 1.83-1.50 (m, 4H), 1.23 (t, J = 7.1 Hz, 3H).

^{13}C NMR (101 MHz, $CDCl_3$) δ 207.9, 171.7, 153.4, 137.7, 133.3, 133.1, 124.7, 103.4, 61.5, 61.4, 61.0, 56.2, 41.3, 38.8, 36.2, 27.6, 22.7, 14.3.

HRMS (m/z, ESI-TOF) calcd for $C_{21}H_{29}O_6^+$ [M+H$^+$]: 377.1959, found 377.1955.

Ethyl (*E*)-1-(3-(3,4-dimethoxyphenyl)allyl)-2-oxocyclohexane-1-carboxylate **(3he)**

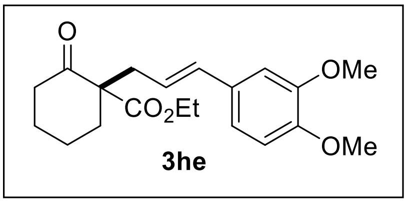

Links NMR CATALOG DETAILS

Following procedure 1-1, the reaction of substrate **2e** (0.2 mmol) for 48 hours afforded **3he** in a yield of 56.5 mg (82%).

^{1}H NMR (400 MHz, $CDCl_3$) δ 6.87-6.83 (m, 2H), 6.78 (d, J = 8.2 Hz, 1H), 6.30 (d, J = 15.8 Hz, 1H), 6.12-5.98 (m, 1H), 4.17 (q, J = 7.1 Hz, 2H), 3.89 (s, 3H), 3.86 (s, 3H), 2.72 (ddd, J = 13.8, 7.0, 1.1 Hz, 1H), 2.57-2.42 (m, 4H), 2.06-1.97 (m, 1H), 1.80-1.62 (m, 3H), 1.57-1.49 (m, 1H), 1.21 (t, J = 7.1 Hz, 3H).

^{13}C NMR (101 MHz, $CDCl_3$) δ 207.8, 171.7, 149.1, 148.7, 133.0, 130.6, 123.2, 119.3, 111.3, 108.9, 61.5, 61.3, 56.0, 56.0, 41.3, 38.8, 36.2, 27.6, 22.7, 14.3.

HRMS (m/z, ESI-TOF) calcd for $C_{20}H_{27}O_6^+$ [M+H$^+$]: 347.1853, found 347.1853.

Ethyl (*E*)-1-(3-(4-methoxyphenyl)allyl)-2-oxocyclohexane-1-carboxylate **(3hf)**

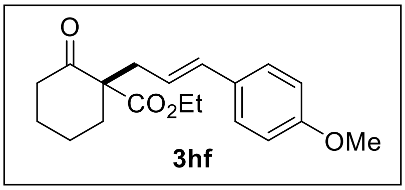

Links NMR CATALOG DETAILS

Following procedure 1-1, the reaction of substrate **2f** (0.2 mmol) for 48 hours afforded **3hf** in a yield of 56.0 mg (89%).

^{1}H NMR (400 MHz, $CDCl_3$) δ 7.19 (t, J = 7.9 Hz, 1H), 6.91 (d, J = 7.6 Hz, 1H), 6.85 (s, 1H), 6.76 (dd, J = 8.2, 2.3 Hz, 1H), 6.34 (d, J = 15.8 Hz, 1H), 6.23-6.12 (m, 1H), 4.17 (q, J = 7.1 Hz, 2H), 3.80 (s, 3H), 2.73 (dd, J = 13.9, 6.9 Hz, 1H), 2.54-2.47 (m, 4H), 2.07-1.96 (m, 1H), 1.80-1.62 (m, 3H), 1.56-1.48 (m, 1H), 1.22 (t, J = 7.1 Hz, 3H).
^{13}C NMR (101 MHz, $CDCl_3$) δ 207.8, 171.7, 159.9, 138.9, 133.3, 129. 6, 125.7, 119.0, 113.1, 111.6, 61.5, 61.5, 55.3, 41.3, 38.8, 36.2, 27.7, 22.7, 14.4.
HRMS (m/z, ESI-TOF) calcd for $C_{19}H_{25}O_4^+$ [M+H$^+$]: 317.1747, found 317.1748.

Ethyl (*E*)-1-(3-(3-(benzyloxy)phenyl)allyl)-2-oxocyclohexane-1-carboxylate **(3hg)**

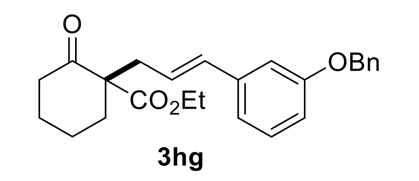

Links NMR CATALOG DETAILS

Following procedure 1-1, the reaction of substrate **2g** (0.2 mmol) for 48 hours afforded **3hg** in a yield of 70.8 mg (90%).
^{1}H NMR (400 MHz, $CDCl_3$) δ 7.44 (d, J = 7.2 Hz, 2H), 7.39 (t, J = 7.4 Hz, 2H), 7.32 (t, J = 7.1 Hz, 1H), 7.19 (t, J = 7.9 Hz, 1H), 6.96-6.90 (m, 2H), 6.84-6.81 (m, 1H), 6.34 (d, J = 15.8 Hz, 1H), 6.22-6.12 (m, 1H), 5.05 (s, 2H), 4.16 (q, J = 7.1 Hz, 2H), 2.73 (dd, J = 13.9, 6.9 Hz, 1H), 2.54-2.46 (m, 4H), 2.08-1.99 (m, 1H), 1.78-1.60 (m, 3H), 1.59-1.45 (m, 1H), 1.21 (t, J = 7.1 Hz, 3H).
^{13}C NMR (101 MHz, $CDCl_3$) δ 207.8, 171.7, 159.1, 138.9, 137.2, 133.2, 129.6, 128.7, 128.1, 127.7, 125.7, 119.3, 113.8, 112.7, 70.1, 61.5, 61.5, 41.3, 38.7, 36.2, 27.7, 22.7, 14.4.
HRMS (m/z, ESI-TOF) calcd for $C_{25}H_{28}NaO_4^+$ [M+Na$^+$]: 415.1880, found 415.1881.

Ethyl (*E*)-1-(3-(2-hydroxyphenyl)allyl)-2-oxocyclohexane-1-carboxylate **(3hh)**

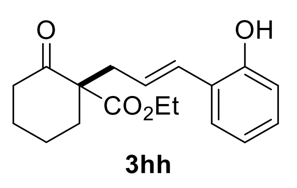

Links NMR CATALOG DETAILS

Following procedure 1-1, the reaction of substrate **2h** (0.2 mmol) for 48 hours afforded **3hh** in a yield of 49.9 mg (83%).
^{1}H NMR (400 MHz, $CDCl_3$) δ 7.27-7.23 (m, 2H), 7.11-7.06 (m, 1H), 6.85 (t, J = 7.4 Hz, 1H), 6.79 (d, J = 8.0 Hz, 1H), 6.55 (d, J = 16.0 Hz, 1H), 6.17-6.05 (m, 1H), 5.51 (brs, 1H), 4.19 (q, J = 7.1 Hz, 2H), 2.79 (dd, J = 13.9, 7.3 Hz, 1H), 2.60-2.45 (m, 4H), 2.07-2.00 (m, 1H), 1.79-1.63 (m, 3H), 1.57-1.48 (m, 1H), 1.24 (t, J = 7.1 Hz, 3H).
^{13}C NMR (101 MHz, $CDCl_3$) δ 207.9, 171.9, 153.0, 128.5, 128.0, 127.8, 127.7, 124.6, 120.8, 116.0, 61.8, 61.6, 41.4, 39.0, 36.3, 27.7, 22.7, 14.3.
HRMS (m/z, ESI-TOF) calcd for $C_{18}H_{23}O_4^+$ [M+H$^+$]: 303.1591, found 303.1591.

Ethyl (*E*)-1-(3-([1,1'-biphenyl]-4-yl)allyl)-2-oxocyclohexane-1-carboxylate **(3hi)**

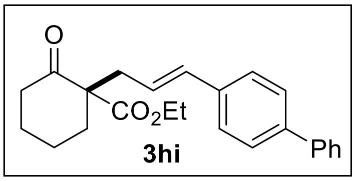

Links NMR CATALOG DETAILS

Following procedure 1-1, the reaction of substrate **2i** (0.2 mmol) for 48 hours afforded **3hi** in a yield of 63.4 mg (88%).

^{1}H NMR (400 MHz, $CDCl_3$) δ 7.58 (d, *J* = 7.6 Hz, 2H), 7.52 (d, *J* = 8.2 Hz, 2H), 7.45-7.38 (m, 4H), 7.33 (t, *J* = 7.3 Hz, 1H), 6.41 (d, *J* = 15.8 Hz, 1H), 6.30-6.18 (m, 1H), 4.18 (q, *J* = 7.1 Hz, 2H), 2.76 (dd, *J* = 13.9, 6.9 Hz, 1H), 2.56-2.48 (m, 4H), 2.09-1.99 (m, 1H), 1.81-1.49 (m, 4H), 1.23 (t, *J* = 7.1 Hz, 3H).

^{13}C NMR (101 MHz, $CDCl_3$) δ 207.8, 171.7, 140.9, 140.1, 136.4, 132.9, 128.9, 127.4, 127.3, 127.0, 126.7, 125.5, 61.5, 61.5, 41.4, 38.9, 36.2, 27.7, 22.7, 14.4.

HRMS (m/z, ESI-TOF) calcd for $C_{24}H_{27}O_3^+$ [M+H$^+$]: 363.1955, found 363.1956.

Ethyl (*E*)-2-oxo-1-(3-(4-vinylphenyl)allyl)cyclohexane-1-carboxylate **(3hj)**

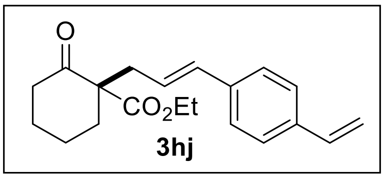

Links NMR CATALOG DETAILS

Following procedure 1-1, the reaction of substrate **2j** (0.2 mmol) for 48 hours afforded **3hj** in a yield of 49.4 mg (79%).

^{1}H NMR (400 MHz, $CDCl_3$) δ 7.32 (d, *J* = 8.3 Hz, 2H), 7.27 (d, *J* = 8.4 Hz, 2H), 6.68 (dd, *J* = 17.6, 10.9 Hz, 1H), 6.35 (d, *J* = 15.8 Hz, 1H), 6.24-6.13 (m, 1H), 5.72 (d, *J* = 17.6 Hz, 1H), 5.21 (d, *J* = 10.9 Hz, 1H), 4.16 (q, *J* = 7.1 Hz, 2H), 2.74 (dd, *J* = 13.9, 6.9 Hz, 1H), 2.61-2.42 (m, 4H), 2.09-1.98 (m, 1H), 1.82-1.47 (m, 4H), 1.21 (t, *J* = 7.1 Hz, 3H).

^{13}C NMR (101 MHz, $CDCl_3$) δ 207.8, 171.7, 136.9, 136.7, 136.6, 132.9, 126.5, 126.4, 125.4, 113.6, 61.5, 61.4, 41.3, 38.9, 36.3, 27.7, 22.7, 14.3.

HRMS (m/z, ESI-TOF) calcd for $C_{20}H_{25}O_3^+$ [M+H$^+$]: 313.1798, found 313.1800.

Ethyl (*E*)-1-(3-(3-cyanophenyl)allyl)-2-oxocyclohexane-1-carboxylate **(3hk)**

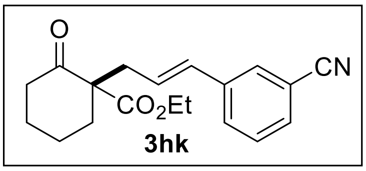

Links NMR CATALOG DETAILS

Following procedure 1-1, the reaction of substrate **2k** (0.2 mmol) for 48 hours afforded **3hk** in a yield of 52.6 mg (85%).

^{1}H NMR (400 MHz, $CDCl_3$) δ 7.30 (s, 1H), 7.23-7.15 (m, 3H), 6.31 (d, *J* = 15.9 Hz, 1H), 6.25-6.13 (m, 1H), 4.24-4.10 (m, 2H), 2.74 (dd, *J* = 14.0, 6.7 Hz, 1H), 2.57-2.44 (m, 4H), 2.07-2.00 (m, 1H), 1.82-1.47 (m, 4H), 1.22 (t, *J* = 7.1 Hz, 3H).

^{13}C NMR (101 MHz, $CDCl_3$) δ 207.7, 171.6, 139.2, 134.6, 132.0, 129.8, 127.3, 127.1, 126.2, 124.5, 61.5, 61.5, 41.3, 38.7, 36.3, 27.7, 22.7, 14.4.

HRMS (m/z, ESI-TOF) calcd for $C_{19}H_{22}NO_3^+$ [M+H$^+$]: 312.1594, found 312.1595.

Ethyl (*E*)-1-(3-(4-cyanophenyl)allyl)-2-oxocyclohexane-1-carboxylate **(3hl)**

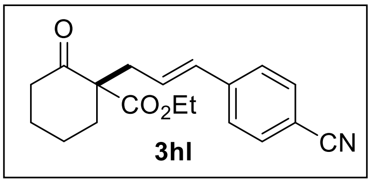

Links NMR CATALOG DETAILS

Following procedure 1-1, the reaction of substrate **2l** (0.2 mmol) for 48 hours afforded **3hl** in a yield of 49.2 mg (79%).

^{1}H NMR (400 MHz, $CDCl_3$) δ 7.24 (s, 4H), 6.32 (d, *J* = 15.8 Hz, 1H), 6.23-6.11 (m, 1H), 4.16 (q, *J* = 7.1 Hz, 2H), 2.73 (dd, *J* = 13.8, 6.7 Hz, 1H), 2.54-2.44 (m, 4H), 2.06-1.98 (m, 1H), 1.81-1.62 (m, 3H), 1.55-1.44 (m, 1H), 1.20 (t, *J* = 7.1 Hz, 3H).

^{13}C NMR (101 MHz, $CDCl_3$) δ 207.8, 171.7, 135.9, 132.9, 132.1, 128.8, 127.5, 126.2, 61.5, 61.5, 41.4, 38.8, 36.4, 27.7, 22.7, 14.4.

HRMS (m/z, ESI-TOF) calcd for $C_{19}H_{22}NO_3^+$ [M+H$^+$]: 312.1594, found 312.1595.

Ethyl (*E*)-2-oxo-1-(3-(perfluorophenyl)allyl)cyclohexane-1-carboxylate **(3hm)**

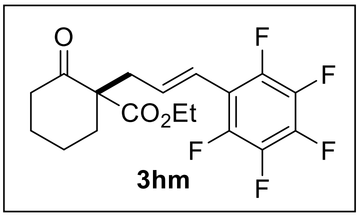

Links NMR CATALOG DETAILS

Following procedure 1-1, the reaction of substrate **2m** (0.2 mmol) for 48 hours afforded **3hm** in a yield of 24.6 mg (33%); the reaction at 50 °C afforded **3hm** in a yield of 66.3 mg (88%).

^{1}H NMR (400 MHz, $CDCl_3$) δ 6.55-6.44 (m, 1H), 6.28 (d, *J* = 16.3 Hz, 1H), 4.27-4.15 (m, 2H), 2.82 (dd, *J* = 14.0, 7.1 Hz, 1H), 2.59-2.44 (m, 4H), 2.13-1.99 (m, 1H), 1.86-1.45 (m, 5H), 1.25 (t, *J* = 7.1 Hz, 3H).

^{13}C NMR (101 MHz, $CDCl_3$) δ 207.3, 171.4, 135.8, 135.7, 117.4, 61.6, 61.2, 41.2, 39.9, 36.4, 27.7, 22.7, 14.3.

^{19}F NMR (376 MHz, $CDCl_3$) δ -143.41 (dd, $J_{F\text{-}F}$ = 21.7, 7.9 Hz), -156.99 (t, $J_{F\text{-}F}$ = 20.8 Hz), -163.21 (tt, $J_{F\text{-}F}$ = 10.0, 4.9 Hz).

HRMS (m/z, ESI-TOF) calcd for $C_{18}H_{18}F_5O_3^+$ [M+H$^+$]: 377.1171, found 377.1173..

Ethyl (*E*)-1-(3-(4-fluorophenyl)allyl)-2-oxocyclohexane-1-carboxylate **(3hn)**

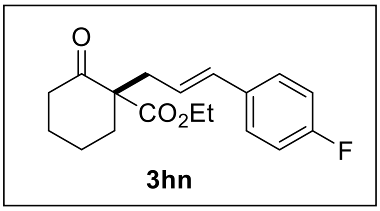

Links NMR CATALOG DETAILS

Following procedure 1-1, the reaction of substrate **2n** (0.2 mmol) for 48 hours afforded **3hn** in a yield of 49.3 mg (81%).

^{1}H NMR (400 MHz, $CDCl_3$) δ 7.29-7.26 (m, 2H), 6.96 (t, *J* = 8.6 Hz, 2H), 6.33 (d, *J* = 15.8 Hz, 1H), 6.18-6.04 (m, 1H), 4.17 (q, *J* = 7.1 Hz, 2H), 2.73 (dd, *J* = 13.9, 6.9 Hz, 1H), 2.54-2.44 (m, 4H), 2.03 (dd, *J* = 6.3, 2.7 Hz, 1H), 1.83-1.48 (m, 4H), 1.21 (t, J = 7.1 Hz, 3H).

^{13}C NMR (101 MHz, $CDCl_3$) δ 207.8, 171.7, 161.0, 133.6, 132.1, 127.8 (d, $J_{C\text{-}F}$ = 7.0 Hz), 125.1, 115.5 (d, $J_{C\text{-}F}$ = 21.3 Hz), 61.5, 61.5, 41.4, 38.7, 36.3, 27.7, 22.7, 14.3.

^{19}F NMR (376 MHz, $CDCl_3$) δ 1.40.

HRMS (m/z, ESI-TOF) calcd for $C_{18}H_{22}FO_3^+$ [M+H$^+$]: 305.1547, found 305.1549.

Ethyl (*E*)-1-(3-(3,5-bis(trifluoromethyl)phenyl)allyl)-2-oxocyclohexane-1-carboxylate **(3ho)**

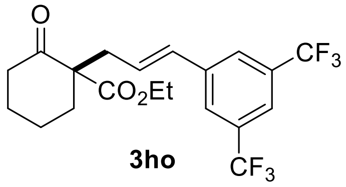

3ho

Links NMR CATALOG DETAILS

Following procedure 1-1, the reaction of substrate **2o** (0.2 mmol) for 48 hours afforded **3ho** in a yield of 73.3 mg (87%).

^{1}H NMR (400 MHz, $CDCl_3$) δ 7.71 (s, 2H), 7.70 (s, 1H), 6.51-6.31 (m, 2H), 4.25-4.11 (m, 2H), 2.78 (dd, *J* = 14.0, 6.0 Hz, 1H), 2.62-2.47 (m, 4H), 2.06-2.03 (m, 1H), 1.84-1.61 (m, 3H), 1.56-1.49 (m, 1H), 1.22 (t, *J* = 7.1 Hz, 3H).

^{13}C NMR (101 MHz, $CDCl_3$) δ 207.6, 171.5, 139.3, 132.0 (q, $J_{C\text{-}F}$ = 33.2 Hz), 130.6, 130.1, 123.5 (q, $J_{C\text{-}F}$ = 272.7 Hz). 120.8, 120.8 , 61.6, 61.4, 41.3, 38.8, 36.6, 27.7, 22.7, 14.3.

HRMS (m/z, ESI-TOF) calcd for $C_{20}H_{21}F_6O_3^+$ [M+H$^+$]: 423.1389, found 423.1393.

Ethyl (*E*)-1-(3-(3-nitrophenyl)allyl)-2-oxocyclohexane-1-carboxylate **(3hp)**

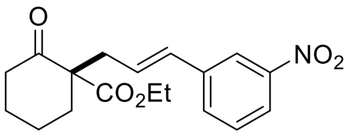

3hp

Links NMR CATALOG DETAILS

Following procedure 1-1, the reaction of substrate **2p** (0.2 mmol) for 48 hours afforded **3hp** in a yield of 57.1 mg (86%).

^{1}H NMR (400 MHz, $CDCl_3$) δ 8.14 (s, 1H), 8.05 (d, *J* = 8.1 Hz, 1H), 7.62 (d, *J* = 7.7 Hz, 1H), 7.45 (t, *J* = 7.9 Hz, 1H), 6.43 (d, *J* = 15.9 Hz, 1H), 6.39-6.30 (m, 1H), 4.25-4.09 (m, 2H), 2.78 (dd, *J* = 14.0, 6.5 Hz, 1H), 2.59-2.45 (m, 4H), 2.09-1.99 (m, 1H), 1.83-1.48 (m, 4H), 1.22 (t, *J* = 7.1 Hz, 3H).

^{13}C NMR (101 MHz, $CDCl_3$) δ 207.6, 171.6, 148.7, 139.1, 132.0, 131.1, 129.5, 129.1, 122.0, 120.9, 61.6, 61.4, 41.3, 38.7, 36.5, 27.7, 22.7, 14.4.

HRMS (m/z, ESI-TOF) calcd for $C_{18}H_{22}NO_5^+$ [M+H$^+$]: 332.1492, found 332.1490.

Ethyl (*E*)-1-(3-(3-fluoro-4-methylphenyl)allyl)-2-oxocyclohexane-1-carboxylate **(3hq)**

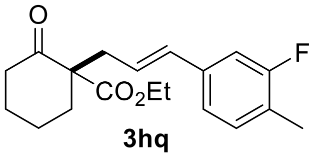

3hq

Links NMR CATALOG DETAILS

Following procedure 1-1, the reaction of substrate **2q** (0.2 mmol) for 48 hours afforded **3hq** in a yield of 53.1 mg (83%).

^{1}H NMR (400 MHz, $CDCl_3$) δ 8.14 (s, 1H), 8.05 (d, J = 8.1 Hz, 1H), 7.62 (d, J = 7.7 Hz, 1H), 7.45 (t, J = 7.9 Hz, 1H), 6.43 (d, J = 15.9 Hz, 1H), 6.39-6.30 (m, 1H), 4.25-4.09 (m, 2H), 2.78 (dd, J = 14.0, 6.5 Hz, 1H), 2.59-2.45 (m, 4H), 2.09-1.99 (m, 1H), 1.83-1.48 (m, 4H), 1.22 (t, J = 7.1 Hz, 3H).

^{13}C NMR (101 MHz, $CDCl_3$) δ 207.8, 171.7, 161.6 (d, $J_{C\text{-}F}$ = 243.9 Hz), 137.2 (d, $J_{C\text{-}F}$ = 7.7 Hz), 132.2 (d, $J_{C\text{-}F}$ = 2.4 Hz), 131.5 (d, $J_{C\text{-}F}$ = 5.6 Hz), 125.7, 123.8 (d, $J_{C\text{-}F}$ = 17.5 Hz) 121.9, 121.8, 112.4 (d, $J_{C\text{-}F}$ = 22.7 Hz), 61.5, 61.5, 41.3, 38.7, 36.3, 27.7, 22.7, 14.5, 14.5, 14.4.

HRMS (m/z, ESI-TOF) calcd for $C_{19}H_{24}FO_3^+$ [M+H$^+$]: 319.1704, found 319.1709.

Ethyl (*E*)-1-(3-(2-fluoropyridin-4-yl)allyl)-2-oxocyclohexane-1-carboxylate **(3hr)**

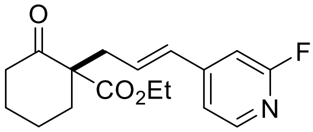

3hr

Links NMR CATALOG DETAILS

Following procedure 1-1, the reaction of substrate **2r** (0.2 mmol) for 48 hours afforded **3hr** in a yield of 46.6 mg (77%).

^{1}H NMR (400 MHz, $CDCl_3$) δ 8.14 (s, 1H), 8.05 (d, J = 8.1 Hz, 1H), 7.62 (d, J = 7.7 Hz, 1H), 7.45 (t, J = 7.9 Hz, 1H), 6.43 (d, J = 15.9 Hz, 1H), 6.39-6.30 (m, 1H), 4.25-4.09 (m, 2H), 2.78 (dd, J = 14.0, 6.5 Hz, 1H), 2.59-2.45 (m, 4H), 2.09-1.99 (m, 1H), 1.83-1.48 (m, 4H), 1.22 (t, J = 7.1 Hz, 3H).

^{13}C NMR (101 MHz, $CDCl_3$) δ 207.5, 171.4, 164.6 (d, $J_{C\text{-}F}$ = 237.6 Hz), 150.3 (d, $J_{C\text{-}F}$ = 8.2 Hz), 147.8 (d, $J_{C\text{-}F}$ = 15.5 Hz), 132.5, 130.1 (d, $J_{C\text{-}F}$ = 3.6 Hz), 118.6 (d, $J_{C\text{-}F}$ = 3.9 Hz), 106.2 (d, $J_{C\text{-}F}$ = 37.9 Hz), 61.6, 61.3, 41.3, 38.7, 36.6, 27.6, 22.7, 14.3.

HRMS (m/z, ESI-TOF) calcd for $C_{17}H_{21}FNO_3^+$ [M+H$^+$]: 306.1500, found 306.1502.

Ethyl (*E*)-1-(3-(naphthalen-1-yl)allyl)-2-oxocyclohexane-1-carboxylate **(3hs)**

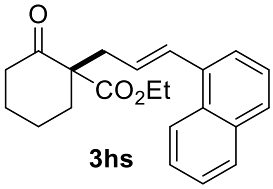

3hs

Links NMR CATALOG DETAILS

Following procedure 1-1, the reaction of substrate **2s** (0.2 mmol) for 48 hours afforded **3hs** in a yield of 52.9 mg (79%).

^{1}H NMR (400 MHz, $CDCl_3$) δ 8.06 (d, *J* = 7.9 Hz, 1H), 7.87-7.80 (m, 1H), 7.74 (d, *J* = 8.2 Hz, 1H), 7.55-7.38 (m, 4H), 7.12 (d, *J* = 15.5 Hz, 1H), 6.20 (dt, *J* = 15.3, 7.5 Hz, 1H), 4.25-4.07 (m, 2H), 2.88 (dd, *J* = 13.8, 7.0 Hz, 1H), 2.65-2.58 (m, 2H), 2.53-2.49 (m, 2H), 2.04-2.02 (m, 1H), 1.84-1.55 (m, 4H), 1.21 (t, *J* = 7.1 Hz, 3H).
^{13}C NMR (101 MHz, $CDCl_3$) δ 207.7, 171.7, 135.2, 133.7, 131.2, 130.7, 128.6, 128.6, 127.7, 126.0, 125.8, 125.8, 124.0, 123.9, 61.6, 61.5, 41.4, 39.1, 36.3, 27.7, 22.8, 14.3.
HRMS (m/z, ESI-TOF) calcd for $C_{22}H_{25}O_3^+$ [M+H$^+$]: 337.1798, found 337.1800.

Ethyl (*E*)-1-(3-(naphthalen-2-yl)allyl)-2-oxocyclohexane-1-carboxylate **(3ht)**

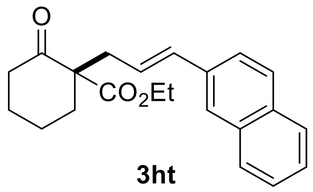

3ht

Links NMR CATALOG DETAILS

Following procedure 1-1, the reaction of substrate **2t** (0.2 mmol) for 48 hours afforded **3ht** in a yield of 59.9 mg (89%).
^{1}H NMR (400 MHz, $CDCl_3$) δ 7.81-7.72 (m, 3H), 7.66 (s, 1H), 7.54 (d, *J* = 8.5 Hz, 1H), 7.47-7.36 (m, 2H), 6.53 (d, *J* = 15.8 Hz, 1H), 6.39-6.25 (m, 1H), 4.18 (q, *J* = 7.1 Hz, 2H), 2.80 (dd, *J* = 13.9, 6.9 Hz, 1H), 2.62-2.46 (m, 4H), 2.04-2.02 (m, 1H), 1.81-1.50 (m, 4H), 1.21 (t, *J* = 7.1 Hz, 3H).
^{13}C NMR (101 MHz, $CDCl_3$) δ 207.9, 171.8, 134.9, 133.7, 133.4, 133.0, 128.2, 128.0, 127.8, 126.3, 125.9, 125.8, 125.8, 123.8, 61.6, 61.5, 41.9, 39.0, 36.3, 27.7, 22.8, 14.4.
HRMS (m/z, ESI-TOF) calcd for $C_{22}H_{25}O_3^+$ [M+H$^+$]: 337.1798, found 337.1800.

Tert-butyl (*E*)-2-(3-(1-(ethoxycarbonyl)-2-oxocyclohexyl)prop-1-en-1-yl)-1H-indole-1-carboxylate **(3hu)**

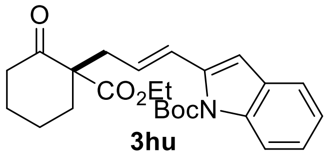

3hu

Links NMR CATALOG DETAILS

Following procedure 1-1, the reaction of substrate **2u** (0.2 mmol) for 48 hours afforded **3hu** in a yield of 71.3 mg (84%).
^{1}H NMR (400 MHz, $CDCl_3$) δ 8.05 (d, *J* = 8.2 Hz, 1H), 7.46 (d, *J* = 7.4 Hz, 1H), 7.21 (dt, *J* = 20.6, 7.3 Hz, 2H), 6.95 (d, *J* = 15.6 Hz, 1H), 6.63 (s, 1H), 6.11 (dt, *J* = 15.5, 7.6 Hz, 1H), 4.20 (q, *J* = 7.1 Hz, 2H), 2.79 (dd, *J* = 13.9, 7.1 Hz, 1H), 2.55-2.47 (m, 4H), 2.10-1.96 (m, 1H), 1.79-1.49 (m, 13H), 1.25 (t, *J* = 7.1 Hz, 3H).
^{13}C NMR (101 MHz, $CDCl_3$) δ 207.7, 171.6, 150.6, 139.2, 136.6, 129.5, 127.7, 125.7, 124.0, 123.0, 120.3, 115.8, 106.9, 84.1, 61.5, 61.5, 41.3, 38.7, 36.3, 28.4, 27.7, 22.7, 14.3.
HRMS (m/z, ESI-TOF) calcd for $C_{25}H_{32}NO_5^+$ [M+H$^+$]: 426.2275, found 426.2278.

3.6 **Characterization of amination products**

1-cinnamylazepane **(7aa)**

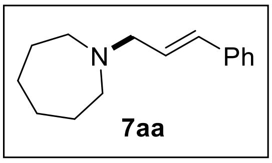

Links NMR CATALOG DETAILS

Following procedure 2-1, the reaction of substrate **6a** (0.2 mmol) for 84 hours afforded **7aa**: 35.9 mg, 83%.

^{1}H NMR (400 MHz, $CDCl_3$) δ 7.38 (d, *J* = 7.3 Hz, 2H), 7.30 (t, *J* = 7.5 Hz, 2H), 7.21 (t, *J* = 7.2 Hz, 1H), 6.50 (d, *J* = 15.9 Hz, 1H), 6.31 (dt, *J* = 15.8, 6.6 Hz, 1H), 3.28 (dd, *J* = 6.6, 0.9 Hz, 2H), 2.68-2.65 (m, 4H), 1.70-1.61 (m, 8H).

^{13}C NMR (101 MHz, $CDCl_3$) δ 137.3, 132.3, 128.7, 128.2, 127.4 126.4, 61.2, 55.9, 28.1, 27.0.

HRMS (m/z, ESI-TOF) calcd for $C_{15}H_{22}N^+$: 216.1747, found 216.1749.

1-cinnamylpiperidine **(7ba)**

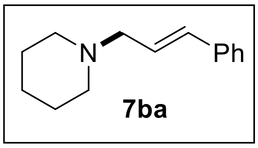

Links NMR CATALOG DETAILS

Following procedure 2-1, the reaction of substrate **6b** (0.2 mmol) for 84 hours afforded **7ba**: 28.2 mg, 70% yield.

^{1}H NMR (400 MHz, $CDCl_3$) δ 7.37 (d, *J* = 7.3 Hz, 2H), 7.30 (t, *J* = 7.5 Hz, 2H), 7.22 (t, *J* = 7.2 Hz, 1H), 6.50 (d, *J* = 15.9 Hz, 1H), 6.31 (dt, *J* = 15.8, 6.8 Hz, 1H), 3.13 (dd, *J* = 6.8, 0.9 Hz, 2H), 2.45 (s, 2H), 1.62 (dt, *J* = 11.1, 5.6 Hz, 4H), 1.48-1.42 (m, 2H).

^{13}C NMR (101 MHz, $CDCl_3$) δ 137.2, 132.9, 128.7, 127.5, 127.2, 126.4, 62.0, 54.7, 26.1, 24.5.

HRMS (m/z, ESI-TOF) calcd for $C_{14}H_{20}N^+$: 202.1590, found 202.1592.

1-cinnamylazocane **(7ca)**

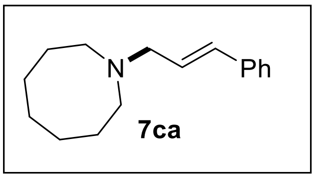

Links NMR CATALOG DETAILS

Following procedure 2-1, the reaction of substrate **6c** (0.2 mmol) for 84 hours afforded **7ca**: 27.3 mg, 64% yield.

^{1}H NMR (400 MHz, $CDCl_3$) δ 7.37 (d, *J* = 7.4 Hz, 2H), 7.30 (t, *J* = 7.6 Hz, 2H), 7.21 (t, *J* = 7.2 Hz, 1H), 6.49 (d, *J* = 15.9 Hz, 1H), 6.28 (dt, *J* = 15.8, 6.5 Hz, 1H), 3.25 (d, *J* = 6.4 Hz, 2H), 2.61 (s, 4H), 1.60-1.59 (m, 10H).

^{13}C NMR (101 MHz, $CDCl_3$) δ 137.6, 131.5, 129.3, 128.7, 127.3, 126.4, 61.4, 53.8, 27.9, 27.6, 26.4.

HRMS (m/z, ESI-TOF) calcd for $C_{16}H_{24}N^+$: 230.1903, found 230.1904.

(*E*)-*N*,*N*-diisopropyl-3-phenylprop-2-en-1-amine **(7da)**

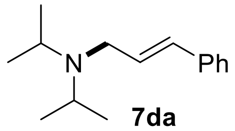

7da

Links NMR CATALOG DETAILS

Following procedure 2-1, the reaction of substrate **6d** (0.2 mmol) for 84 hours afforded **7da**: 23.8 mg, 55% yield.

^{1}H NMR (400 MHz, $CDCl_3$) δ 7.37 (d, *J* = 7.3 Hz, 2H), 7.29 (t, *J* = 7.6 Hz, 2H), 7.19 (t, *J* = 7.2 Hz, 1H), 6.50 (d, *J* = 15.8 Hz, 1H), 6.24 (dt, *J* = 15.8, 6.2 Hz, 1H), 3.28 (dd, *J* = 6.1, 1.0 Hz, 2H), 3.10 (dp, *J* = 13.1, 6.5 Hz, 2H), 1.05 (s, 6H), 1.03 (s, 6H).

^{13}C NMR (101 MHz, $CDCl_3$) δ 137.8, 132.0, 130.1, 128.6, 127.1, 126.2, 48.5, 47.8, 20.9.

HRMS (m/z, ESI-TOF) calcd for $C_{15}H_{24}N^{+}$: 218.1903, found 218.1905.

(*E*)-*N*-benzyl-*N*-isopropyl-3-phenylprop-2-en-1-amine (**7ea**)

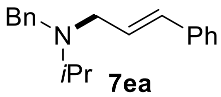

7ea

Links NMR CATALOG DETAILS

Following procedure 2-1, the reaction of substrate **6e** (0.2 mmol) for 72 hours afforded **7ea**: 50.5 mg, 95% yield.

^{1}H NMR (400 MHz, $CDCl_3$) δ 7.38-7.26 (m, 8H), 7.22-7.17 (m, 2H), 6.50 (d, *J* = 15.9 Hz, 1H), 6.20 (dt, *J* = 15.7, 6.3 Hz, 1H), 3.59 (s, 2H), 3.22 (d, *J* = 6.3 Hz, 2H), 3.05 (dt, *J* = 13.1, 6.5 Hz, 1H), 1.07 (s, 3H), 1.05 (s, 3H).

^{13}C NMR (101 MHz, $CDCl_3$) δ 141.2, 137.5, 131.3, 129.8, 128.7, 128.6, 128.3, 127.2, 126.7, 126.3, 53.4, 52.3, 49.5, 18.1.

HRMS (m/z, ESI-TOF) calcd for $C_{19}H_{24}N^{+}$: 266.1903, found 266.1905.

(*E*)-*N*-benzyl-*N*-(*tert*-butyl)-3-phenylprop-2-en-1-amine (**7fa**)

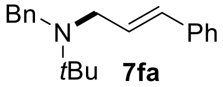

7fa

Links NMR CATALOG DETAILS

Following procedure 2-1, the reaction of substrate **6f** (0.2 mmol) for 72 hours afforded **7fa**: 53.5 mg, 96% yield.

^{1}H NMR (400 MHz, $CDCl_3$) δ 7.38 (d, *J* = 7.3 Hz, 2H), 7.28-7.16(m, 8H), 6.30 (d, *J* = 15.9 Hz, 1H), 6.20-6.11 (m, 1H), 3.73 (s, 2H), 3.37 (d, *J* = 6.2 Hz, 2H), 1.18 (s, 9H).

^{13}C NMR (101 MHz, $CDCl_3$) δ 142.9, 137.7, 130.7, 130.3, 128.5, 128.4, 128.1, 127.0, 126.3, 126.2, 55.2, 52.8, 51.9, 28.0.

HRMS (m/z, ESI-TOF) calcd for $C_{20}H_{26}N^{+}$: 280.2060, found 280.2061.

(*E*)-*N*-methyl-*N*-(naphthalen-1-ylmethyl)-3-phenylprop-2-en-1-amine (**7ga**)

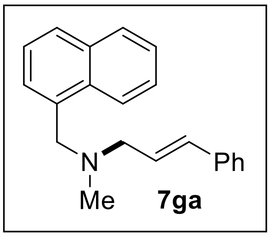

Links NMR CATALOG DETAILS

Following procedure 2-1, the reaction of substrate **6g** (0.2 mmol) for 72 hours afforded **7ga**: 43.5 mg, 92% yield.

Following procedure 2-3, the reaction of substrate **6g** (2.5 mmol) for 84 hours afforded **7ga**: 587.6 mg, 82% yield.

^{1}H NMR (400 MHz, $CDCl_3$) δ 8.22 (d, J = 8.2 Hz, 1H), 7.78 (d, J = 7.8 Hz, 1H), 7.71 (d, J = 8.0 Hz, 1H), 7.46-7.30 (m, 6H), 7.24 (t, J = 7.5 Hz, 2H), 7.15 (t, J = 7.3 Hz, 1H), 6.51 (d, J = 15.9 Hz, 1H), 6.31 (dt, J = 15.8, 6.7 Hz, 1H), 3.90 (s, 2H), 3.23 (d, J = 6.5 Hz, 2H), 2.22 (s, 3H).

^{13}C NMR (101 MHz, $CDCl_3$) δ 137.3, 134.9, 134.0, 132.9, 132.7, 128.7, 128.6, 128.1, 127.6, 127.5, 126.5, 126.0, 125.7, 125.3, 124.7, 60.5, 60.1, 42.6.

HRMS (m/z, ESI-TOF) calcd for $C_{17}H_{20}N^+$: 238.1590, found 238.1586.

4-cinnamylmorpholine **(7ha)**

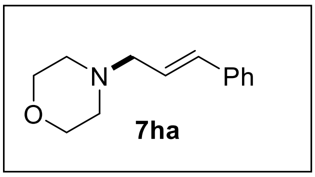

Links NMR CATALOG DETAILS E-Fact

Following procedure 2-1, the reaction of substrate **6h** (0.2 mmol) for 72 hours afforded **7ha**: 37.3 mg, 92% yield (with 0.24 mmol **2a**); the reaction of substrate **6f** (2.5 mmol) for 84 hours afforded **7ha**: 10.8 mg, 27% yield (with 0.24 mmol **2v**).

^{1}H NMR (400 MHz, $CDCl_3$) δ 7.40-7.36 (m, 2H), 7.31 (t, J = 7.5 Hz, 2H), 7.24 (dd, J = 8.2, 6.2 Hz, 1H), 6.54 (d, J = 15.9 Hz, 1H), 6.26 (dt, J = 15.8, 6.8 Hz, 1H), 3.77-3.73 (m, 4H), 3.17 (dd, J = 6.8, 0.7 Hz, 2H), 2.52 (s, 4H).

^{13}C NMR (101 MHz, $CDCl_3$) δ 136.9, 133.7, 128.7, 127.8, 126.5, 126.0, 67.1, 61.6, 53.8.

HRMS (m/z, ESI-TOF) calcd for $C_{13}H_{18}NO^+$: 204.1383, found 204.1379.

4-cinnamylthiomorpholine **(7ia)**

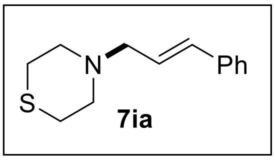

Links NMR CATALOG DETAILS

Following procedure 2-1, the reaction of substrate **6i** (0.2 mmol) for 84 hours afforded **7ia**: 19.7 mg, 45% yield.

^{1}H NMR (400 MHz, $CDCl_3$) δ 7.32-7.28 (m, 2H), 7.27-7.22 (m, 2H), 7.19-7.13 (m, 1H), 6.45 (d, J = 15.9 Hz, 1H), 6.17 (dt, J = 15.8, 6.8 Hz, 1H), 3.12 (dd, J = 6.8, 0.9 Hz, 2H), 2.73-2.70 (m, 4H), 2.66-2.61 (m, 4H).

^{13}C NMR (101 MHz, $CDCl_3$) δ 136.9, 133.6, 128.7, 127.8, 126.5, 62.0, 55.1, 28.1.

HRMS (m/z, ESI-TOF) calcd for $C_{13}H_{18}NS^+$: 220.1154, found 220.1153.

13-cinnamyl-1,4,7,10-tetraoxa-13-azacyclopentadecane **(7ja)**

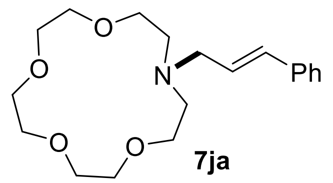

7ja

Links NMR CATALOG DETAILS

Following procedure 2-1, the reaction of substrate **6j** (0.2 mmol) for 84 hours afforded **7ja**: 60.0 mg, 90% yield.

^{1}H NMR (400 MHz, $CDCl_3$) δ 7.36 (d, *J* = 7.3 Hz, 2H), 7.30 (t, *J* = 7.5 Hz, 2H), 7.21 (t, *J* = 7.2 Hz, 1H), 6.50 (d, *J* = 15.9 Hz, 1H), 6.29 (dt, *J* = 15.8, 6.6 Hz, 1H), 3.72-3.66 (m, 16H), 3.34 (d, *J* = 6.5 Hz, 2H), 2.83 (t, *J* = 6.0 Hz, 4H).

^{13}C NMR (101 MHz, $CDCl_3$) δ 137.2, 132.7, 128.6, 127.5, 126.4, 71.1, 70.5, 70.3, 70.0, 59.4, 54.4.

HRMS (m/z, ESI-TOF) calcd for calcd for $C_{19}H_{30}NO_4^+$: 336.2169, found 336.2171.

1,4-dicinnamylpiperazine **(7ka)**

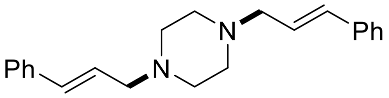

7ka

Links NMR CATALOG DETAILS

Following procedure 2-1, the reaction of substrate **6k** (0.1 mmol) for 84 hours afforded **7ka**: 29.5 mg, 93% yield.

^{1}H NMR (400 MHz, $CDCl_3$) δ 7.37 (d, *J* = 7.3 Hz, 4H), 7.30 (t, *J* = 7.5 Hz, 4H), 7.22 (t, *J* = 7.2 Hz, 2H), 6.53 (d, *J* = 15.9 Hz, 2H), 6.28 (dt, *J* = 15.8, 6.8 Hz, 2H), 3.18 (d, *J* = 6.3 Hz, 4H), 2.58 (brs, 8H).

^{13}C NMR (101 MHz, $CDCl_3$) δ 137.0, 133.3, 128.7, 127.6, 126.5, 126.4, 61.1, 53.2.

HRMS (m/z, ESI-TOF) calcd for $C_{22}H_{27}N_2^+$: 319.2169, found 319.2166.

Tert-butyl 4-cinnamylpiperazine-1-carboxylate **(7la)**

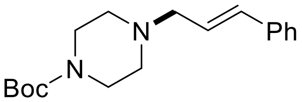

7la

Links NMR CATALOG DETAILS

Following procedure 2-1, the reaction of substrate **6l** (0.2 mmol) for 84 hours afforded **7la**: 58.1 mg, 96% yield.

^{1}H NMR (400 MHz, $CDCl_3$) δ 7.43-7.26 (m, 10H), 7.24-7.13 (m, 5H), 6.51 (d, *J* = 15.8 Hz, 1H), 6.31-6.22 (m, 1H), 4.24 (s, 1H), 3.18 (d, J = 6.8 Hz, 2H), 2.62-2.42 (m, 8H).

^{13}C NMR (101 MHz, $CDCl_3$) δ 142.8, 137.0, 133.4, 128.7, 128.6, 128.0, 127.6, 127.0, 126.4, 126.3, 76.3, 61.1, 53.5, 51.9.

HRMS (m/z, ESI-TOF) calcd for $C_{18}H_{27}N_2O_2^+$: 303.2067, found 303.2070.

2-(4-cinnamylpiperazin-1-yl)pyrimidine **(7ma)**

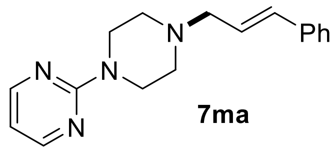

7ma

Links NMR CATALOG DETAILS

Following procedure 2-1, the reaction of substrate **6m** (0.2 mmol) for 84 hours afforded **7ma**: 50.7 mg, 91% yield.

^{1}H NMR (400 MHz, $CDCl_3$) δ 8.32 (d, *J* = 4.7 Hz, 2H), 7.41 (d, *J* = 7.4 Hz, 2H), 7.34 (t, *J* = 7.5 Hz, 2H), 7.26 (t, *J* = 7.2 Hz, 1H), 6.57 (d, *J* = 15.9 Hz, 1H), 6.50 (t, *J* = 4.7 Hz, 1H), 6.33 (dt, *J* = 15.8, 6.8 Hz, 1H), 3.91-3.83 (m, 4H), 3.23 (d, *J* = 6.7 Hz, 2H), 2.61-2.57 (m, 3H).

^{13}C NMR (101 MHz, $CDCl_3$) δ 161.8, 157.8, 136.9, 133.4, 128.7, 127.7, 126.5, 126.4, 110.0, 61.3, 53.2, 43.8.

HRMS (m/z, ESI-TOF) calcd for $C_{17}H_{21}N_4^+$: 281.1761, found 281.1763.

1-benzhydryl-4-cinnamylpiperazine **(7na)**

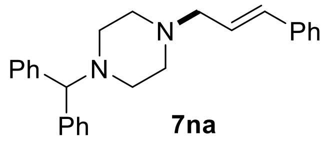

7na

Links NMR CATALOG DETAILS

Following procedure 2-1, the reaction of substrate **6n** (0.2 mmol) for 84 hours afforded **7na**: 70.7 mg, 96% yield.
Following procedure 2-3, the reaction of substrate **6n** (2.5 mmol) for 84 hours afforded **7na**: 696.8 mg, 65% yield.

^{1}H NMR (400 MHz, $CDCl_3$) δ 7.43-7.26 (m, 10H), 7.24-7.13 (m, 5H), 6.51 (d, *J* = 15.8 Hz, 1H), 6.31-6.22 (m, 1H), 4.24 (s, 1H), 3.18 (d, J = 6.8 Hz, 2H), 2.62-2.42 (m, 8H).

^{13}C NMR (101 MHz, $CDCl_3$) δ 142.8, 137.0, 133.4, 128.7, 128.6, 128.0, 127.6, 127.0, 126.4, 126.3, 76.3, 61.1, 53.5, 51.9.

HRMS (m/z, ESI-TOF) calcd for $C_{26}H_{29}N_2^+$: 369.2325, found 369.2323.

1-(bis(4-fluorophenyl)methyl)-4-cinnamylpiperazine **(7oa)**

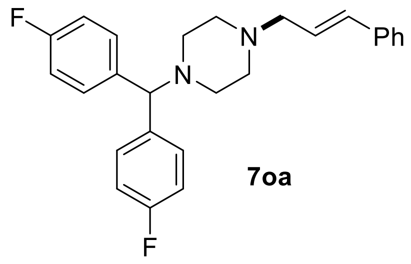

7oa

Links NMR CATALOG DETAILS

Following procedure 2-1, the reaction of substrate **6o** (0.2 mmol) for 84 hours afforded **7oa**: 75.8 mg, 94% yield.
Following procedure 2-3, the reaction of substrate **6o** (2.5 mmol) for 84 hours afforded **7oa**: 617.2 mg, 61% yield.

^{1}H NMR (400 MHz, $CDCl_3$) δ 7.38-7.19 (m, 9H), 7.00-6.93 (m, 4H), 6.51 (d, *J* = 15.8 Hz, 1H), 6.26 (dt, *J* = 15.7, 6.8 Hz, 1H), 4.23 (s, 1H), 3.18 (d, *J* = 6.7 Hz, 2H), 2.61-2.30 (m, 8H).

^{13}C NMR (101 MHz, $CDCl_3$) δ 161.9 (d, $J_{C\text{-}F}$ = 245.5 Hz), 138.3 (d, $J_{C\text{-}F}$ = 3.0 Hz), 136.9, 133.5, 129.4 (d, $J_{C\text{-}F}$ = 7.8 Hz), 128.7, 127.7, 126.4, 126.1, 115.5 (d, $J_{C\text{-}F}$ = 21.2 Hz), 74.5, 61.0, 53.4, 51.7.
^{19}F NMR (376 MHz, $CDCl_3$) δ -115.64 (ddd, $J_{F\text{-}F}$ = 13.9, 8.6, 5.6 Hz).
HRMS (m/z, ESI-TOF) calcd for $C_{26}H_{27}F_2N_2^+$: 405.2137, found 405.2141.

(*E*)-4-(3-(2,4,6-trimethoxyphenyl)allyl)morpholine **(7hd)**

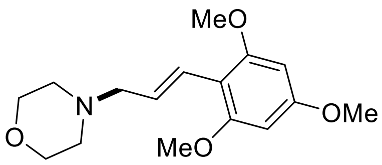

7hd

Links NMR CATALOG DETAILS

Following procedure 2-1, the reaction of substrate **6h** (0.2 mmol) for 72 hours afforded **7hd**: 51.6 mg, 88% yield.
^{1}H NMR (400 MHz, $CDCl_3$) δ 6.61 (s, 2H), 6.47 (d, *J* = 15.8 Hz, 1H), 6.29-6.11 (m, 1H), 3.87 (s, 4H), 3.84 (s, 3H), 3.78-3.72 (m, 4H), 3.16 (d, *J* = 6.7 Hz, 2H), 2.53 (s, 4H).
^{13}C NMR (101 MHz, $CDCl_3$) δ 153.4, 137.9, 133.5, 132.6, 125.6, 103.5, 67.1, 61.5, 61.1, 56.2, 53.8.
HRMS (m/z, ESI-TOF) calcd for $C_{16}H_{24}NO_4^+$: 294.1700, found 294.1699.

(*E*)-4-(3-(3,4-dimethoxyphenyl)allyl)morpholine **(7he)**

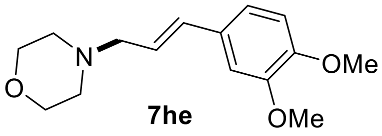

7he

Links NMR CATALOG DETAILS

Following procedure 2-1, the reaction of substrate **6h** (0.2 mmol) for 72 hours afforded **7he**: 43.8 mg, 83% yield.
^{1}H NMR (400 MHz, $CDCl_3$) δ 6.96 (d, *J* = 1.5 Hz, 1H), 6.90 (dd, *J* = 8.2, 1.6 Hz, 1H), 6.81 (d, *J* = 8.2 Hz, 1H), 6.47 (d, *J* = 15.8 Hz, 1H), 6.13 (dt, *J* = 15.7, 6.9 Hz, 1H), 3.89 (s, 3H), 3.88 (s, 3H), 3.77-3.73 (m, 4H), 3.15 (d, *J* = 6.7 Hz, 2H), 2.52 (s, 4H).
^{13}C NMR (101 MHz, $CDCl_3$) δ 149.2, 148.9, 133.3, 130.0, 124.0, 119.7, 111.2, 108.7, 67.1, 61.6, 56.1, 55.9, 53.8.
HRMS (m/z, ESI-TOF) calcd for $C_{15}H_{22}NO_3^+$: 264.1594, found 264.1591.

(*E*)-4-(3-(3-(benzyloxy)phenyl)allyl)morpholine **(7hg)**

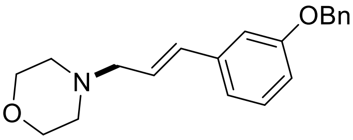

7hg

Links NMR CATALOG DETAILS

Following procedure 2-1, the reaction of substrate **6h** (0.05 mmol) for 72 hours afforded **7hg**: 13.6 mg, 88% yield.
^{1}H NMR (400 MHz, $CDCl_3$) δ 7.46-7.37 (m, 4H), 7.36-7.30 (m, 1H), 7.25-7.21 (m, 1H), 7.05-6.97 (m, 2H), 6.87 (dd, *J* = 8.1, 1.7 Hz, 1H), 6.51 (d, *J* = 15.8 Hz, 1H), 6.32-6.20 (m, 1H), 5.07 (s, 2H), 3.76 (s, 2H), 3.18 (d, *J* = 6.1 Hz, 2H), 2.54 (s, 4H).
^{13}C NMR (101 MHz, $CDCl_3$) δ 159.2, 138.3, 137.1, 133.7, 129.8, 128.7, 128.1, 127.6, 126.2, 119.5, 114.4, 112.8, 70.1, 67.0, 61.5, 53.7.
HRMS (m/z, ESI-TOF) calcd for $C_{20}H_{24}NO_2^+$: 310.1802, found 310.1805.

(*E*) -2-(3-morpholinoprop-1-en-1-yl)phenol **(7hh)**

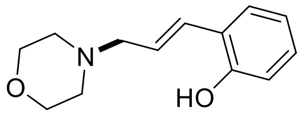

7hh

Links NMR CATALOG DETAILS

Following procedure 2-1, the reaction of substrate **6h** (0.2 mmol) for 72 hours afforded **7hh**: 34.2 mg, 78% yield.
^{1}H NMR (400 MHz, $CDCl_3$) δ 7.36 (dd, *J* = 7.7, 1.3 Hz, 1H), 7.15-7.08 (m, 1H), 6.89 (t, *J* = 7.3 Hz, 1H), 6.81-6.75 (m, 2H), 6.33 (dt, *J* = 15.9, 6.9 Hz, 1H), 3.79-3.74 (m, 4H), 3.22 (dd, *J* = 6.8, 0.9 Hz, 2H), 2.57 (s, 4H).
^{13}C NMR (101 MHz, $CDCl_3$) δ 153.3, 128.9, 128.8, 127.9, 127.0, 124.1, 120.9, 116.1, 77.5, 76.8, 66.9, 62.0, 53.7.
HRMS (m/z, ESI-TOF) calcd for $C_{13}H_{17}NNaO_2^+$: 242.1151, found 242.1149.

(*E*)-4-(3-(4-vinylphenyl)allyl)morpholine **(7hi)**

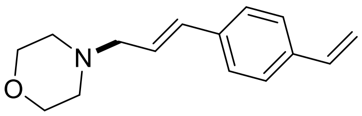

7hi

Links NMR CATALOG DETAILS

Following procedure 2-1, the reaction of substrate **6h** (0.2 mmol) for 72 hours afforded **7hi**: 40.1 mg, 87% yield.
^{1}H NMR (400 MHz, $CDCl_3$) δ 7.38-7.31 (m, 4H), 6.69 (dd, *J* = 17.6, 10.9 Hz, 1H), 6.53 (d, *J* = 15.8 Hz, 1H), 6.27 (dt, *J* = 15.8, 6.9 Hz, 1H), 5.74 (dd, *J* = 17.6, 0.6 Hz, 1H), 5.24 (d, *J* = 10.9 Hz, 1H), 3.77 (t, *J* = 4.5 Hz, 4H), 3.20 (d, *J* = 6.7 Hz, 2H), 2.56 (brs, 4H).
^{13}C NMR (101 MHz, $CDCl_3$) δ 137.1, 136.5, 136.3, 133.7, 126.7, 126.6, 113.9, 66.9, 61.5, 53.7.
HRMS (m/z, ESI-TOF) calcd for $C_{15}H_{20}NO^+$: 230.1539, found 230.1541.

(*E*)-4-(3-(perfluorophenyl)allyl)morpholine **(7hm)**

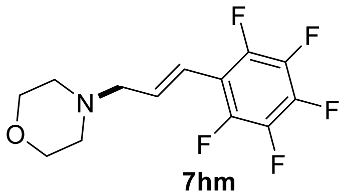

7hm

Links NMR CATALOG DETAILS

Following procedure 2-1, the reaction of substrate **6h** (0.2 mmol) for 96 hours afforded **7hm**: 42.5 mg, 73% yield.

^{1}H NMR (400 MHz, $CDCl_3$) δ 6.52 (dt, J = 16.3, 6.3 Hz, 1H), 6.40 (d, J = 16.4 Hz, 1H), 3.69-3.66 (m, 3H), 3.14 (d, J = 6.3 Hz, 2H), 2.46-2.42 (m, 4H).
^{13}C NMR (101 MHz, $CDCl_3$) δ 136.3, 117.2, 67.0, 62.1, 53.8.
^{19}F NMR (376 MHz, $CDCl_3$) δ -143.06 (dd, J_{F-F} = 21.6, 7.8 Hz), -156.37 (t, J_{F-F} = 20.8 Hz), -162.96 (tt, J_{F-F} = 9.9, 4.9 Hz).
HRMS (m/z, ESI-TOF) calcd for $C_{13}H_{13}F_5NO^+$: 294.0912, found 294.0915.

(E)-4-(3-(naphthalen-2-yl)allyl)morpholine **(7ht)**

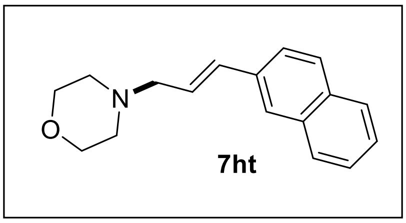

Links NMR CATALOG DETAILS

Following procedure 2-1, the reaction of substrate **6h** (0.2 mmol) for 72 hours afforded **7ht**: 40.1 mg, 87% yield.
^{1}H NMR (400 MHz, $CDCl_3$) δ 7.38-7.31 (m, 4H), 6.69 (dd, J = 17.6, 10.9 Hz, 1H), 6.53 (d, J = 15.8 Hz, 1H), 6.27 (dt, J = 15.8, 6.9 Hz, 1H), 5.74 (dd, J = 17.6, 0.6 Hz, 1H), 5.24 (d, J = 10.9 Hz, 1H), 3.77 (t, J = 4.5 Hz, 4H), 3.20 (d, J = 6.7 Hz, 2H), 2.56 (brs, 4H).
^{13}C NMR (101 MHz, $CDCl_3$) δ 137.1, 136.5, 136.3, 133.7, 126.7, 126.6, 113.9, 66.9, 61.5, 53.7.
HRMS (m/z, ESI-TOF) calcd for $C_{15}H_{20}NO^+$: 230.1539, found 230.1541.

(E)-4-(4-phenylbut-2-en-1-yl)morpholine **(7hw)**

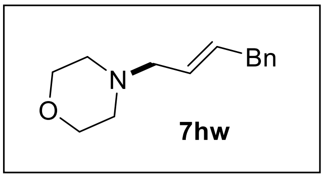

Links NMR CATALOG DETAILS

Following procedure 2-1, the reaction of substrate **6h** (0.2 mmol) for 72 hours afforded **7hw**: 16.0 mg, 37% yield.
^{1}H NMR (400 MHz, $CDCl_3$) δ 7.32-7.25 (m, 2H), 7.21-7.16 (m, 3H), 5.83-5.72 (m, 1H), 5.63-5.50 (m, 1H), 3.78-3.68 (m, 4H), 3.38 (d, J = 6.6 Hz, 2H), 2.98 (d, J = 6.7 Hz, 2H), 2.44 (s, 4H).
^{13}C NMR (101 MHz, $CDCl_3$) δ 140.3, 133.6, 128.6, 128.6, 127.4, 126.2, 67.1, 61.3, 53.7, 39.0.
HRMS (m/z, ESI-TOF) calcd for $C_{14}H_{20}NO^+$: 218.1539, found 218.1538.

methyl (E)-N-(3-(3,4-dimethoxyphenyl)allyl)-N-(4-fluorobenzyl)glycinate **(7pe)**

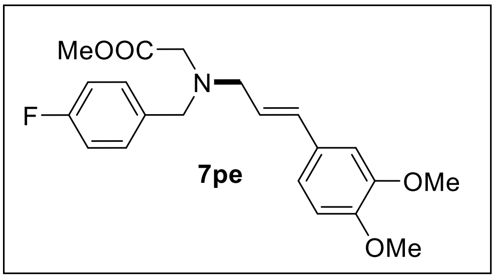

Links NMR CATALOG DETAILS

Following procedure 2-1, the reaction of substrate **6e** (0.2 mmol) for 72 hours afforded **7pe**: 69.4 mg, 93% yield. Following procedure 2-3, the reaction of substrate **6e** (2.5 mmol) for 84 hours afforded **7pe**: 818.6 mg, 88% yield.

^{1}H NMR (400 MHz, $CDCl_3$) δ 7.33 (dd, *J* = 8.1, 5.6 Hz, 2H), 7.00 (t, *J* = 8.6 Hz, 2H), 6.93 (s, 1H), 6.88 (d, *J* = 8.3 Hz, 1H), 6.79 (d, *J* = 8.2 Hz, 1H), 6.46 (d, *J* = 15.8 Hz, 1H), 6.14-6.06 (m, 1H), 3.88 (s, 3H), 3.86 (s, 3H), 3.76 (s, 2H), 3.66 (s, 3H), 3.39-3.32 (m, 4H).

^{13}C NMR (101 MHz, $CDCl_3$) δ 171.9, 162.1 (d, $J_{C\text{-}F}$ = 245.0 Hz), 149.1, 148.8, 134.3 (d, $J_{C\text{-}F}$ = 2.3 Hz), 132.9, 130.6 (d, $J_{C\text{-}F}$ = 7.9 Hz), 130.0, 125.0, 119.6, 115.2 (d, $J_{C\text{-}F}$ = 21.2 Hz), 111.1, 108.7, 57.5, 56.5, 56.0, 55.9, 53.8, 51.5.

HRMS (m/z, ESI-TOF) calcd for $C_{21}H_{25}FNO_4^+$: 374.1762, found 374.1765.

1-cinnamyl-1,2,3,4-tetrahydroquinoline **(8aa)**

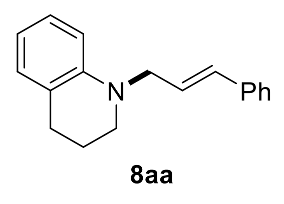

Links NMR CATALOG DETAILS

Following procedure 2-4, the reaction of substrate **6a’** (0.2 mmol) for 72 hours afforded **6aa**: 44.8 mg, 91% yield.

^{1}H NMR (400 MHz, $CDCl_3$) δ 7.37-7.33 (m, 2H), 7.28 (t, *J* = 7.5 Hz, 2H), 7.20 (t, *J* = 7.1 Hz, 1H), 7.07-7.00 (m, 1H), 6.96 (d, *J* = 7.3 Hz, 1H), 6.63 (d, *J* = 8.2 Hz, 1H), 6.58 (t, *J* = 7.3 Hz, 1H), 6.53 (d, *J* = 15.9 Hz, 1H), 6.24 (dt, *J* = 15.9, 5.4 Hz, 1H), 4.02 (dd, *J* = 5.4, 1.5 Hz, 2H), 3.34-3.28 (m, 2H), 2.77 (t, *J* = 6.3 Hz, 2H), 2.04-1.93 (m, 2H).

^{13}C NMR (101 MHz, $CDCl_3$) δ 145.5, 137.1, 131.1, 129.2, 128.6, 127.5, 127.3, 126.4, 125.7, 122.7, 116.0, 111.2, 53.6, 49.29, 28.3, 22.5.

HRMS (m/z, ESI-TOF) calcd for $C_{18}H_{20}N^+$: 250.1590, found 250.1591.

N-cinnamylaniline **(8ba-mono)**

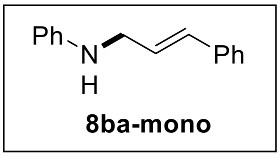

Links NMR CATALOG DETAILS

Following procedure 2-1, the reaction of substrate **2a** (0.2 mmol) for 72 hours afforded **8ba-mono**: 37.2 mg, 89% yield.

^{1}H NMR (400 MHz, $CDCl_3$) δ 7.31-7.20 (m, 4H), 7.18-7.08 (m, 3H), 6.69-6.48 (m, 4H), 6.25 (dt, *J* = 15.9, 5.8 Hz, 1H), 3.86 (dd, *J* = 5.7, 1.2 Hz, 2H).

^{13}C NMR (101 MHz, $CDCl_3$) δ 148.2, 137.0, 131.6, 129.4, 128.7, 127.7, 127.2, 126.5, 117.8, 113.2, 46.3.

HRMS (m/z, ESI-TOF) calcd for $C_{15}H_{16}N^+$: 210.1277, found 210.1280.

N-cinnamyl-3-isopropylaniline **(8ca-mono)**

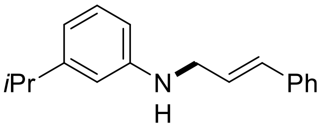

8ca-mono

Links NMR CATALOG DETAILS

Following procedure 2-1, the reaction of substrate **2a** (0.2 mmol) for 72 hours afforded **8ca-mono**: 36.6 mg, 73% yield.

^{1}H NMR (400 MHz, $CDCl_3$) δ 7.29 (d, *J* = 7.3 Hz, 2H), 7.23 (t, *J* = 7.5 Hz, 2H), 7.17-7.14 (m, 1H), 7.05 (t, *J* = 7.8 Hz, 1H), 6.56 (d, *J* = 6.9 Hz, 1H), 6.53 (s, 1H), 6.47 (s, 1H), 6.44-6.41 (m, 1H), 6.26 (dt, *J* = 15.9, 5.8 Hz, 1H), 3.86-3.68 (m, 3H), 2.80-2.68 (m, 1H), 1.16 (s, 3H), 1.15 (s, 3H).

^{13}C NMR (101 MHz, $CDCl_3$) δ 150.3, 148.2, 137.0, 131.7, 129.3, 128.7, 127.6, 127.3, 126.5, 116.1, 111.7, 110.6, 46.5, 34.4, 24.1.

HRMS (m/z, ESI-TOF) calcd for $C_{18}H_{22}N^{+}$: 252.1747, found 252.1750.

N-cinnamyl-2,4,6-trimethylaniline **(8da-mono)**

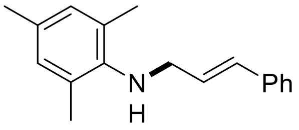

8da-mono

Links NMR CATALOG DETAILS

Following procedure 2-1, the reaction of substrate **2a** (0.2 mmol) for 72 hours afforded **8da-mono**: 36.1 mg, 72% yield.

^{1}H NMR (400 MHz, $CDCl_3$) δ 7.30 (d, *J* = 7.2 Hz, 2H), 7.24 (t, *J* = 7.5 Hz, 2H), 7.17-7.14 (m, 1H), 6.77 (s, 2H), 6.53 (d, *J* = 15.8 Hz, 1H), 6.29 (dt, *J* = 15.8, 6.4 Hz, 1H), 3.63 (dd, *J* = 6.4, 1.0 Hz, 2H), 3.01 (brs, 1H), 2.22 (s, 6H), 2.17 (s, 3H).

^{13}C NMR (101 MHz, $CDCl_3$) δ 143.1, 137.1, 131.8, 131.4, 130.2, 129.6, 128.7, 128.2, 127.6, 126.5, 51.3, 20.7, 18.5.

HRMS (m/z, ESI-TOF) calcd for $C_{18}H_{22}N^{+}$: 252.1747, found 252.1750.

N-cinnamyl-4-(pyridin-2-yl)aniline **(8ea-mono)**

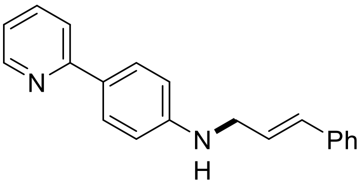

8ea-mono

Links NMR CATALOG DETAILS

Following procedure 2-1, the reaction of substrate **2a** (0.2 mmol) for 72 hours afforded **8ea-mono**: 47.4 mg, 83% yield.

^{1}H NMR (400 MHz, $CDCl_3$) δ 8.55-8.54 (m, 1H), 7.84-7.77 (m, 2H), 7.63-7.52 (m, 2H), 7.32-7.26 (m, 2H), 7.25-7.21 (m, 2H), 7.17-7.13 (m, 1H), 7.03 (ddd, J = 7.0, 4.9, 1.3 Hz, 1H), 6.70-6.64 (m, 2H), 6.56 (d, J = 15.9 Hz, 1H), 6.26 (dt, J = 15.9, 5.8 Hz, 1H), 3.92 (dd, J = 5.7, 1.5 Hz, 2H).
^{13}C NMR (101 MHz, $CDCl_3$) δ 157.6, 149.4, 149.0, 136.9, 136.8, 131.9, 128.7, 128.2, 127.7, 126.7, 126.5, 120.9, 119.4, 113.1, 46.1.
HRMS (m/z, ESI-TOF) calcd for $C_{20}H_{19}N_2^+$: 287.1543, found 287.1545.

N-cinnamyl-2-fluoroaniline **(8fa-mono)**

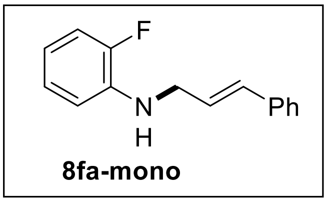

Links NMR CATALOG DETAILS

Following procedure 2-1, the reaction of substrate **2a** (0.2 mmol) for 72 hours afforded **8fa-mono**: 30.8 mg, 68% yield.
^{1}H NMR (400 MHz, $CDCl_3$) δ 7.37 (d, J = 7.3 Hz, 2H), 7.30 (t, J = 7.5 Hz, 2H), 7.24-7.21 (m, 1H), 7.03-6.94 (m, 2H), 6.77-6.72 (m, 1H), 6.67-6.59 (m, 2H), 6.31 (dt, J = 15.9, 5.7 Hz, 1H), 4.13 (brs, 1H), 3.97 (d, J = 5.7 Hz, 2H).
^{19}F NMR (376 MHz, $CDCl_3$) δ -136.49 (ddd, J = 16.1, 8.5, 4.2 Hz).
^{13}C NMR (101 MHz, $CDCl_3$) δ 151.8 (d, J_{C-F} = 238.3 Hz), 136.9, 136.6 (d, J_{C-F} = 11.5 Hz), 131.9, 128.7, 127.7, 126.7, 126.5, 124.7 (d, J_{C-F} = 3.4 Hz), 117.0 (d, J_{C-F} = 7.0 Hz), 114.6 (d, J_{C-F} = 18.5 Hz), 112.6 (d, J_{C-F} = 3.2 Hz). 45.9.
HRMS (m/z, ESI-TOF) calcd for $C_{15}H_{15}FN^+$: 228.1183, found 228.1185.

3-bromo-*N*-cinnamylaniline **(8ga-mono)**

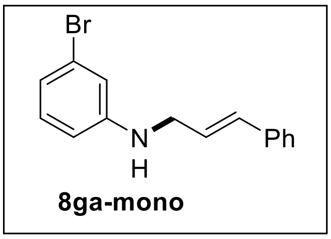

Links NMR CATALOG DETAILS

Following procedure 2-1, the reaction of substrate **2a** (0.2 mmol) for 72 hours afforded **8ga-mono**: 44.2 mg, 77% yield.
^{1}H NMR (400 MHz, $CDCl_3$) δ 7.39-7.29 (m, 4H), 7.25-7.21 (m, 1H), 7.02 (t, J = 8.0 Hz, 1H), 6.84-6.78 (m, 2H), 6.61 (d, J = 15.9 Hz, 1H), 6.56 (dd, J = 8.2, 1.7 Hz, 1H), 6.28 (dt, J = 15.9, 5.7 Hz, 1H), 3.95-3.87 (m, 3H).
^{13}C NMR (101 MHz, $CDCl_3$) δ 149.4, 136.8, 132.0, 130.7, 128.7, 127.8, 126.5, 126.3, 123.4, 120.5, 115.7, 111.8, 46.1.
HRMS (m/z, ESI-TOF) calcd for $C_{15}H_{15}BrN^+$: 288.0382, found 288.0384.

4,5-dichloro-*N*-cinnamyl-2-nitroaniline **(8ha-mono)**

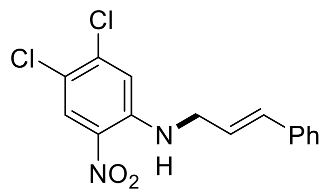

8ha-mono

Links NMR CATALOG DETAILS

Following procedure 2-1, the reaction of substrate **2a** (0.2 mmol) for 72 hours afforded **8ha-mono**: 28.4 mg, 44% yield.

^{1}H NMR (400 MHz, $CDCl_3$) 1H NMR (400 MHz, CDCl3) δ 8.30 (s, 1H), 8.15 (brs, 1H), 7.41-7.26 (m, 5H), 7.02 (s, 1H), 6.64 (d, *J* = 15.9 Hz, 1H), 6.26 (dt, *J* = 15.9, 5.6 Hz, 1H), 4.12 (t, *J* = 5.5 Hz, 2H).

^{13}C NMR (101 MHz, $CDCl_3$) δ 144.0, 141.3, 136.1, 133.2, 131.0, 128.9, 128.3, 127.9, 126.7, 123.6, 119.3, 115.5, 45.4.

HRMS (m/z, ESI-TOF) calcd for $C_{15}H_{13}Cl_2N_2O_2^+$: 323.0349, found 323.0346.

N-cinnamyl-2-fluoro-6-methylaniline **(8ia-mono)**

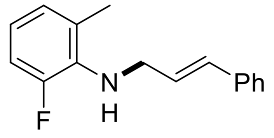

8ia-mono

Links NMR CATALOG DETAILS

Following procedure 2-1, the reaction of substrate **2a** (0.2 mmol) for 72 hours afforded **8ia-mono**: 44.9 mg, 93% yield.

^{1}H NMR (400 MHz, $CDCl_3$) δ 7.37 (d, *J* = 7.3 Hz, 2H), 7.31 (t, *J* = 7.5 Hz, 2H), 7.23 (t, *J* = 6.7 Hz, 1H), 6.96 (t, *J* = 7.2 Hz, 1H), 6.61 (d, *J* = 15.9 Hz, 1H), 6.40-6.27 (m, 3H), 3.92 (dd, *J* = 5.8, 1.0 Hz, 2H), 3.79 (brs, 1H), 2.10 (s, 3H).

^{13}C NMR (101 MHz, $CDCl_3$) δ 163.0 (d, $J_{C\text{-}F}$ = 240.2 Hz), 147.4 (d, $J_{C\text{-}F}$ = 10.6 Hz), 136.8, 132.1, 130.7 (d, $J_{C\text{-}F}$ = 9.8 Hz), 128.7, 127.8, 126.5, 126.4, 117.4 (d, $J_{C\text{-}F}$ = 2.6 Hz), 102.9 (d, $J_{C\text{-}F}$ = 21.2 Hz), 97.6 (d, $J_{C\text{-}F}$ = 26.3 Hz), 46.2, 17.0.

HRMS (m/z, ESI-TOF) calcd for $C_{16}H_{17}FN^+$: 242.1340, found 242.1338.

(2-(cinnamylamino)phenyl)(phenyl)methanone **(8ja-mono)**

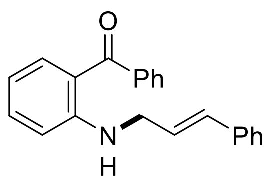

8ja-mono

Links NMR CATALOG DETAILS

Following procedure 2-1, the reaction of substrate **2a** (0.2 mmol) for 72 hours afforded **8ja-mono**: 56.0 mg, 89% yield.

^{1}H NMR (400 MHz, $CDCl_3$) δ 8.89 (brs, 1H), 7.71-7.67 (m, 2H), 7.59-7.54 (m, 2H), 7.53-7.48 (m, 2H), 7.47-7.41 (m, 3H), 7.39-7.35 (m, 2H), 7.31-7.26 (m, 1H), 6.88 (d, J = 8.4 Hz, 1H), 6.72 (d, J = 15.9 Hz, 1H), 6.65-6.59 (m, 1H), 6.40 (dt, J = 15.9, 5.5 Hz, 1H), 4.15 (t, J = 4.7 Hz, 2H).

^{13}C NMR (101 MHz, $CDCl_3$) δ 199.5, 151.7, 140.6, 136.9, 135.6, 135.1, 131.6, 130.9, 129.1, 128.6, 128.1, 127.6, 126.5, 126.2, 117.6, 114.2, 112.1, 44.9.

HRMS (m/z, ESI-TOF) calcd for $C_{22}H_{20}NO^{+}$: 314.1539, found 314.1537.

N,*N*-dicinnamylaniline **(8ba-di)**

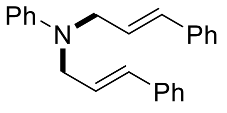

8ba-di

Links NMR CATALOG DETAILS

Following procedure 2-1, the reaction of substrate **6b'** (0.2 mmol) for 84 hours afforded **8ba-di**: 54.0 mg, 83% yield.

^{1}H NMR (400 MHz, $CDCl_3$) δ 7.29 (d, *J* = 7.4 Hz, 4H), 7.22 (t, *J* = 7.5 Hz, 4H), 7.19-7.12 (m, 4H), 6.75 (d, *J* = 8.2 Hz, 2H), 6.65 (t, *J* = 7.2 Hz, 1H), 6.47 (d, *J* = 15.9 Hz, 2H), 6.21 (dt, *J* = 15.9, 5.2 Hz, 2H), 4.06 (d, *J* = 4.8 Hz, 4H).

^{13}C NMR (101 MHz, $CDCl_3$) δ 149.0, 137.0, 131.3, 129.4, 128.7, 127.6, 126.5, 126.0, 116.7, 112.7, 52.3.

HRMS (m/z, ESI-TOF) calcd for $C_{24}H_{24}N^{+}$: 326.1903, found 326.1906.

N,*N*-dicinnamyl-3-isopropylaniline **(8ca-di)**

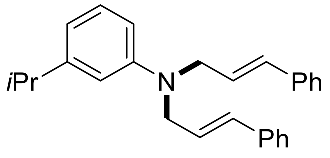

8ca-di

Links NMR CATALOG DETAILS

Following procedure 2-1, the reaction of substrate **6c'** (0.2 mmol) for 84 hours afforded **8ca-di**: 57.9 mg, 79% yield.

^{1}H NMR (400 MHz, $CDCl_3$) δ 7.29 (d, *J* = 7.3 Hz, 4H), 7.22 (t, *J* = 7.5 Hz, 4H), 7.17-7.07 (m, 3H), 6.65-6.53 (m, 3H), 6.48 (d, *J* = 15.9 Hz, 2H), 6.21 (dt, *J* = 15.8, 5.2 Hz, 2H), 4.06 (d, *J* = 4.9 Hz, 4H), 2.85-2.69 (m, 1H), 1.17 (s, 3H), 1.16 (s, 3H).

^{13}C NMR (101 MHz, $CDCl_3$) δ 150.2, 149.1, 137.1, 131.4, 129.3, 128.7, 127.5, 126.5, 126.3, 115.0, 111.2, 110.5, 52.4, 34.7, 24.2.

HRMS (m/z, ESI-TOF) calcd for $C_{27}H_{30}N^{+}$: 368.2373, found 368.2369.

N,*N*-dicinnamyl-2,4,6-trimethylaniline **(8da-di)**

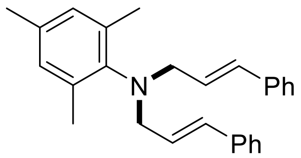

8da-di

Links NMR CATALOG DETAILS

Following procedure 2-1, the reaction of substrate **6d'** (0.2 mmol) for 84 hours afforded **8da-di**: 59.9 mg, 82% yield.

^{1}H NMR (400 MHz, $CDCl_3$) δ 7.35-7.26 (m, 8H), 7.20 (t, *J* = 7.0 Hz, 2H), 6.84 (s, 2H), 6.46 (d, *J* = 15.8 Hz, 2H), 6.30-6.18 (m, 2H), 3.80 (d, *J* = 6.6 Hz, 4H), 2.33 (s, 6H), 2.24 (s, 3H).

^{13}C NMR (101 MHz, $CDCl_3$) δ 145.9, 137.5, 137.2, 134.6, 131.3, 129.7, 129.0, 128.6, 127.3, 126.4, 55.6, 20.9, 19.8.

HRMS (m/z, ESI-TOF) calcd for $C_{27}H_{30}N^+$: 368.2373, found 368.2369.

N,*N*-dicinnamyl-4-(pyridin-2-yl)aniline **(8ea-di)**

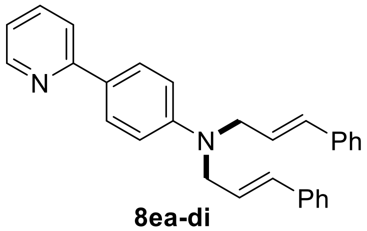

8ea-di

Links NMR CATALOG DETAILS

Following procedure 2-1, the reaction of substrate **6e'** (0.2 mmol) for 84 hours afforded **8ea-di**: 73.1 mg, 91% yield.

^{1}H NMR (400 MHz, $CDCl_3$) δ 8.55-8.52 (m, 1H), 7.82 (d, *J* = 9.0 Hz, 2H), 7.62-7.53 (m, 2H), 7.31-7.27 (m, 4H), 7.22 (t, *J* = 7.5 Hz, 4H), 7.18-7.12 (m, 2H), 7.02 (ddd, *J* = 6.6, 4.9, 1.5 Hz, 1H), 6.82 (d, *J* = 9.0 Hz, 2H), 6.47 (d, *J* = 15.9 Hz, 2H), 6.21 (dt, *J* = 15.9, 5.2 Hz, 2H), 4.14-4.10 (m, 4H).

^{13}C NMR (101 MHz, $CDCl_3$) δ 157.6, 149.5, 149.4, 136.9, 136.7, 131.5, 128.7, 128.1, 127.6, 127.6, 126.5, 125.5, 120.8, 119.4, 112.6, 52.3.

HRMS (m/z, ESI-TOF) calcd for $C_{29}H_{27}N_2^+$: 403.2169, found 403.2167.

3-(*tert*-butyl)-*N*,*N*-dicinnamylaniline **(8la-di)**

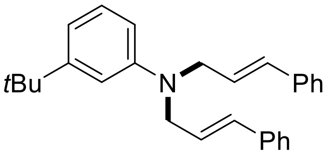

8la-di

Links NMR CATALOG DETAILS

Following procedure 2-1, the reaction of substrate **6l'** (0.2 mmol) for 84 hours afforded **8la-di**: 58.6 mg, 77% yield.

^{1}H NMR (400 MHz, $CDCl_3$) δ 7.28 (d, J = 7.2 Hz, 4H), 7.22 (t, J = 7.5 Hz, 4H), 7.17-7.08 (m, 3H), 6.81 (s, 1H), 6.71 (d, J = 7.6 Hz, 1H), 6.60 (d, J = 8.1 Hz, 1H), 6.50 (d, J = 15.9 Hz, 2H), 6.21 (dt, J = 15.9, 5.4 Hz, 2H), 4.07 (d, J = 4.6 Hz, 4H), 1.23 (s, 9H).
^{13}C NMR (101 MHz, $CDCl_3$) δ 152.3, 148.9, 137.1, 131.5, 129.0, 128.7, 127.5, 126.5, 114.2, 110.4, 110.3, 52.7, 35.0, 31.6.
HRMS (m/z, ESI-TOF) calcd for $C_{28}H_{32}N^+$: 382.2529, found 382.2533.

N,*N*-dicinnamylquinolin-6-amine **(8ma-di)**

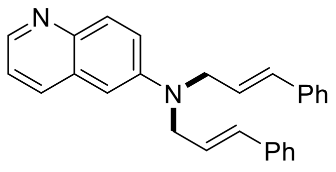

8ma-di

Links NMR CATALOG DETAILS

Following procedure 2-1, the reaction of substrate **6m'** (0.2 mmol) for 84 hours afforded **8m-di**: 46.2 mg, 61% yield.
^{1}H NMR (400 MHz, $CDCl_3$) δ 8.56 (d, J = 2.9 Hz, 1H), 7.90 (dd, J = 15.8, 8.8 Hz, 2H), 7.37-7.27 (m, 5H), 7.27-7.13 (m, 8H), 6.86 (d, J = 2.5 Hz, 1H), 6.51 (d, J = 15.9 Hz, 2H), 6.25 (dt, J = 15.9, 5.0 Hz, 2H), 4.20 (d, J = 4.5 Hz, 4H).
^{13}C NMR (101 MHz, $CDCl_3$) δ 147.1, 146.2, 136.8, 134.5, 131.7, 130.1, 128.7, 127.8, 126.5, 125.2, 121.5, 119.7, 105.2, 52.5.
HRMS (m/z, ESI-TOF) calcd for $C_{27}H_{24}N_2Na^+$: 399.1832, found 399.1829.

N-cinnamyl-2,4,6-trimethyl-*N*-((*E*)-3-(perfluorophenyl)allyl)aniline **(8dam)**

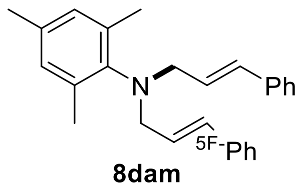

8dam

Links NMR CATALOG DETAILS

Following procedure 2-2, the reaction of substrate **6d** (0.2 mmol) afforded **8dam**: 40.3 mg, 44% yield.
^{1}H NMR (400 MHz, $CDCl_3$) δ 7.35-7.27 (m, 4H), 7.22-7.19 (m, 1H), 6.84 (s, 2H), 6.57 (dt, J = 16.2, 6.4 Hz, 1H), 6.46 (d, J = 15.8 Hz, 1H), 6.37 (d, J = 16.3 Hz, 1H), 6.24 (dt, J = 15.8, 6.8 Hz, 1H), 3.84 (d, J = 6.4 Hz, 2H), 3.79 (d, J = 6.7 Hz, 2H), 2.32 (s, 6H), 2.24 (s, 3H).
^{13}C NMR (101 MHz, $CDCl_3$) 145.6, 139.0, 137.3, 137.1, 135.0, 131.8, 129.8, 128.7, 128.5, 127.5, 126.4, 115.1, 56.3, 56.1, 20.9, 19.7.
^{19}F NMR (376 MHz, $CDCl_3$) δ -143.36 (dd, J_{F-F} = 21.7, 7.8 Hz), -157.26 (t, J_{F-F} = 20.8 Hz), -163.29 (dt, J_{F-F} = 21.6, 8.0 Hz).
HRMS (m/z, ESI-TOF) calcd for $C_{27}H_{25}F_5N^+$: 458.1902, found 458.1899.

N-cinnamyl-*N*-((*E*)-3-cyclohexylallyl)-2,4,6-trimethylaniline **(8dax)**

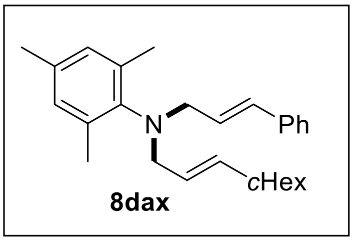

Links NMR CATALOG DETAILS

Following procedure 2-2, the reaction of substrate **6d** (0.2 mmol) afforded **8dax**: 31.1 mg, 42% yield.

^{1}H NMR (400 MHz, $CDCl_3$) δ 7.34-7.27 (m, 4H), 7.21-7.18 (m, 1H), 6.81 (s, 2H), 6.42 (d, *J* = 15.8 Hz, 1H), 6.20 (dt, *J* = 15.7, 6.7 Hz, 1H), 5.49-5.36 (m, 2H), 3.74 (d, *J* = 6.6 Hz, 2H), 3.56 (d, *J* = 5.7 Hz, 2H), 2.28 (s, 6H), 2.23 (s, 3H), 1.95-1.87 (m, 1H), 1.71-1.59 (m, 4H), 1.30-0.96 (m, 6H).

^{13}C NMR (101 MHz, $CDCl_3$) δ 146.2, 138.8, 137.7, 137.2, 134.3, 130.9, 129.5, 129.4, 128.6, 127.2, 126.4, 125.8, 55.5, 55.2, 40.5, 33.2, 26.4, 26.2, 20.9, 19.8.

HRMS (m/z, ESI-TOF) calcd for $C_{27}H_{36}N^+$: 374.2842, found 374.2839.

N-cinnamyladamantan-1-amine **(9aa)**

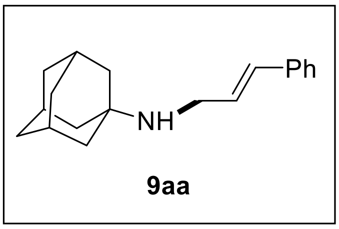

Links NMR CATALOG DETAILS

Following procedure 2-1, the reaction of substrate **6a''** (0.2 mmol) for 84 hours afforded **9aa**: 49.2 mg, 92% yield.

^{1}H NMR (400 MHz, $CDCl_3$) δ 7.37-7.35 (m, 2H), 7.30-7.26 (m, 2H), 7.22-7.18 (m, 1H), 6.52 (d, *J* = 15.9 Hz, 1H), 6.34 (dt, *J* = 15.8, 6.4 Hz, 1H), 3.41 (d, *J* = 6.2 Hz, 2H), 2.08 (s, 3H), 1.70-1.61 (m, 13H).

^{13}C NMR (101 MHz, $CDCl_3$) δ 137.4, 131.1, 129.5, 128.6, 127.4, 126.4, 51.1, 43.3, 42.9, 36.9, 29.8.

HRMS (m/z, ESI-TOF) calcd for $C_{19}H_{26}N^+$: 268.2060, found 268.2061.

Methyl *(R)*-3-(4-(cinnamyloxy)phenyl)-2-(dicinnamylamino)propanoate **(9ba)**

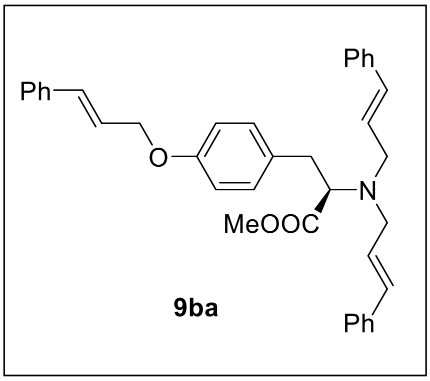

Links NMR CATALOG DETAILS

Following procedure 2-4, the reaction of substrate **6b''** (0.2 mmol) for 72 hours with O_2 balloon afforded **9ba**: 14.6 mg, 27% yield.

^{1}H NMR (400 MHz, $CDCl_3$) δ 7.41 (d, *J* = 7.3 Hz, 2H), 7.37-7.26 (m, 11H), 7.24-7.19 (m, 2H), 7.11 (d, *J* = 8.6 Hz, 2H), 6.87 (d, *J* = 8.6 Hz, 2H), 6.74 (d, *J* = 16.0 Hz, 1H), 6.49-6.38 (m, 3H), 6.08 (ddd, *J* = 15.8, 7.3, 5.3 Hz, 2H), 4.68 (dd, *J* = 5.8, 1.2 Hz, 2H), 3.76 (t, *J* = 7.6 Hz, 1H), 3.66 (s, 3H), 3.58 (d, *J* = 5.2 Hz, 1H), 3.55 (d, *J* = 3.8 Hz, 1H). 3.32 (dd, *J* = 14.7, 7.4 Hz, 2H), 3.05 (dd, *J* = 13.8, 7.4 Hz, 1H), 2.91 (dd, *J* = 13.8, 7.7 Hz, 1H).
^{13}C NMR (101 MHz, $CDCl_3$) δ 173.4, 157.4, 137.3, 136.6, 133.1, 132.4, 131.0, 130.5, 128.7, 128.7, 128.1, 127.5, 126.7, 126.4, 124.7, 114.7, 68.8, 64.4, 53.1, 51.4, 35.1.
HRMS (m/z, ESI-TOF) calcd for $C_{37}H_{38}NO_3^+$: 544.2846, found 544.2841.

3. Supplementary mechanism study

3.1 Supplementary control experiments

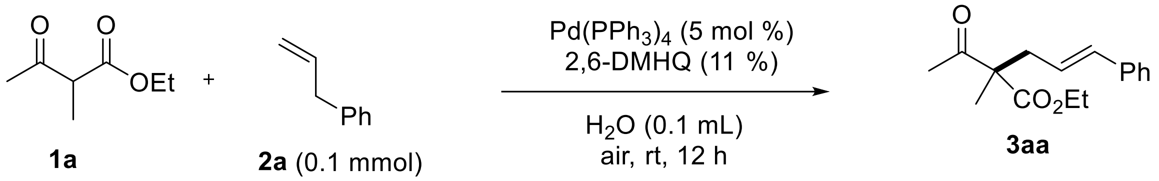

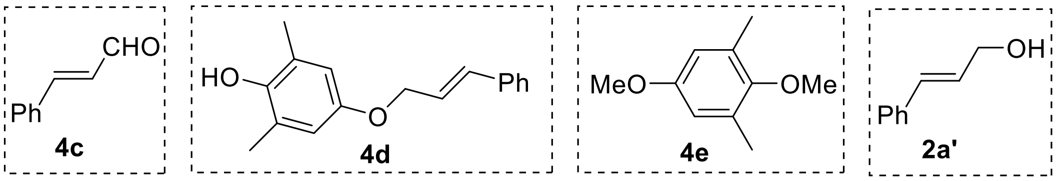

Entry	Conditions	Yield (%)
1	standard conditions	43 (**3aa**), trace (**4c**)
2	without 2,6-DMHQ	11 (**3aa**), 5 (**4c**)
3	$Pd(OAc)_2/4PPh_3$ (5 mol %) instead of $Pd(PPh_3)_4$	41 (**3aa**), trace (**4c**)
4	$Pd(OAc)_2/4POPh_3$ (5 mol %) instead of $Pd(PPh_3)_4$	Trace (**3aa**), 7 (**4c**)
5	$Pd_2(dba)_3$ (2.5 mol %) instead of $Pd(PPh_3)_4$	Trace (**3aa**, **4c**)
6	[2,6-DMBQ (1.2 eq.), N_2] instead of [2,6-DMHQ, air]	Trace (**3aa**, **4c**)
7	[2,6-DMBQ (1.2 eq.), air] instead of [2,6-DMHQ, air]	52 (**3aa**)
8	**4d** (11 mol %) instead of 2,6-DMHQ	8 (**3aa**), 5 (**4c**)
9	**4e** (11 mol %) instead of 2,6-DMHQ	Trace (**3aa**), 6 (**4c**)
10	**2a’** instead of **2a**	Trace (**3aa**, **4c**)

Figure 13 Control experiment of on-water alkylation: following Procedure 1-1 for 12 h; ^{1}H NMR yield with CH_2Br_2.

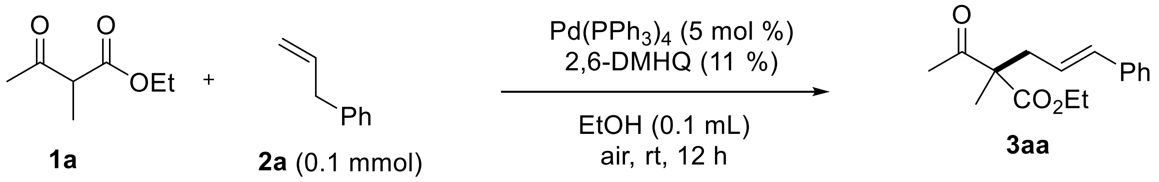

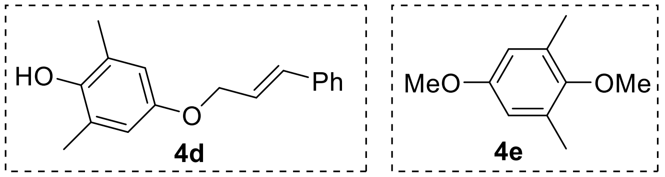

Entry	Conditions	Yield (%)
1	standard conditions	47
2	without 2,6-DMHQ	trace
3	without 2,6-DMHQ，50 °C instead of rt	4
4	**4d** (11 mol %) instead of 2,6-DMHQ	trace

5	**4e** (11 mol %) instead of 2,6-DMHQ	trace

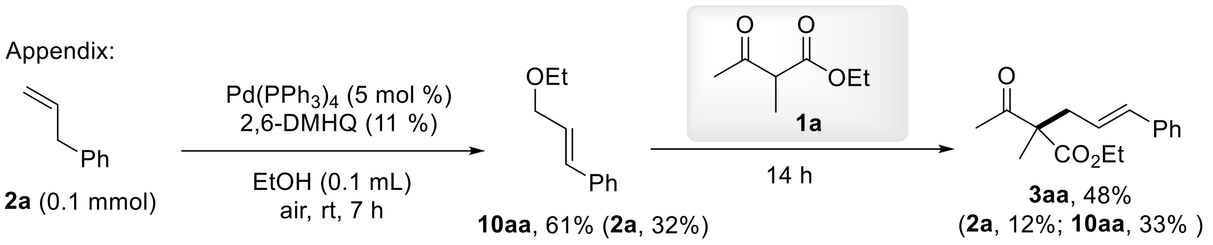

Figure 14 Control experiment of in-ethanol alkylation: following <u>procedure 1-2</u> for 12 h; ^{1}H NMR yield with CH_2Br_2.

3.2 The K_H/K_D experiments

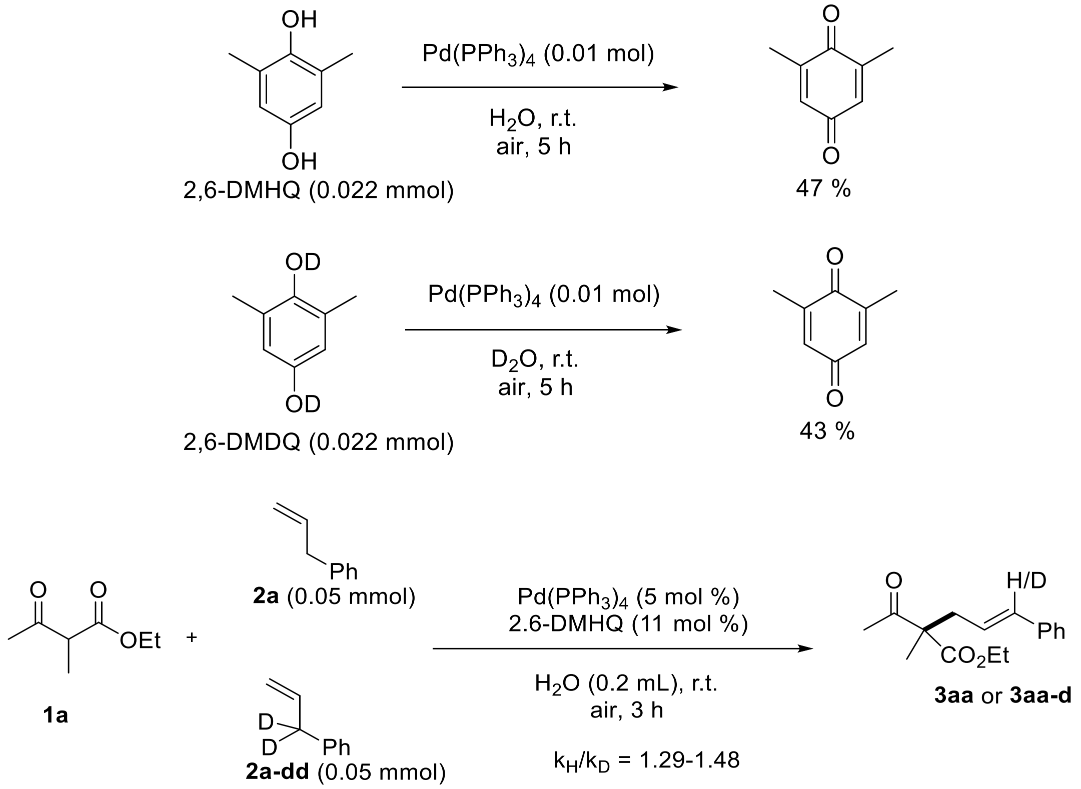

Figure 15 K_H/K_D experiment: the deuterated hydrazine obtained from overnight stirring with deuterated water [1-2] was directly used for the reaction with other substrates and catalysts; the yield was determined by GC with tridecane as internal standard. Repeated trial: 1st, 2,6-DMHQ (43%)/2,6-DMDQ (45%);2nd, 2,6-DMHQ (46%)/2,6-DMDQ (39%);3rd, 3h, 2,6-DMHQ (33%)/2,6-DMDQ (31%).

3.3 Proposed mechanism

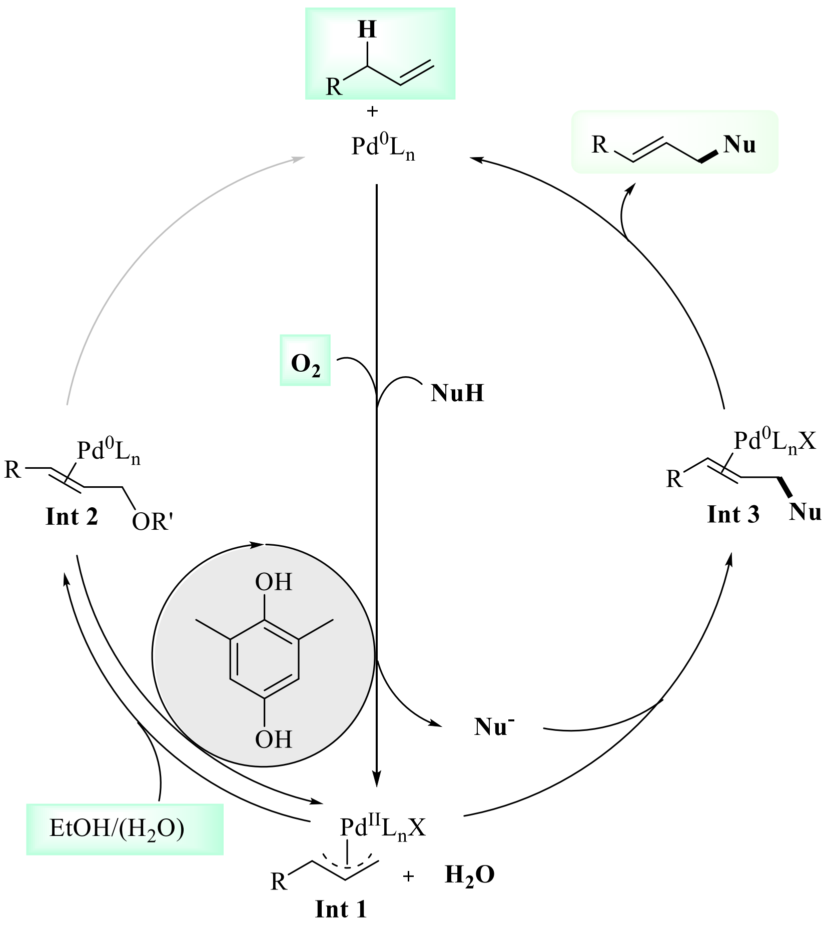

Figure 16 Plausible mechanism.

4. Comparison with conventional protocol

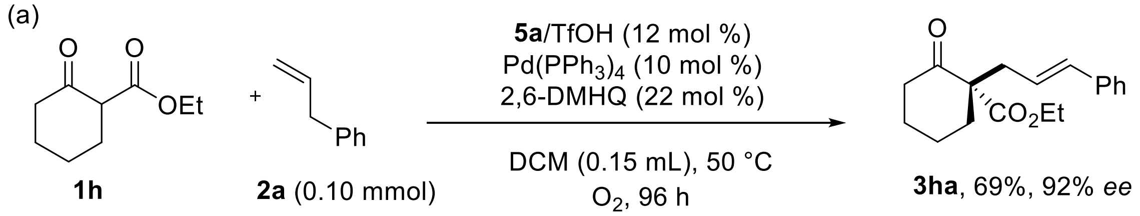

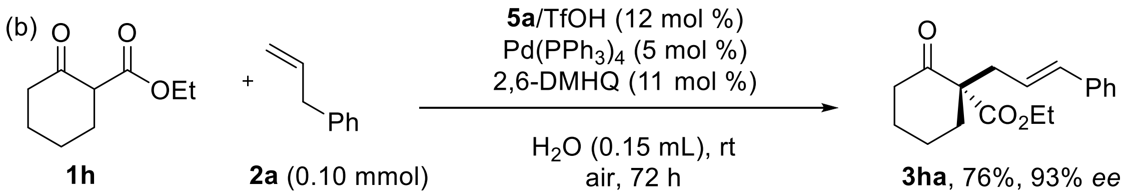

Figure 17 Stereocontrol synthesis of **3ha**: (a) conventional protocol [3]; (b) this work.

According to the E-factor calculated based on the solvent, it accounts for 10% [4]. Our new green paradigm has slightly decreased the E-factor, but the greatly simplified post-treatment and the absence of the need for heating have significantly reduced the energy consumption of the reaction and purification processes.

Links DETAILS NMR HPLC E-Fact

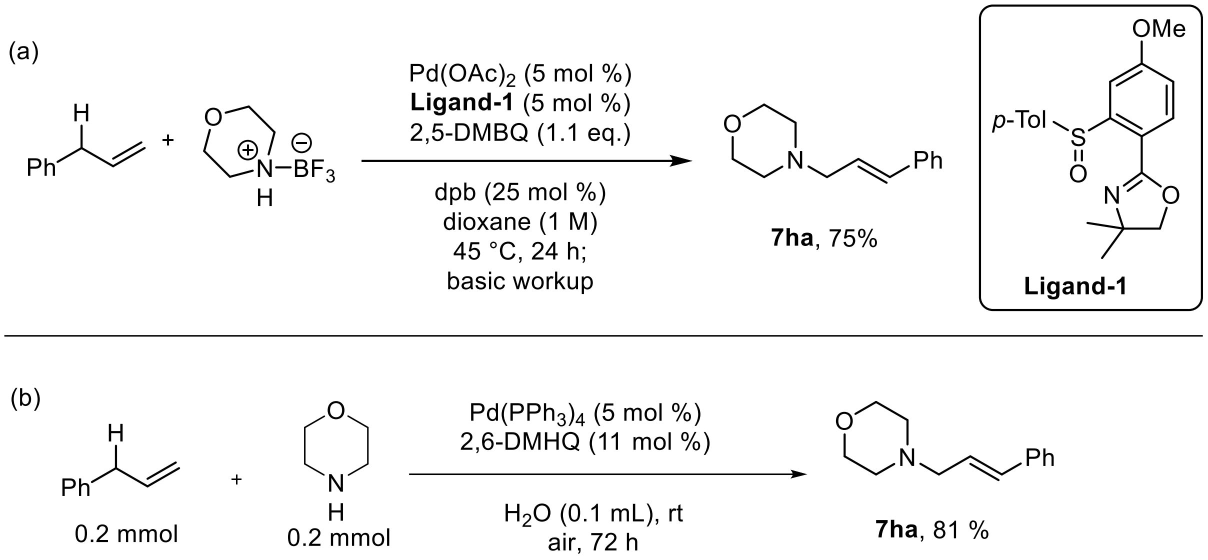

Figure 18 Synthesis of **7ha**: (a) conventional protocol [5]; (b) this work.

-Energy consumption: the greatly simplified post-treatment and the absence of the need for heating have significantly reduced the energy consumption of the reaction and purification processes.

-Chemical consumption: slightly decrease due to use of alternative oxidant and solvent.

-E-Factor: (a) 2.53; (b) 0.59.

Links DETAILS NMR E-Fact

6. NMR spectra

^{1}H NMR spectrum of compound **3aa** ($CDCl_3$, 400 MHz)

Links CATALOG DETAILS NMR HPLC

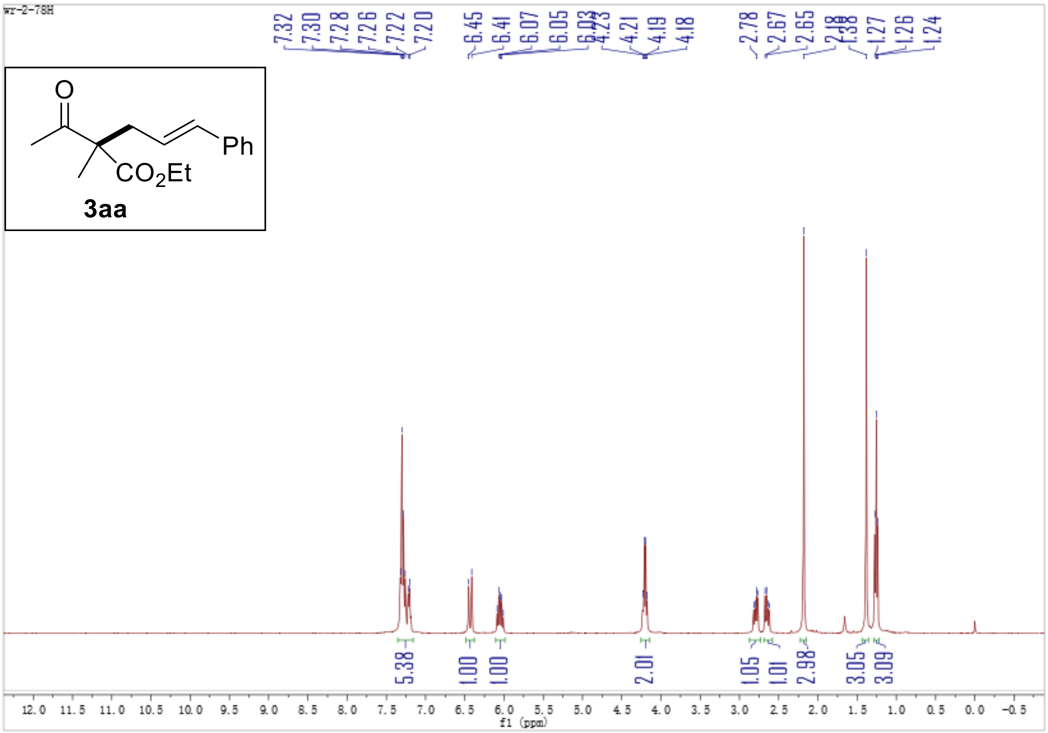

^{13}C NMR spectrum of compound **3aa** ($CDCl_3$, 101 MHz)

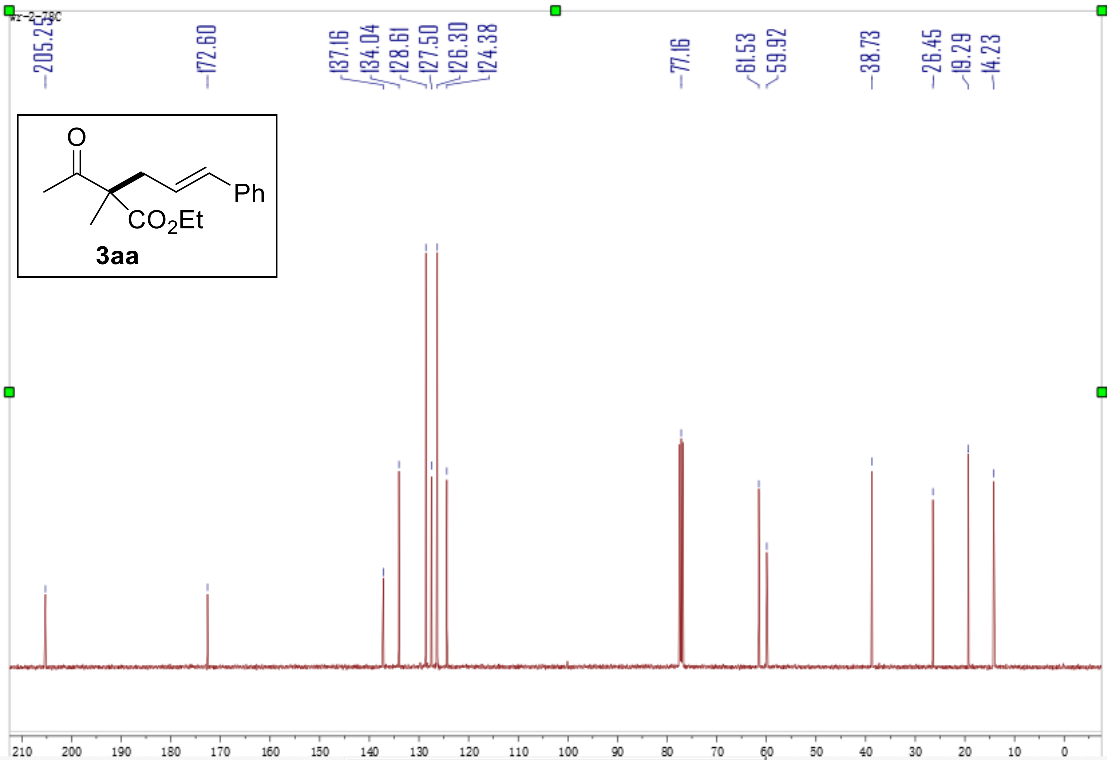

[1]**H NMR** spectrum of compound **3aa-*Z*** ($CDCl_3$, 400 MHz)

Links CATALOG DETAILS NMR

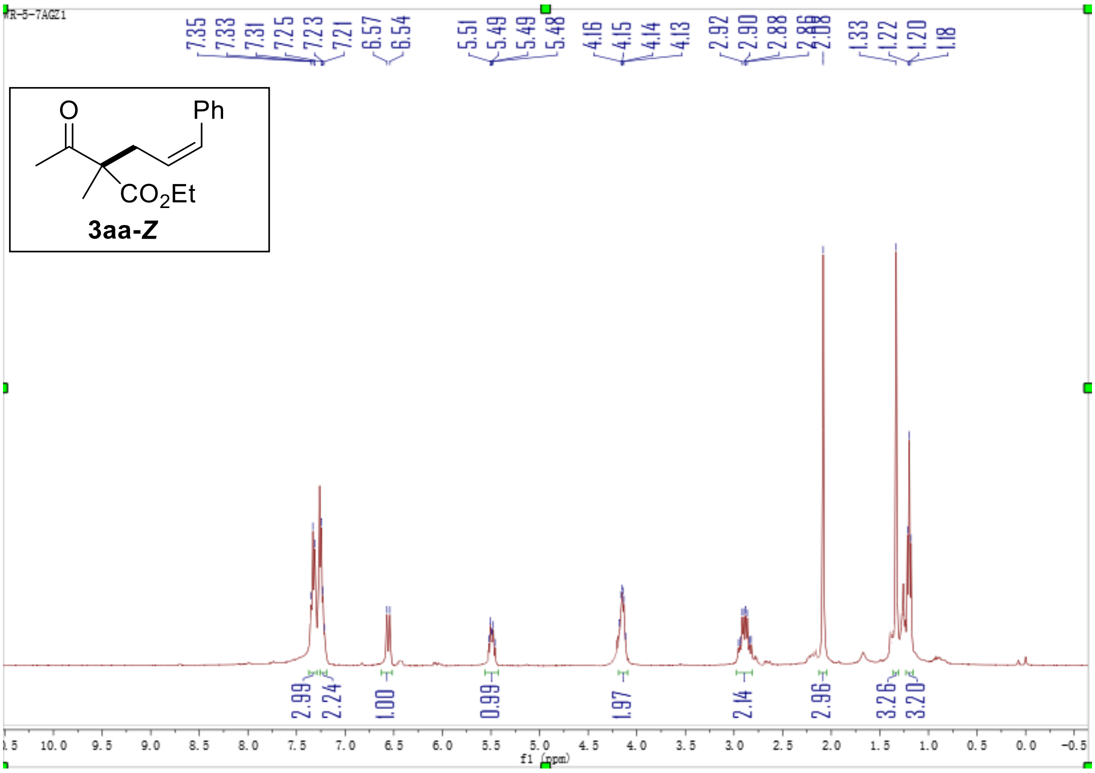

[13]**C NMR** spectrum of compound **3aa-*Z*** ($CDCl_3$, 101 MHz)

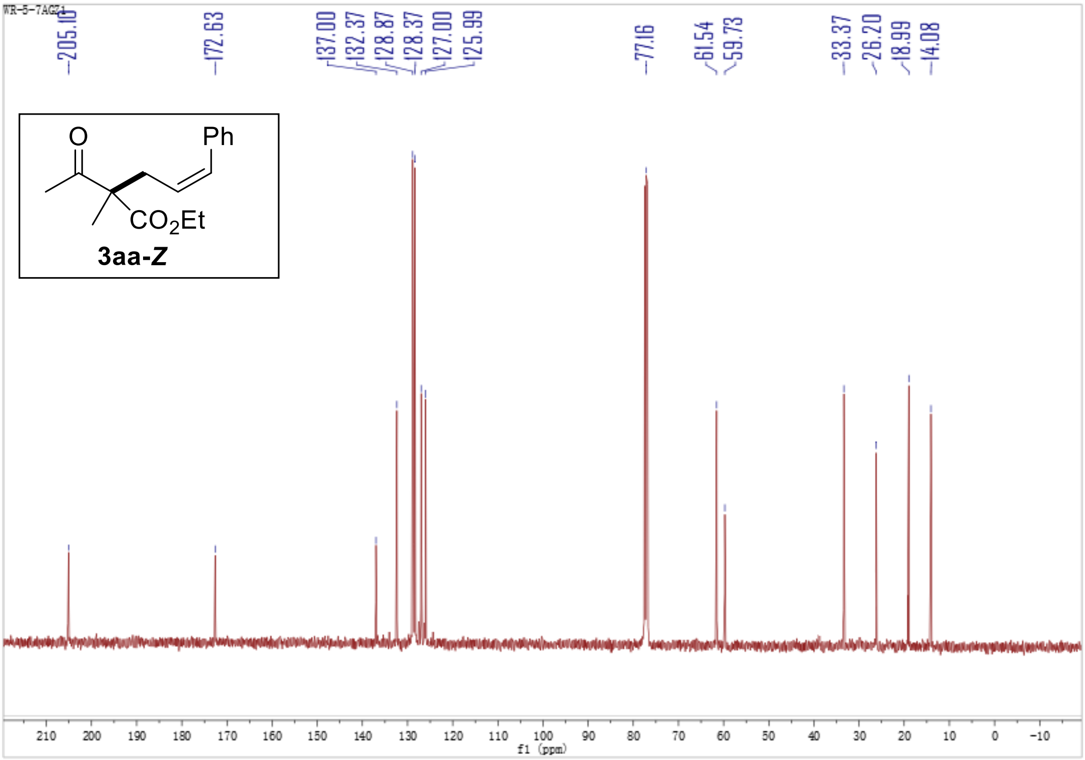

NOE spectrum of compound **3aa-*Z*** ($CDCl_3$, 400 MHz)

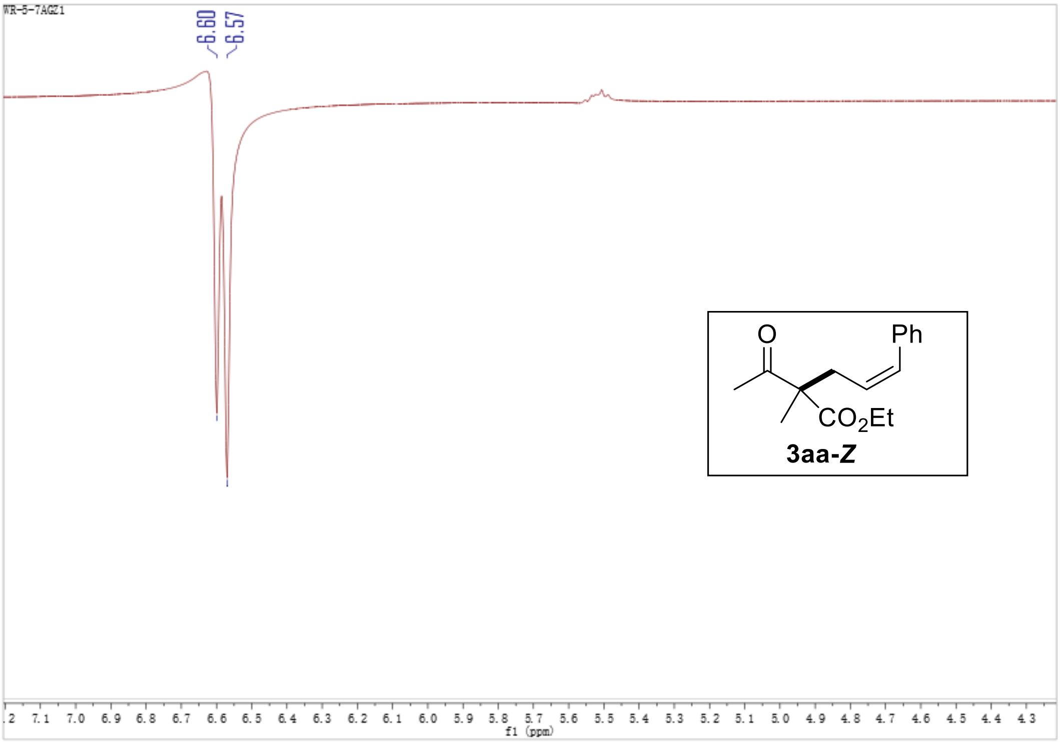

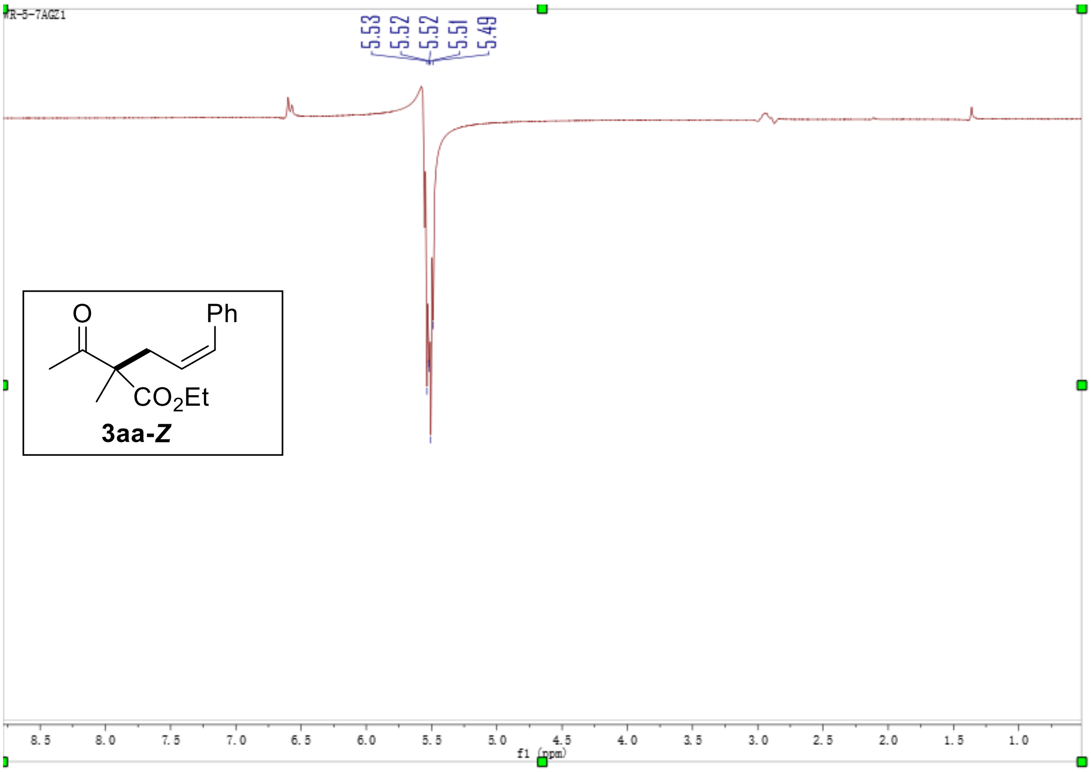

^{1}H-^{1}H COSY spectrum of compound **3aa-*Z*** ($CDCl_3$, 400 MHz)

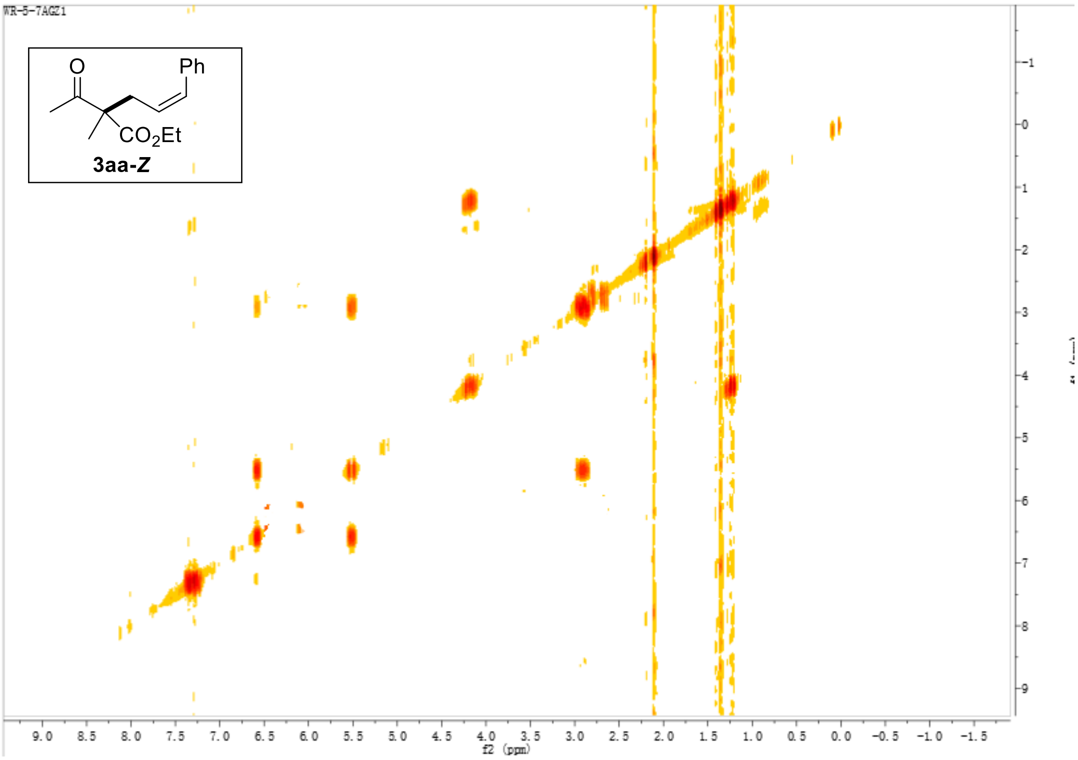

^{1}H NMR spectrum of compound **3ba** ($CDCl_3$, 400 MHz)

Links CATALOG DETAILS NMR

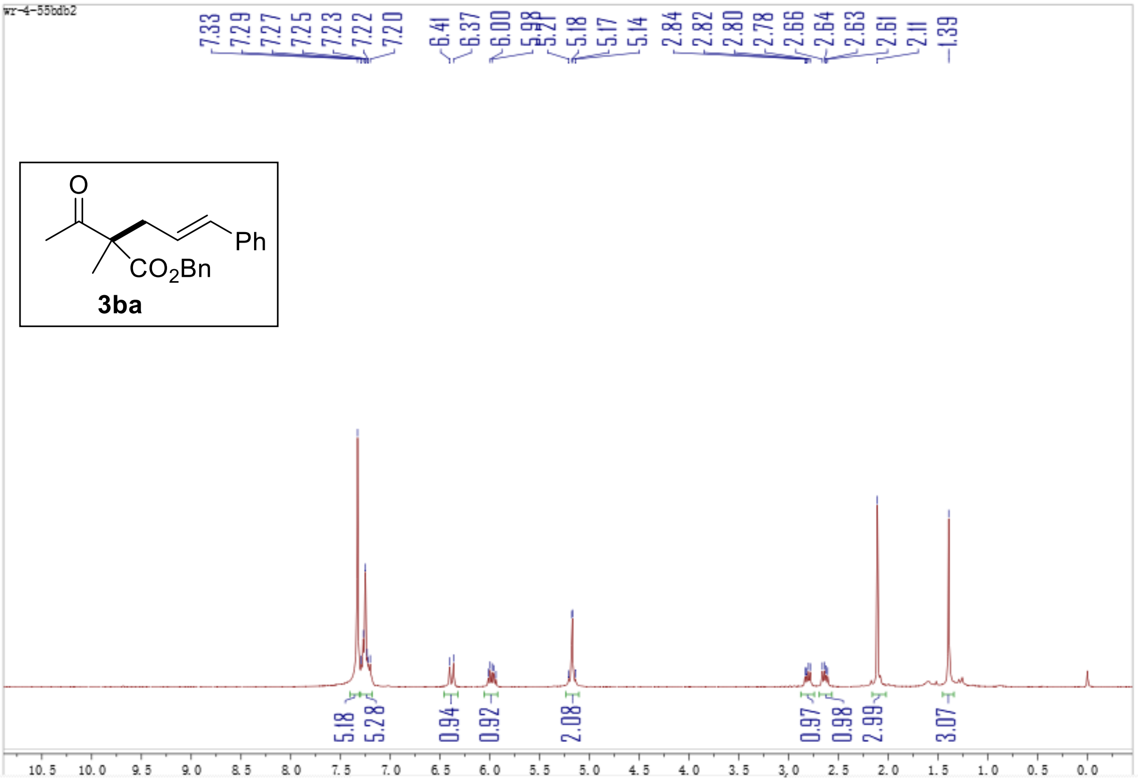

^{13}C NMR spectrum of compound **3ba** ($CDCl_3$, 101 MHz)

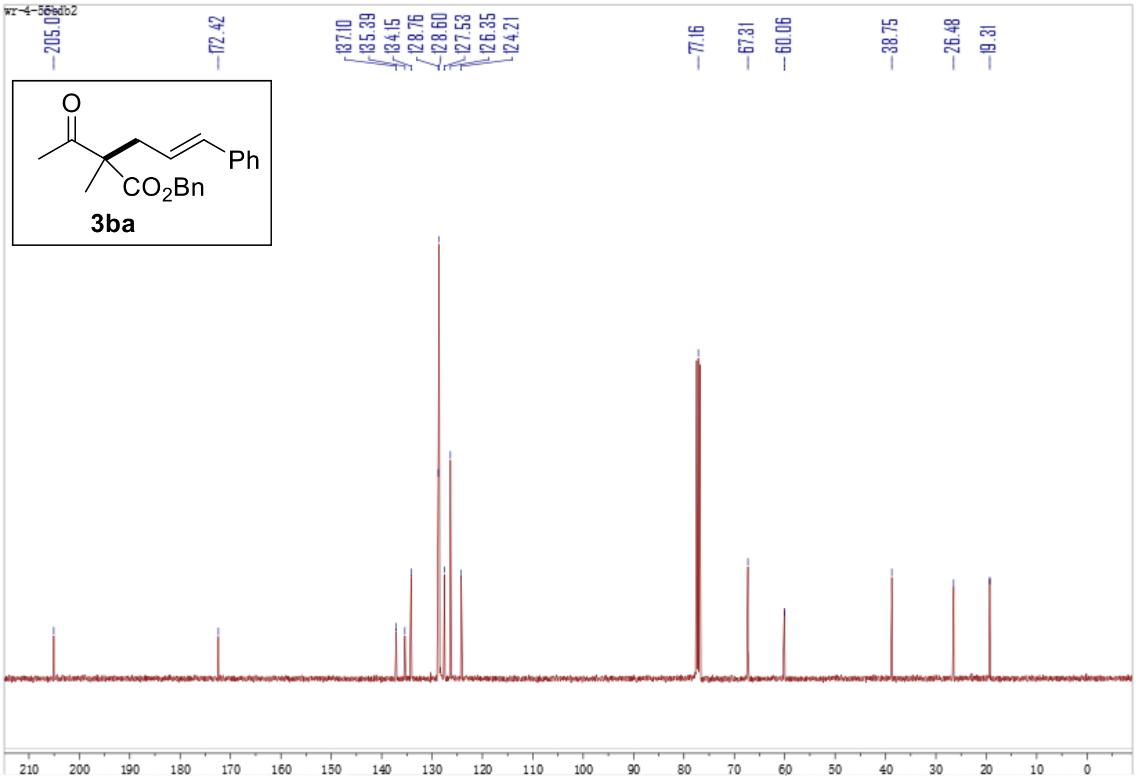

^{1}H NMR spectrum of compound **3ca** ($CDCl_3$, 400 MHz)

Links CATALOG DETAILS NMR

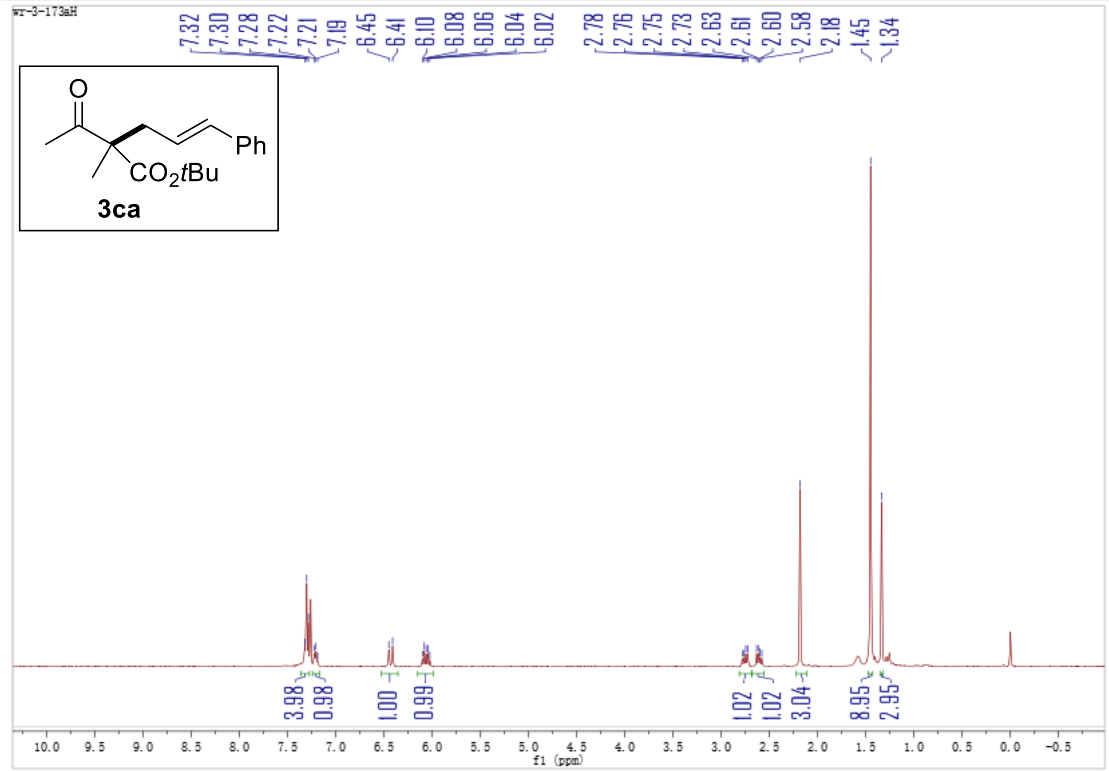

^{13}C NMR spectrum of compound **3ca** ($CDCl_3$, 101 MHz)

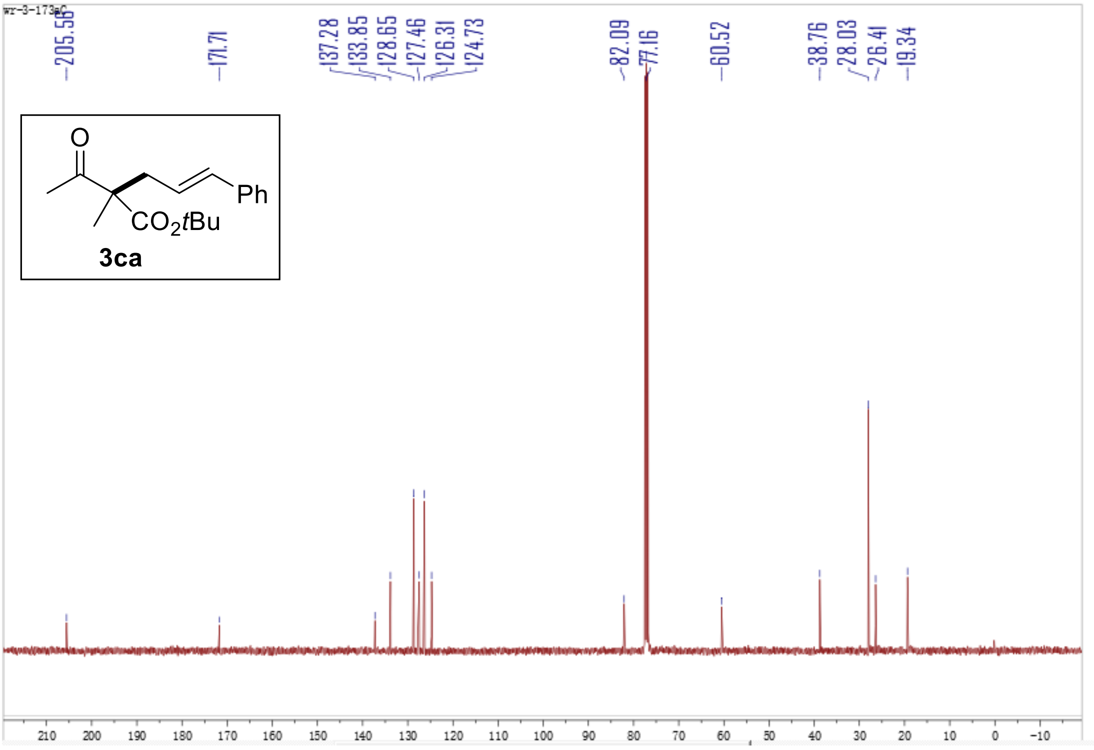

^{1}**H NMR** spectrum of compound **3da** ($CDCl_3$, 400 MHz)

Links CATALOG DETAILS NMR

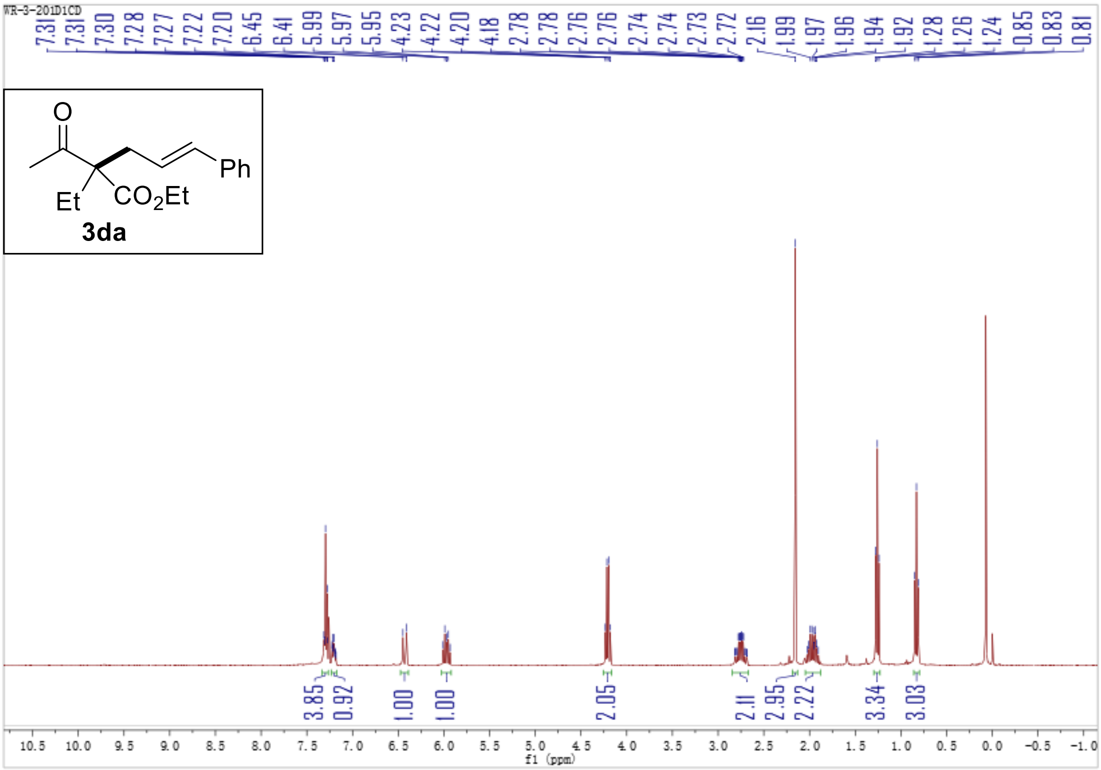

^{13}**C NMR** spectrum of compound **3da** ($CDCl_3$, 101 MHz)

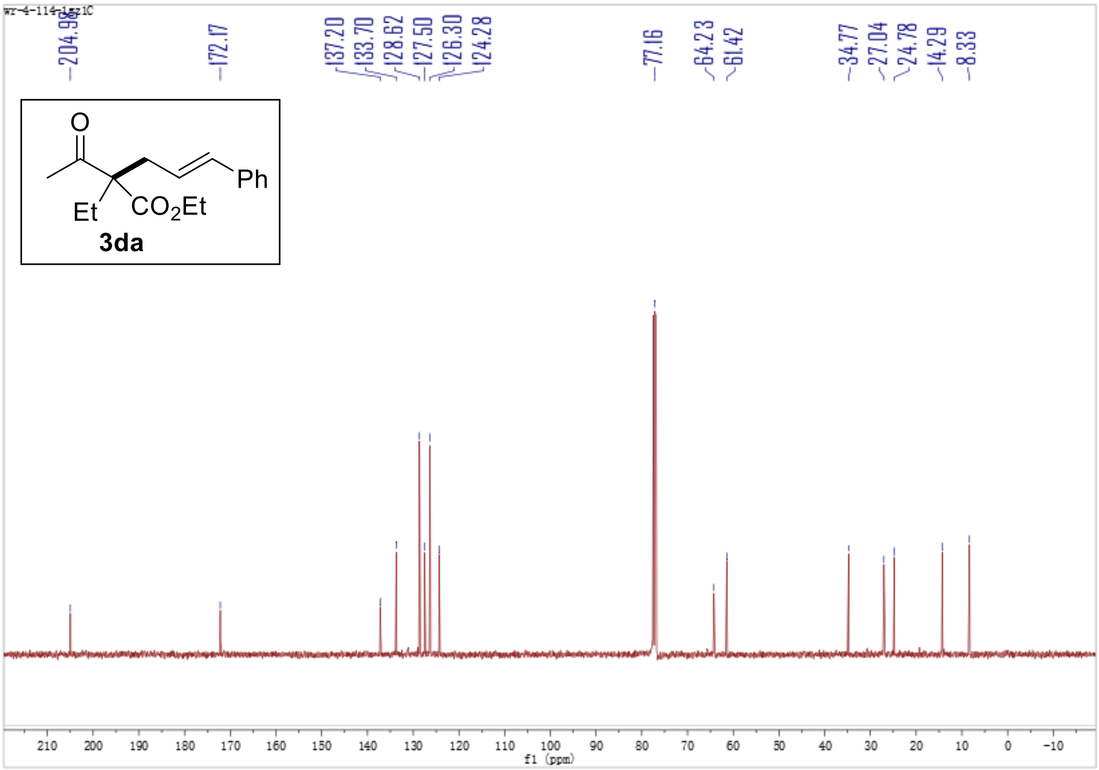

^{1}**H NMR** spectrum of compound **3ea** ($CDCl_3$, 400 MHz)

Links CATALOG DETAILS NMR

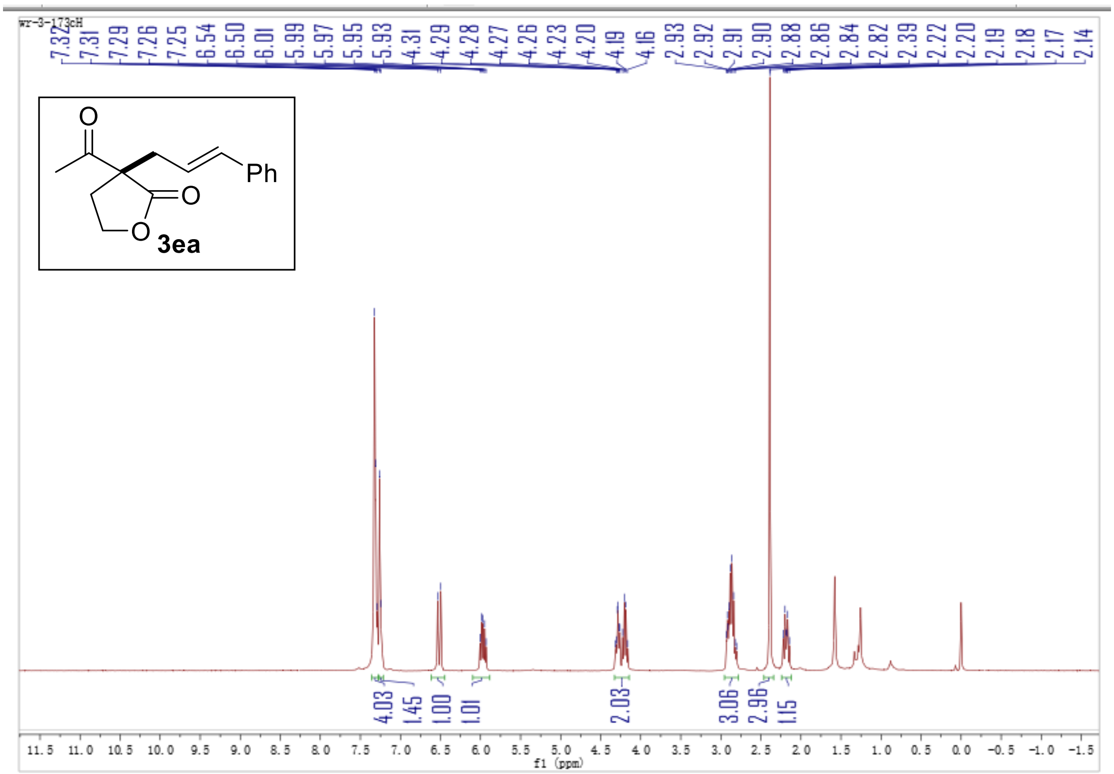

^{13}**C NMR** spectrum of compound **3ea** ($CDCl_3$, 101 MHz)

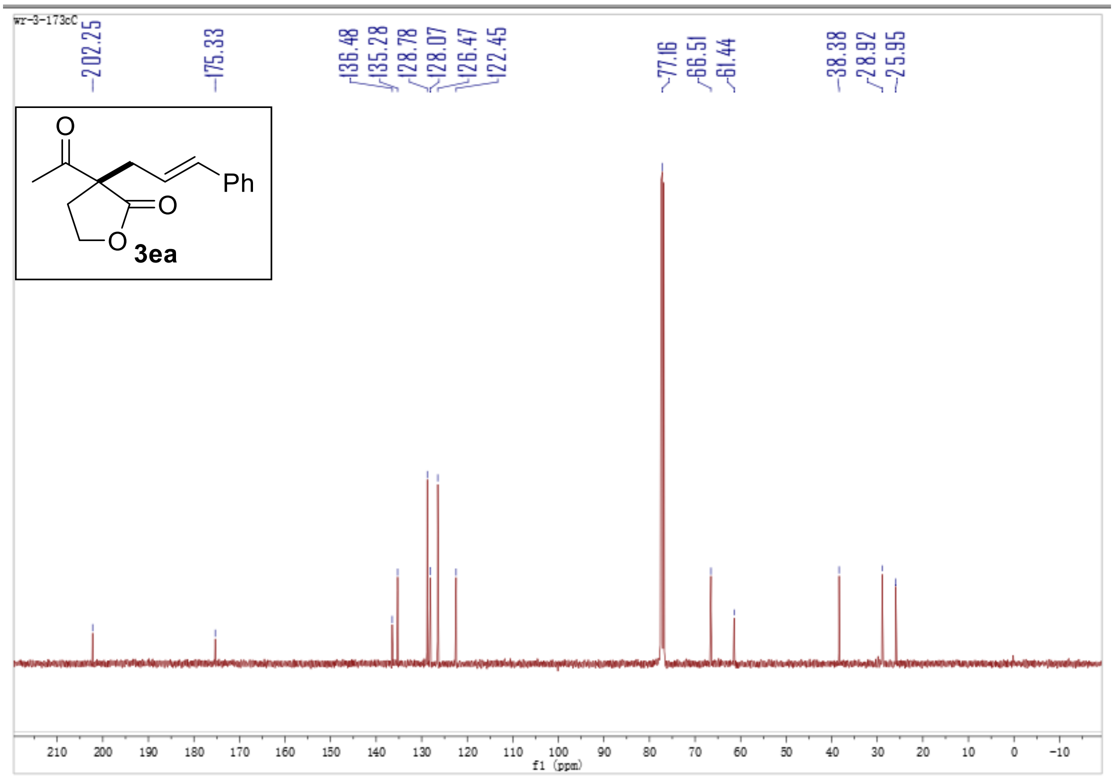

^{1}H NMR spectrum of compound **3fa** ($CDCl_3$, 400 MHz)

Links CATALOG DETAILS NMR

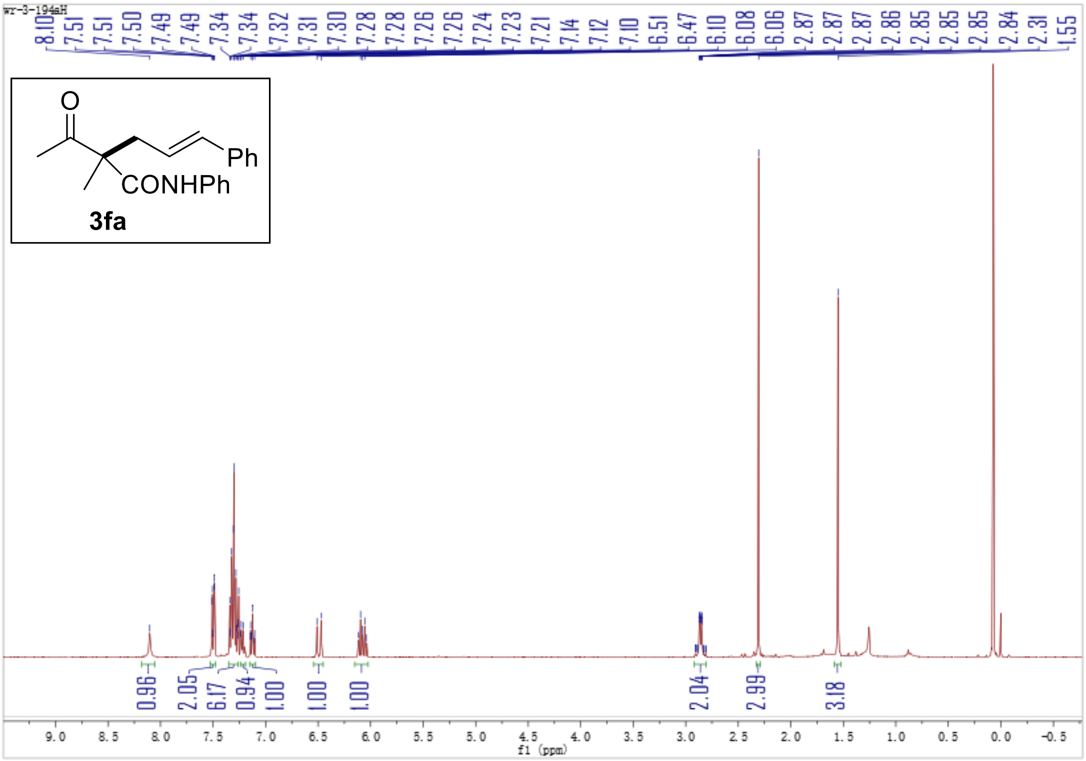

^{13}C NMR spectrum of compound **3fa** ($CDCl_3$, 101 MHz)

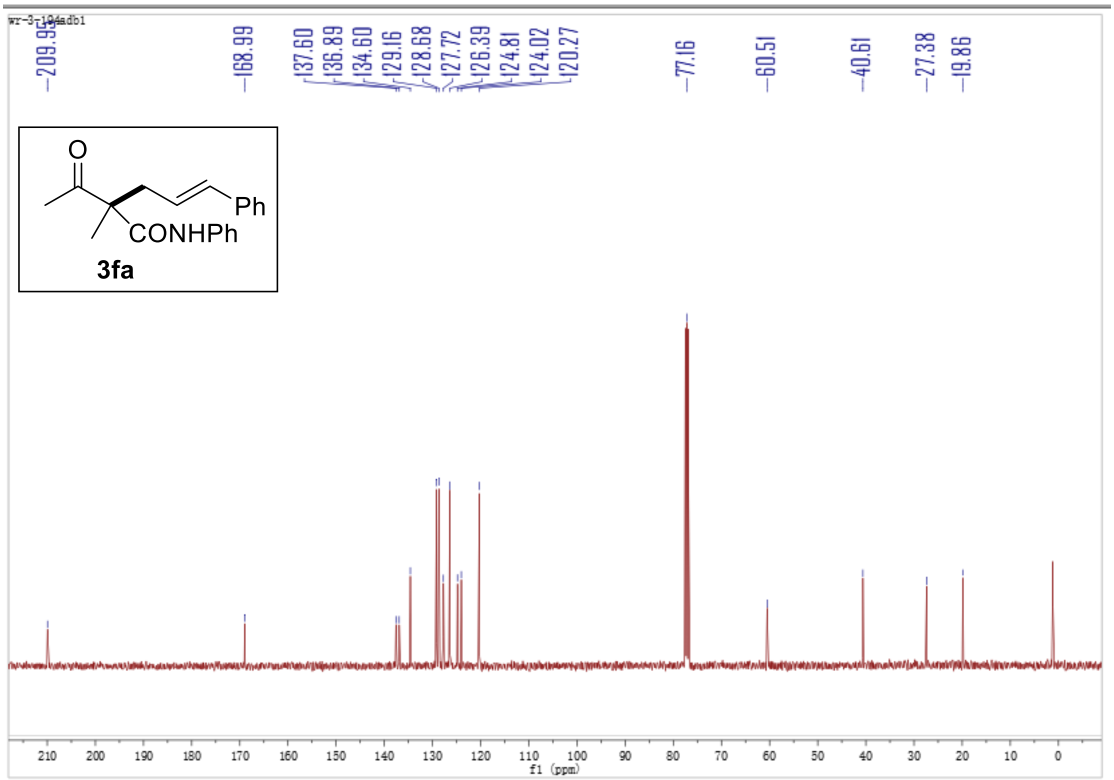

^{1}**H NMR** spectrum of compound **3ga** ($CDCl_3$, 400 MHz)

Links CATALOG DETAILS NMR

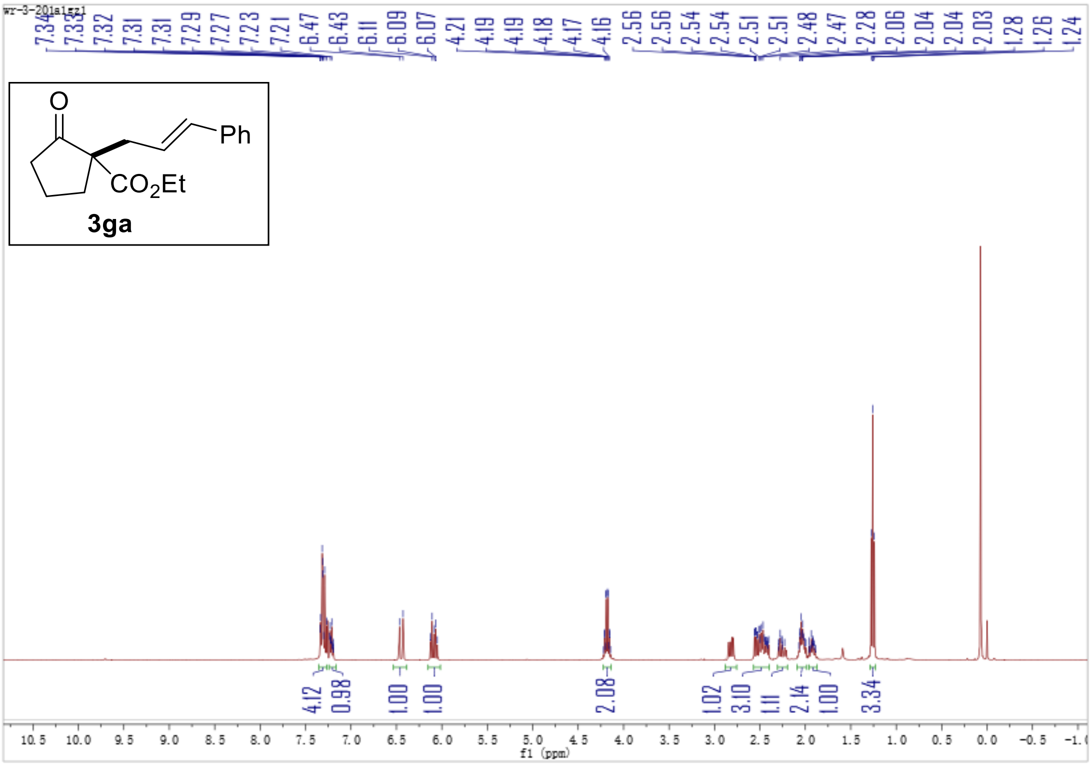

^{13}**C NMR** spectrum of compound **3ga** ($CDCl_3$, 101 MHz)

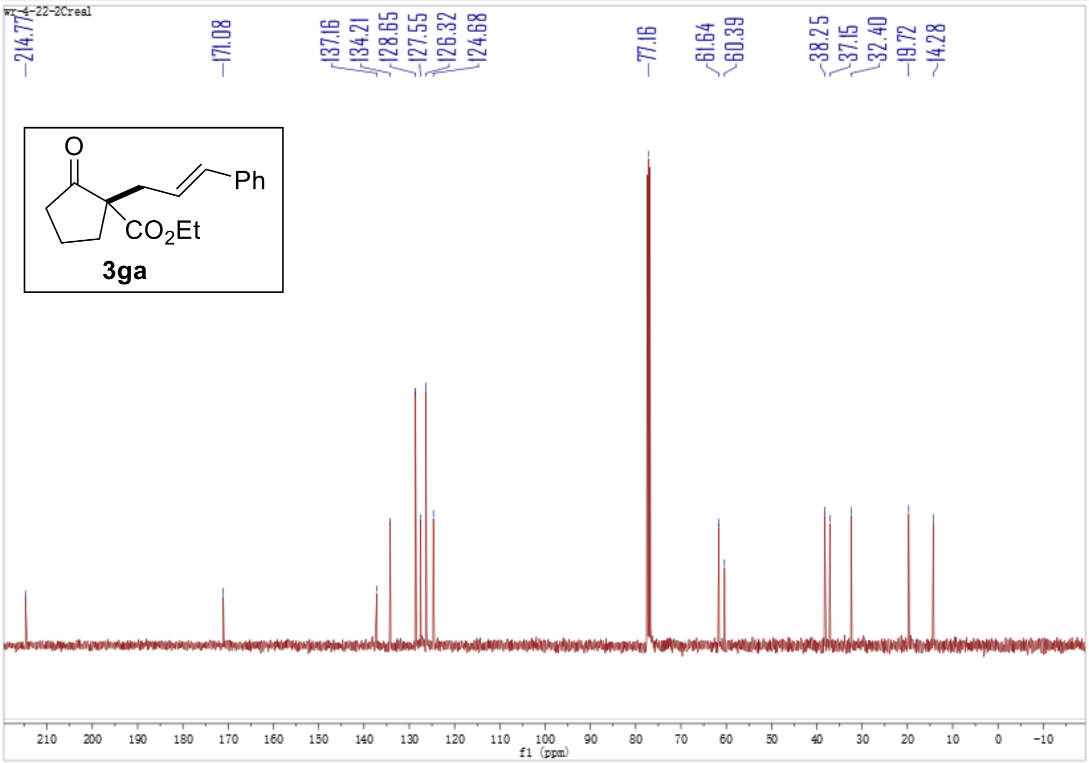

^{1}H NMR spectrum of compound **3ha** ($CDCl_3$, 400 MHz)

Links CATALOG DETAILS NMR HPLC E-Fact

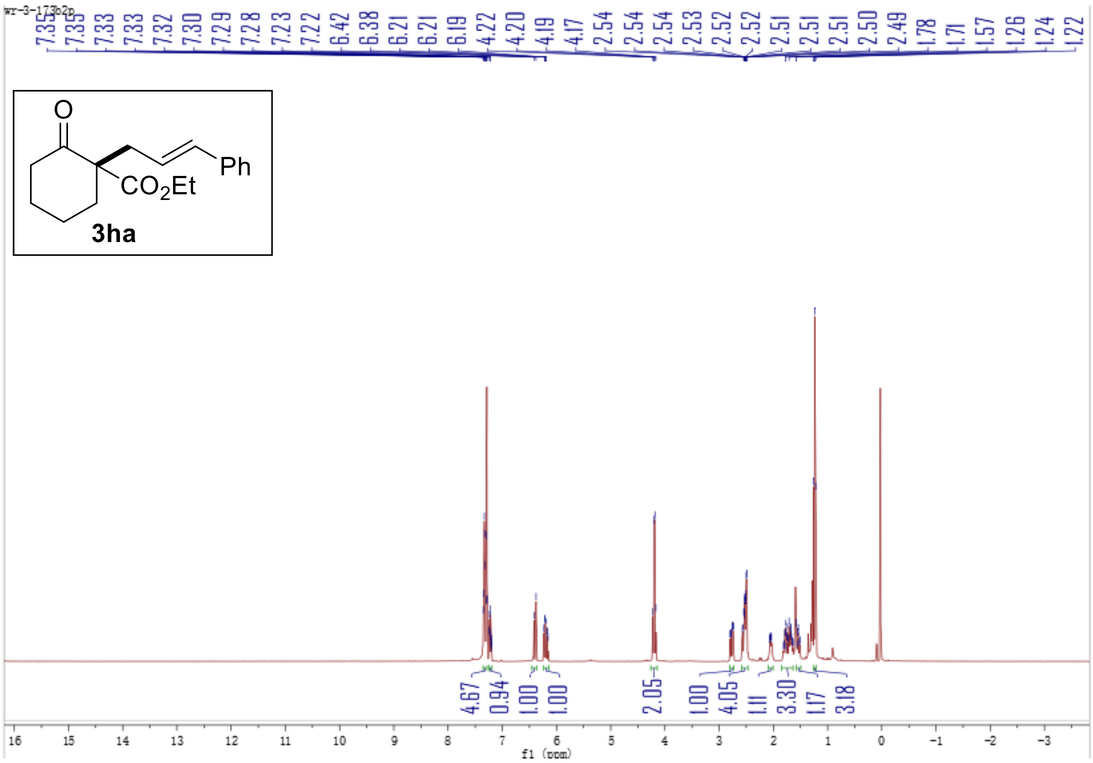

^{13}C NMR spectrum of compound **3ha** ($CDCl_3$, 101 MHz)

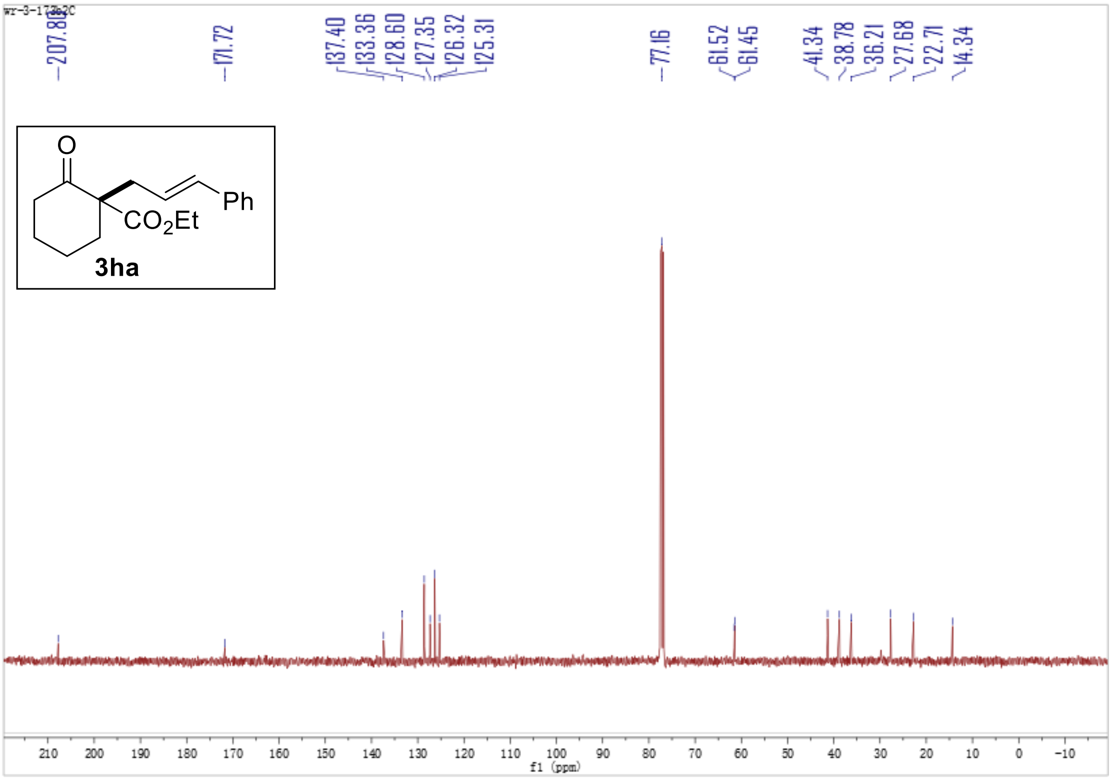

^{1}H **NMR** spectrum of compound **3ha-*Z*** ($CDCl_3$, 400 MHz)

Links CATALOG DETAILS NMR HPLC

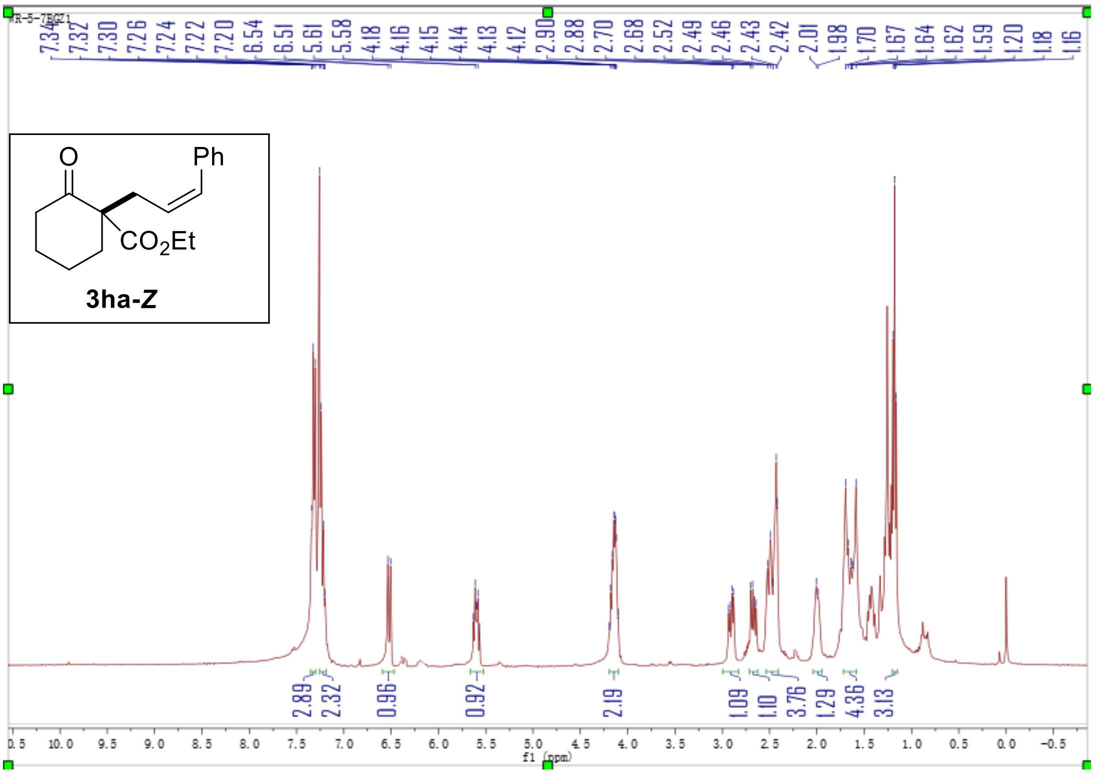

^{13}C **NMR** spectrum of compound **3ha-*Z*** ($CDCl_3$, 101 MHz)

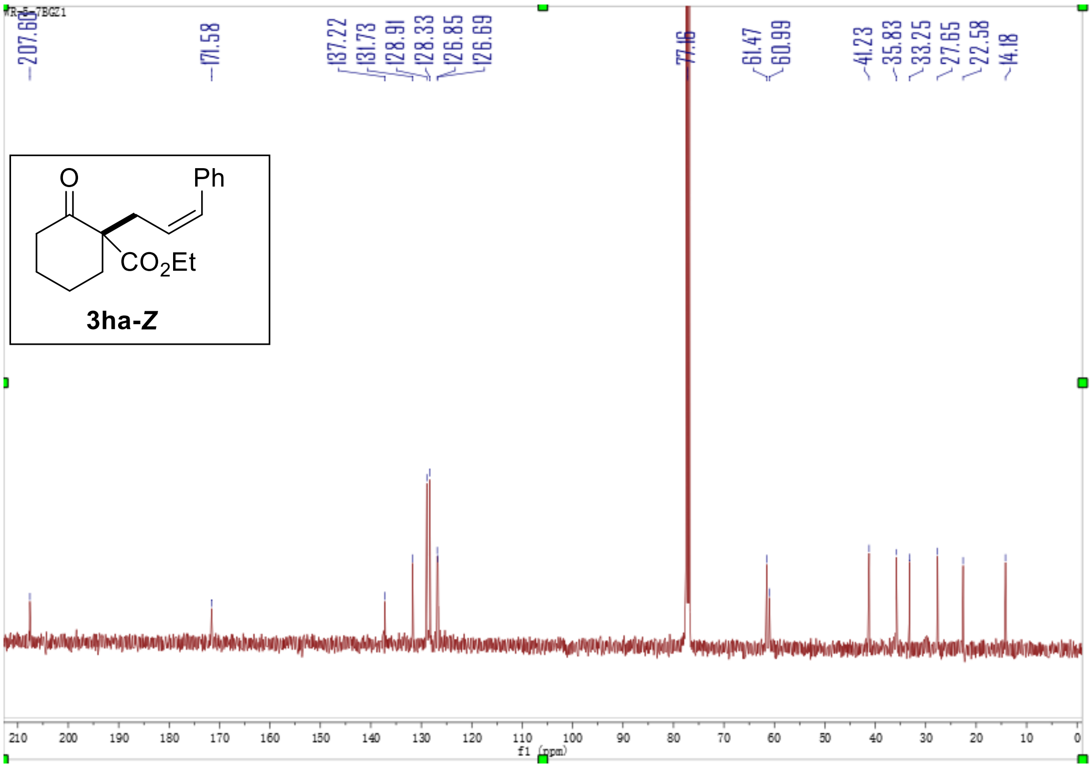

NOE spectrum of compound **3ha-*Z*** ($CDCl_3$, 400 MHz)

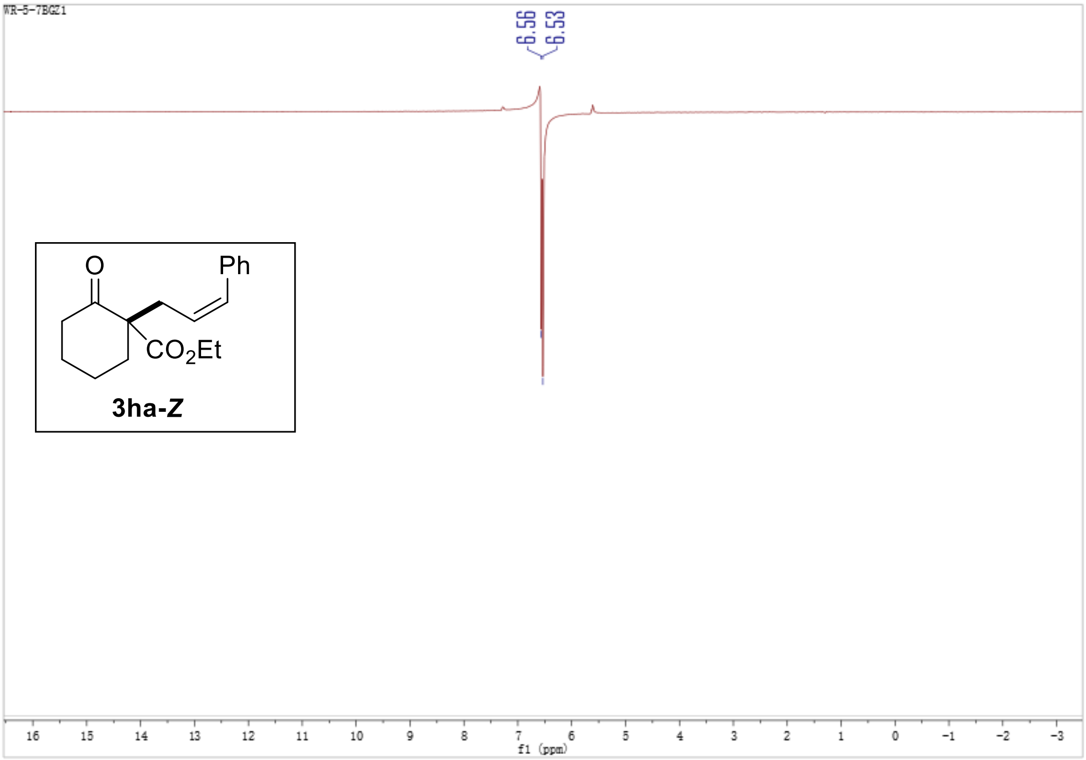

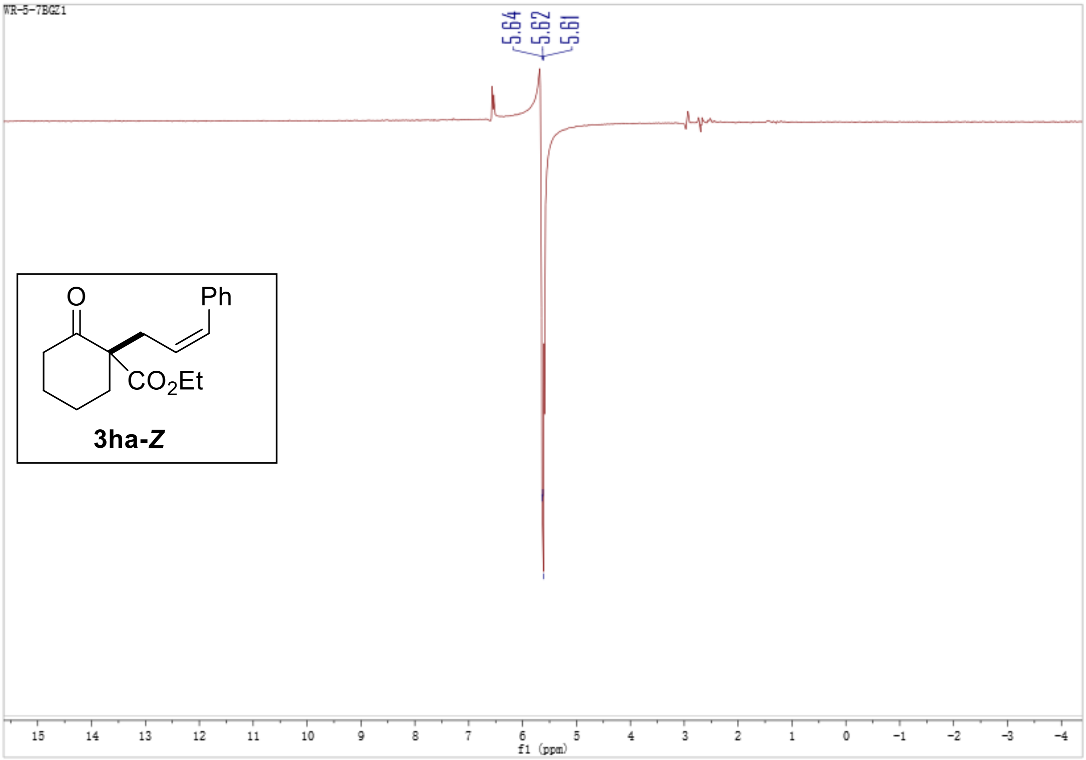

^{1}H-^{1}H COSY spectrum of compound **3ha-*Z*** ($CDCl_3$, 400 MHz)

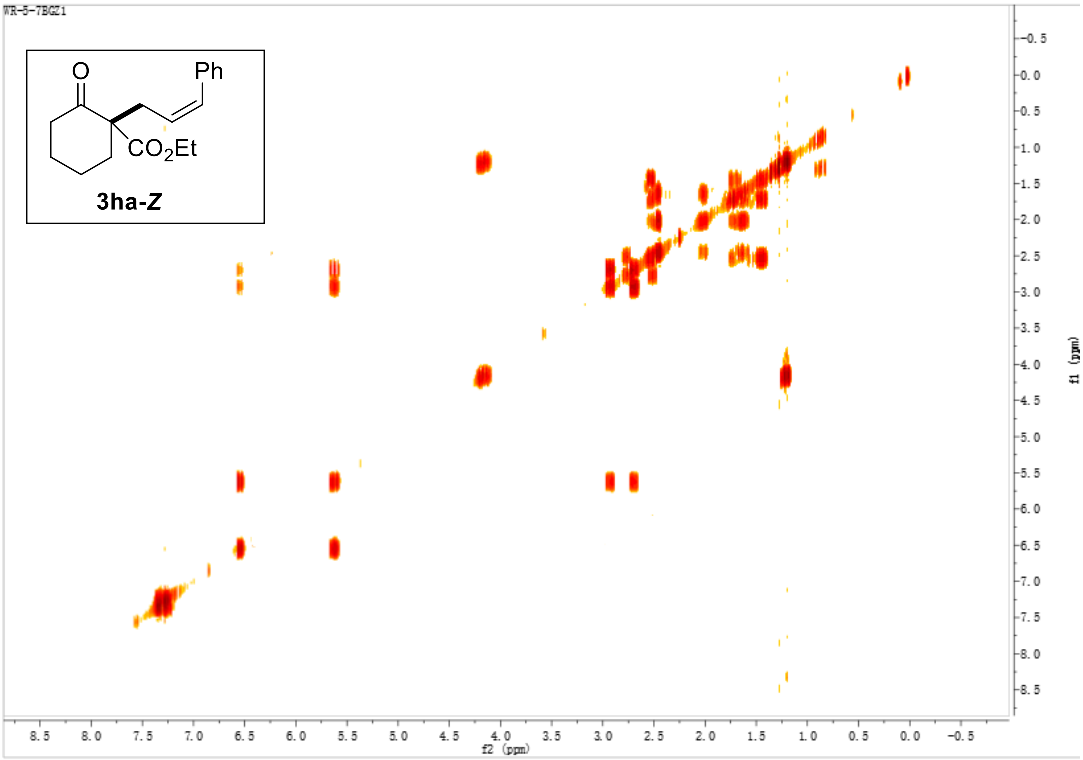

^{1}**H NMR** spectrum of compound **3ia** ($CDCl_3$, 400 MHz)

Links CATALOG DETAILS NMR

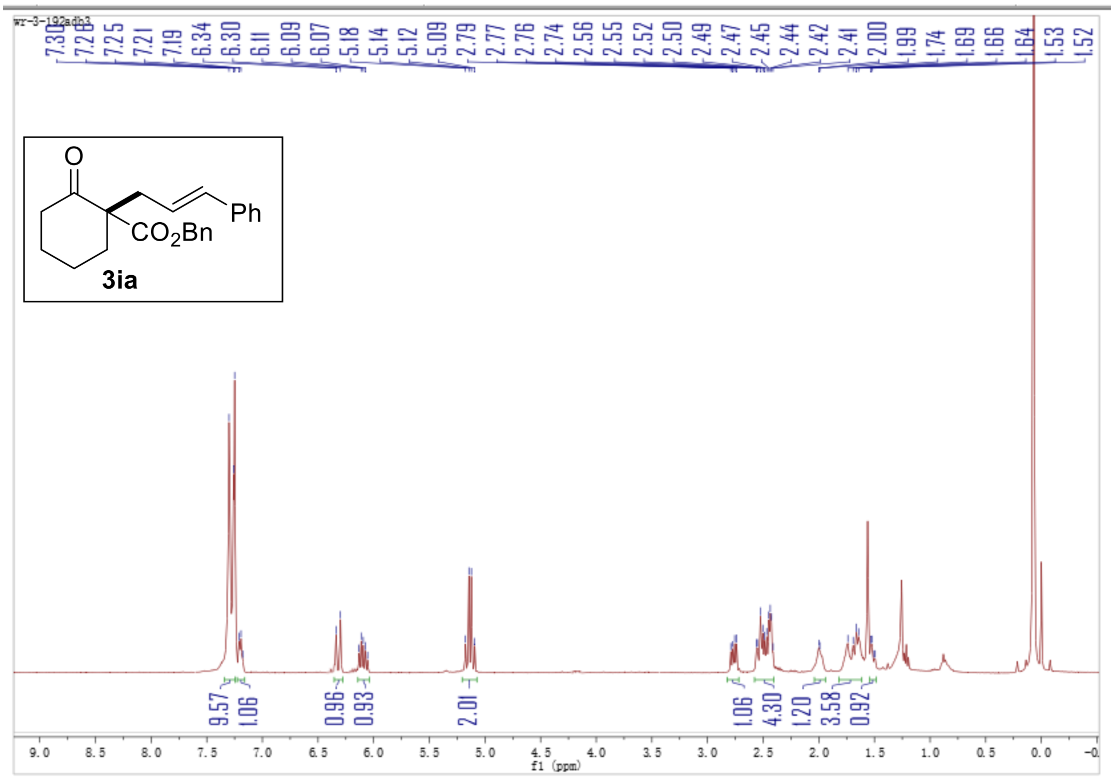

^{13}**C NMR** spectrum of compound **3ia** ($CDCl_3$, 101 MHz)

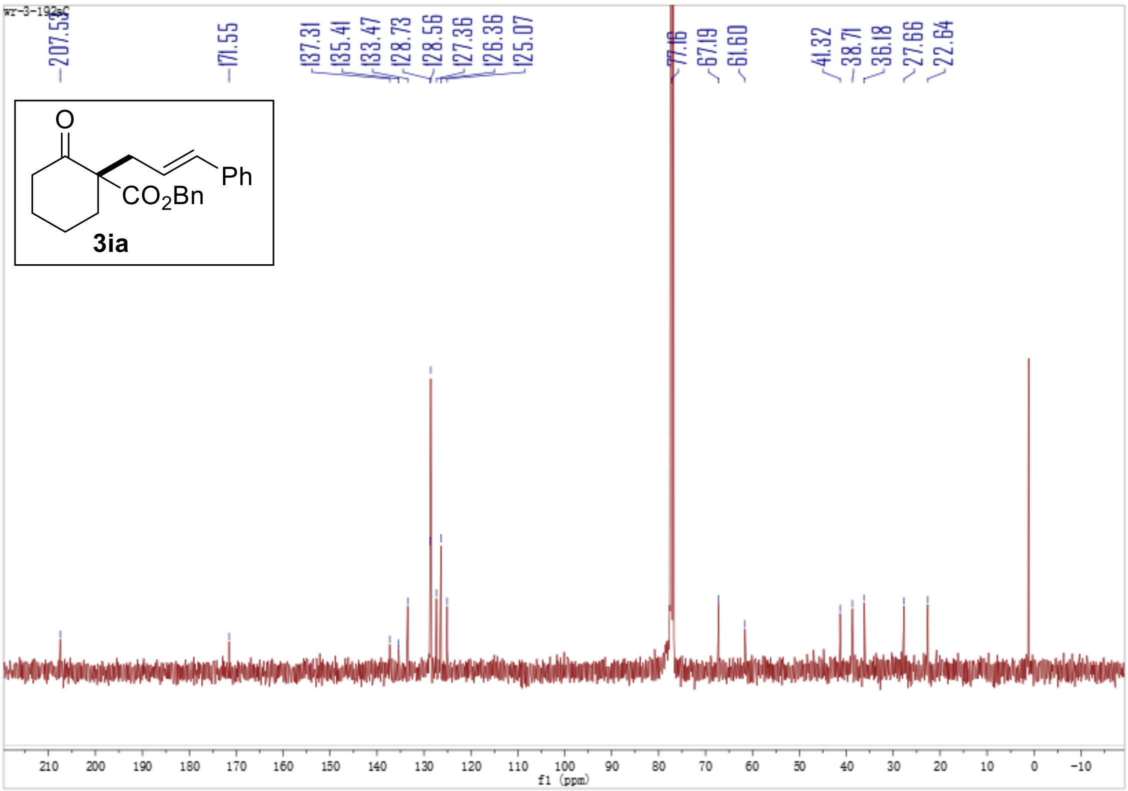

^{1}H NMR spectrum of compound **3ja** ($CDCl_3$, 400 MHz)

Links CATALOG DETAILS NMR

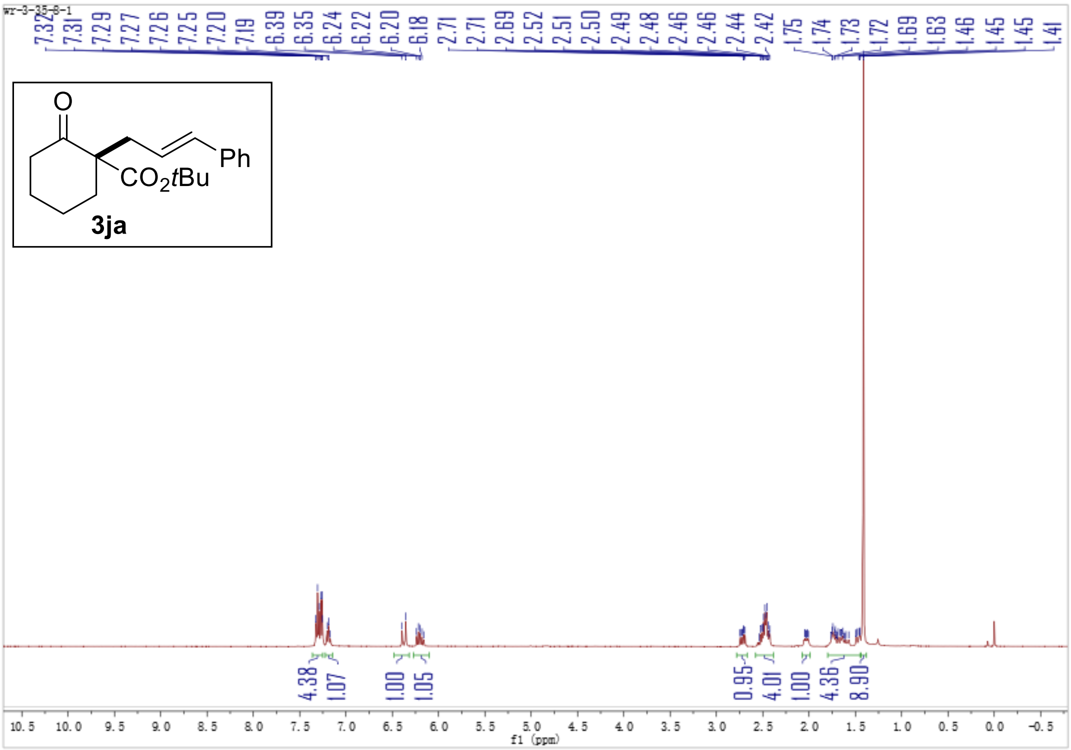

^{13}C NMR spectrum of compound **3ja** ($CDCl_3$, 101 MHz)

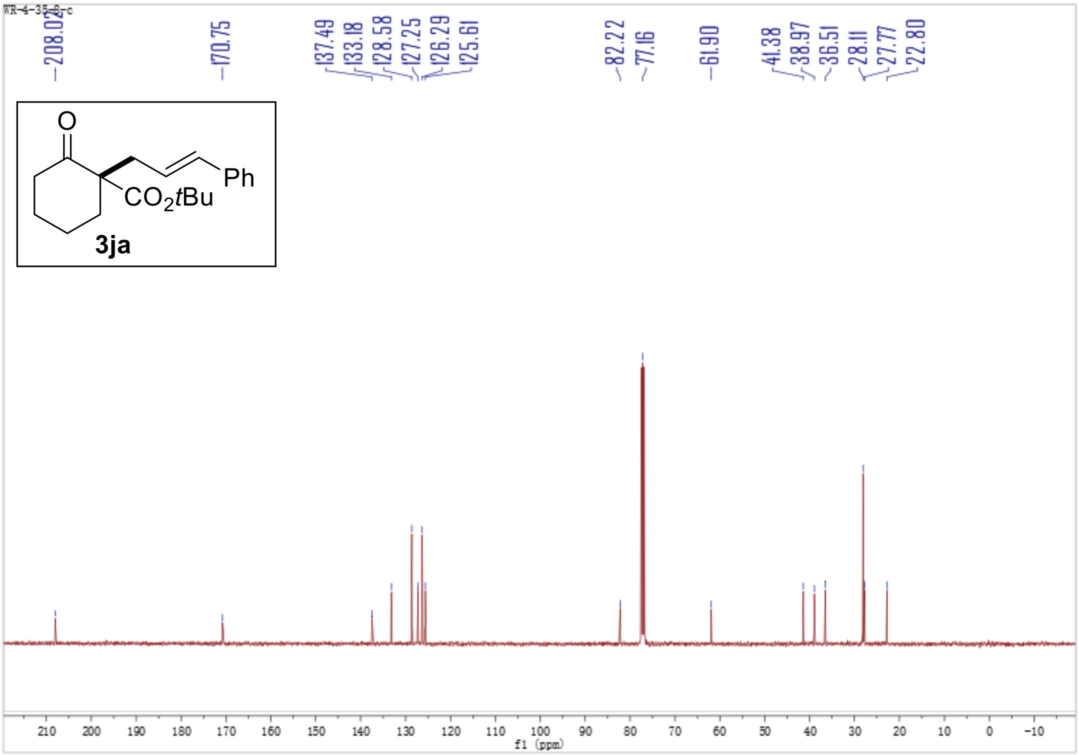

[1]**H NMR** spectrum of compound **3ka** ($CDCl_3$, 400 MHz)

Links CATALOG DETAILS NMR

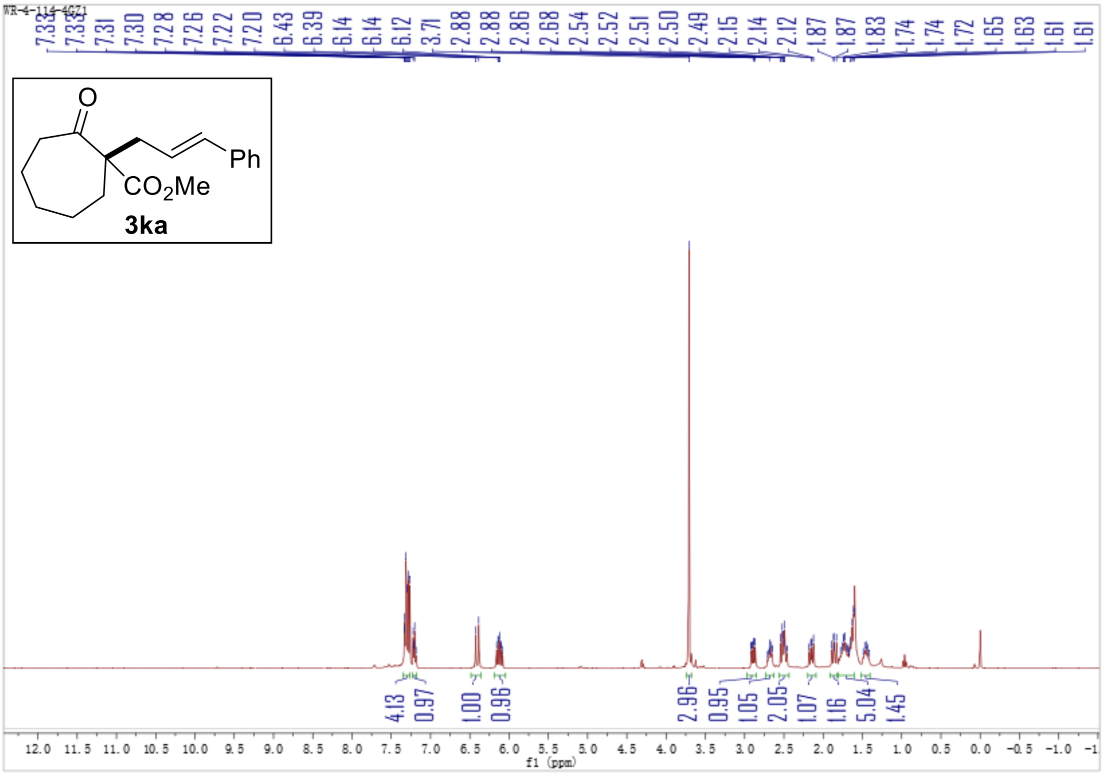

[13]**C NMR** spectrum of compound **3ka** ($CDCl_3$, 101 MHz)

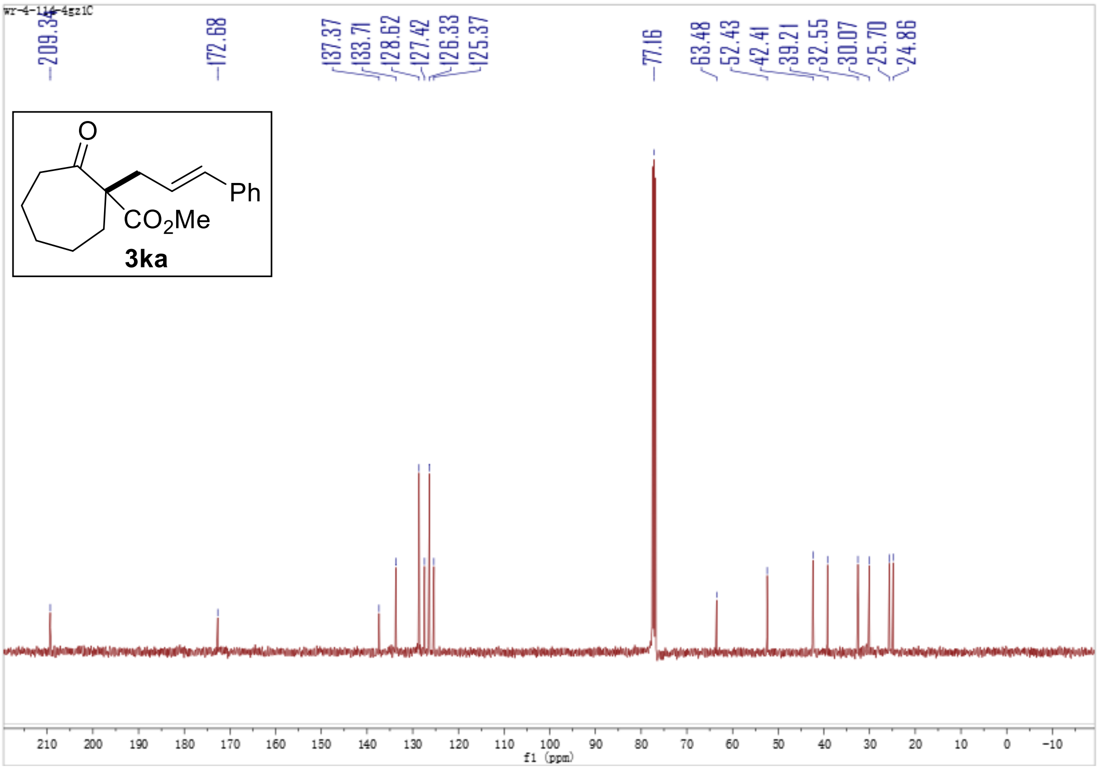

^{1}H NMR spectrum of compound 3la ($CDCl_3$, 400 MHz)

Links CATALOG DETAILS NMR

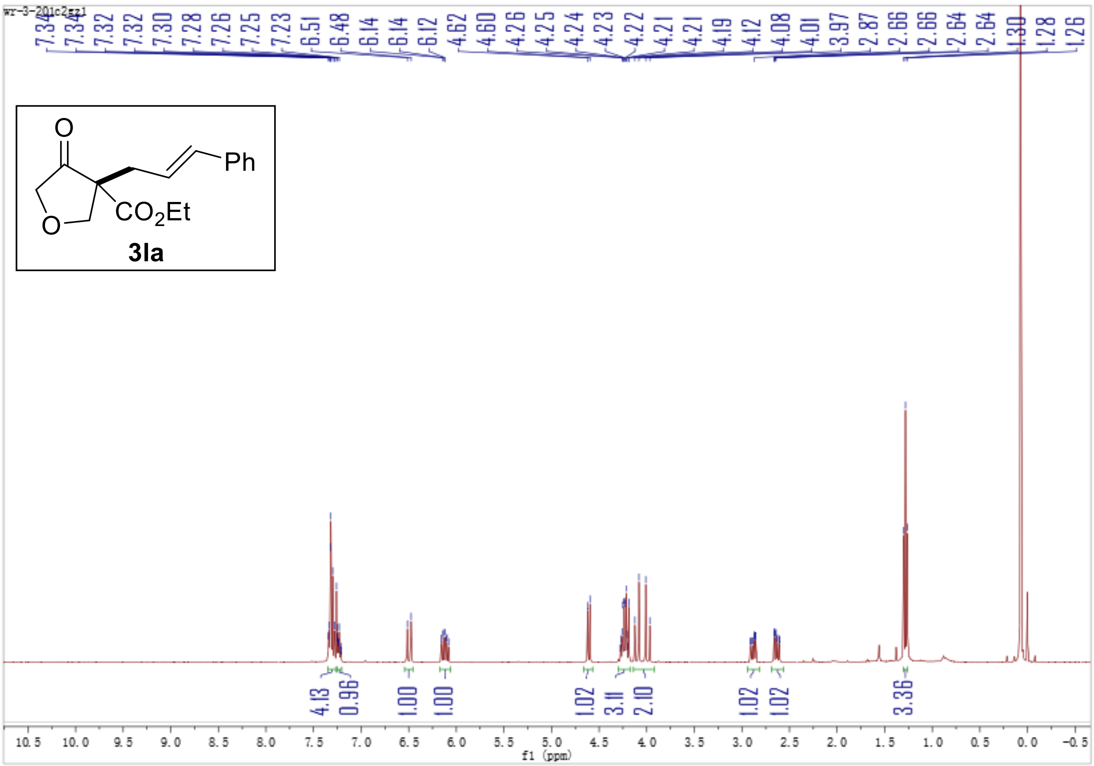

^{13}C NMR spectrum of compound 3la ($CDCl_3$, 101 MHz)

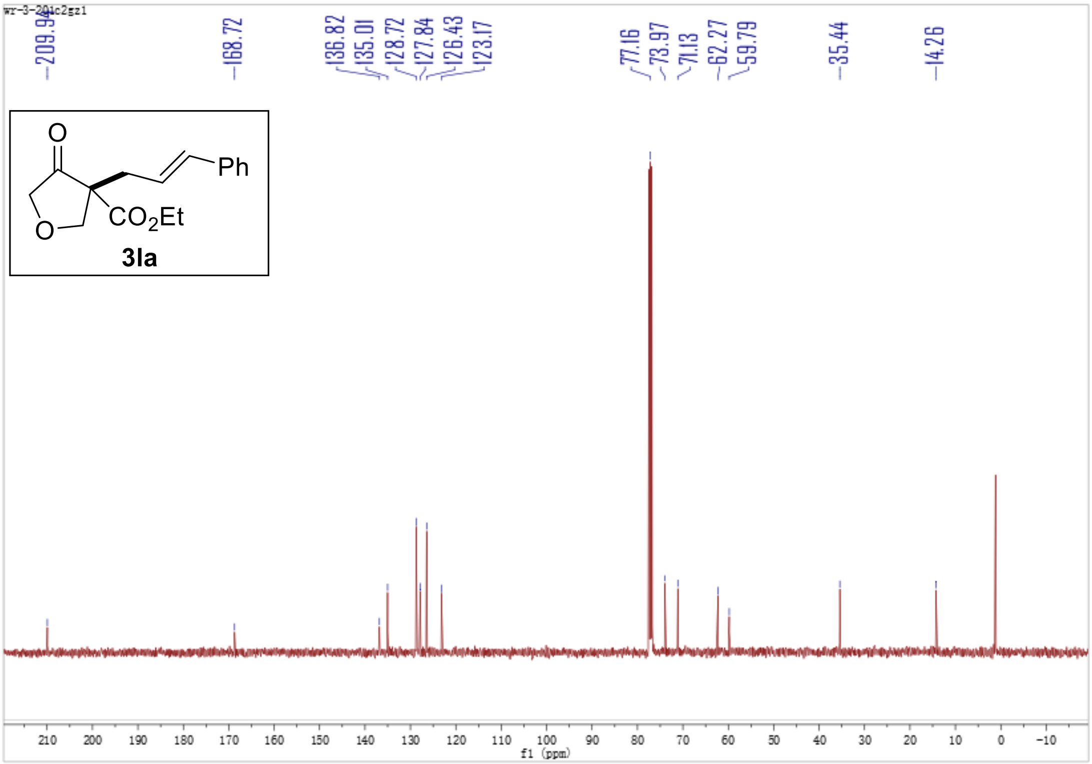

^{1}H NMR spectrum of compound 3ma ($CDCl_3$, 400 MHz)

Links CATALOG DETAILS NMR

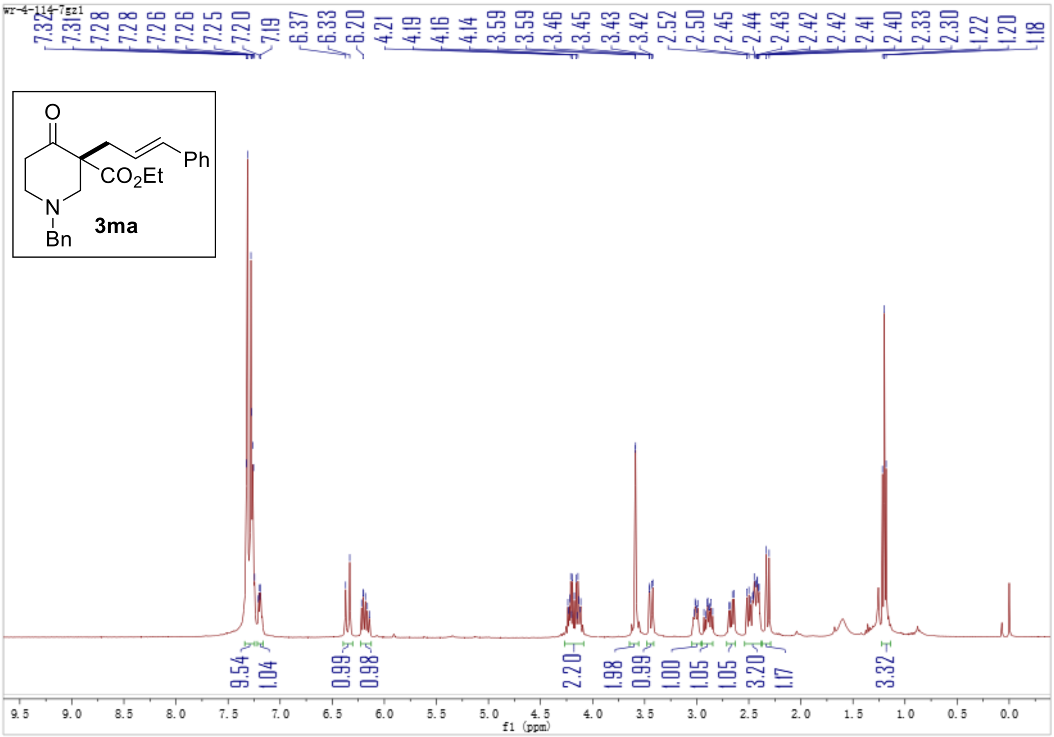

^{13}C NMR spectrum of compound 3ma ($CDCl_3$, 101 MHz)

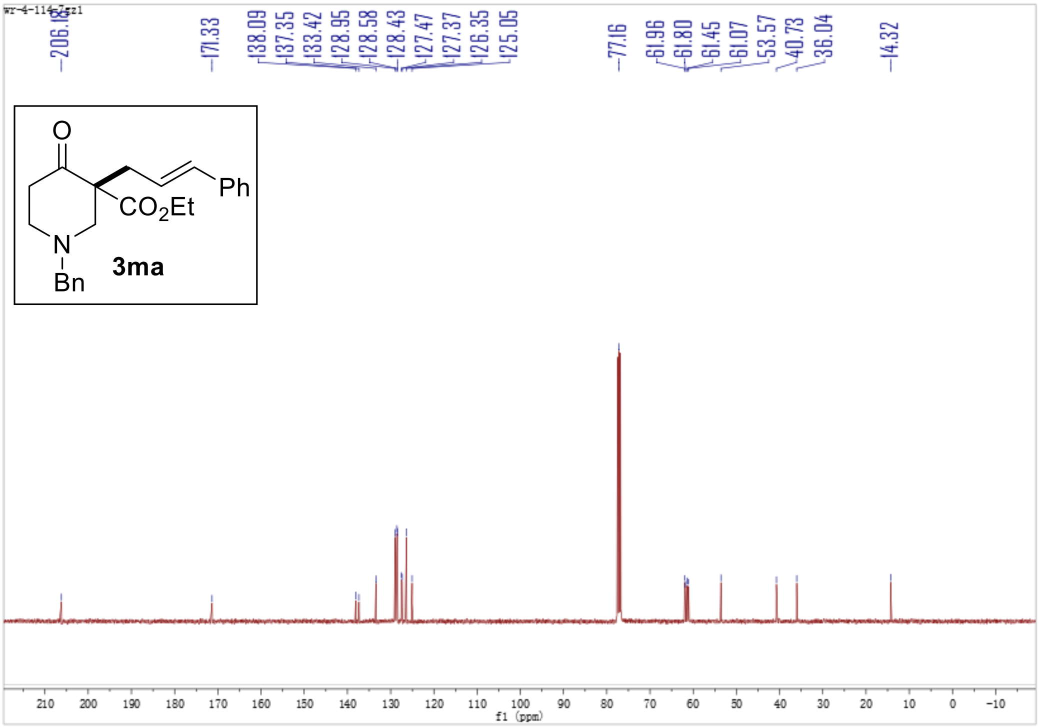

^{1}**H NMR** spectrum of compound **3na** ($CDCl_3$, 400 MHz)

Links CATALOG DETAILS NMR

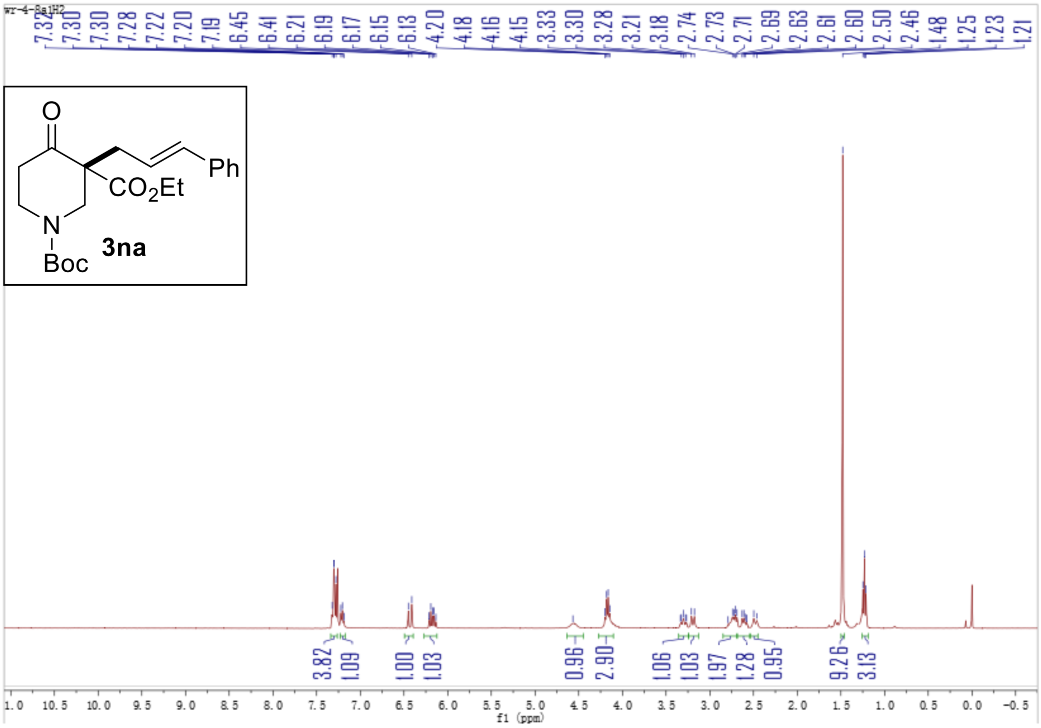

^{13}**C NMR** spectrum of compound **3na** ($CDCl_3$, 101 MHz)

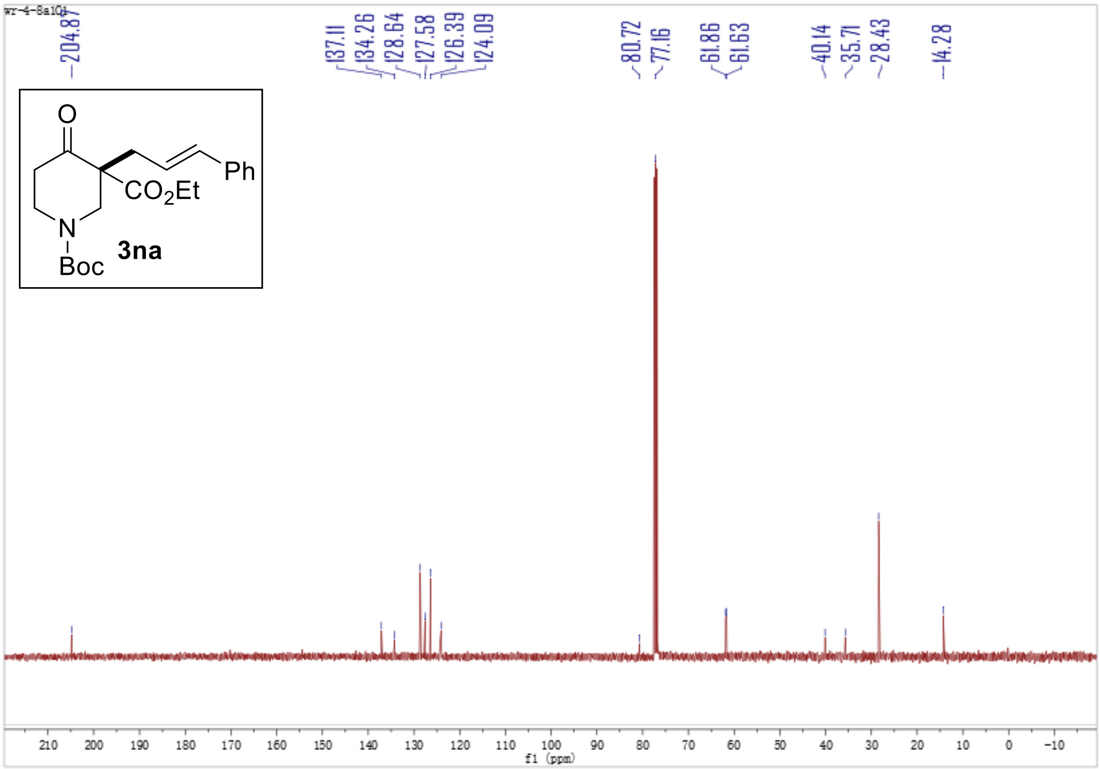

[1]**H NMR** spectrum of compound **3oa** ($CDCl_3$, 400 MHz)

Links CATALOG DETAILS NMR

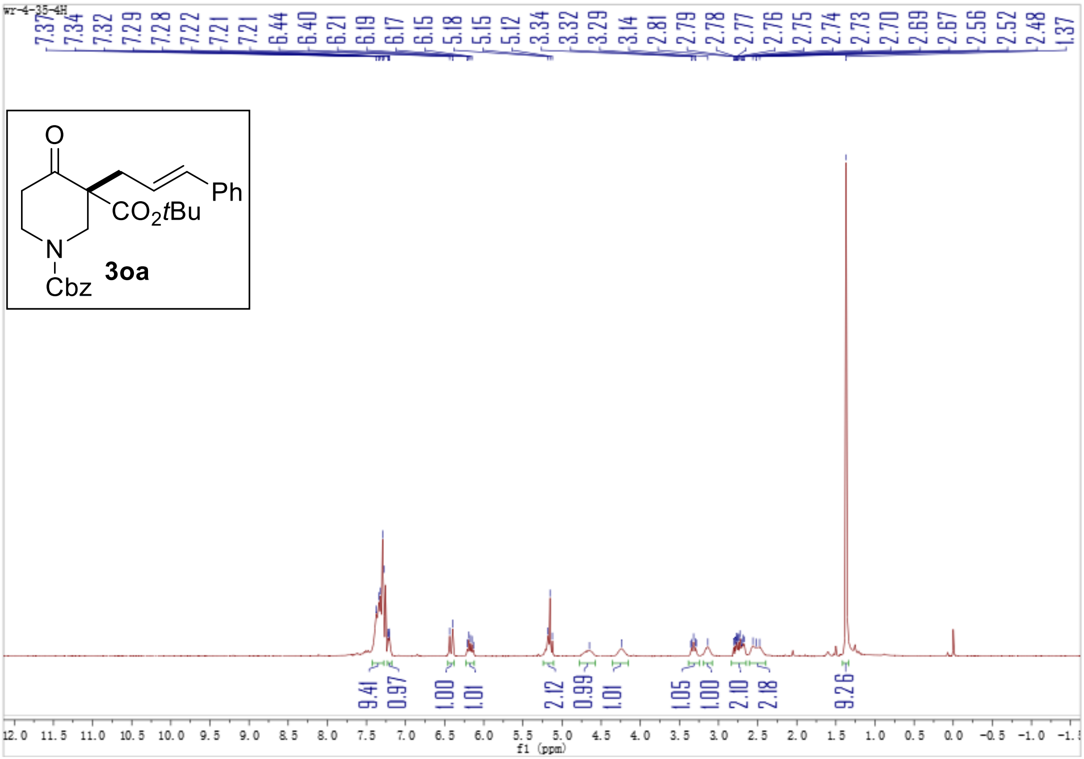

[13]**C NMR** spectrum of compound **3oa** ($CDCl_3$, 101 MHz)

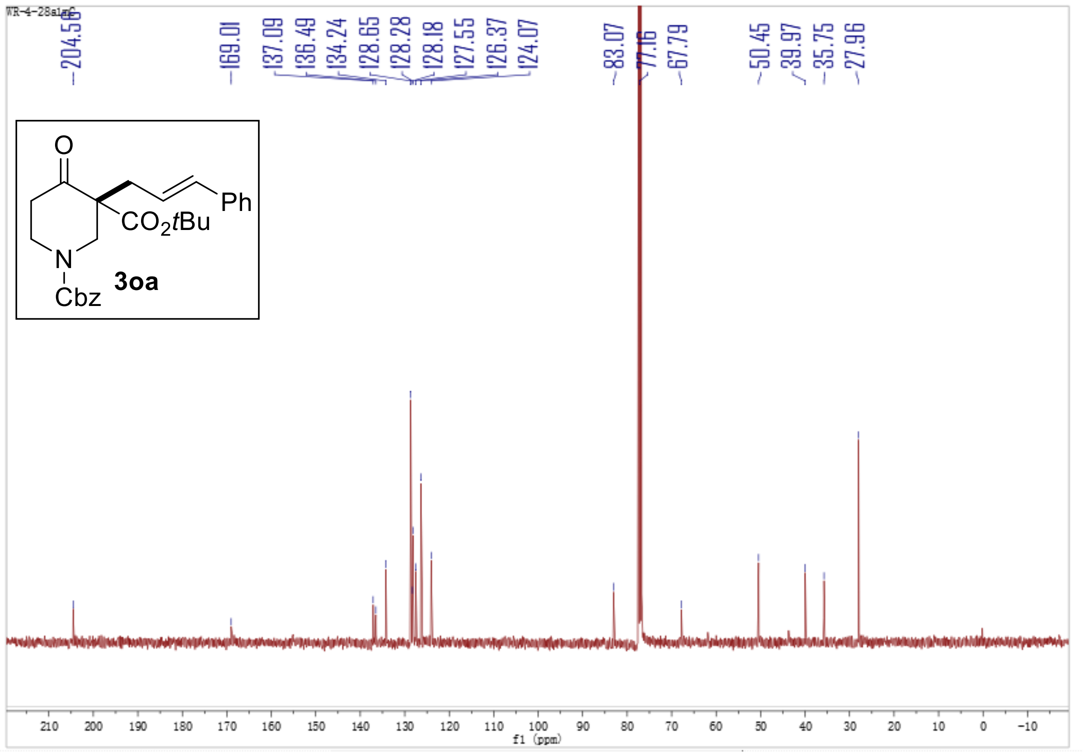

^{1}H NMR spectrum of compound 3pa ($CDCl_3$, 400 MHz)

Links CATALOG DETAILS NMR

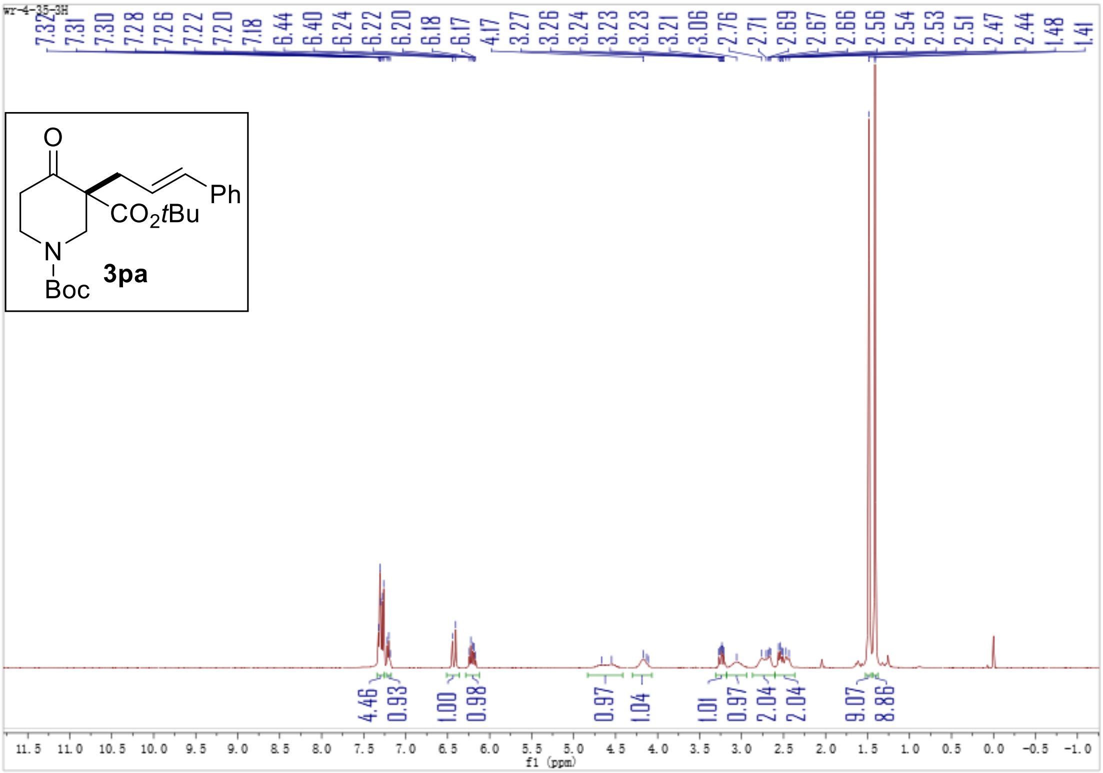

^{13}C NMR spectrum of compound 3pa ($CDCl_3$, 101 MHz)

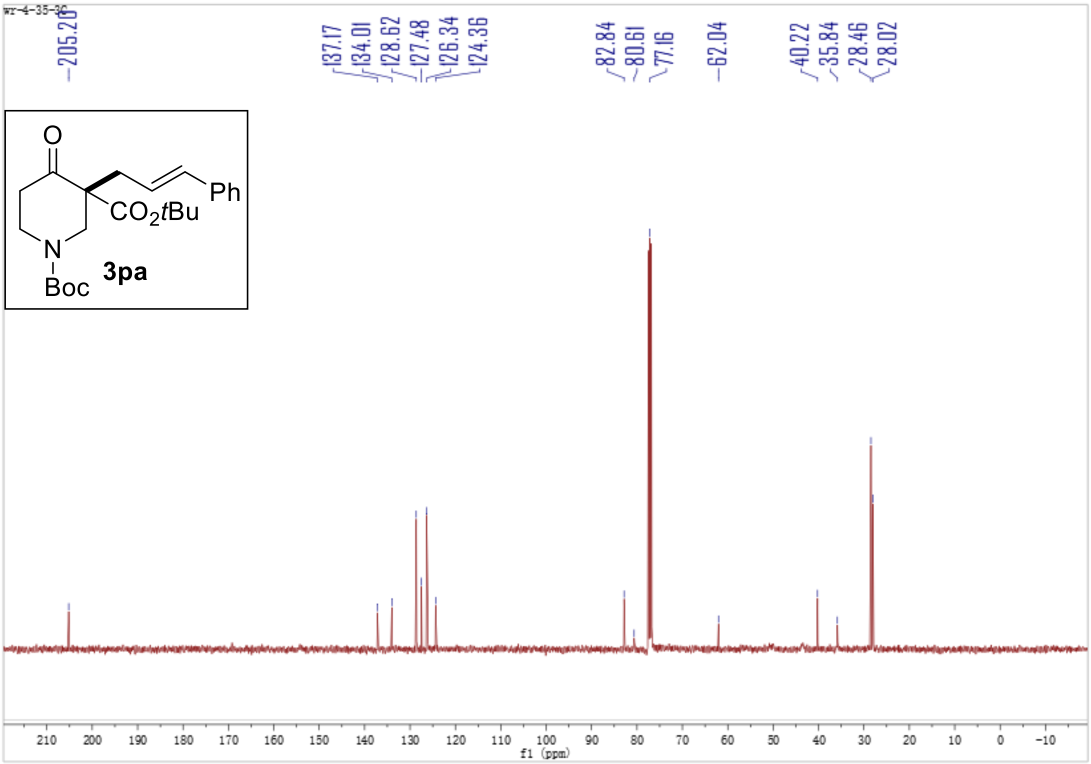

^{1}H NMR spectrum of compound **3qa** ($CDCl_3$, 400 MHz)

Links CATALOG DETAILS NMR

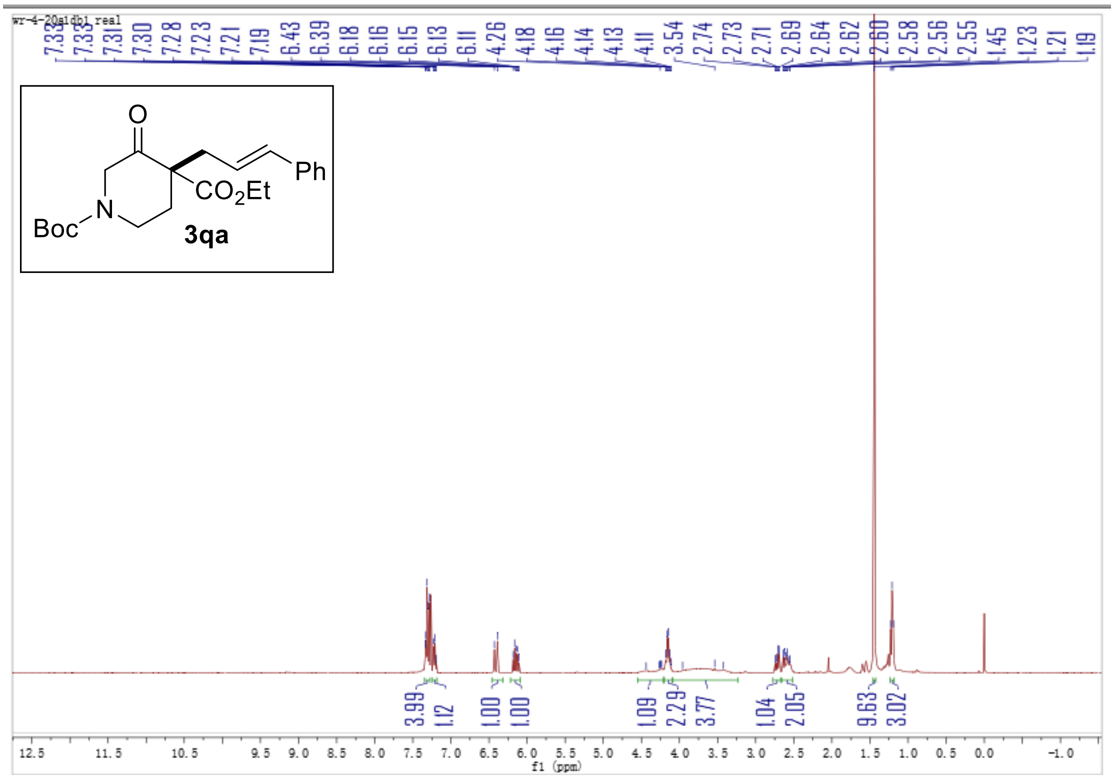

^{13}C NMR spectrum of compound **3pa** ($CDCl_3$, 101 MHz)

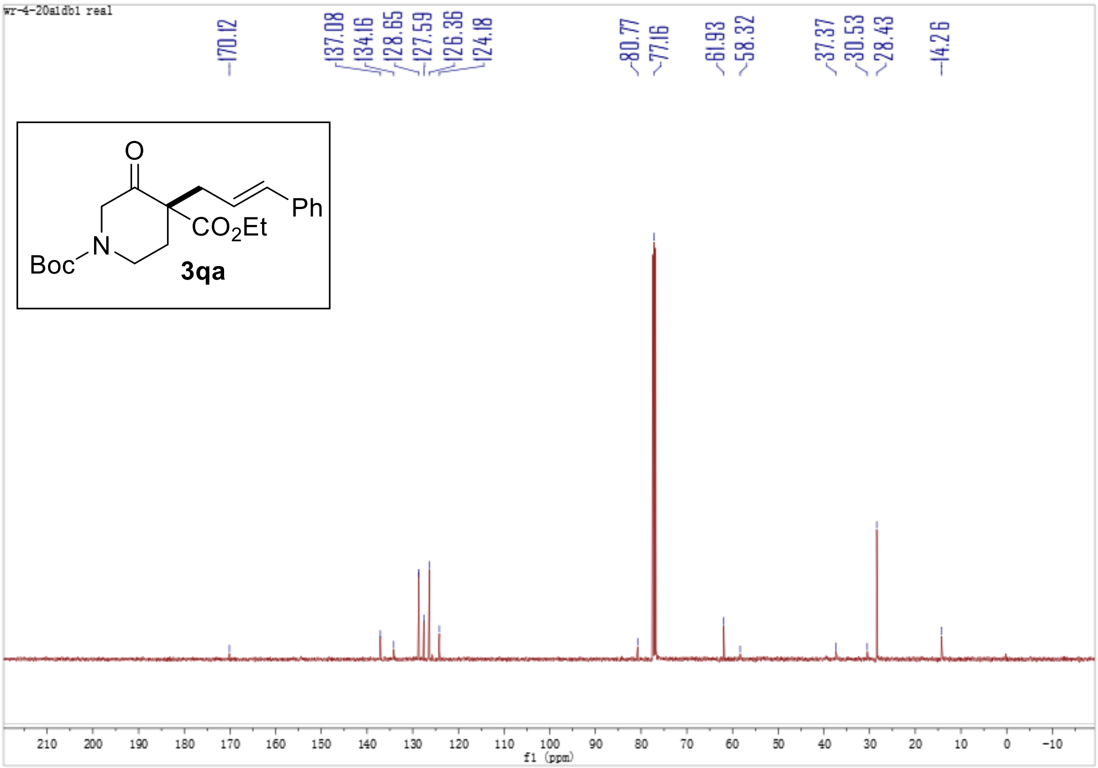

^{1}**H NMR** spectrum of compound **3ra** ($CDCl_3$, 400 MHz)

Links CATALOG DETAILS NMR

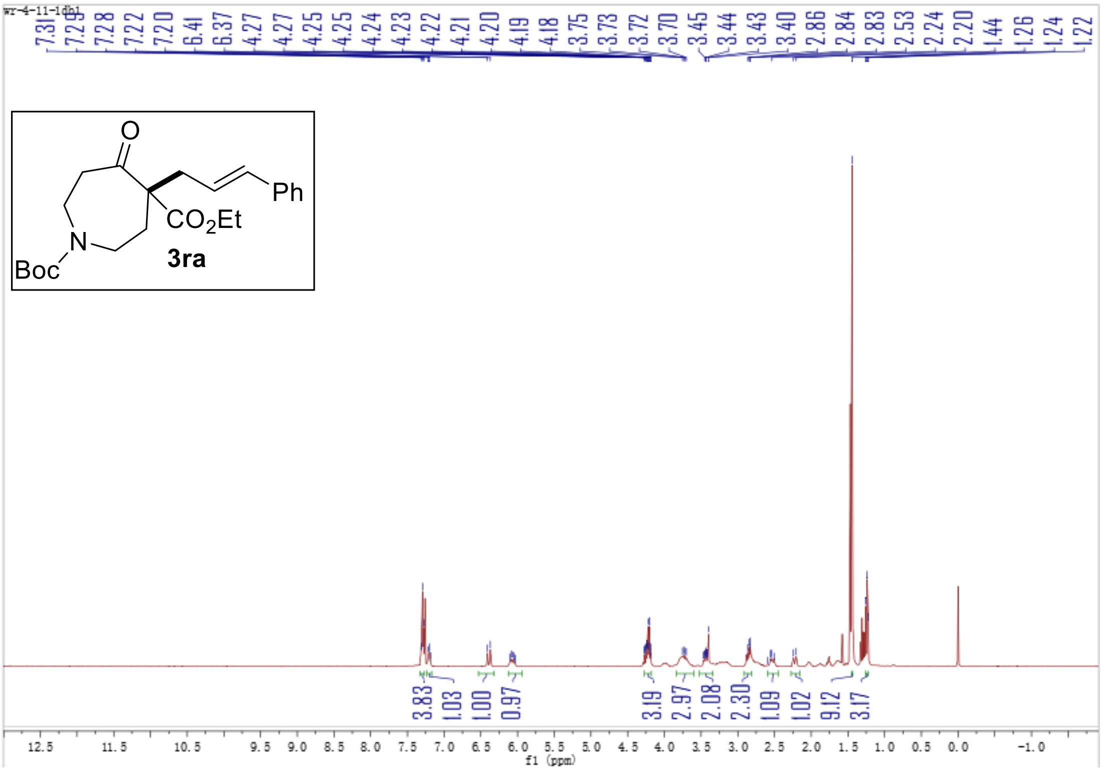

^{13}**C NMR** spectrum of compound **3ra** ($CDCl_3$, 101 MHz)

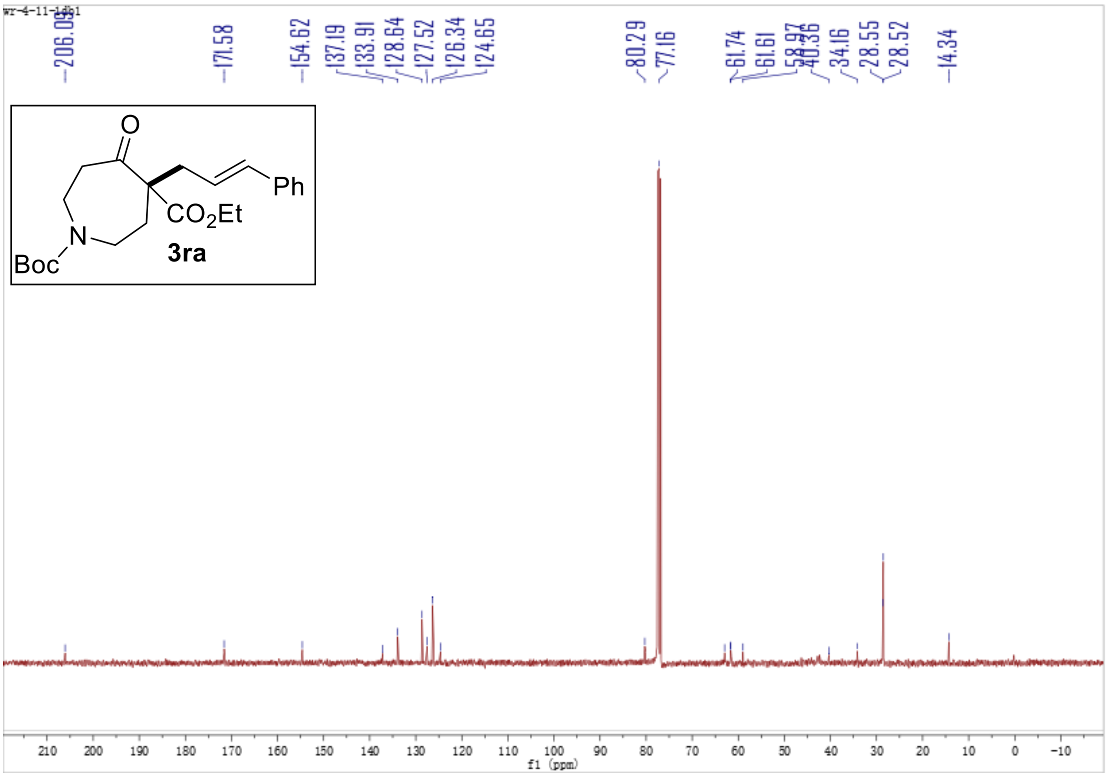

^{1}**H NMR** spectrum of compound **3sa** ($CDCl_3$, 400 MHz)

Links CATALOG DETAILS NMR

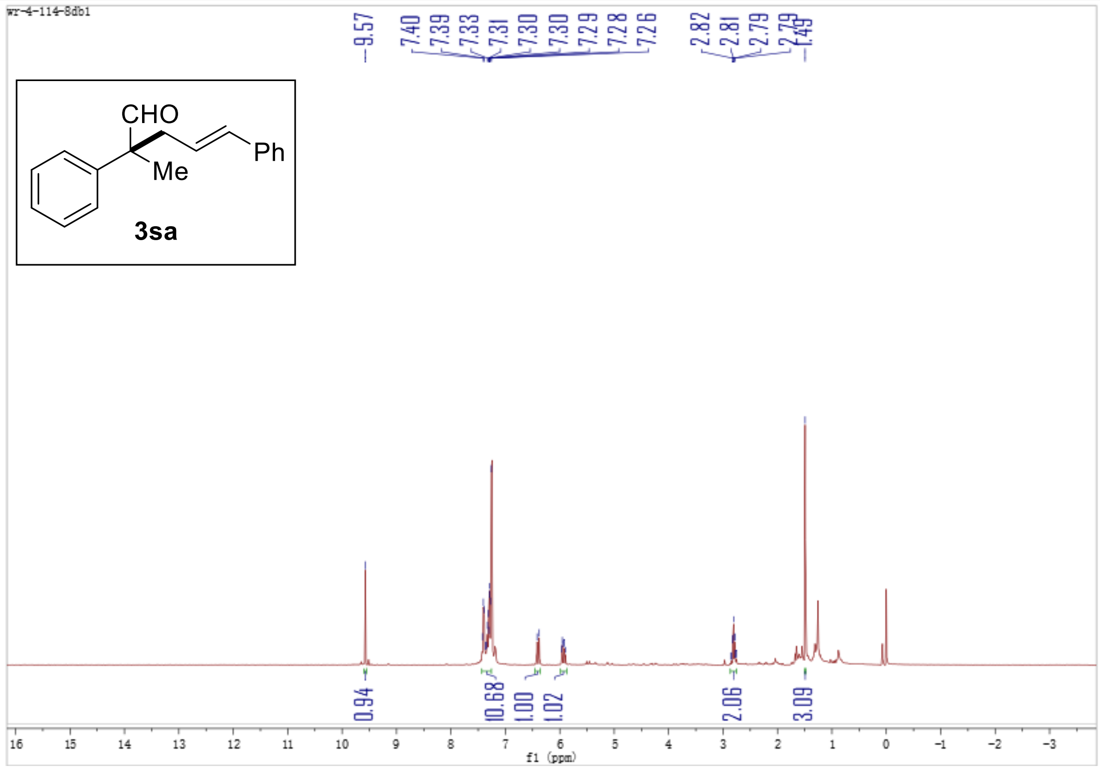

^{13}**C NMR** spectrum of compound **3sa** ($CDCl_3$, 101 MHz)

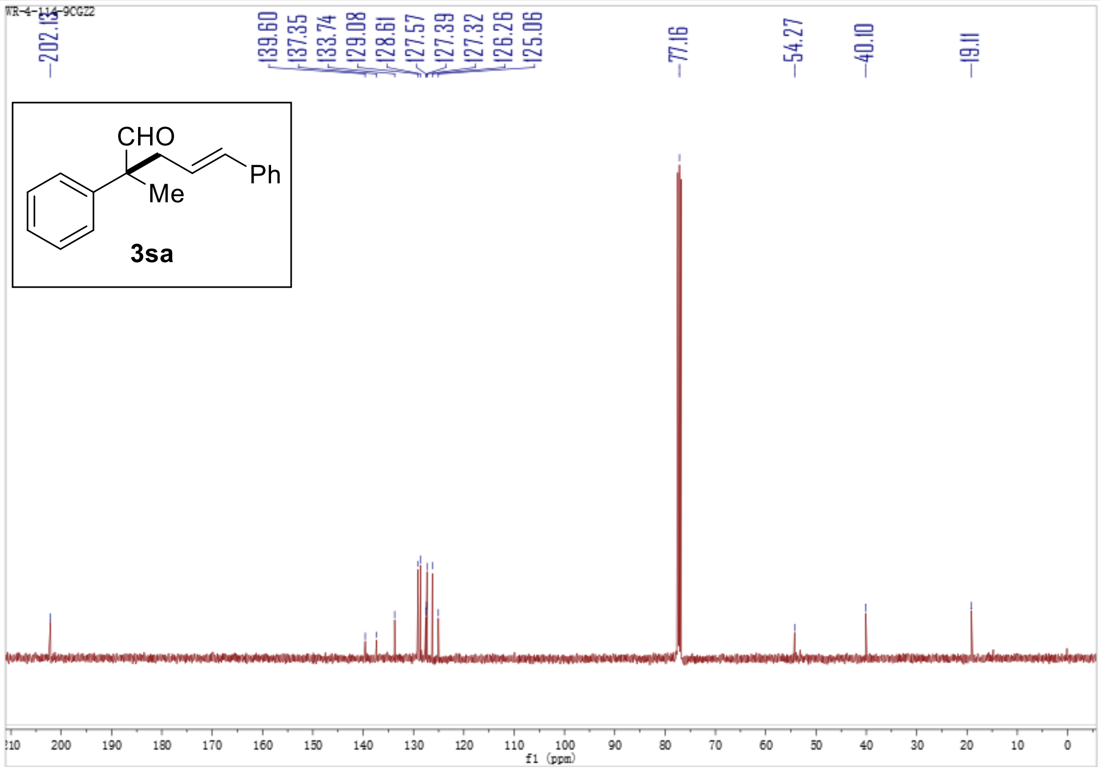

^{1}**H NMR** spectrum of compound **3ta** ($CDCl_3$, 400 MHz)

Links CATALOG DETAILS NMR

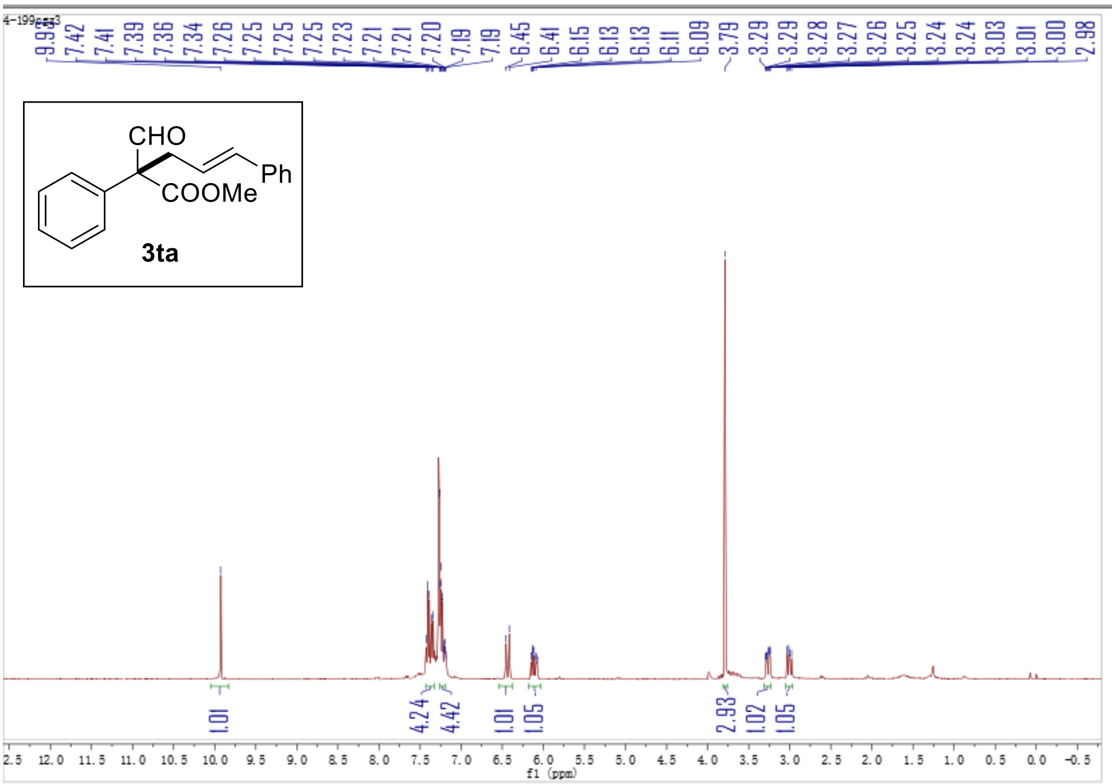

^{13}**C NMR** spectrum of compound **3ta** ($CDCl_3$, 101 MHz)

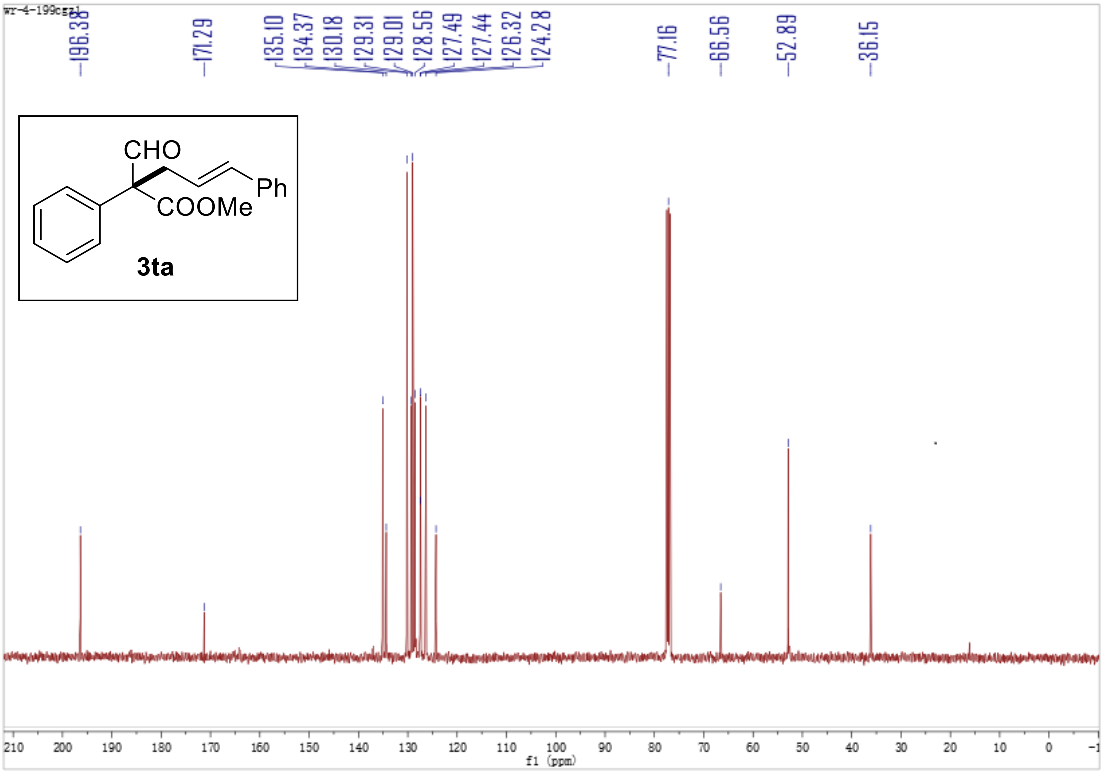

^{1}H NMR spectrum of compound **3hb** ($CDCl_3$, 400 MHz)

Links CATALOG DETAILS NMR

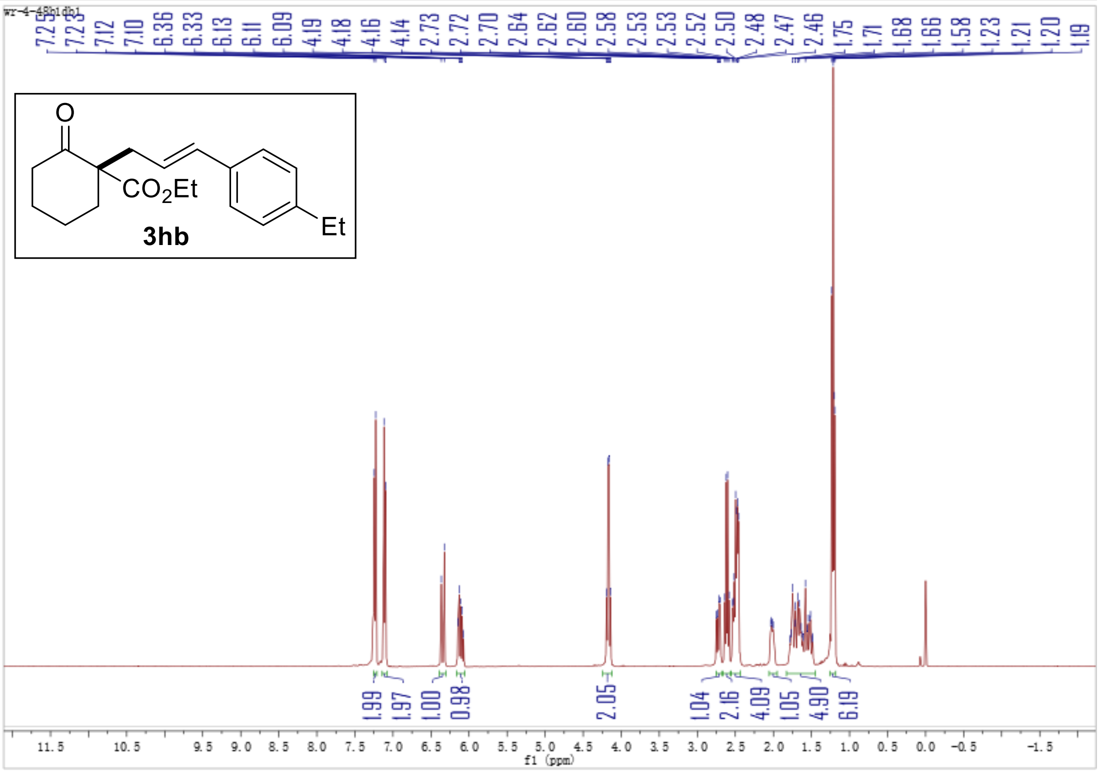

^{13}C NMR spectrum of compound **3hb** ($CDCl_3$, 101 MHz)

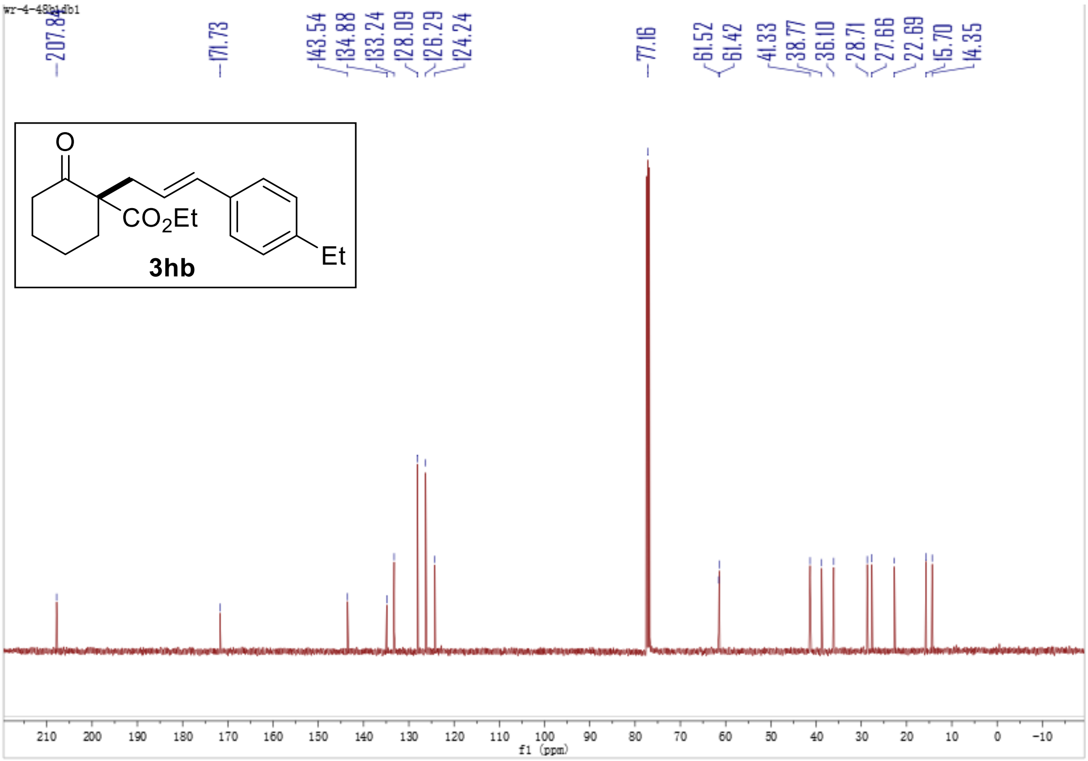

^{1}**H NMR** spectrum of compound **3hc** ($CDCl_3$, 400 MHz)

Links CATALOG DETAILS NMR

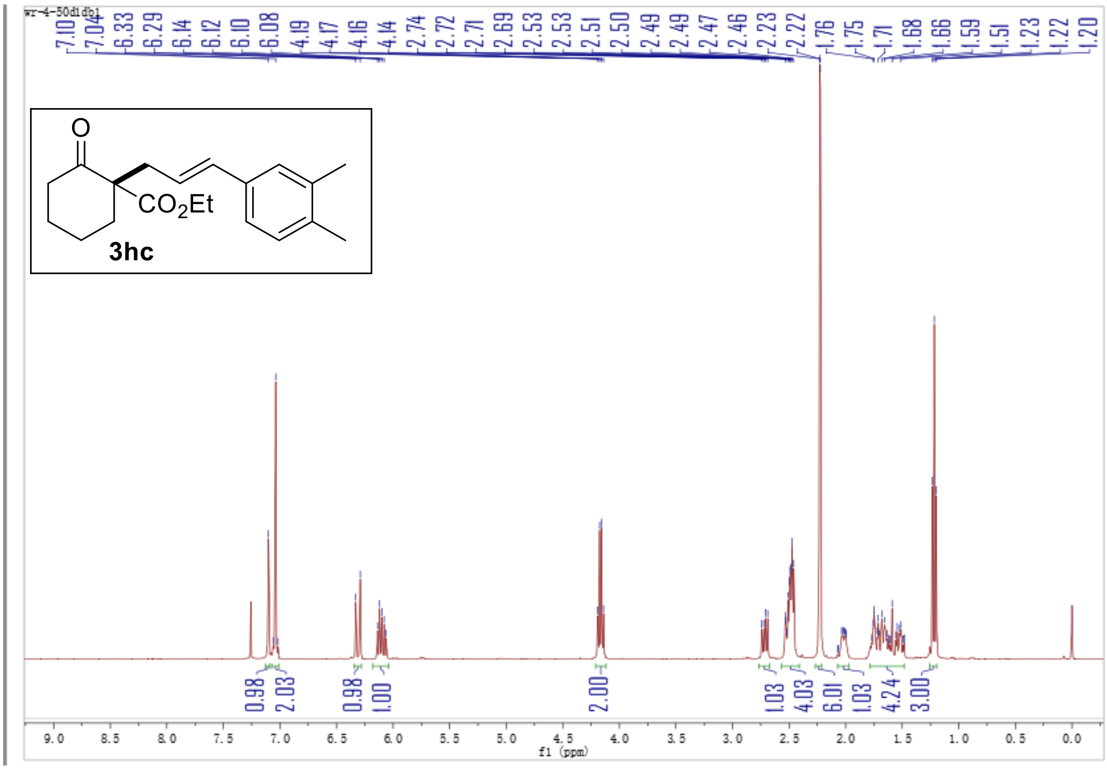

^{13}**C NMR** spectrum of compound **3hc** ($CDCl_3$, 101 MHz)

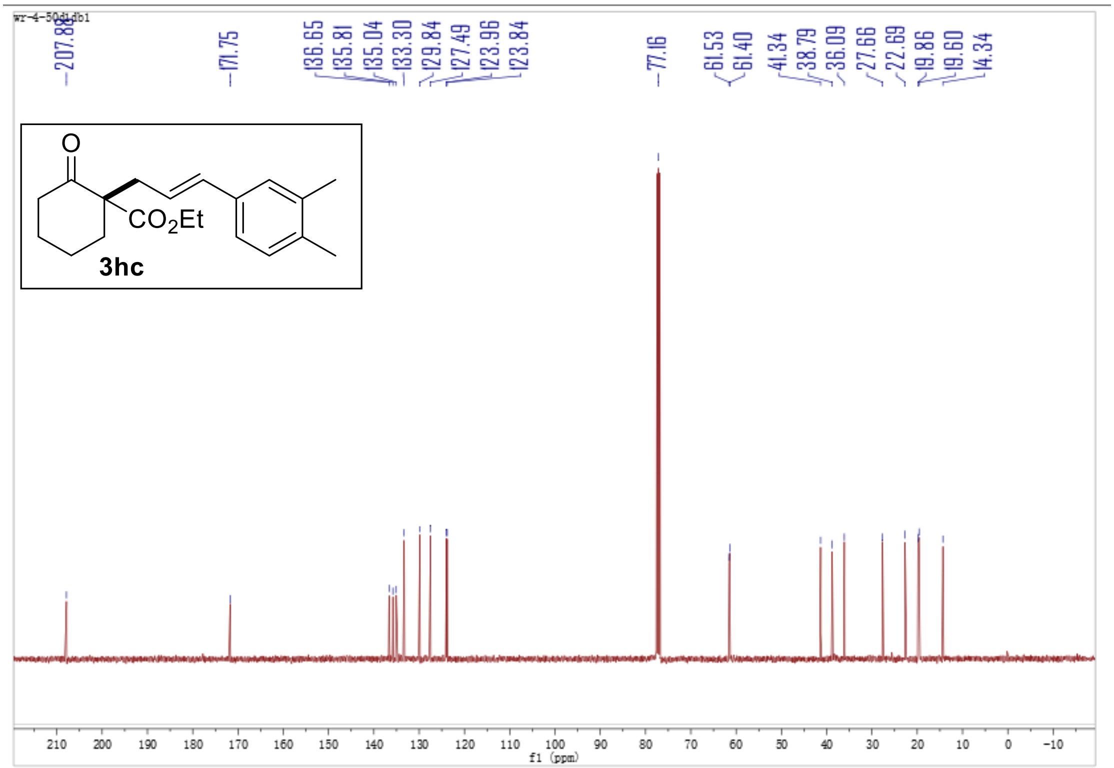

[1]**H NMR** spectrum of compound **3hd** ($CDCl_3$, 400 MHz)

Links CATALOG DETAILS NMR

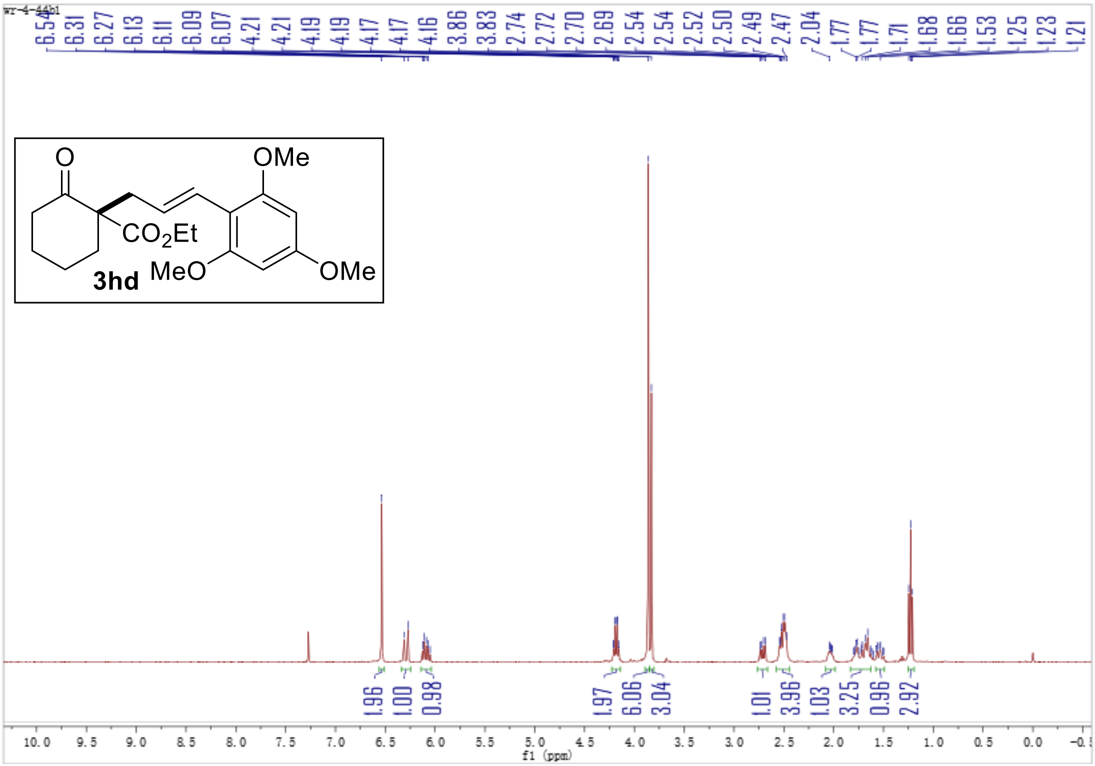

[13]**C NMR** spectrum of compound **3hd** ($CDCl_3$, 101 MHz)

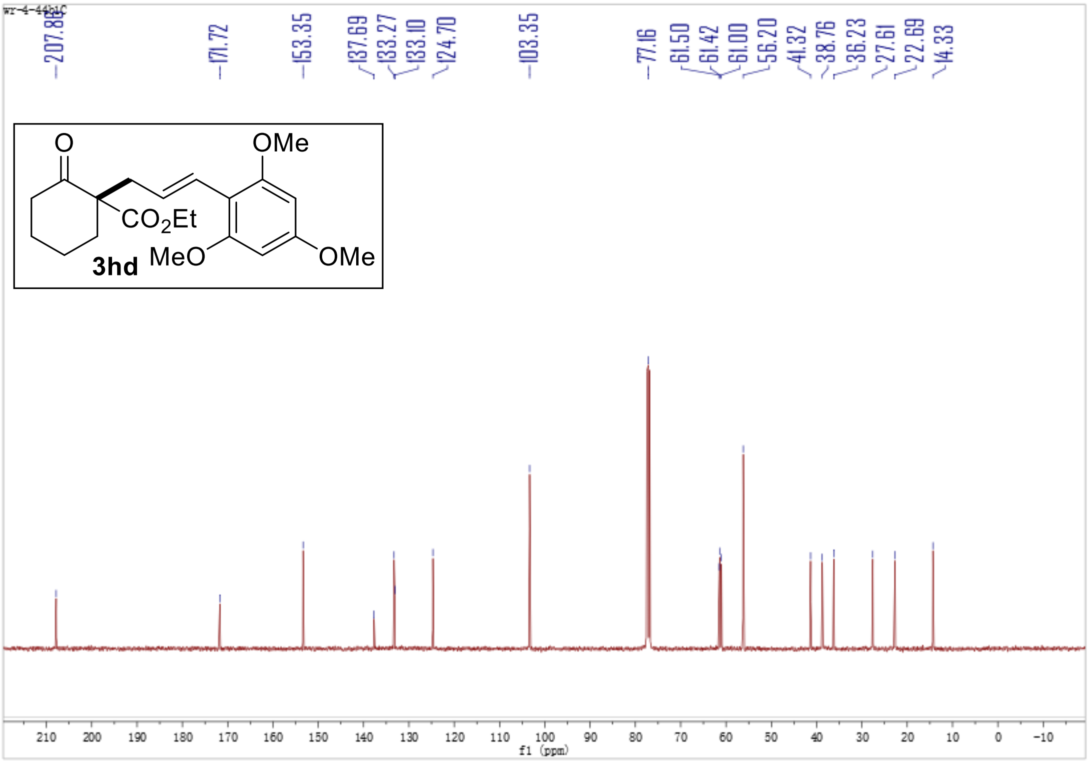

^{1}H NMR spectrum of compound 3he ($CDCl_3$, 400 MHz)

Links CATALOG DETAILS NMR

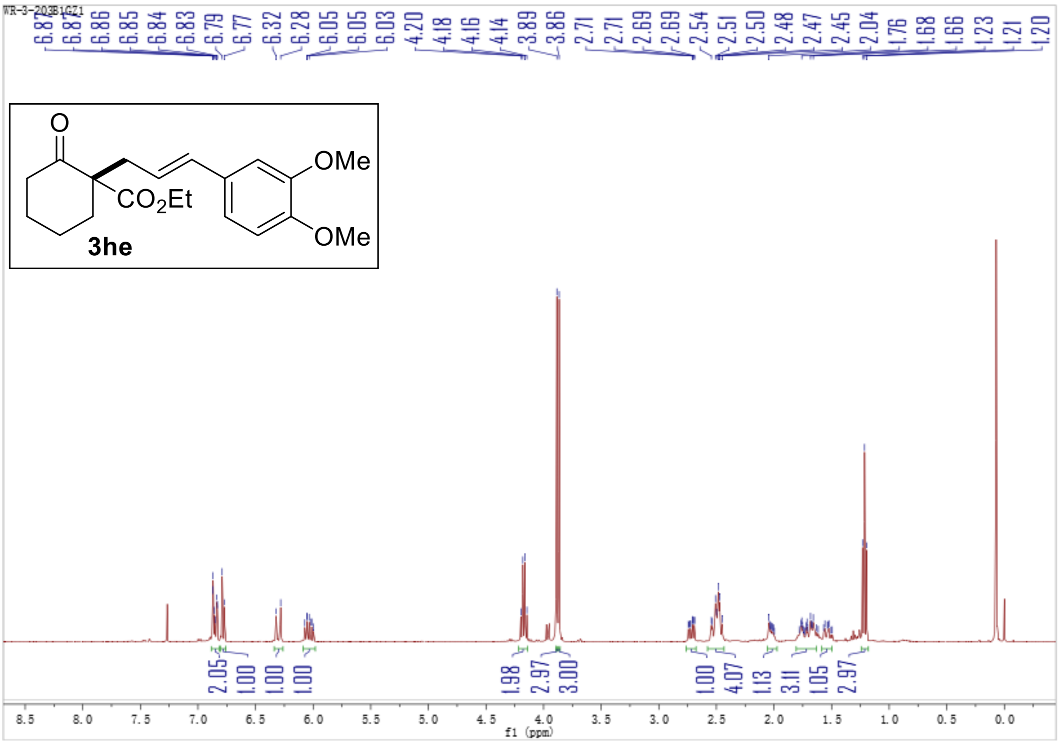

^{13}C NMR spectrum of compound 3he ($CDCl_3$, 101 MHz)

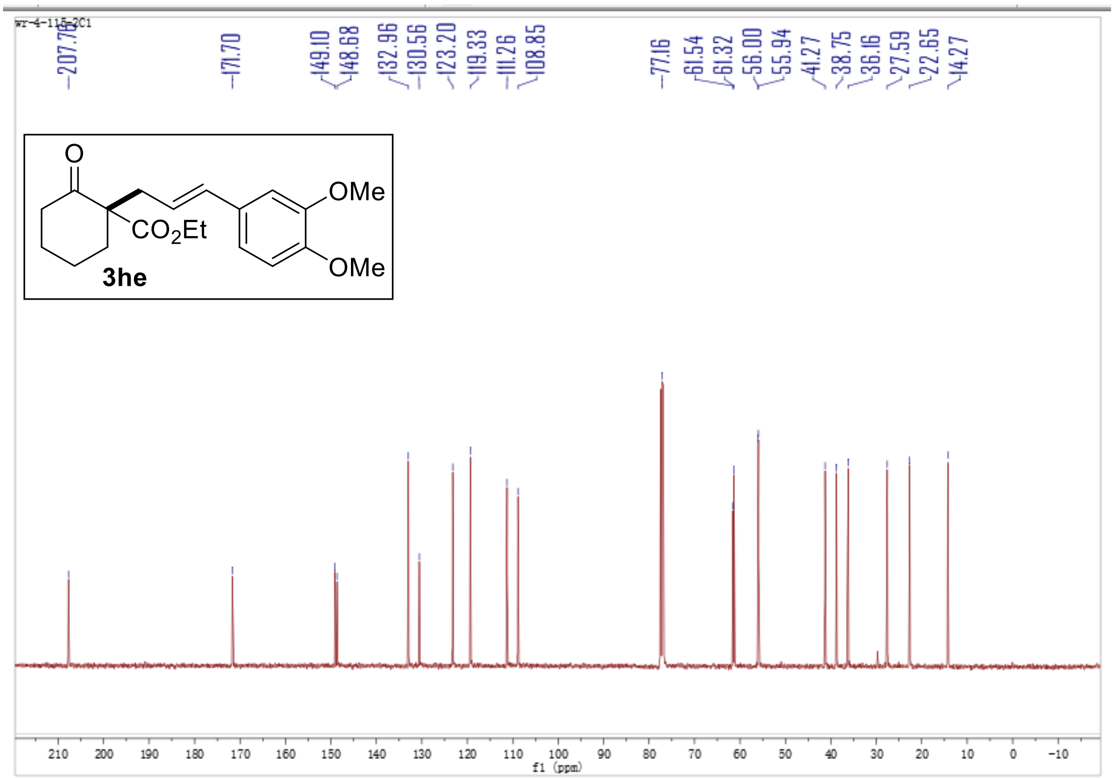

^{1}**H NMR** spectrum of compound **3hf** (CDCl$_3$, 400 MHz)

Links CATALOG DETAILS NMR

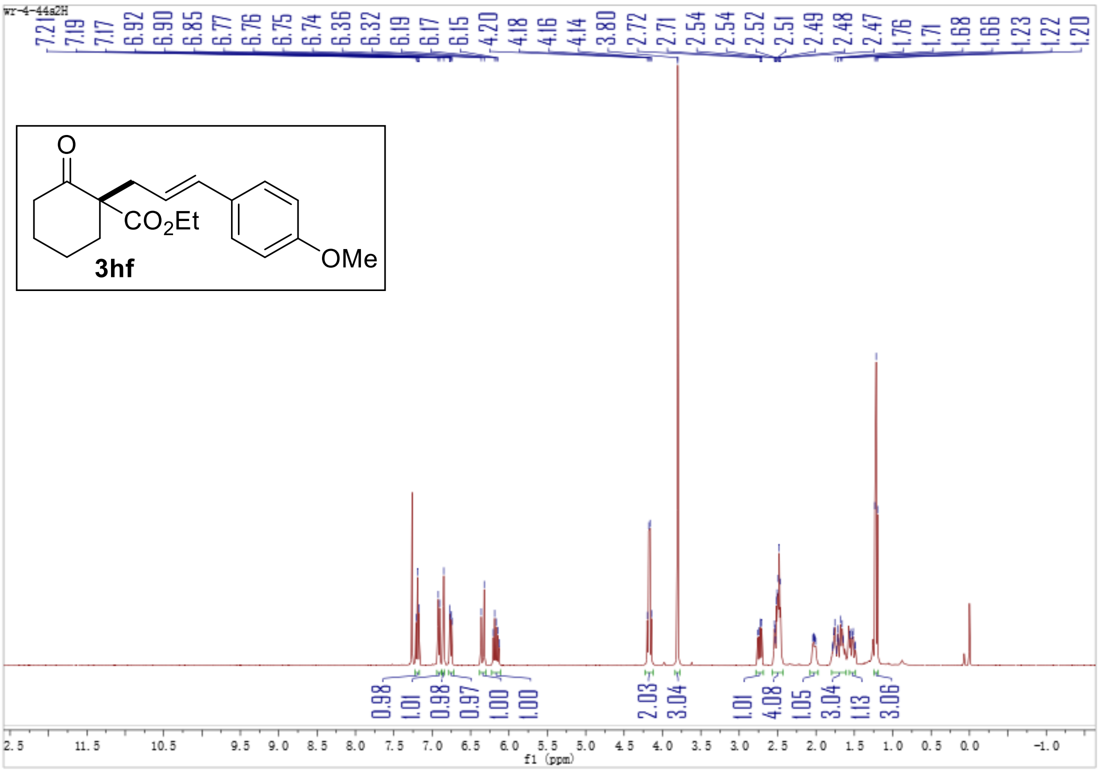

^{13}**C NMR** spectrum of compound **3hf** (CDCl$_3$, 101 MHz)

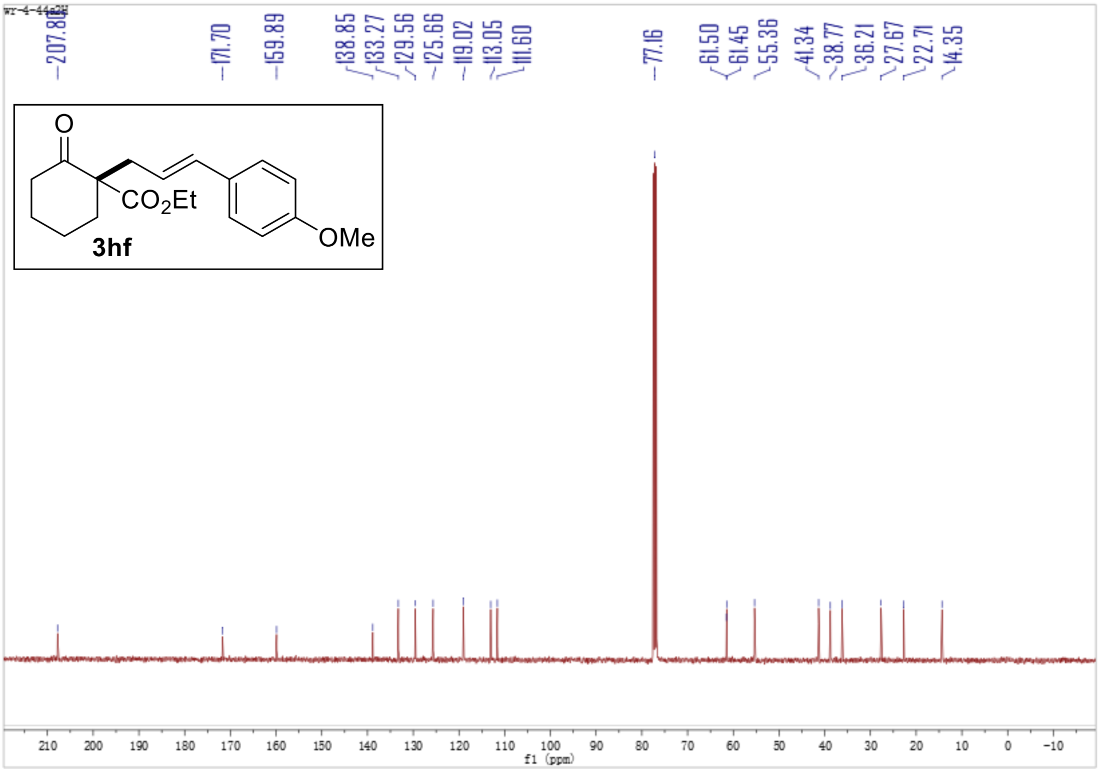

^{1}H NMR spectrum of compound **3hg** ($CDCl_3$, 400 MHz)

Links CATALOG DETAILS NMR

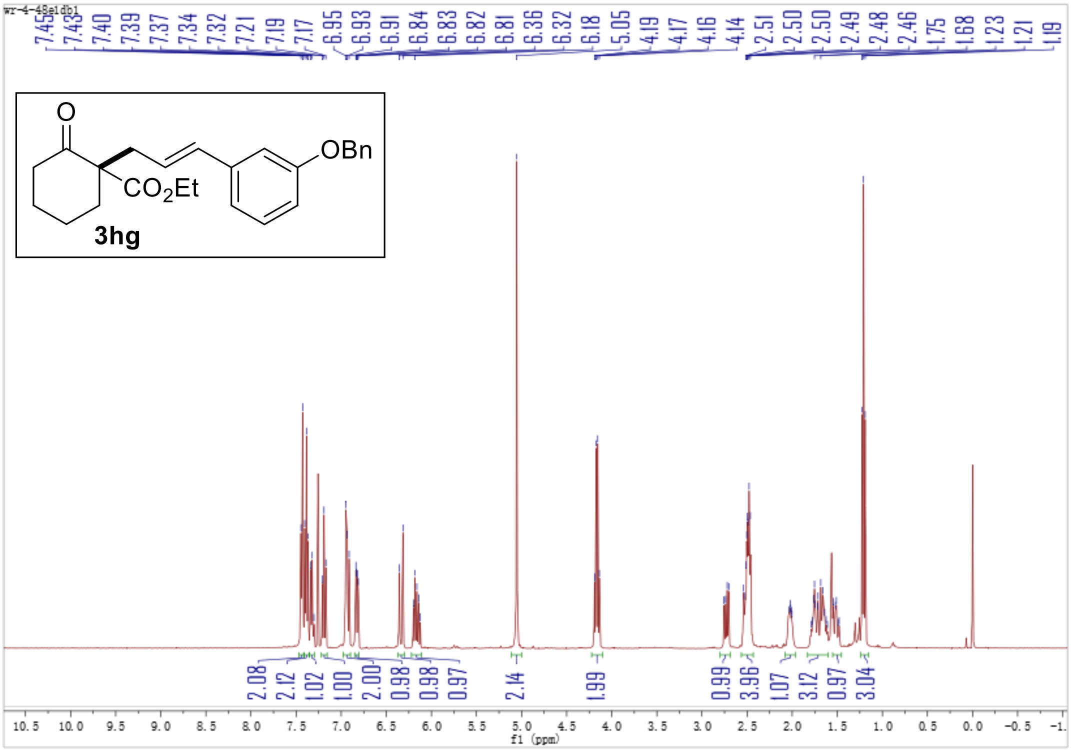

^{13}C NMR spectrum of compound **3hg** ($CDCl_3$, 101 MHz)

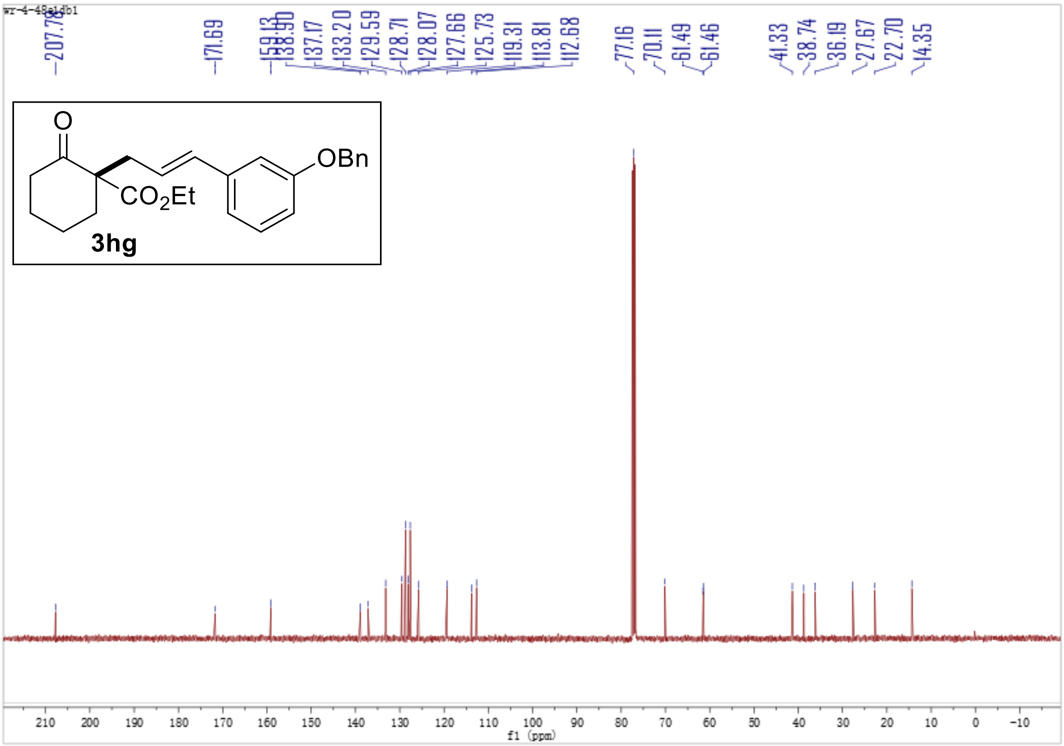

^{1}**H NMR** spectrum of compound **3hh** ($CDCl_3$, 400 MHz)

Links CATALOG DETAILS NMR

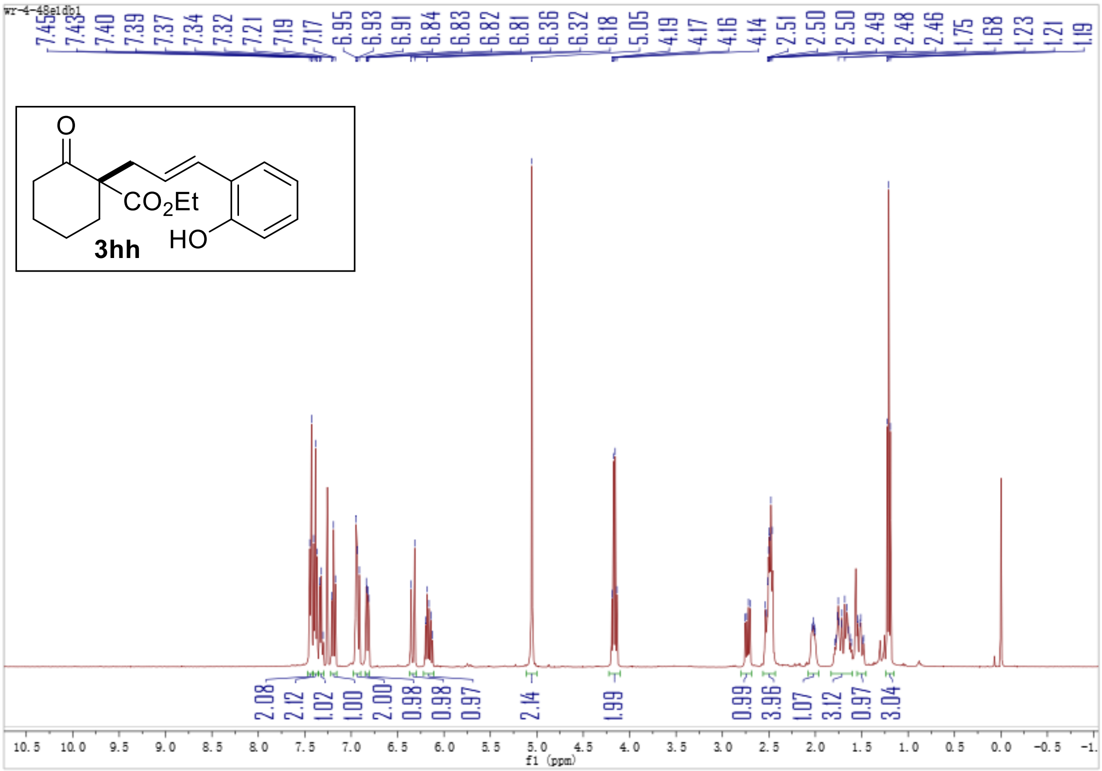

^{13}**C NMR** spectrum of compound **3hh** ($CDCl_3$, 101 MHz)

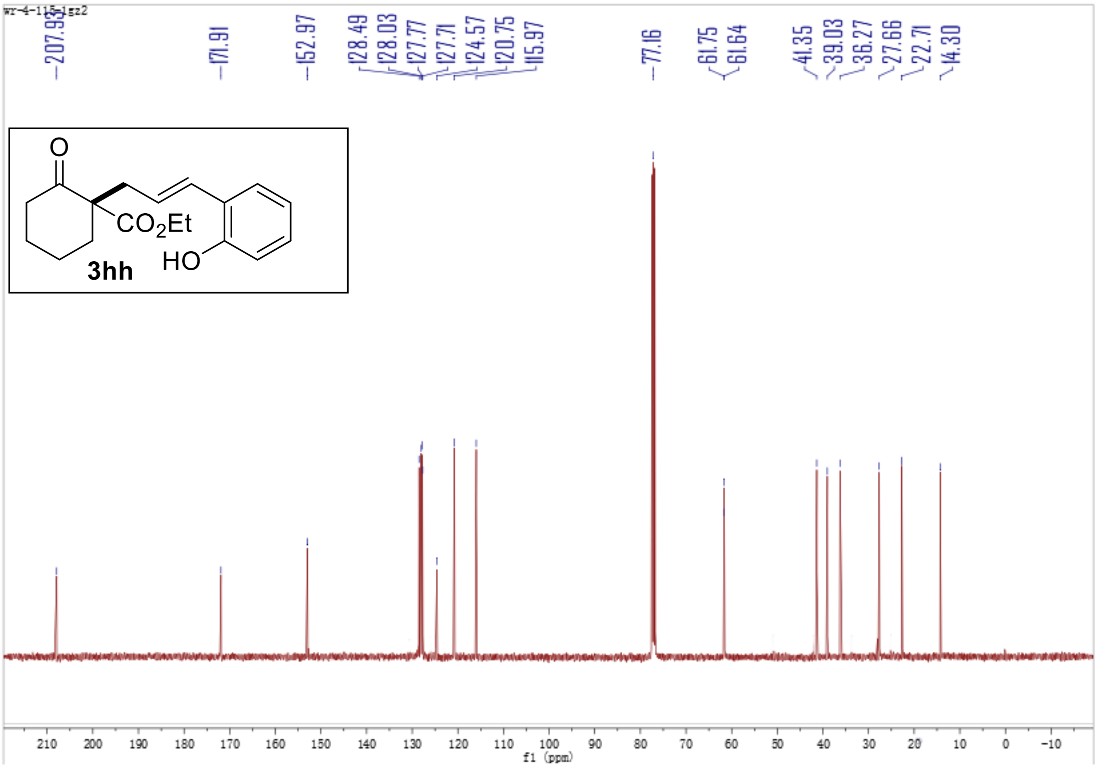

^{1}H NMR spectrum of compound 3hi ($CDCl_3$, 400 MHz)

Links CATALOG DETAILS NMR

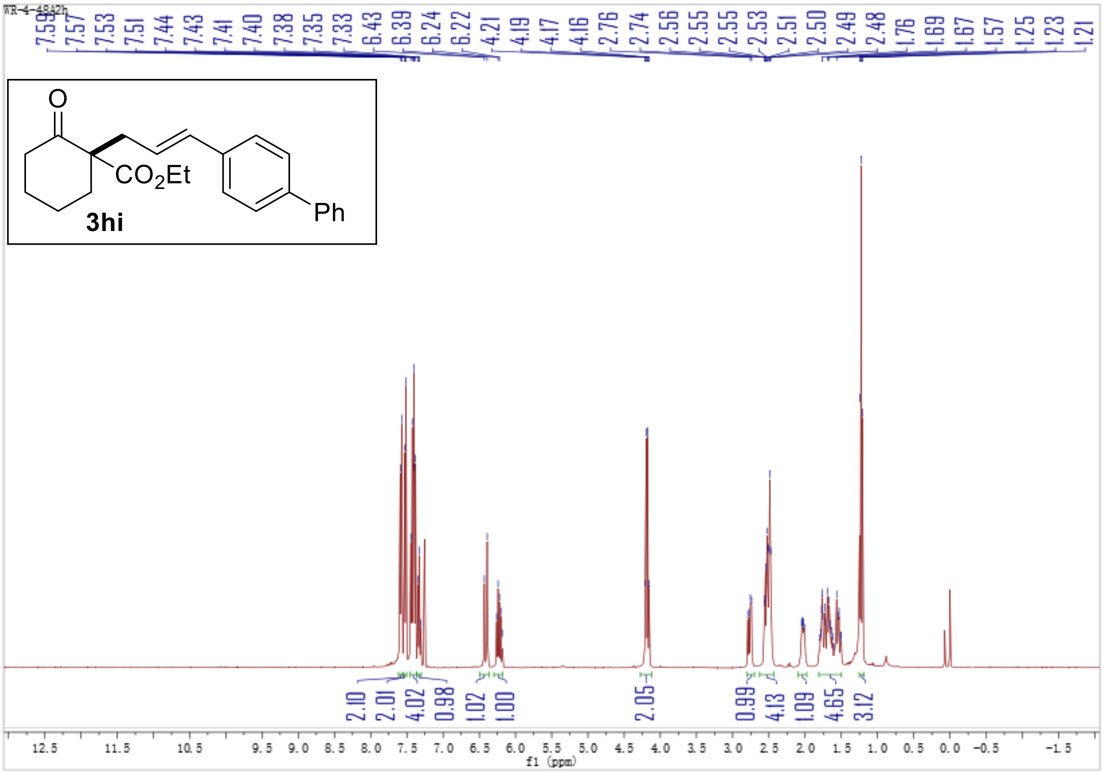

^{13}C NMR spectrum of compound 3hi ($CDCl_3$, 101 MHz)

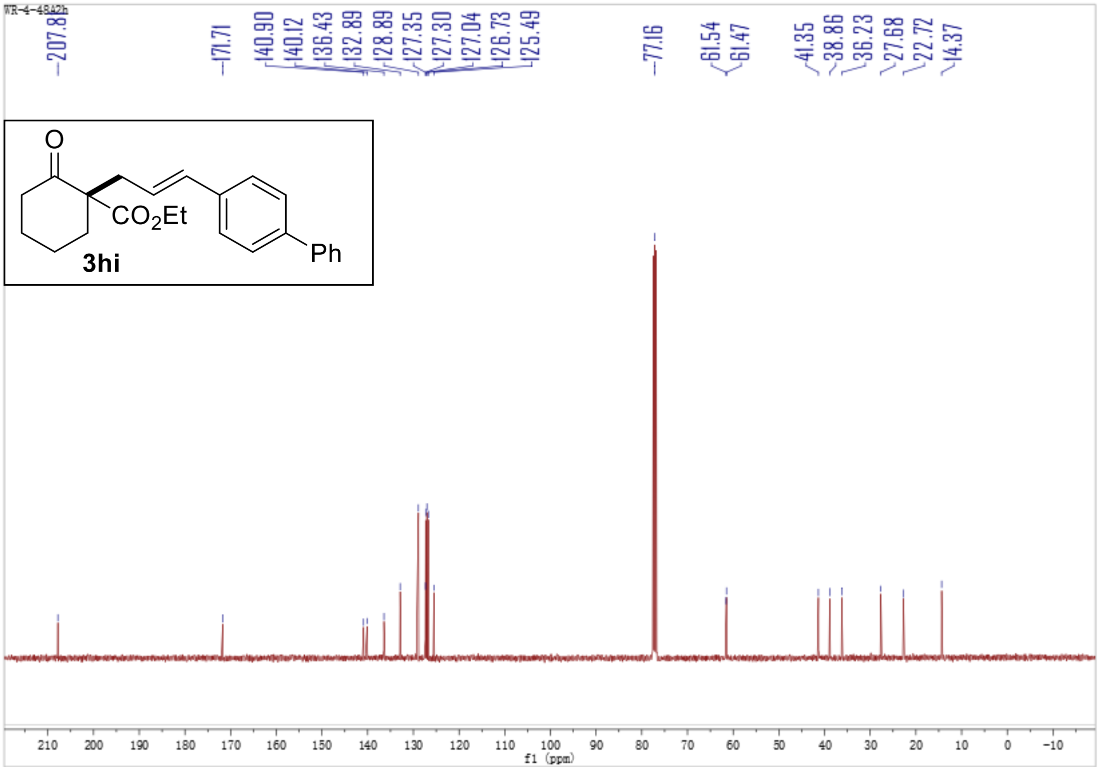

^{1}H NMR spectrum of compound **3hj** ($CDCl_3$, 400 MHz)

Links CATALOG DETAILS NMR

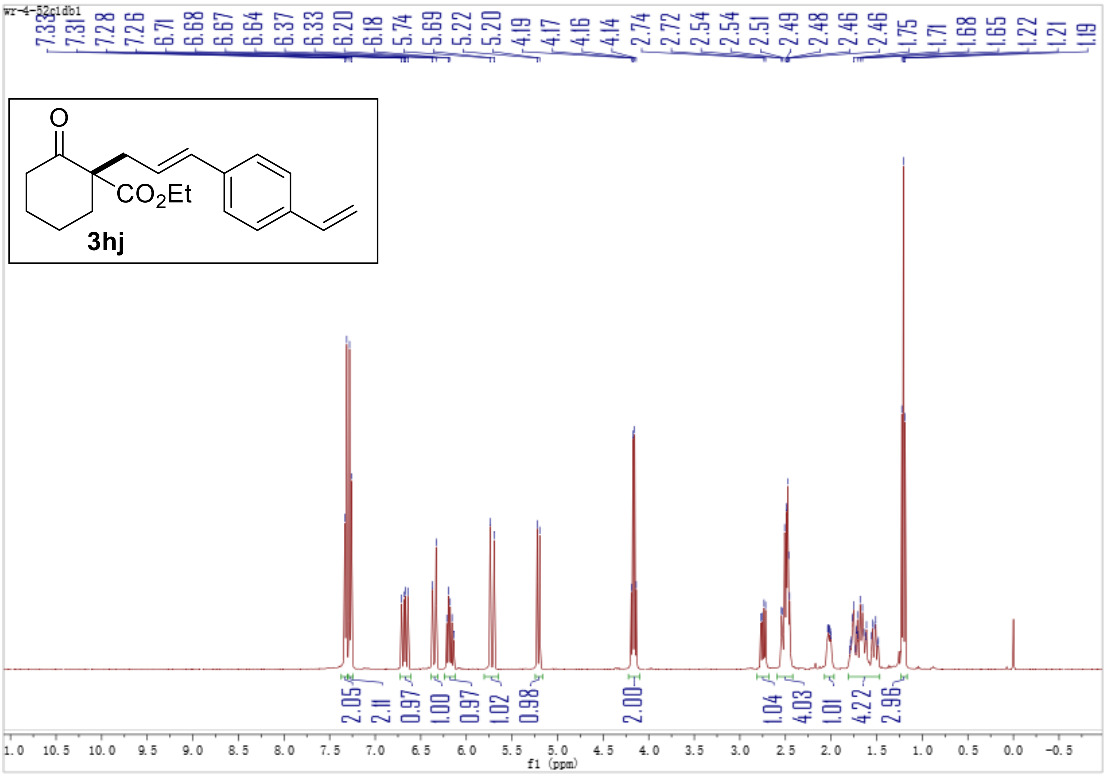

^{13}C NMR spectrum of compound **3hj** ($CDCl_3$, 101 MHz)

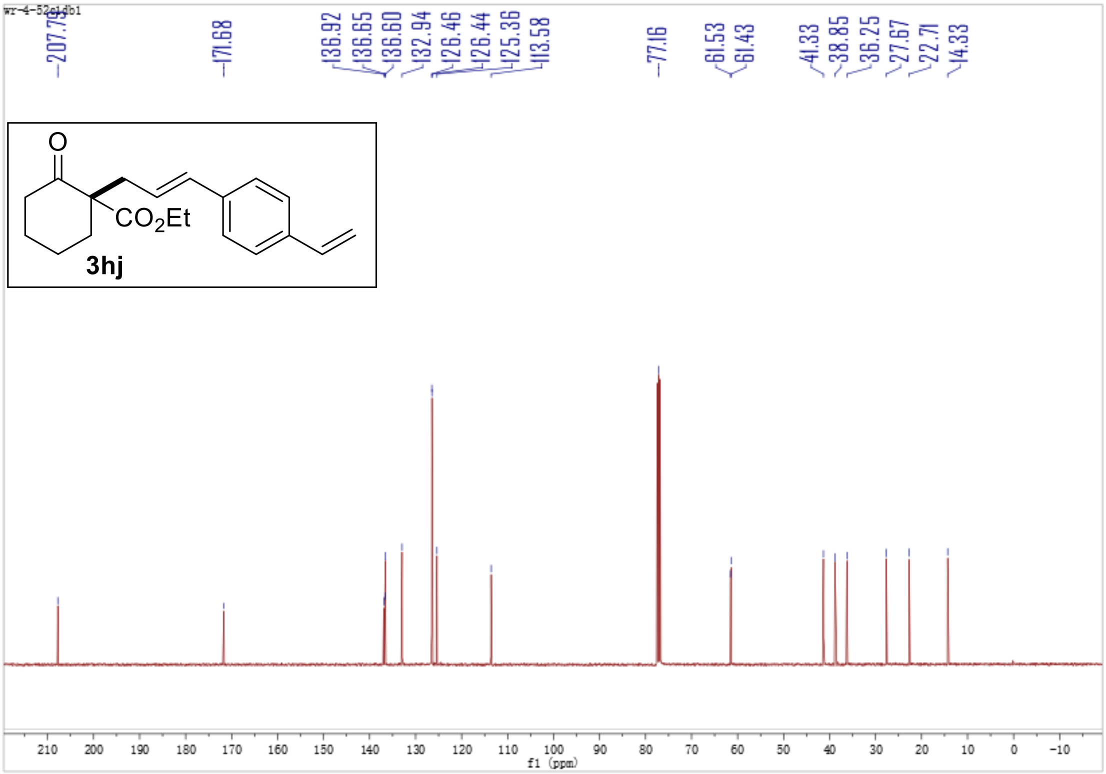

^{1}**H NMR** spectrum of compound **3hk** ($CDCl_3$, 400 MHz)

Links CATALOG DETAILS NMR

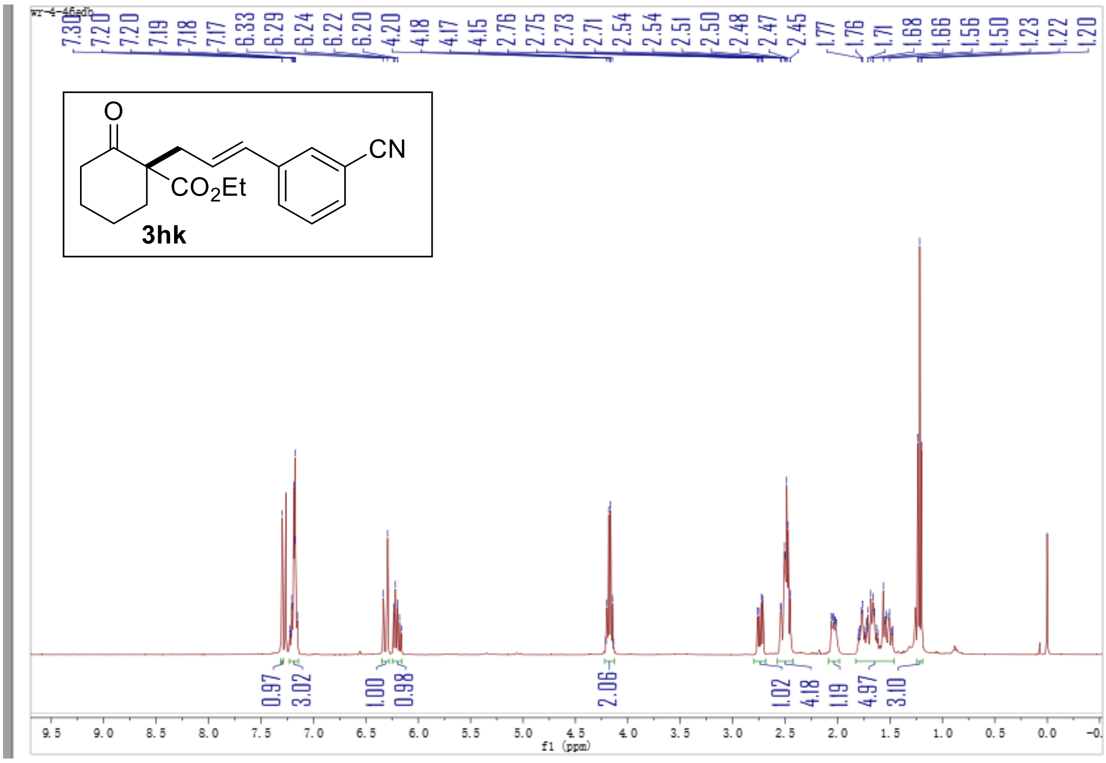

^{13}**C NMR** spectrum of compound **3hk** ($CDCl_3$, 101 MHz)

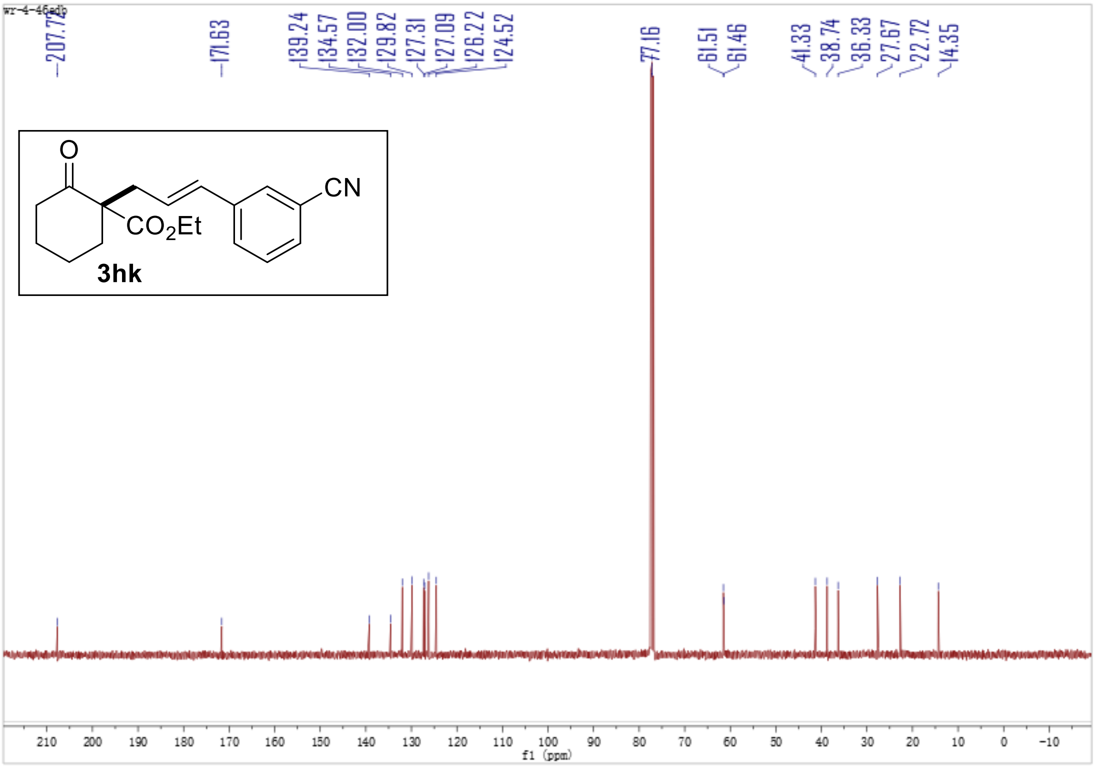

^{1}H NMR spectrum of compound **3hl** ($CDCl_3$, 400 MHz)

Links CATALOG DETAILS NMR

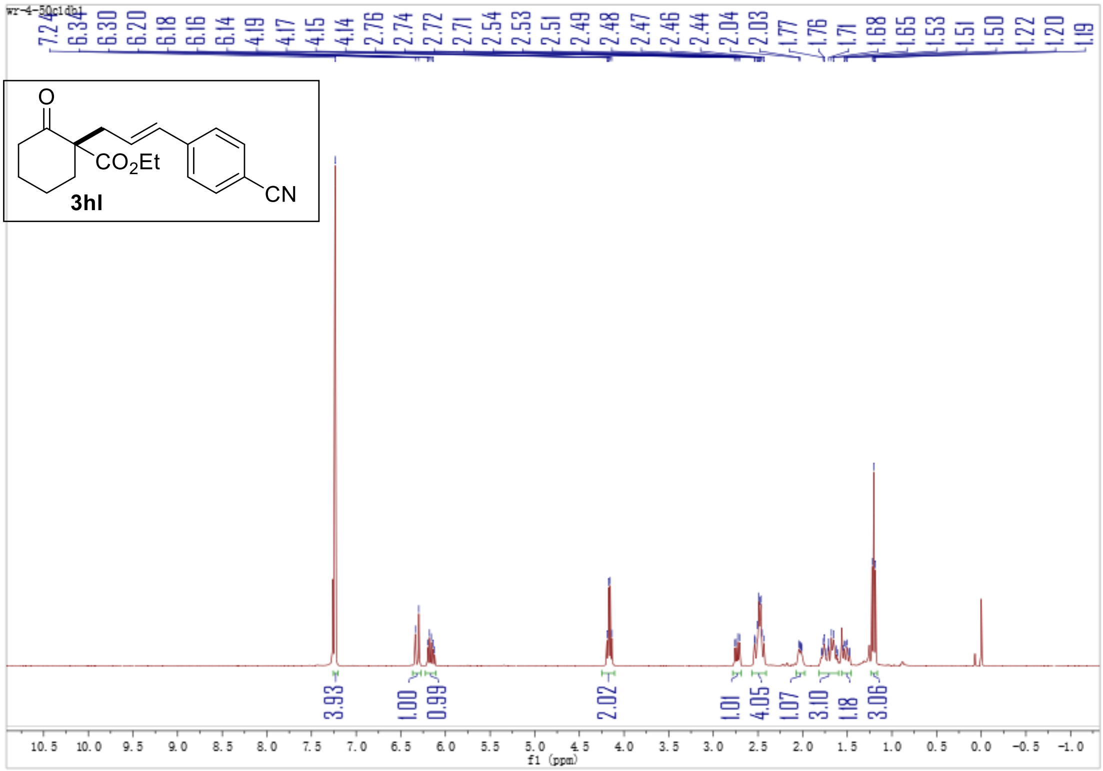

^{13}C NMR spectrum of compound **3hl** ($CDCl_3$, 101 MHz)

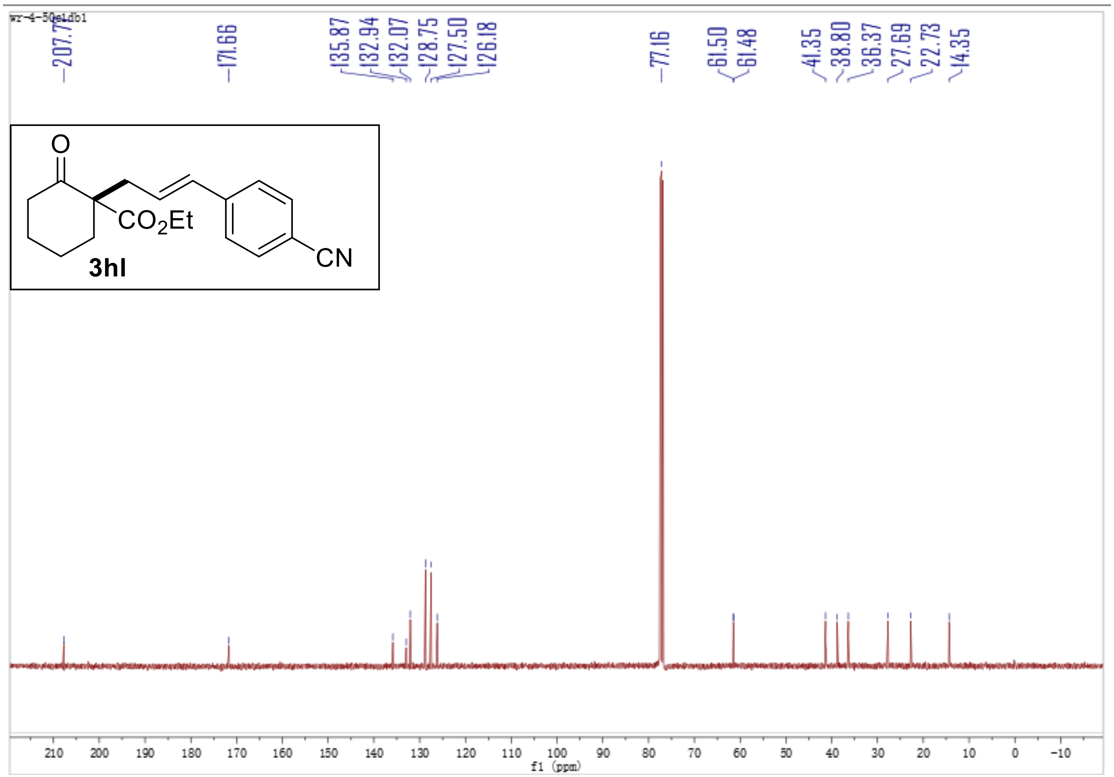

^{1}H NMR spectrum of compound **3hm** ($CDCl_3$, 400 MHz)

Links CATALOG DETAILS NMR

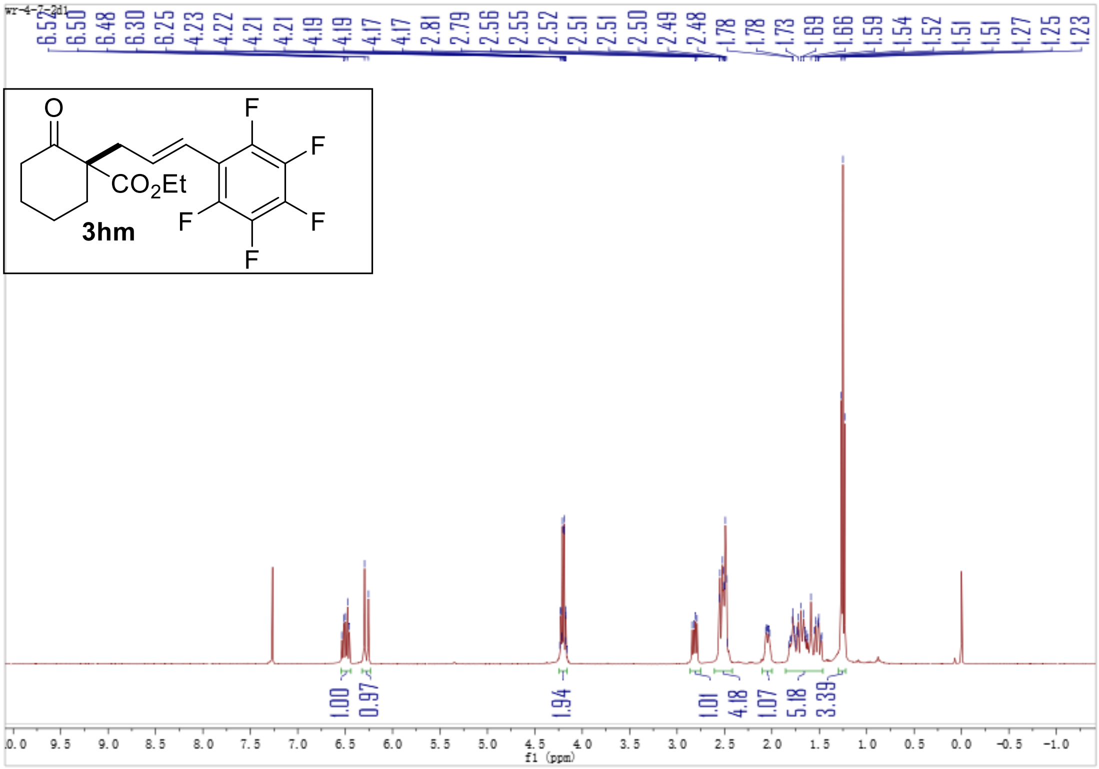

^{13}C NMR spectrum of compound **3hm** ($CDCl_3$, 101 MHz)

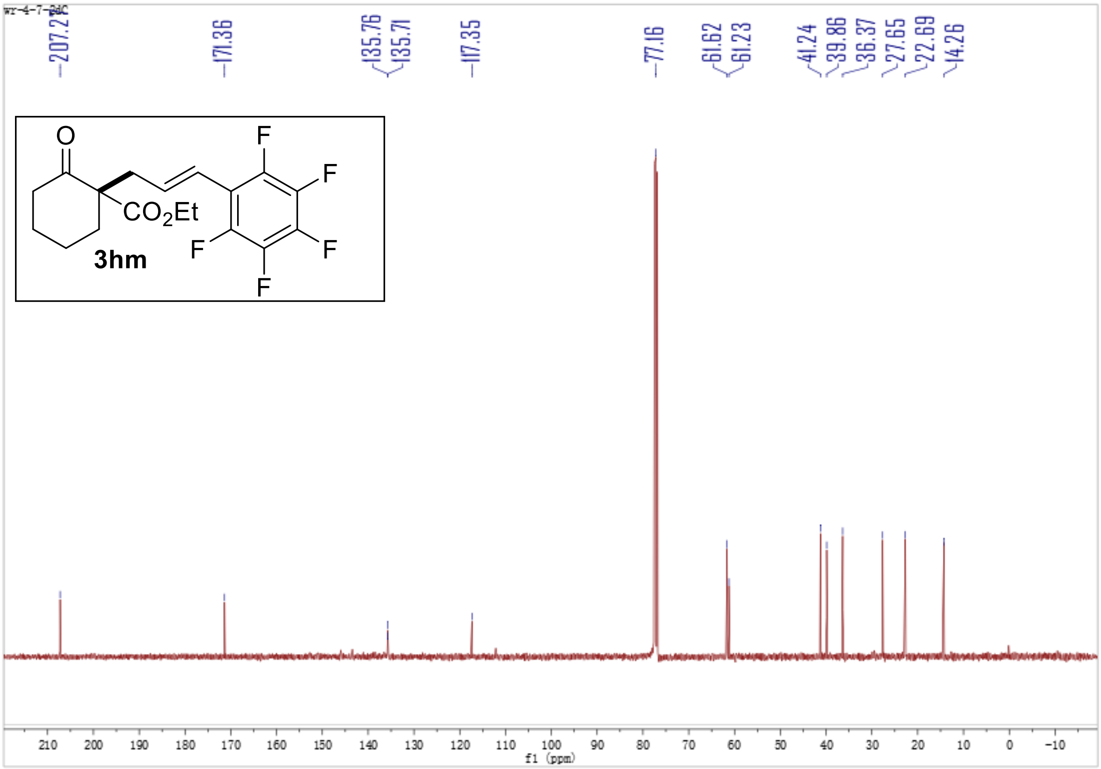

^{19}F NMR spectrum of compound **3hm** ($CDCl_3$, 376 MHz)

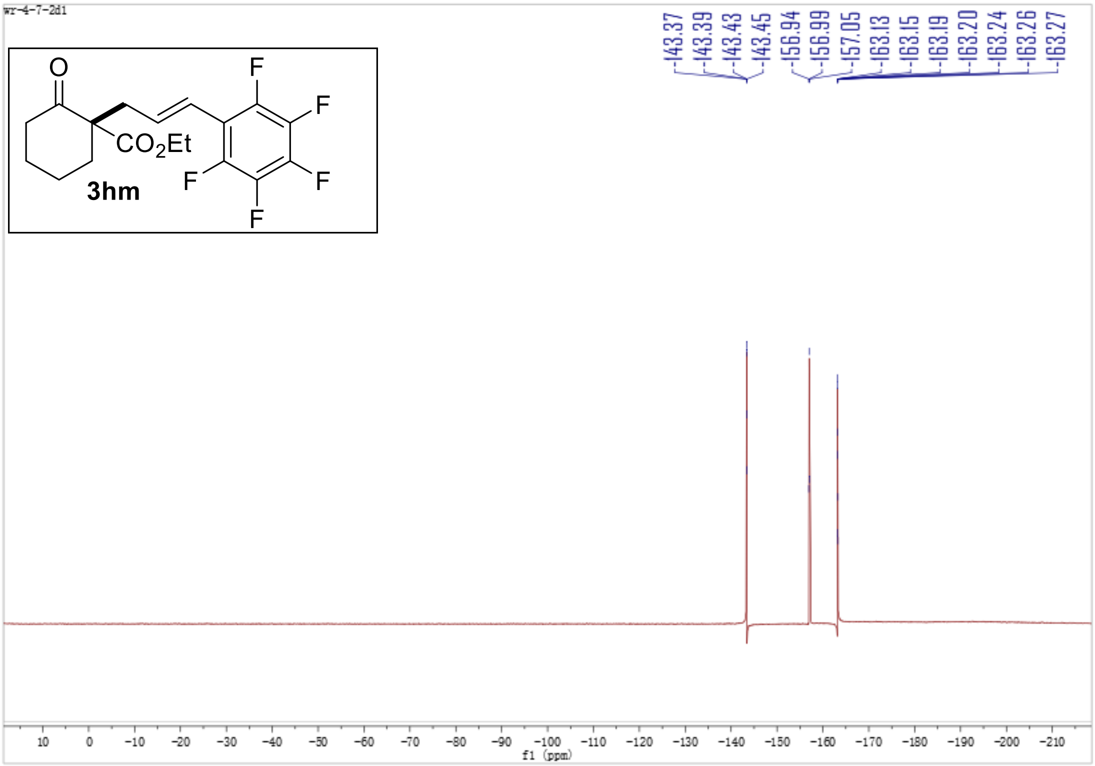

^{1}H NMR spectrum of compound 3hn ($CDCl_3$, 400 MHz)

Links CATALOG DETAILS NMR

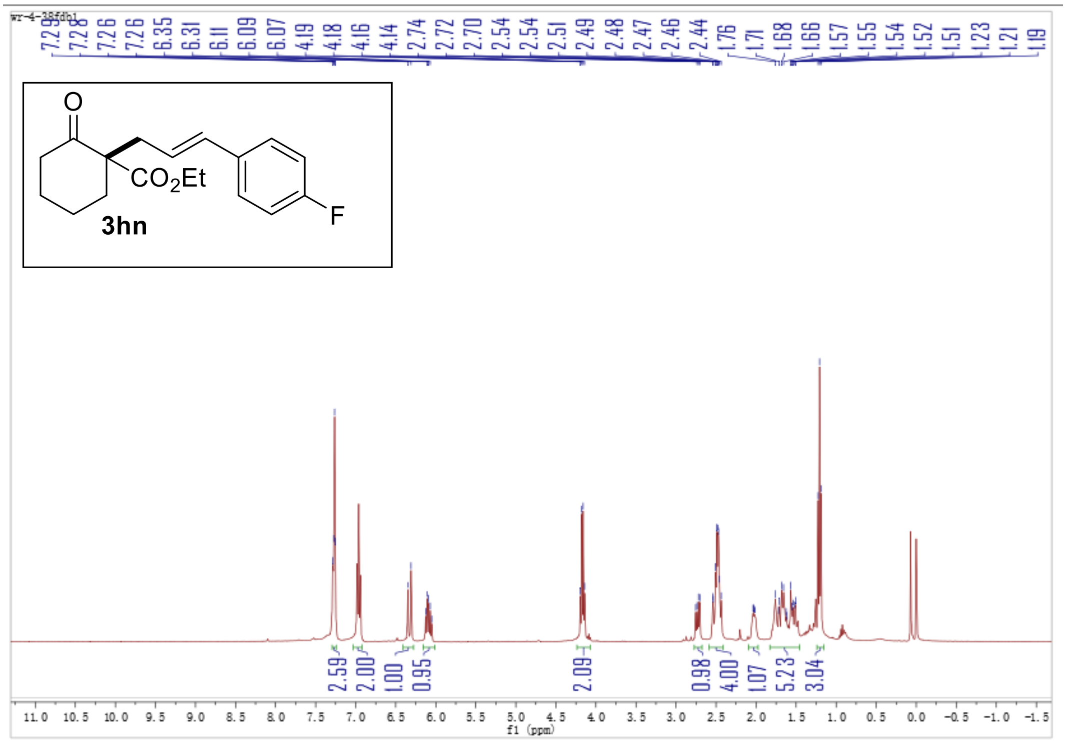

^{13}C NMR spectrum of compound 3hn ($CDCl_3$, 101 MHz)

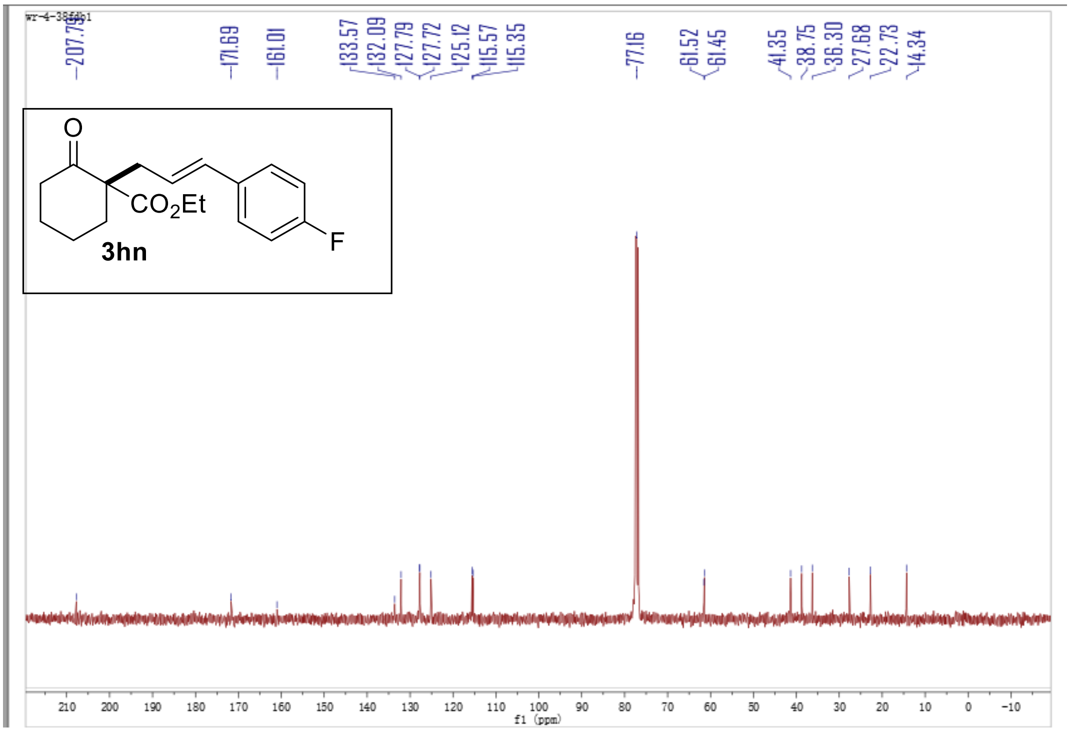

^{19}F NMR spectrum of compound **3hn** ($CDCl_3$, 376MHz)

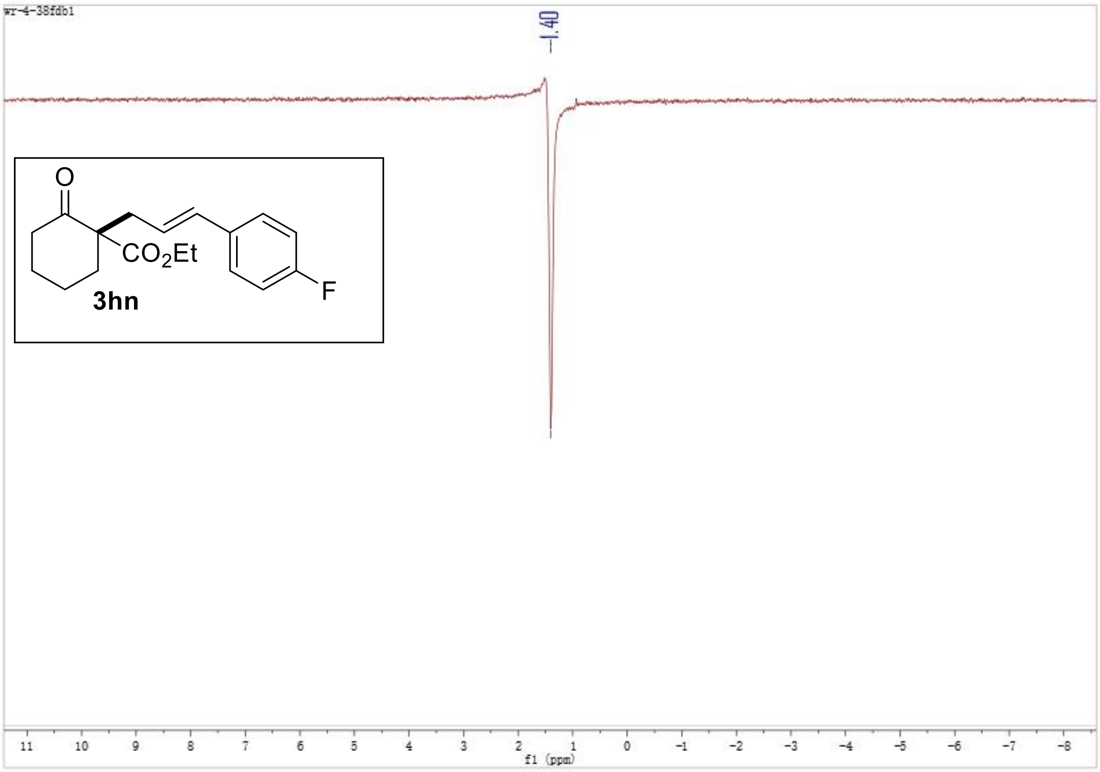

^{1}H NMR spectrum of compound **3ho** ($CDCl_3$, 400 MHz)

Links CATALOG DETAILS NMR

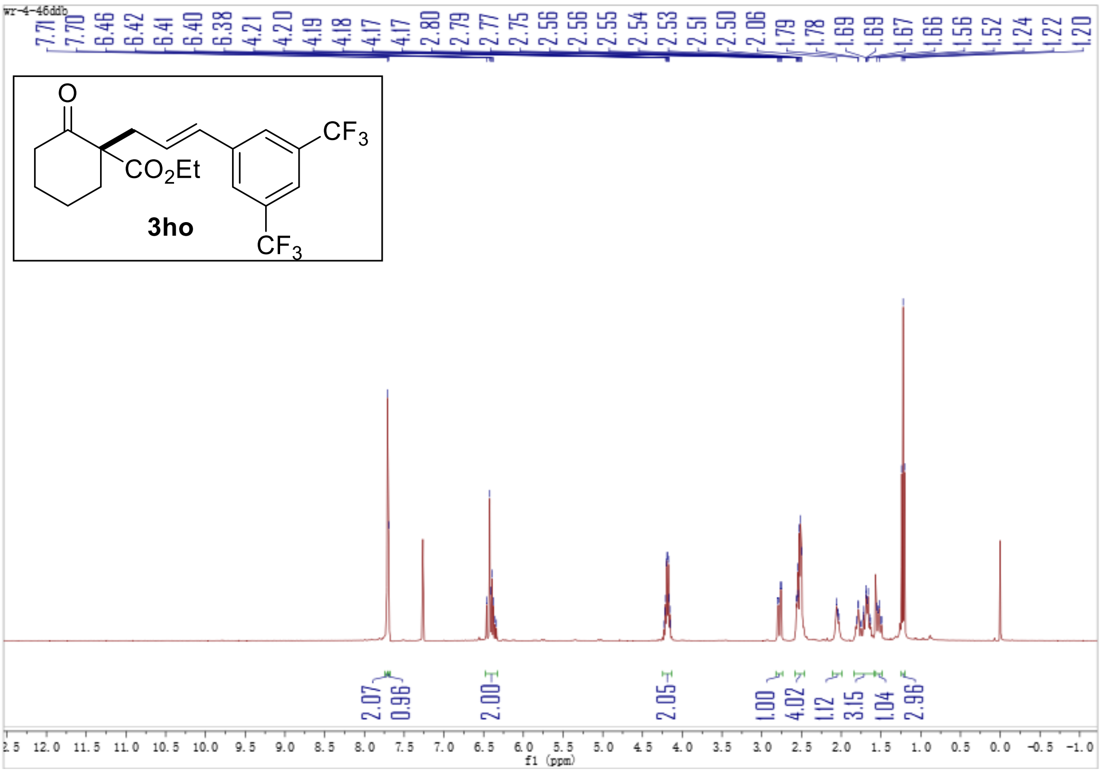

^{13}C NMR spectrum of compound **3ho** ($CDCl_3$, 101 MHz)

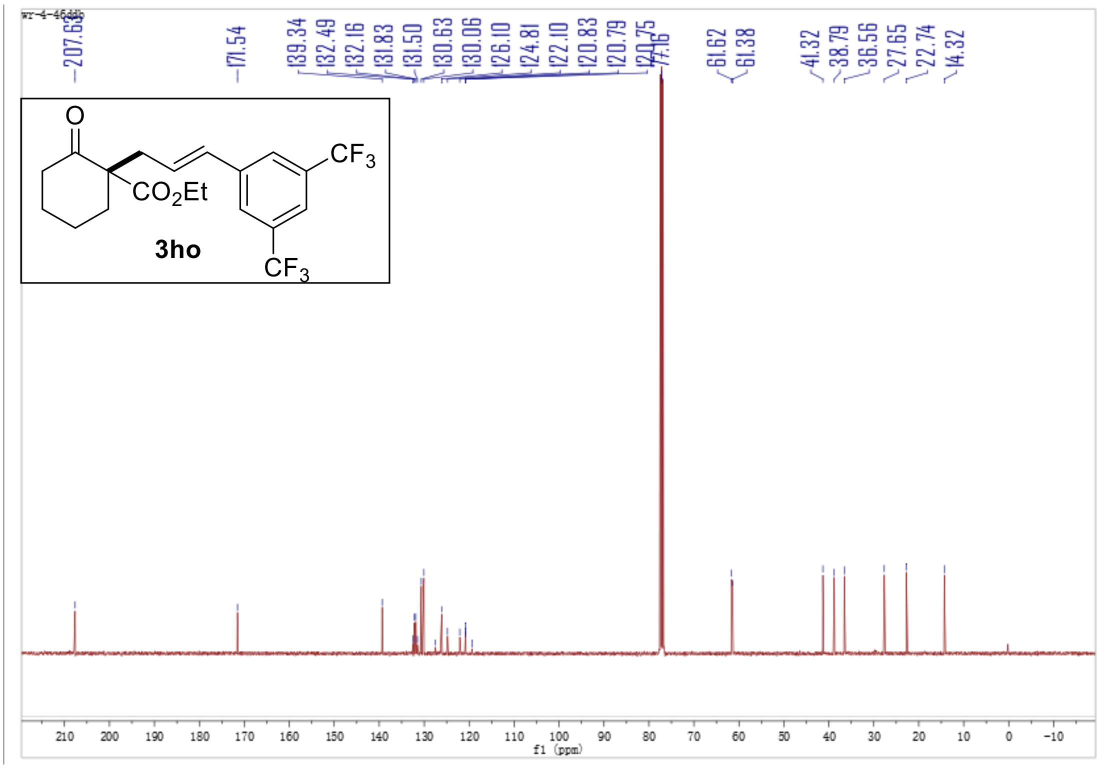

^{1}H **NMR** spectrum of compound **3hp** ($CDCl_3$, 400 MHz)

Links CATALOG DETAILS NMR

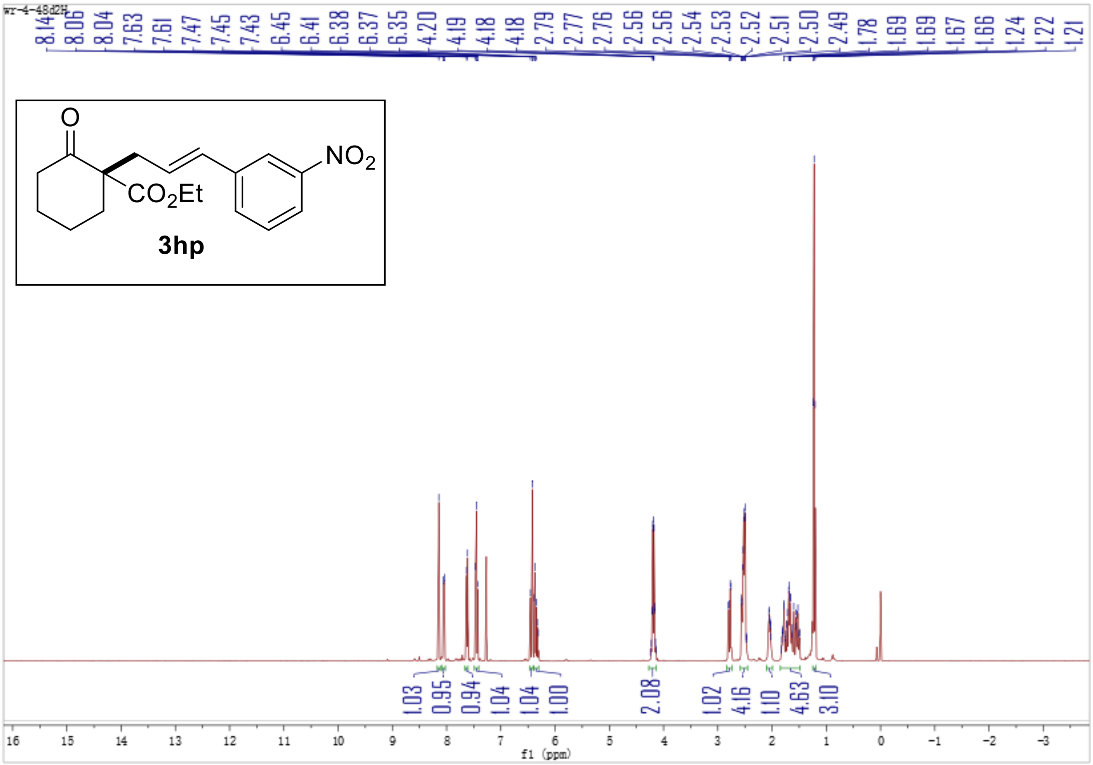

^{13}C **NMR** spectrum of compound **3hp** ($CDCl_3$, 101 MHz)

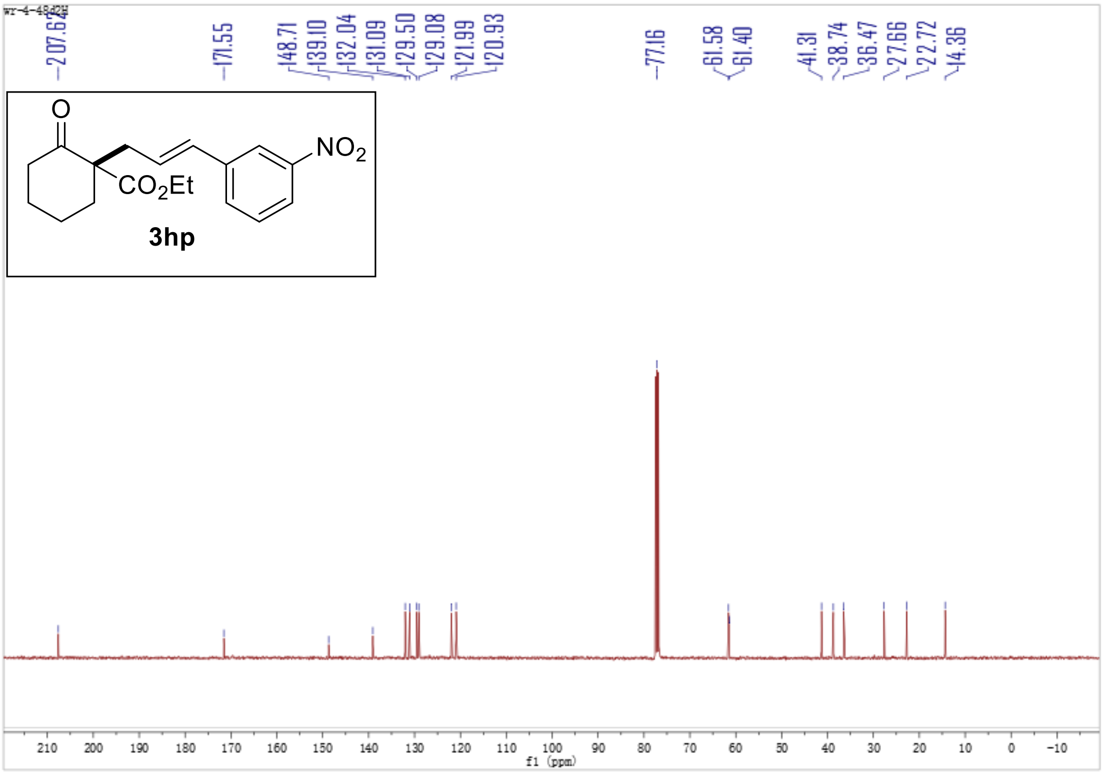

^{1}**H NMR** spectrum of compound **3hq** ($CDCl_3$, 400 MHz)

Links CATALOG DETAILS NMR

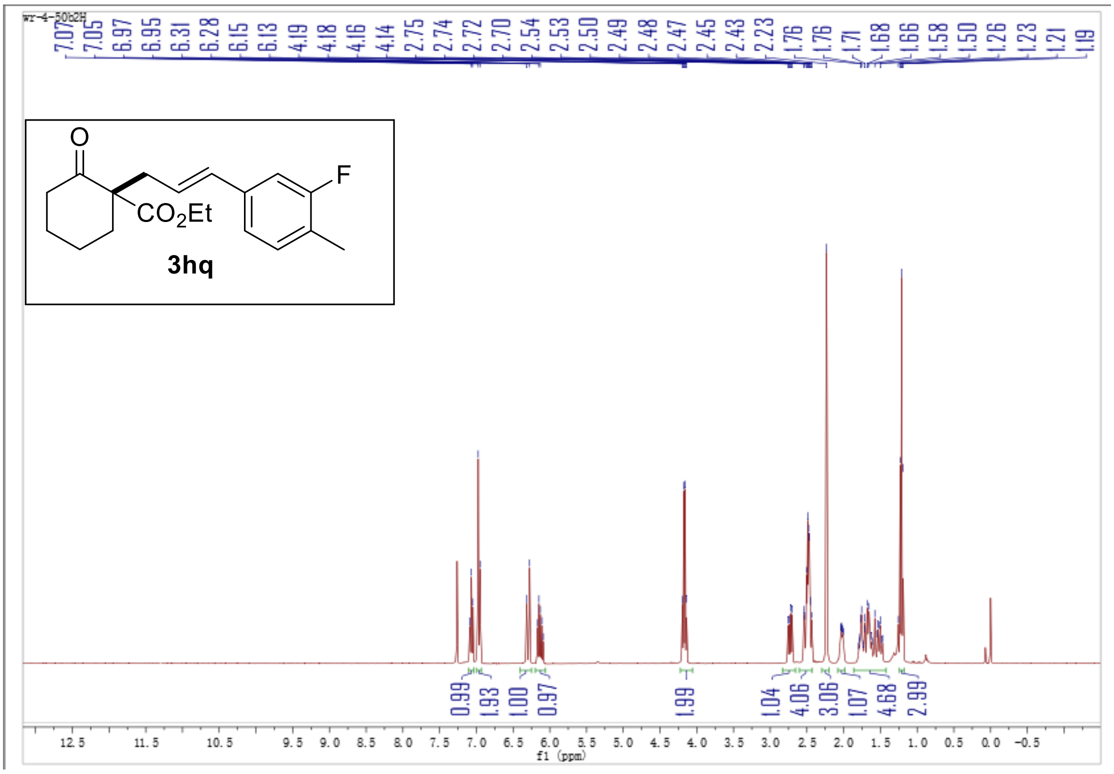

^{13}**C NMR** spectrum of compound **3hq** ($CDCl_3$, 101 MHz)

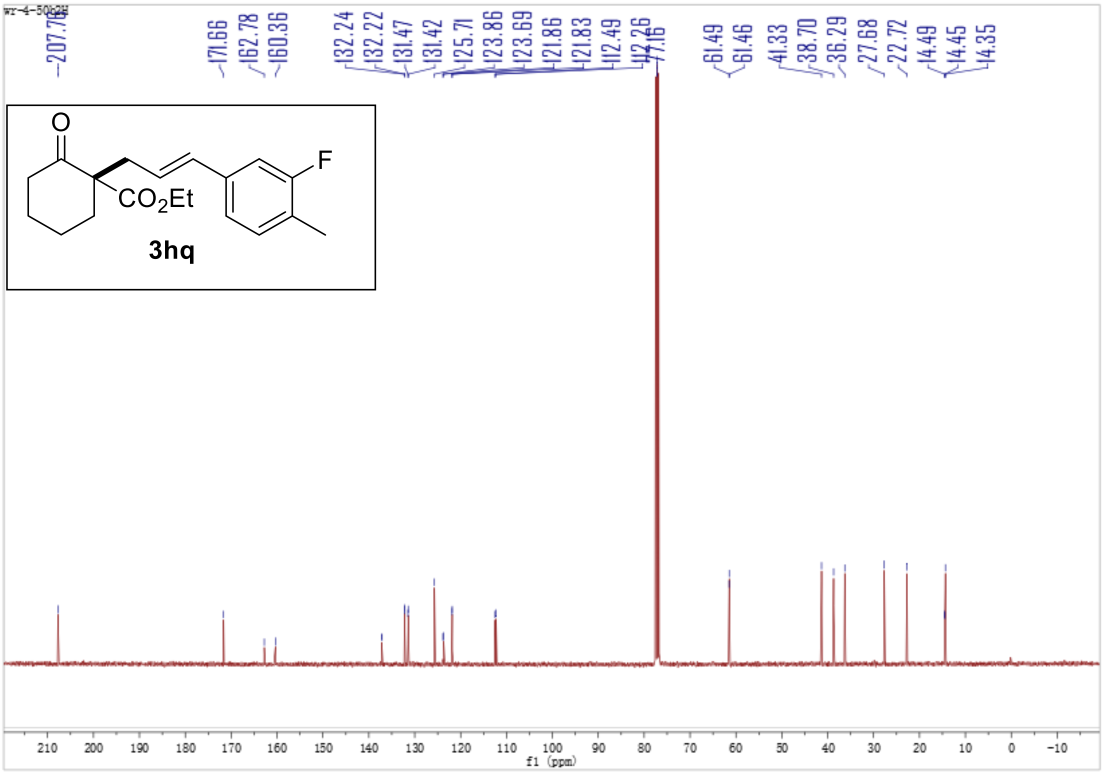

^{1}H NMR spectrum of compound **3hr** ($CDCl_3$, 400 MHz)

Links CATALOG DETAILS NMR

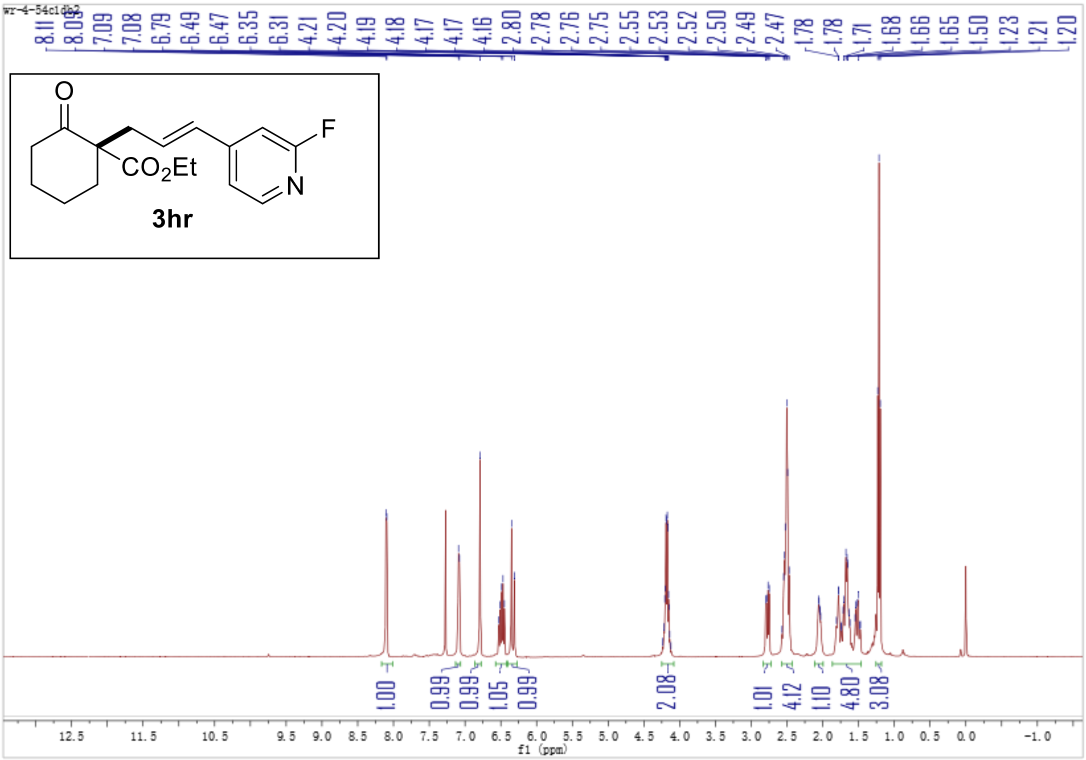

^{13}C NMR spectrum of compound **3hr** ($CDCl_3$, 101 MHz)

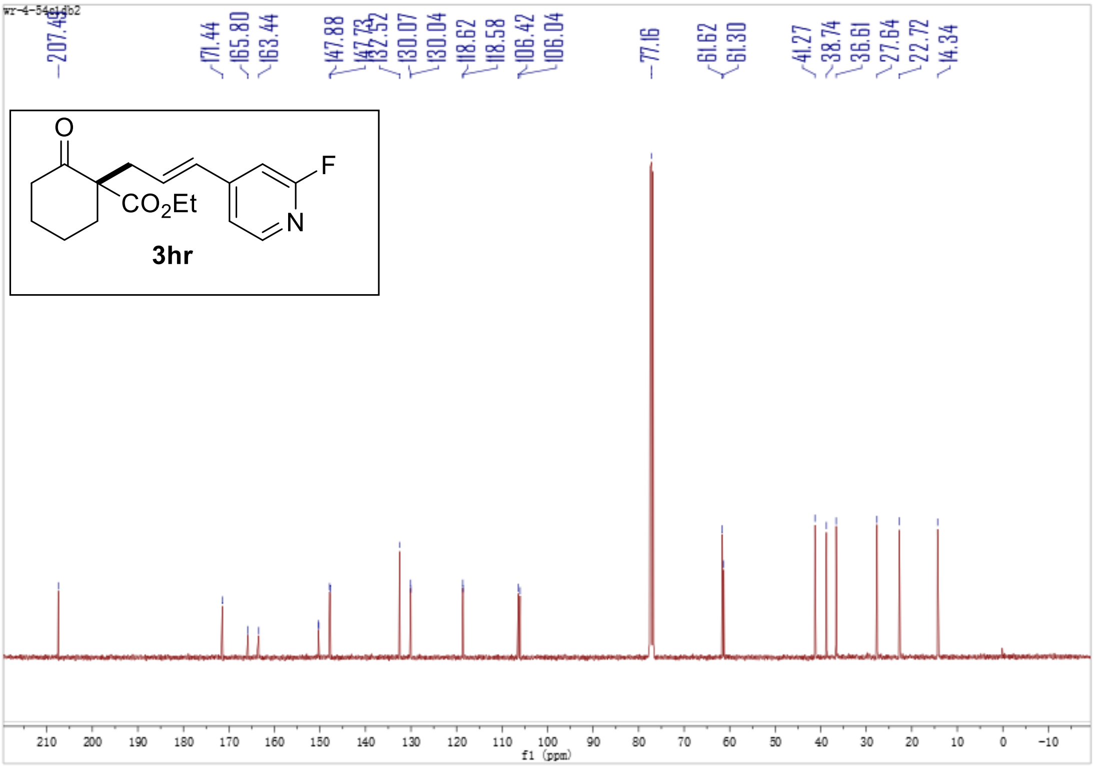

^{1}H **NMR** spectrum of compound **3hs** ($CDCl_3$, 400 MHz)

Links CATALOG DETAILS NMR

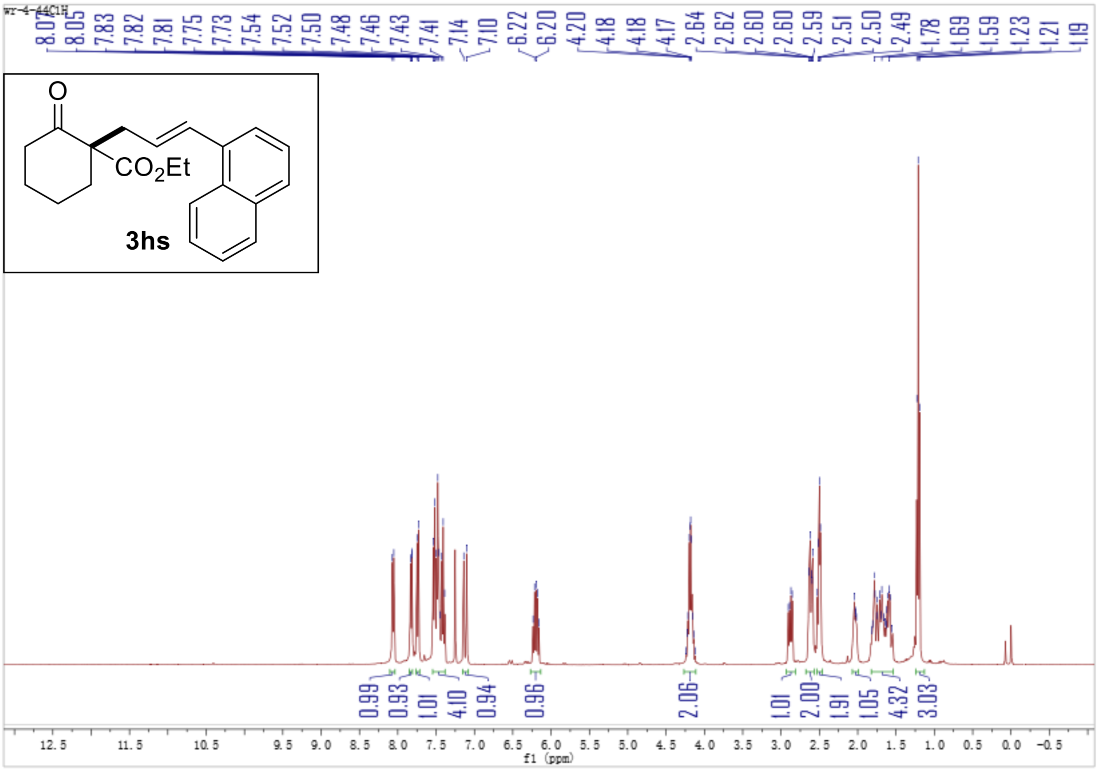

^{13}C **NMR** spectrum of compound **3hs** ($CDCl_3$, 101 MHz)

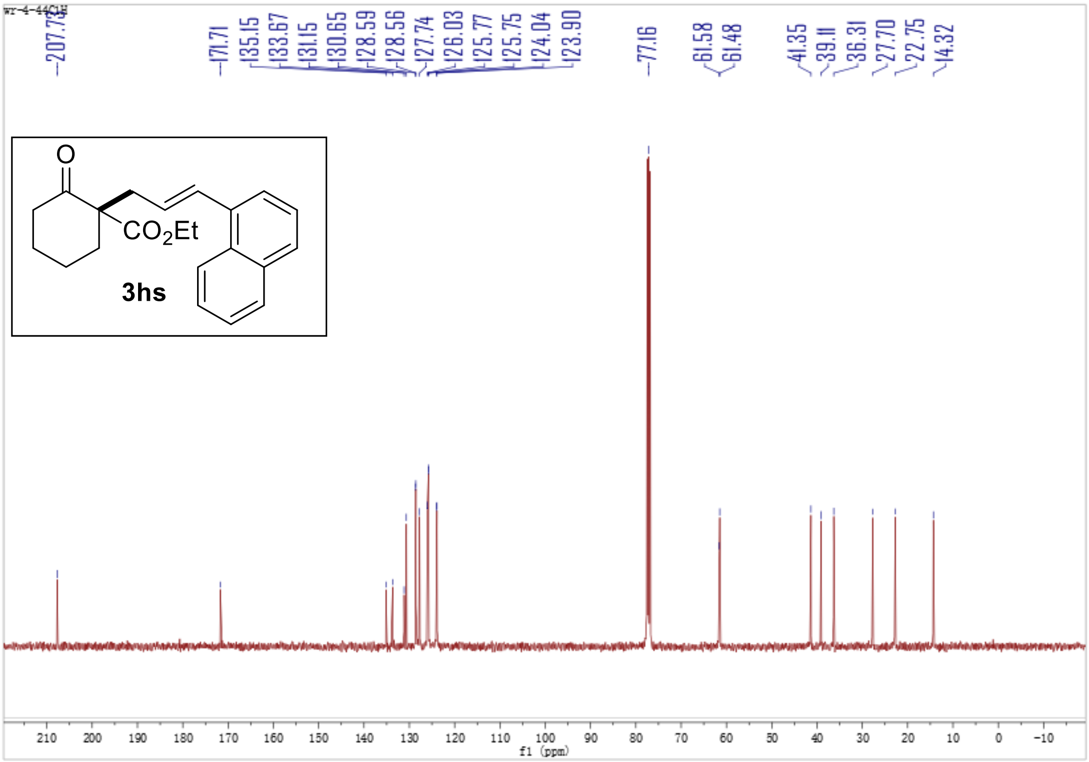

^{1}**H NMR** spectrum of compound **3ht** ($CDCl_3$, 400 MHz)

Links CATALOG DETAILS NMR

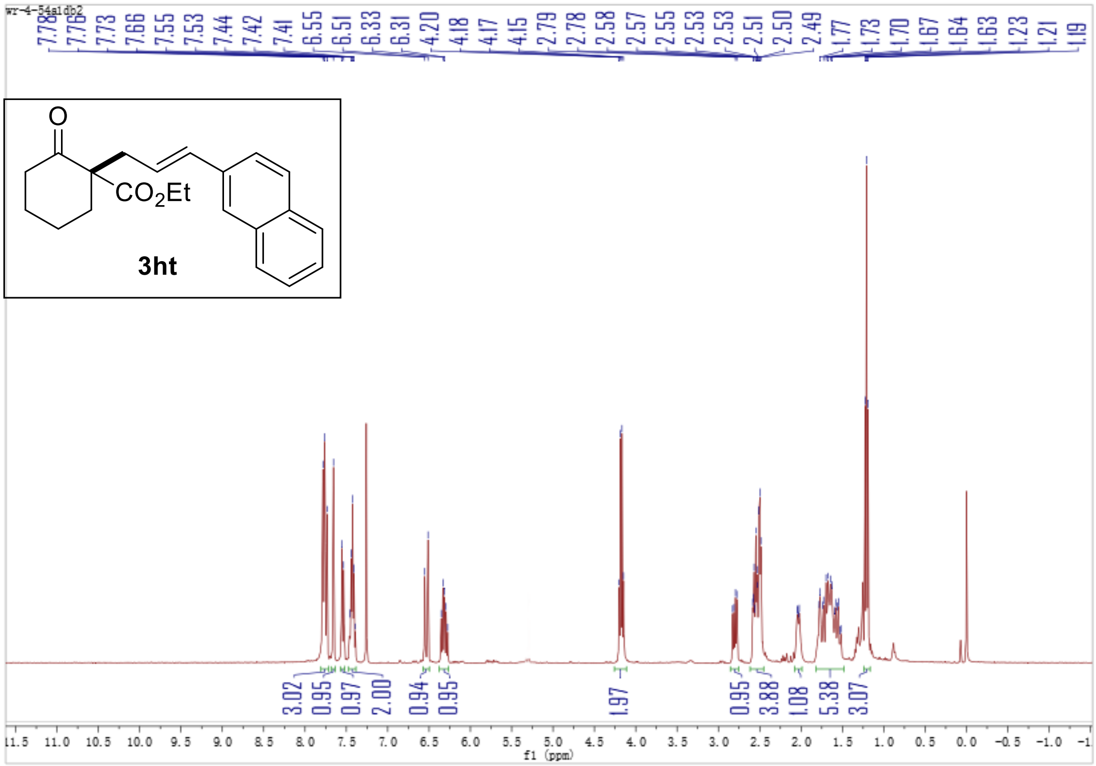

^{13}**C NMR** spectrum of compound **3ht** ($CDCl_3$, 101 MHz)

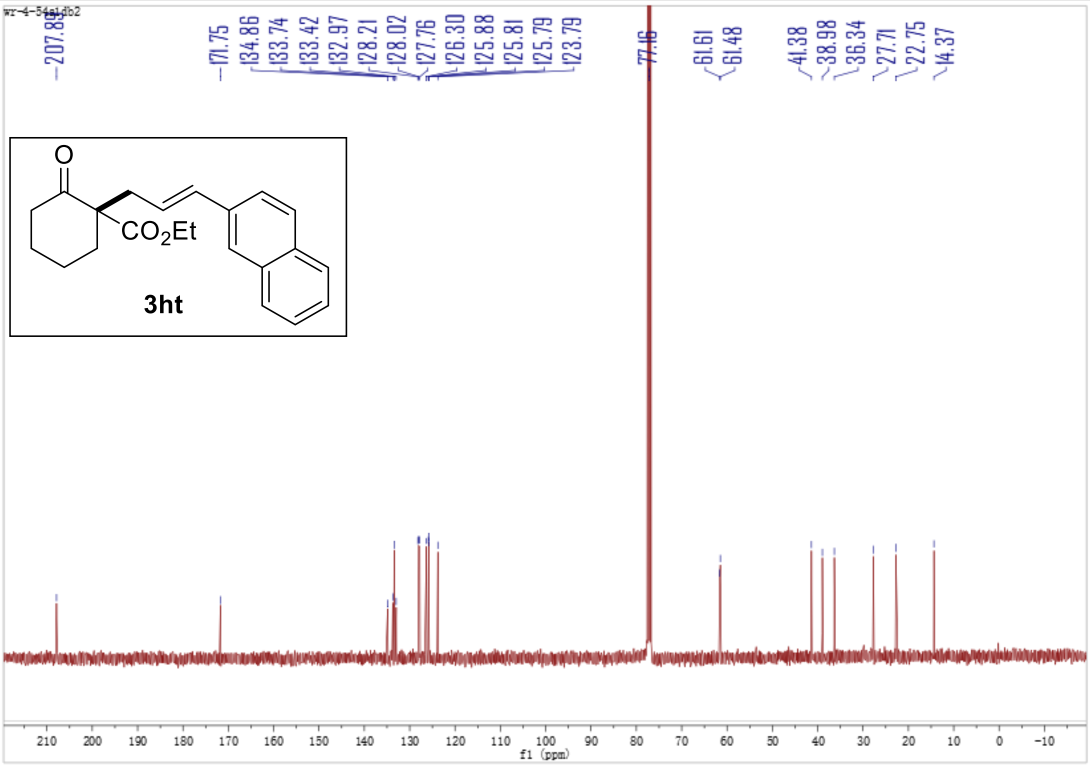

^{1}**H NMR** spectrum of compound **3hu** ($CDCl_3$, 400 MHz)

Links CATALOG DETAILS NMR

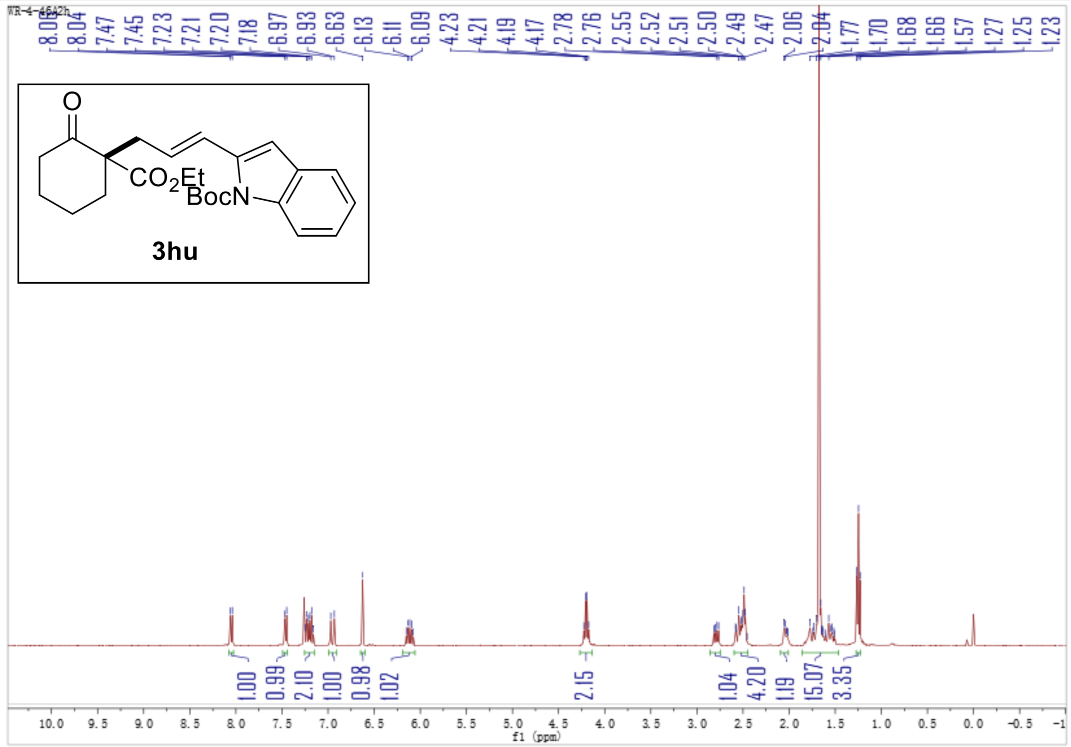

^{13}**C NMR** spectrum of compound **3hu** ($CDCl_3$, 101 MHz)

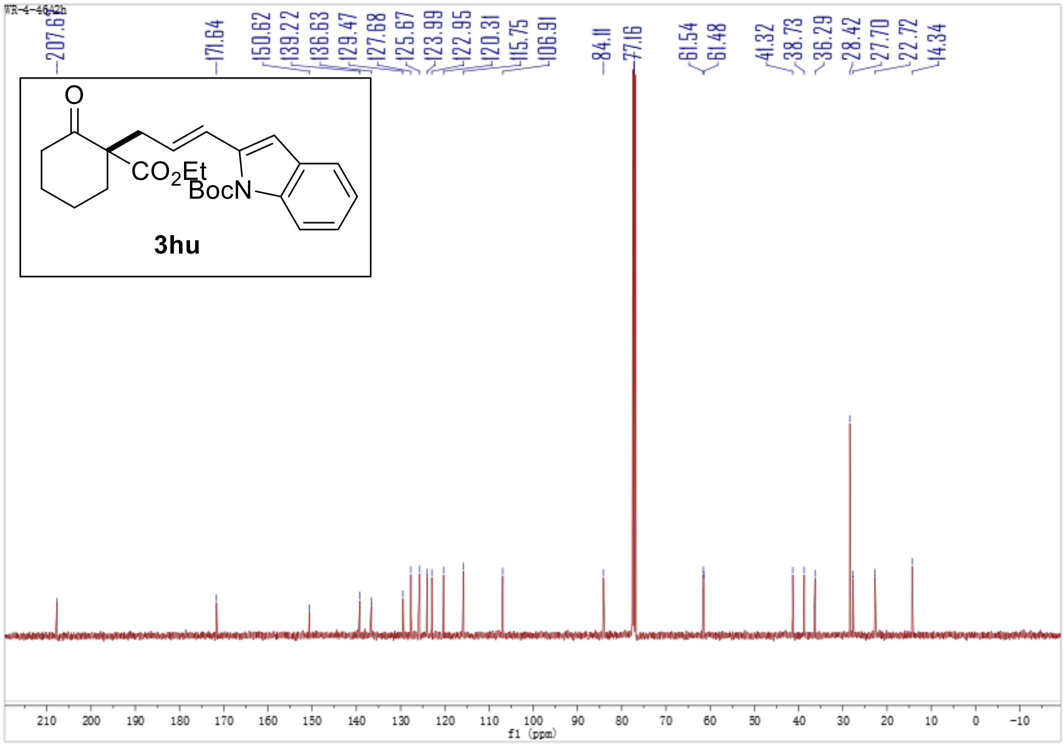

^{1}H NMR spectrum of compound **7aa** (CDCl$_3$, 400 MHz)

Links CATALOG DETAILS NMR

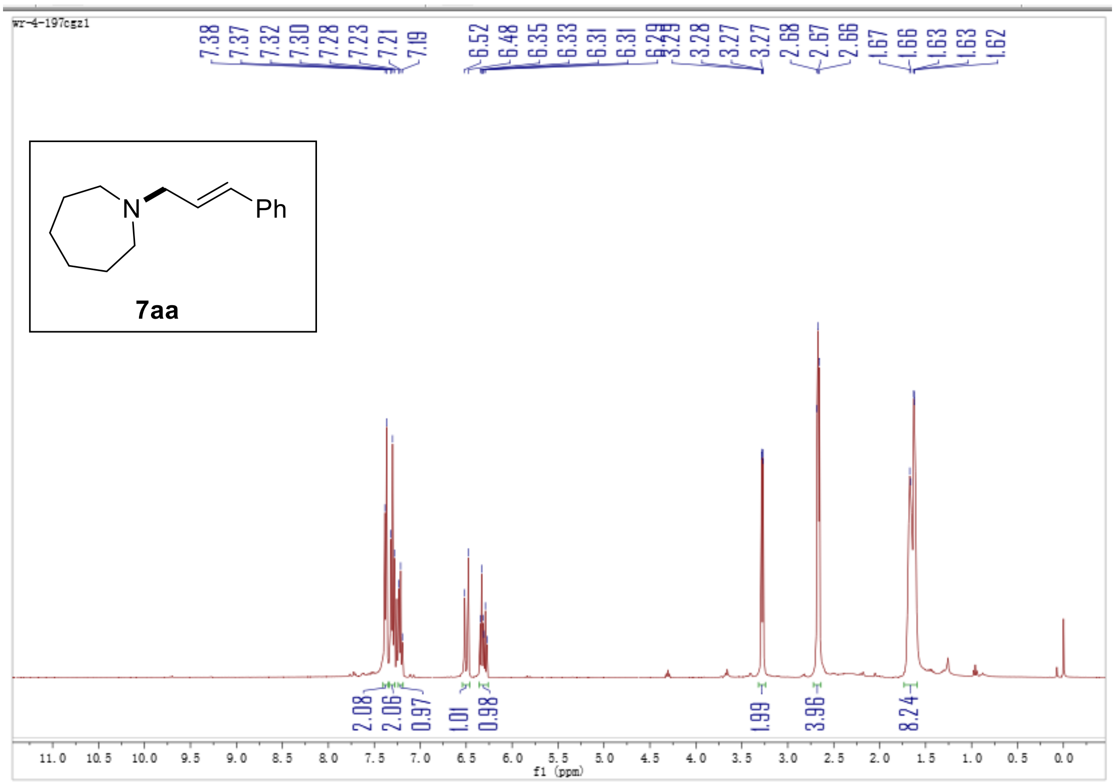

^{13}C NMR spectrum of compound **7aa** (CDCl$_3$, 101 MHz)

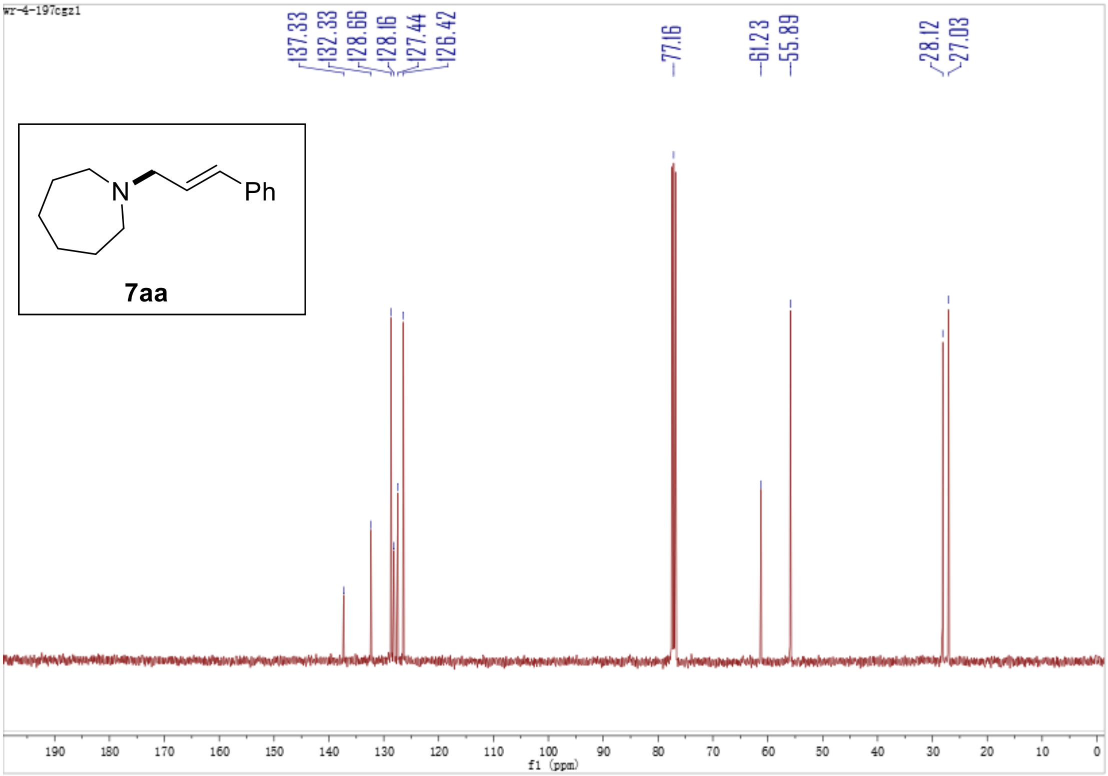

^{1}H NMR spectrum of compound **7ba** ($CDCl_3$, 400 MHz)

Links CATALOG DETAILS NMR

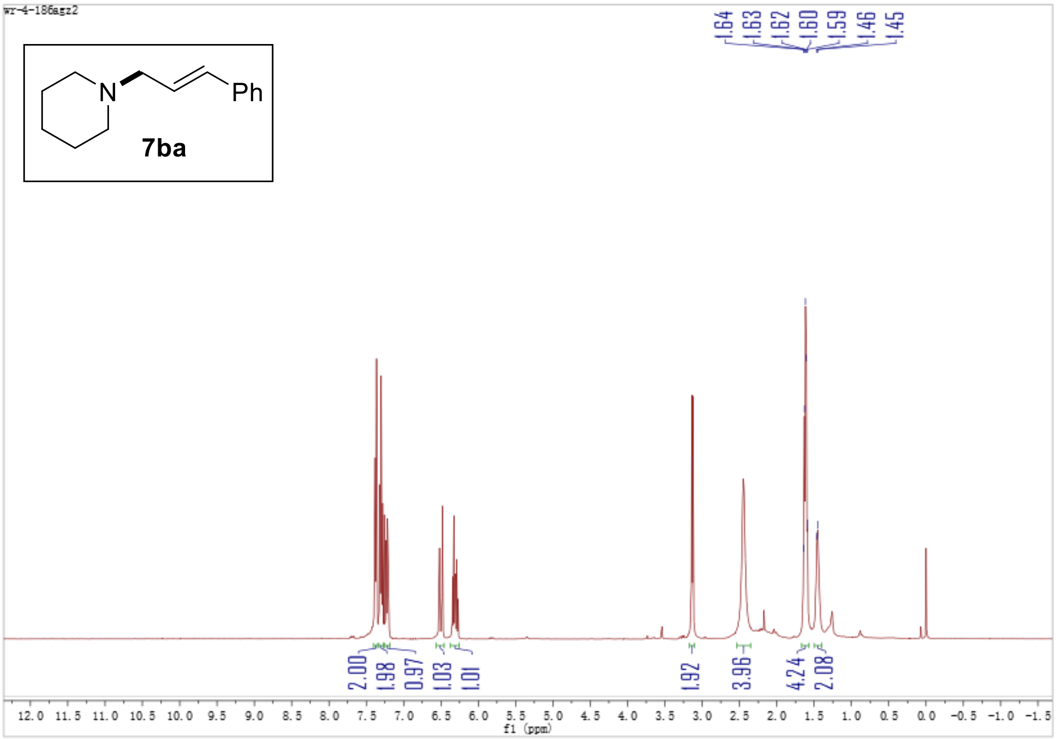

^{13}C NMR spectrum of compound **7ba** ($CDCl_3$, 101 MHz)

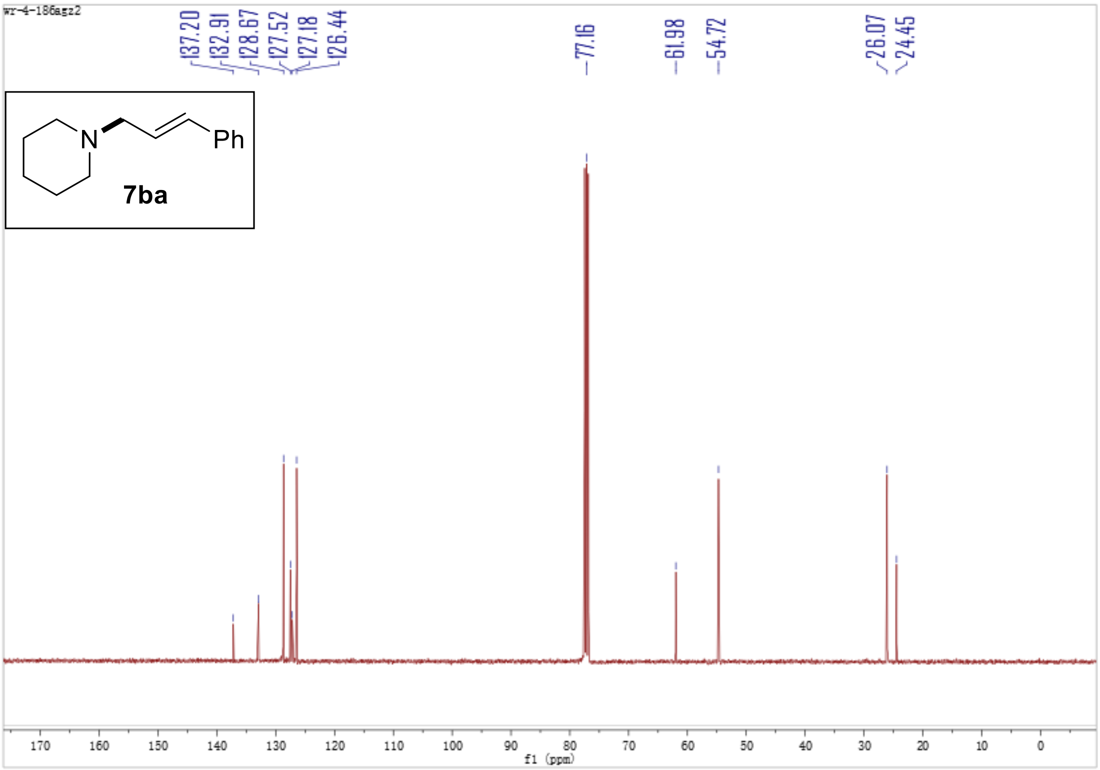

^{1}**H NMR** spectrum of compound **7ca** ($CDCl_3$, 400 MHz)

Links CATALOG DETAILS NMR

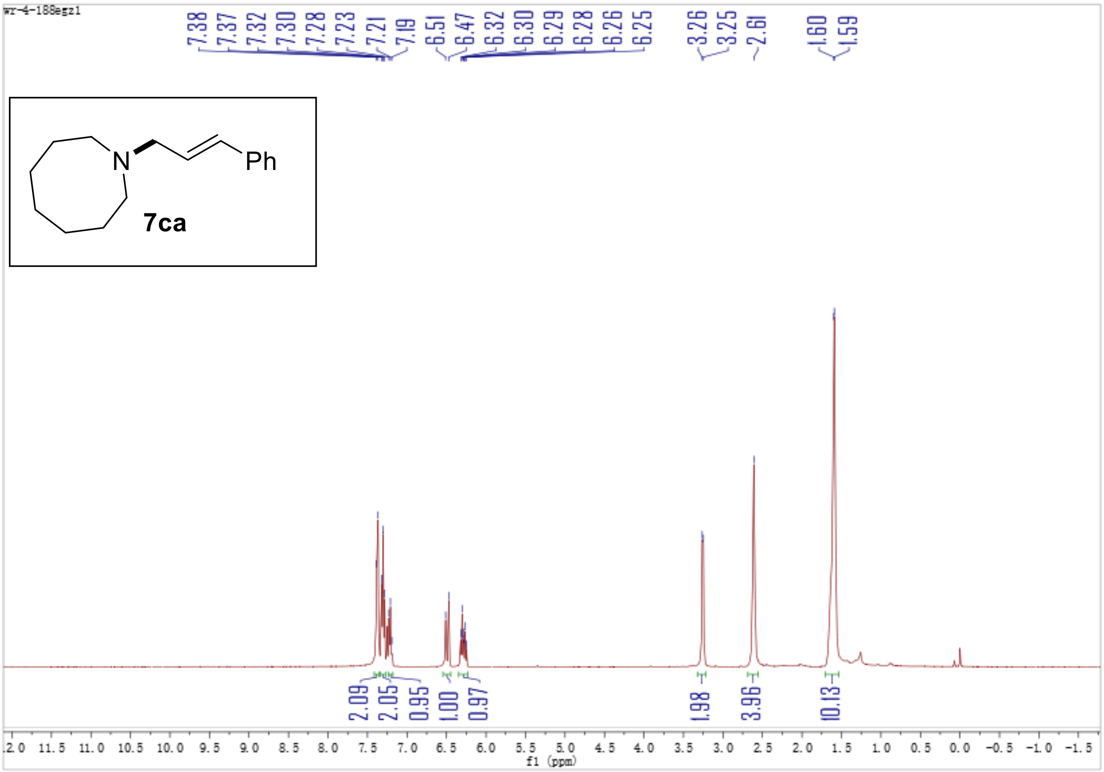

^{13}**C NMR** spectrum of compound **7ca** ($CDCl_3$, 101 MHz)

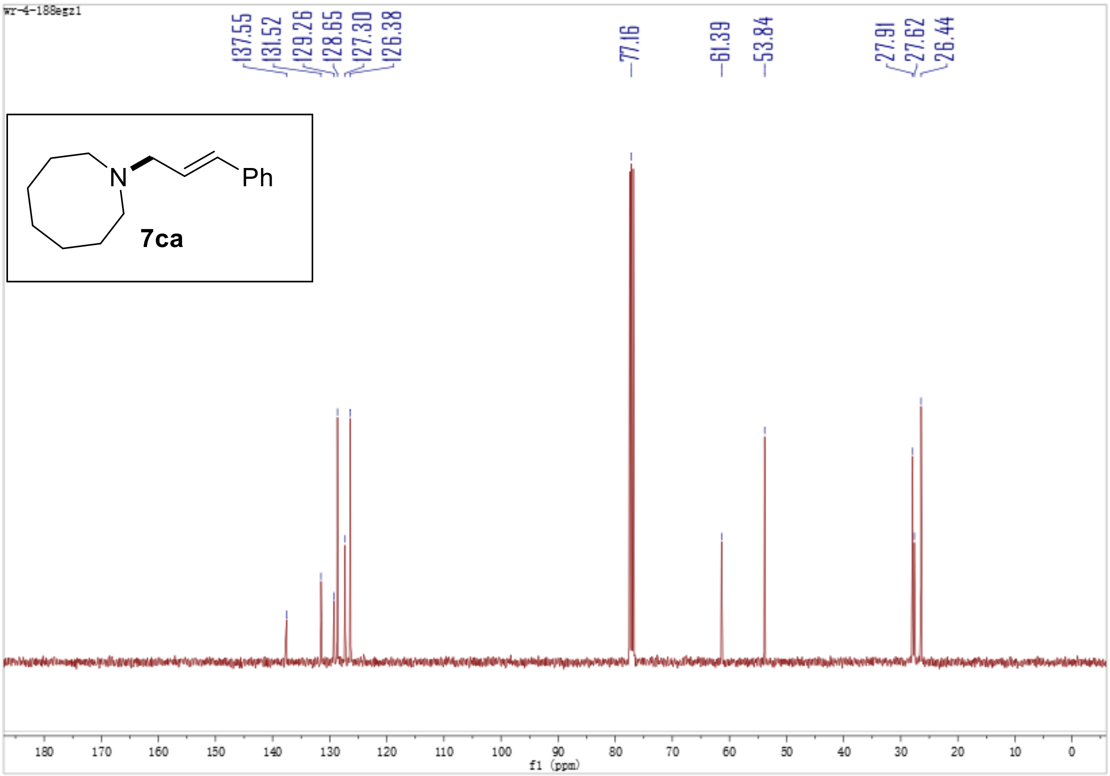

^{1}H NMR spectrum of compound **7da** ($CDCl_3$, 400 MHz)

Links CATALOG DETAILS NMR

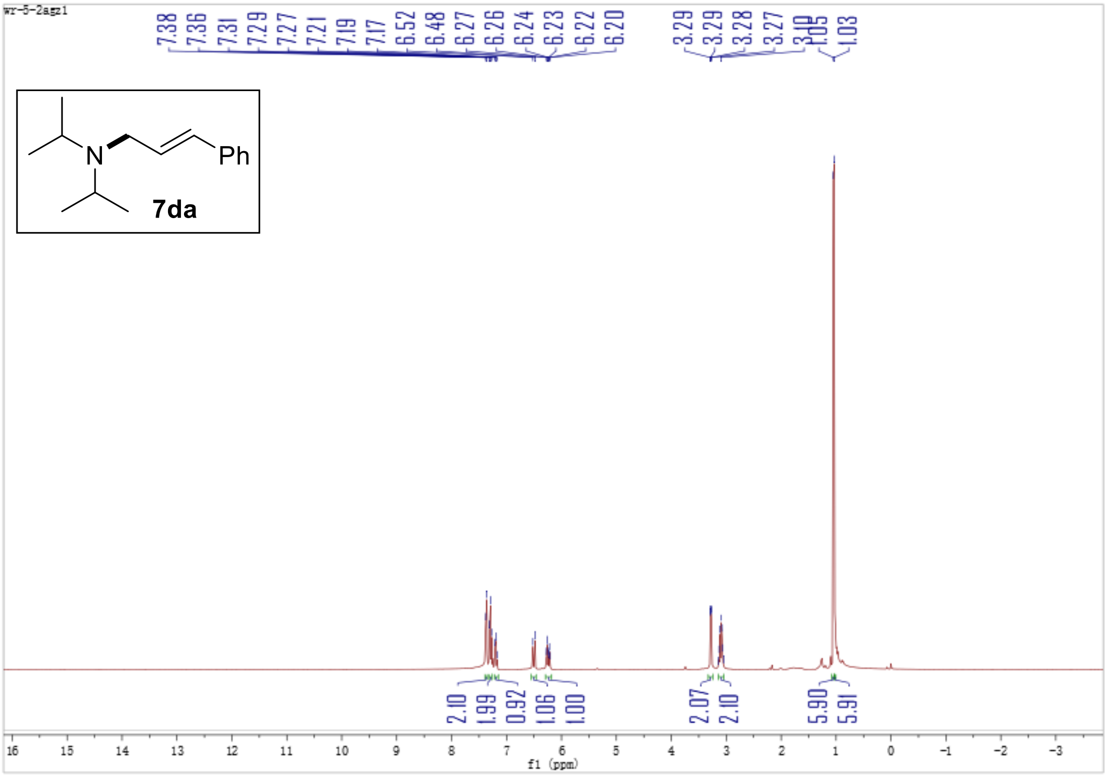

^{13}C NMR spectrum of compound **7da** ($CDCl_3$, 101 MHz)

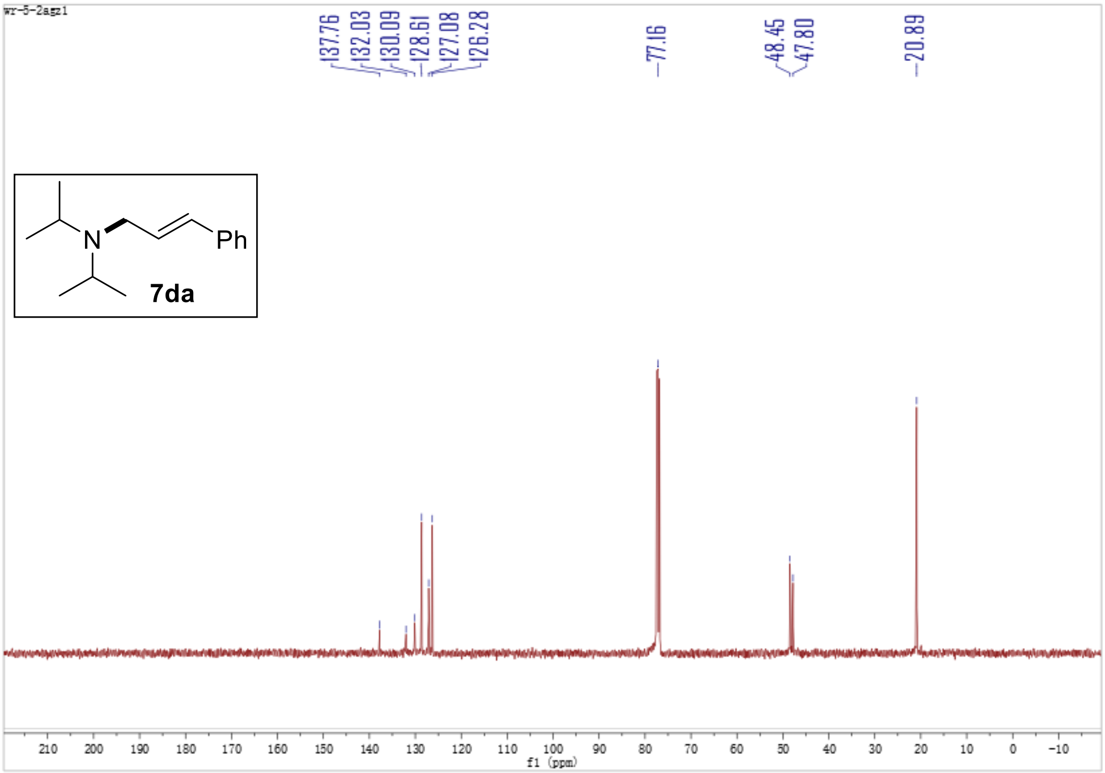

^{1}**H NMR** spectrum of compound **7ea** ($CDCl_3$, 400 MHz)

Links CATALOG DETAILS NMR

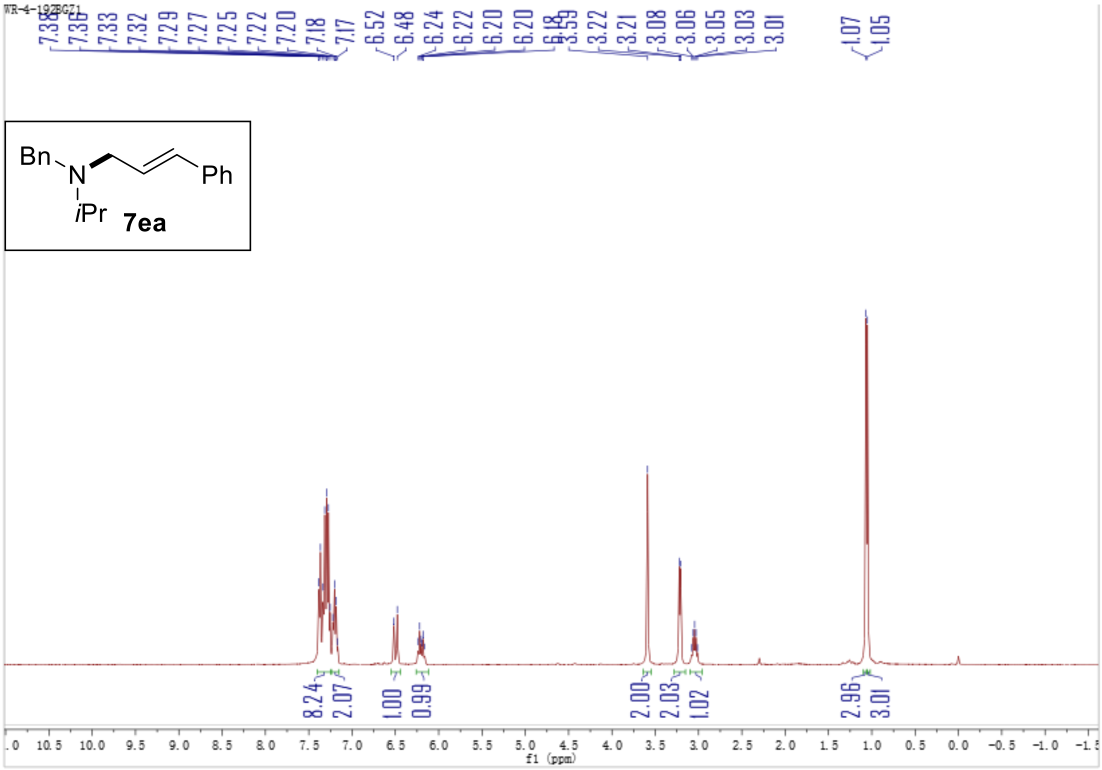

^{13}**C NMR** spectrum of compound **7ea** ($CDCl_3$, 101 MHz)

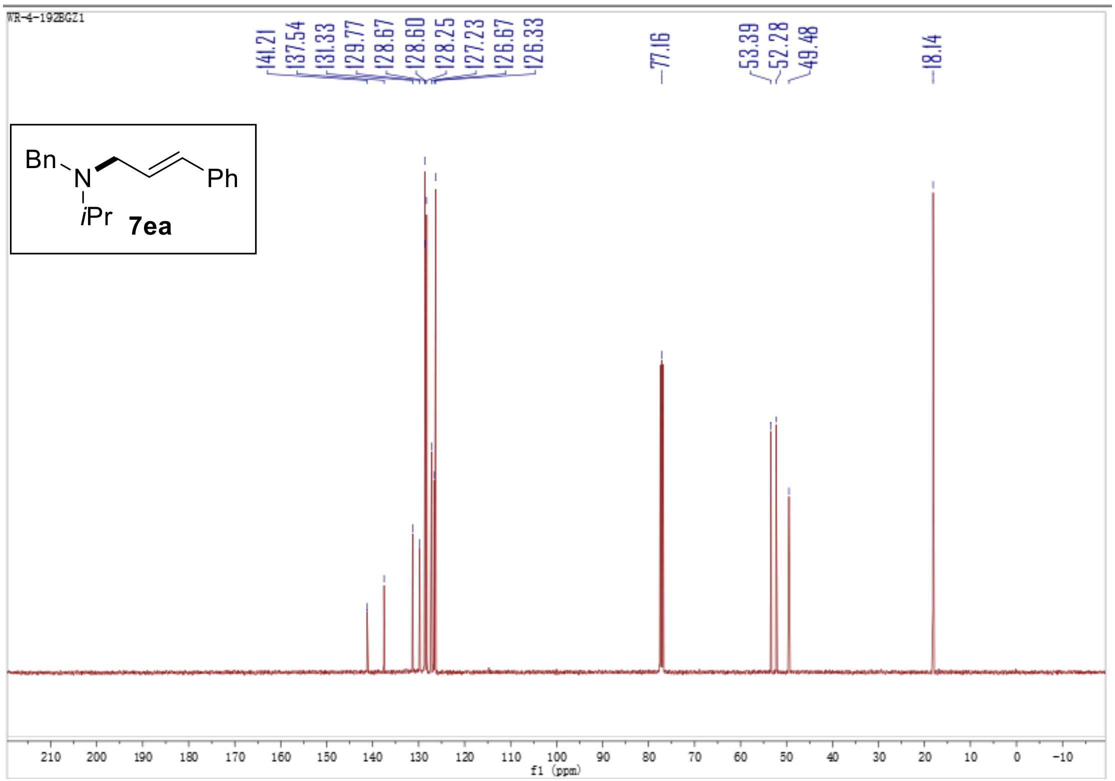

^{1}H NMR spectrum of compound **7fa** ($CDCl_3$, 400 MHz)

Links CATALOG DETAILS NMR

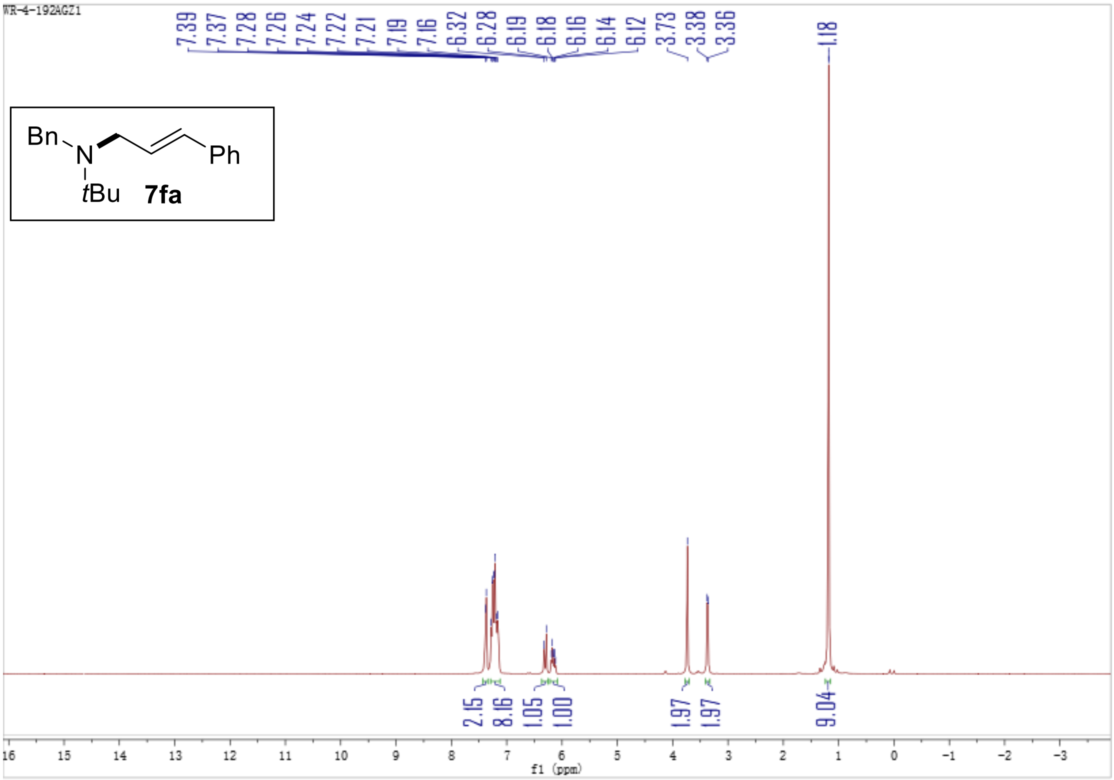

^{13}C NMR spectrum of compound **7fa** ($CDCl_3$, 101 MHz)

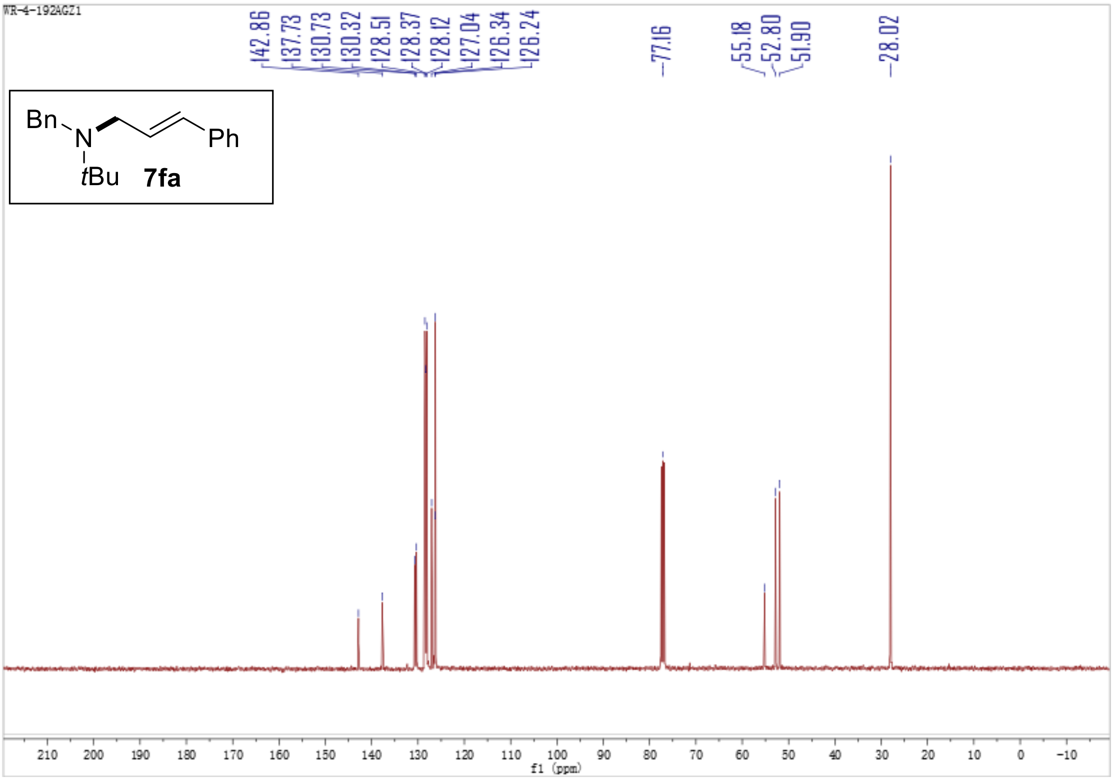

^{1}H NMR spectrum of compound **7ga** ($CDCl_3$, 400 MHz)

Links CATALOG DETAILS NMR

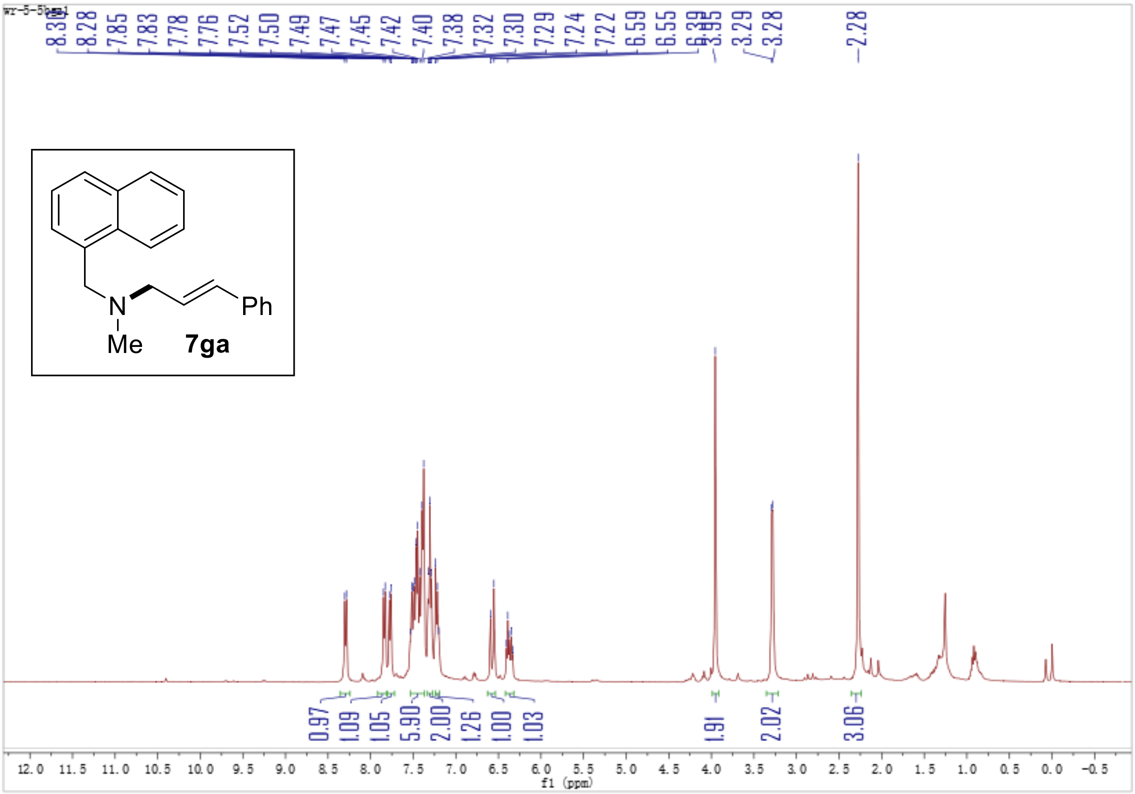

^{13}C NMR spectrum of compound **7ga** ($CDCl_3$, 101 MHz)

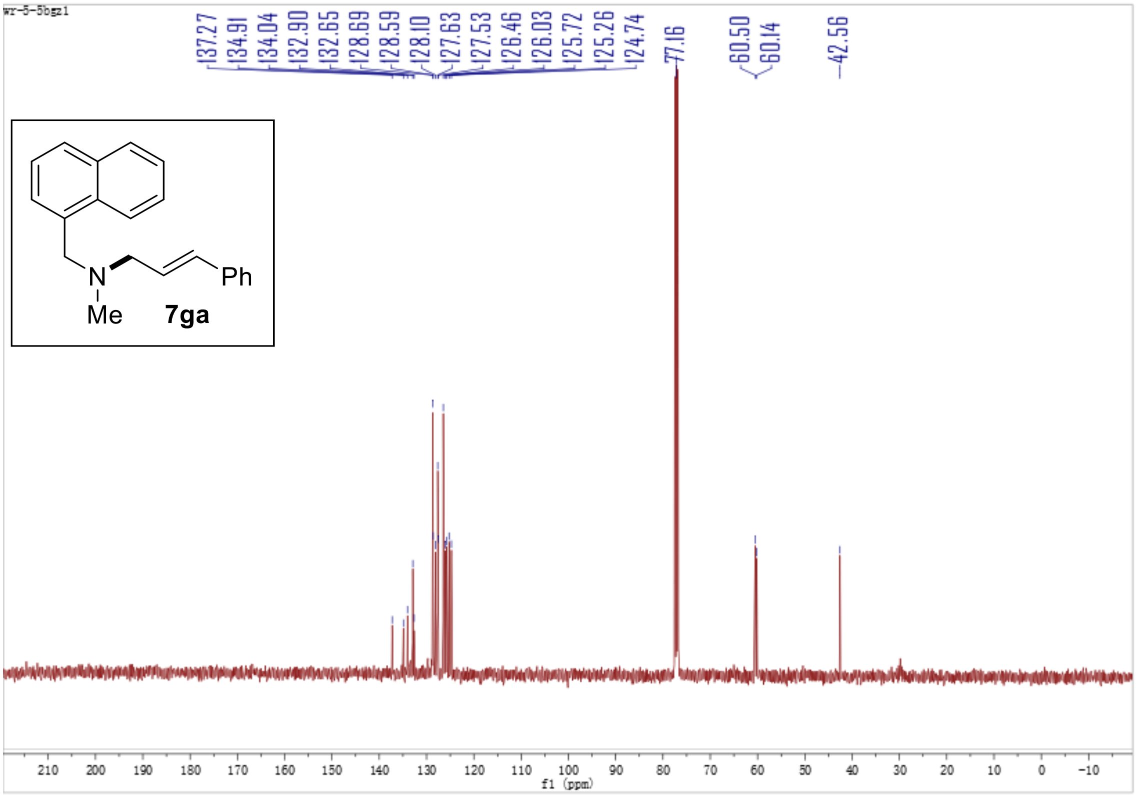

^{1}**H NMR** spectrum of compound **7ha** ($CDCl_3$, 400 MHz)

Links CATALOG DETAILS NMR E-Fact

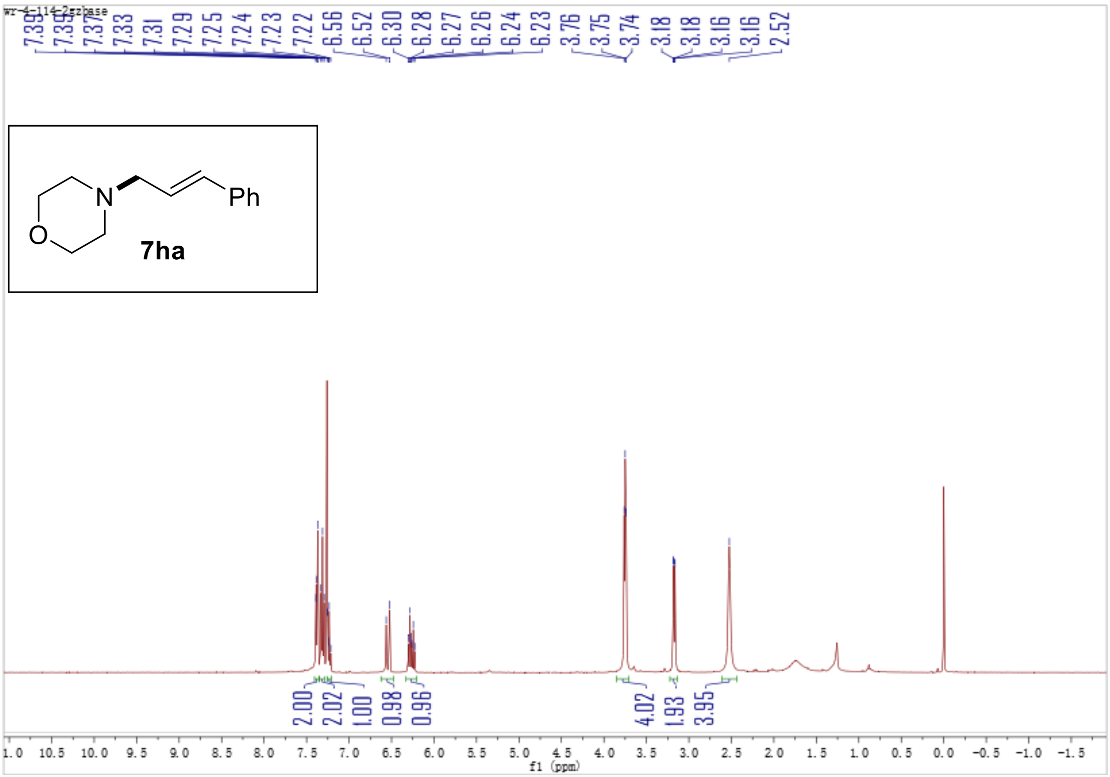

^{13}**C NMR** spectrum of compound **7ha** ($CDCl_3$, 101 MHz)

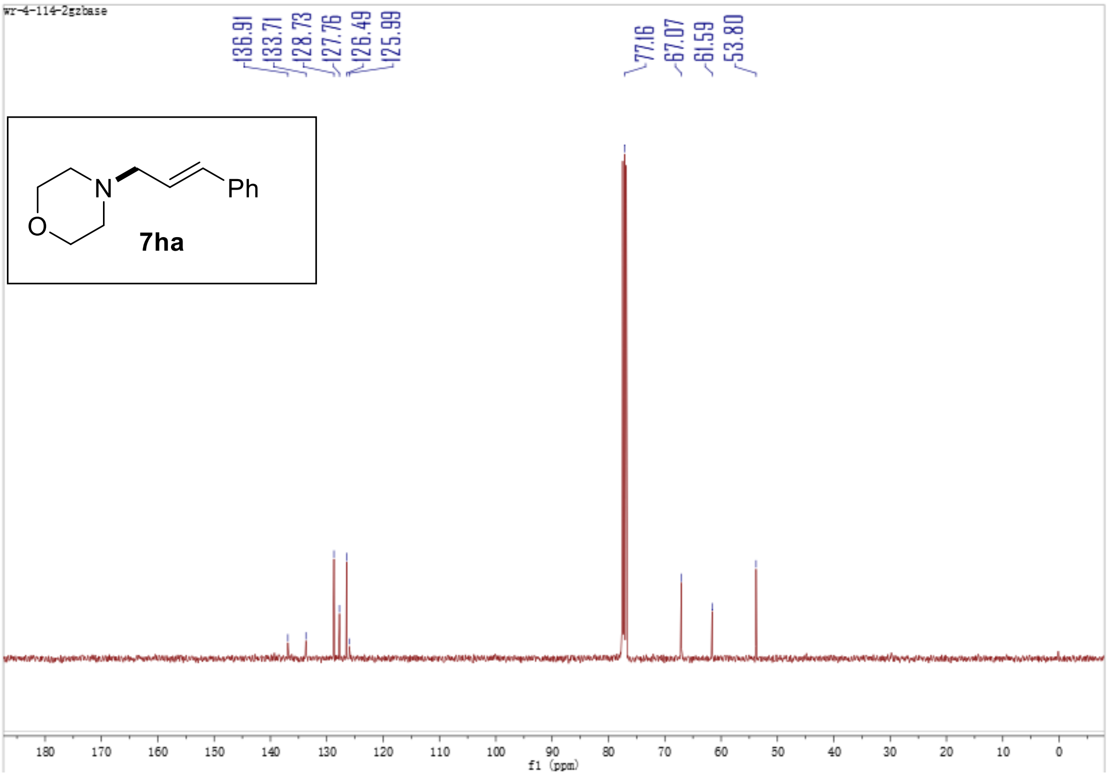

^{1}H NMR spectrum of compound 7ia ($CDCl_3$, 400 MHz)

Links CATALOG DETAILS NMR

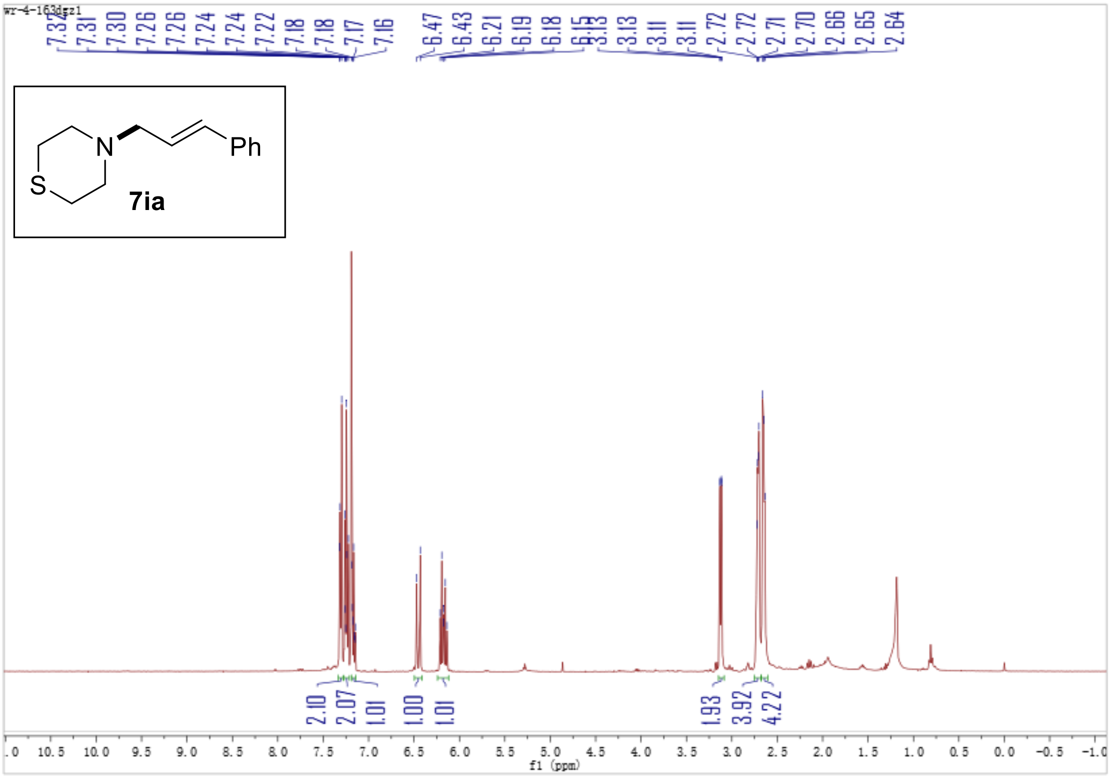

^{13}C NMR spectrum of compound 7ia ($CDCl_3$, 101 MHz)

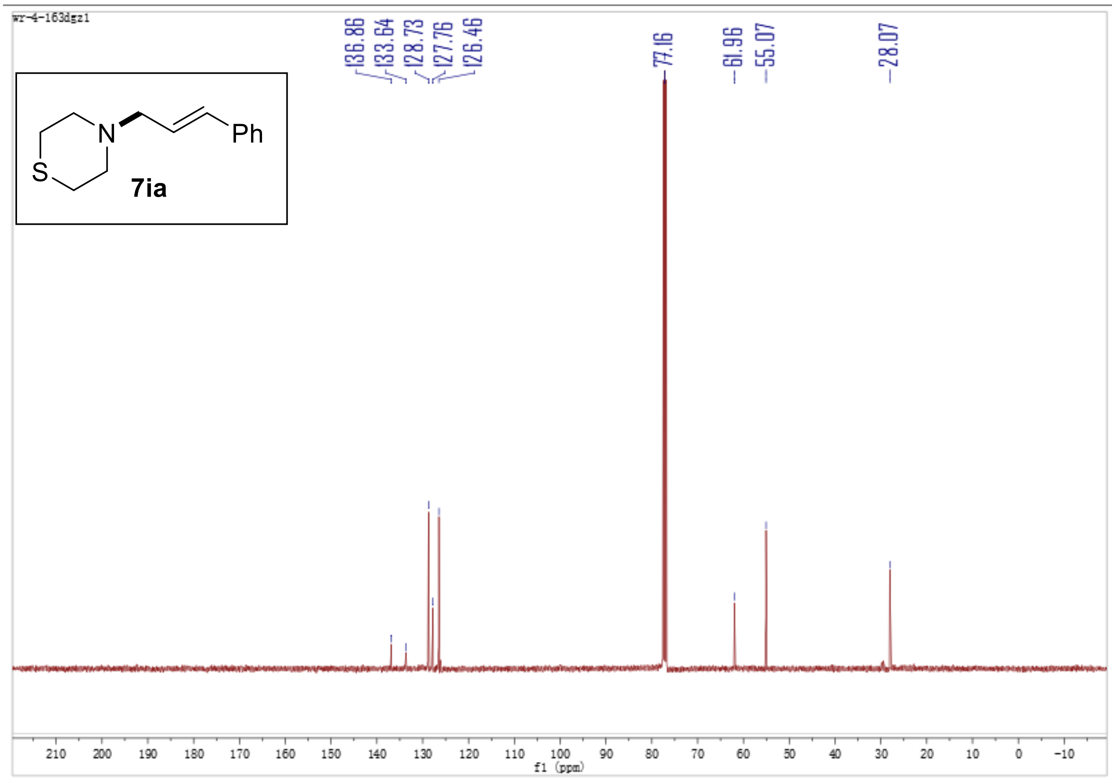

^{1}H NMR spectrum of compound **7ja** ($CDCl_3$, 400 MHz)

Links CATALOG DETAILS NMR

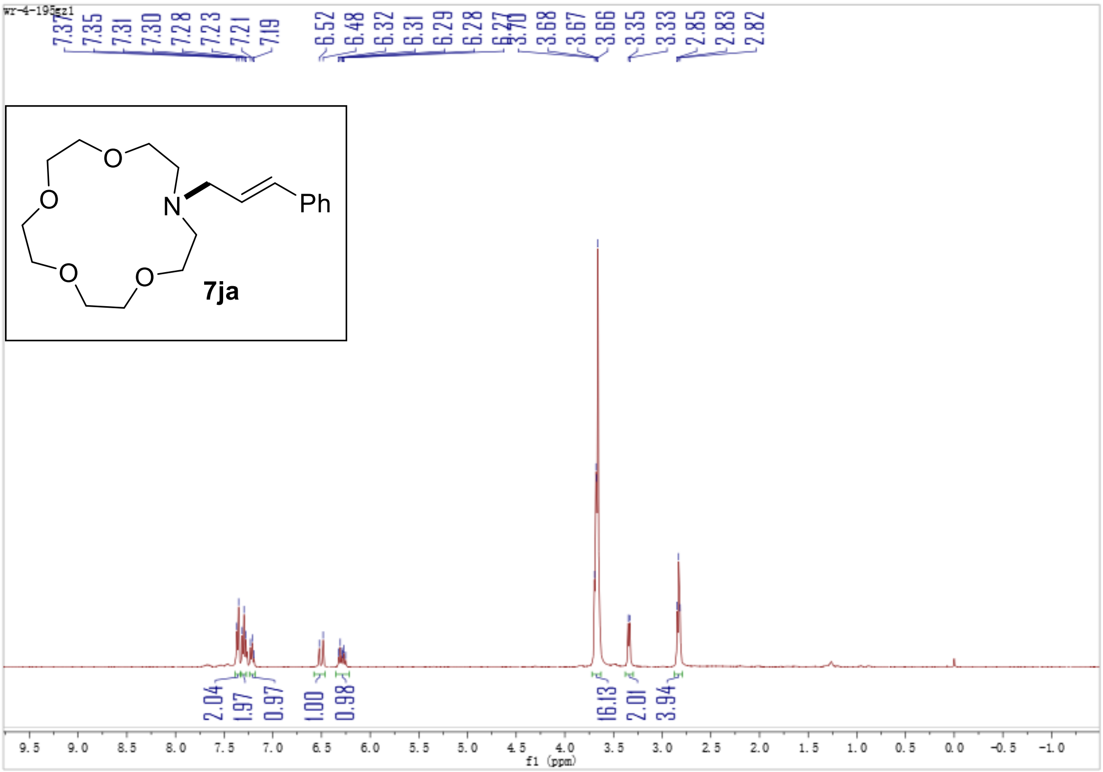

^{13}C NMR spectrum of compound **7ja** ($CDCl_3$, 101 MHz)

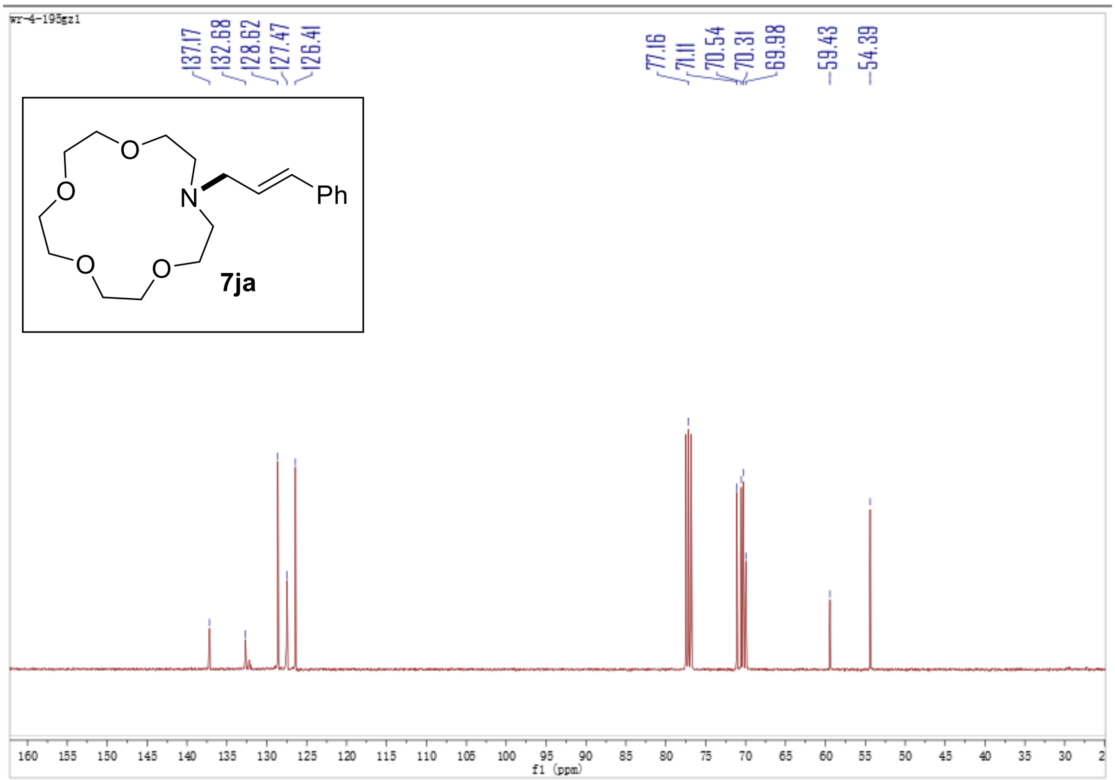

^{1}H NMR spectrum of compound **7ka** ($CDCl_3$, 400 MHz)

Links CATALOG DETAILS NMR

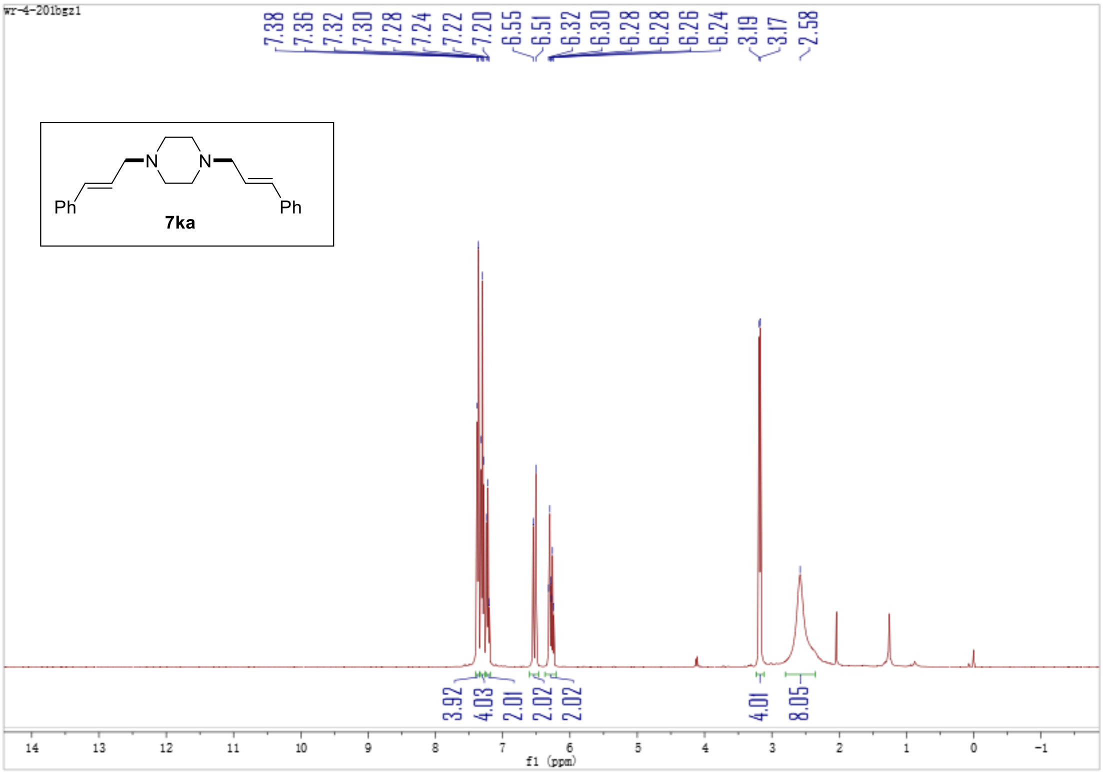

^{13}C NMR spectrum of compound **7ka** ($CDCl_3$, 101 MHz)

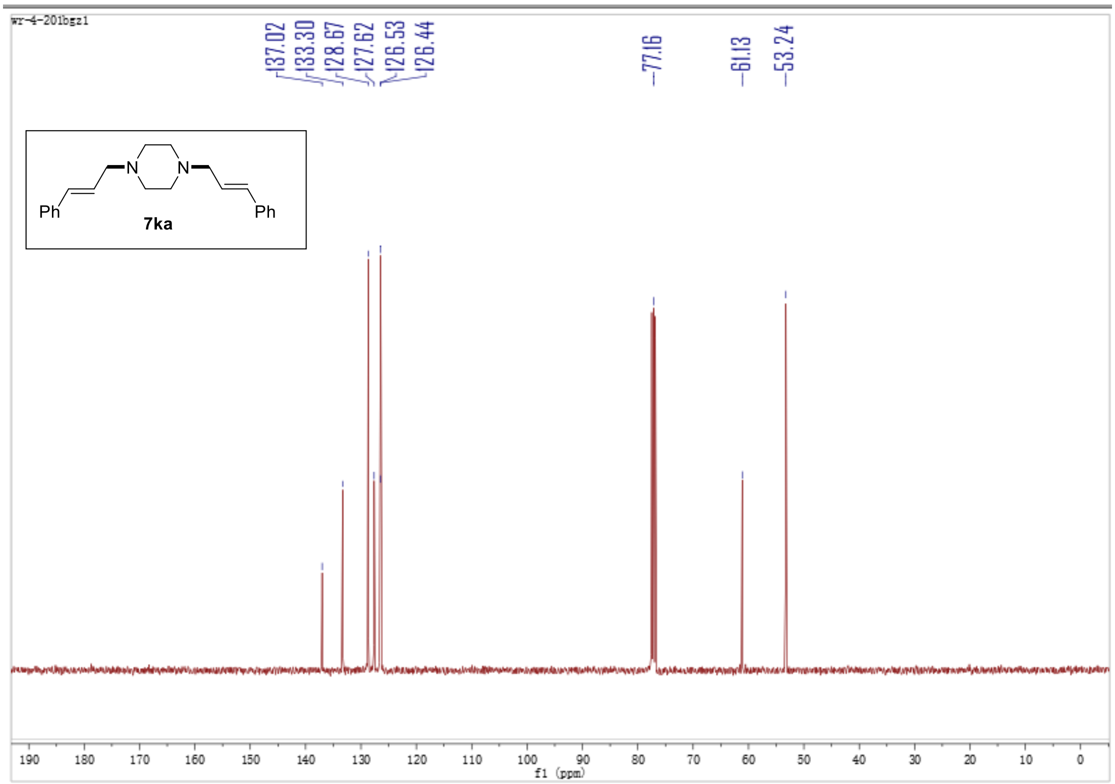

^{1}H NMR spectrum of compound **7la** ($CDCl_3$, 400 MHz)

Links CATALOG DETAILS NMR

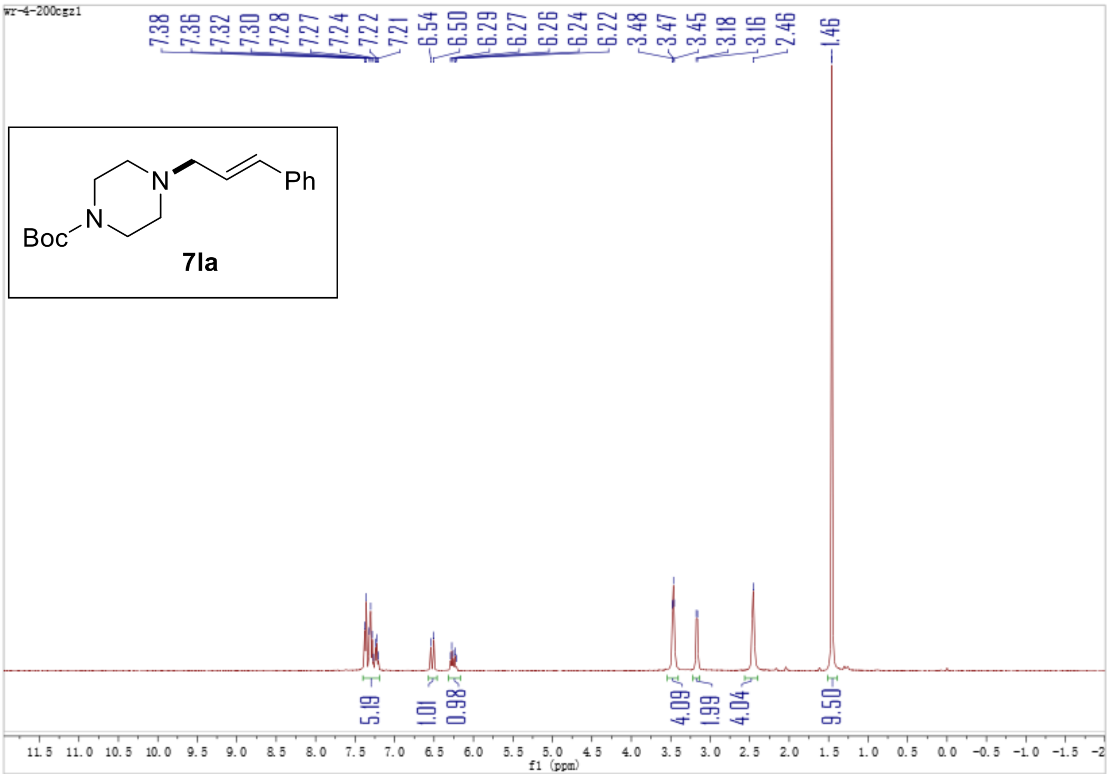

^{13}C NMR spectrum of compound **7la** ($CDCl_3$, 101 MHz)

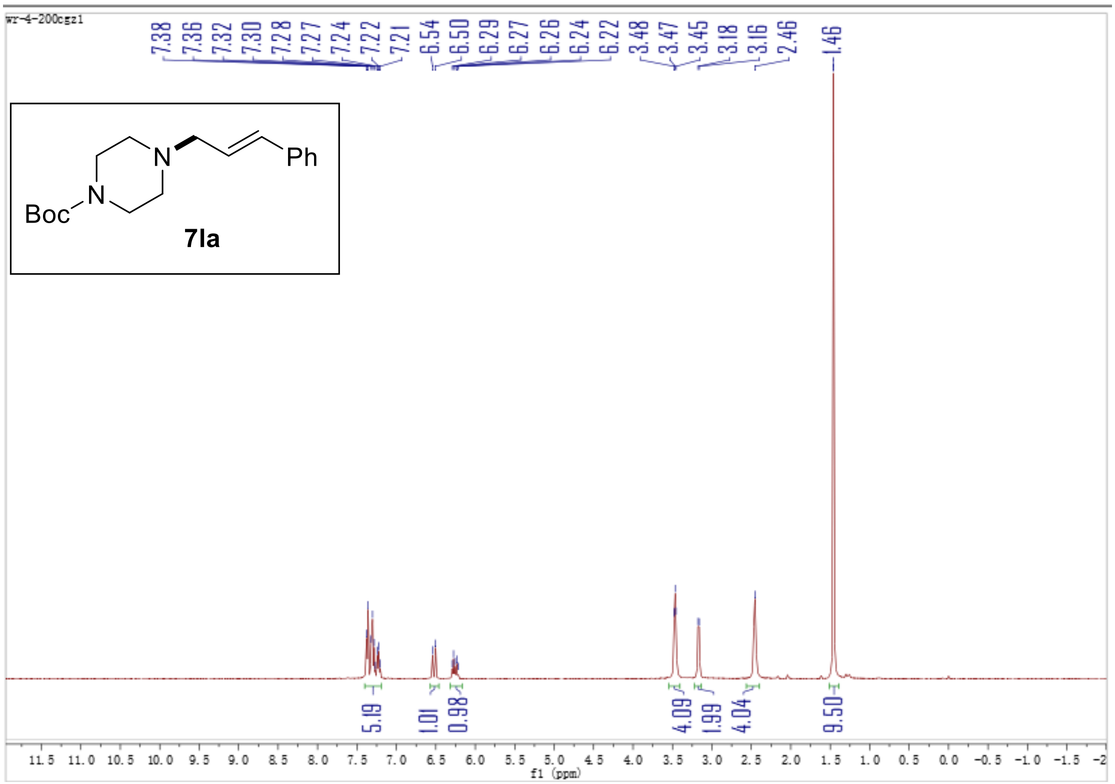

^{1}H NMR spectrum of compound **7ma** ($CDCl_3$, 400 MHz)

Links CATALOG DETAILS NMR

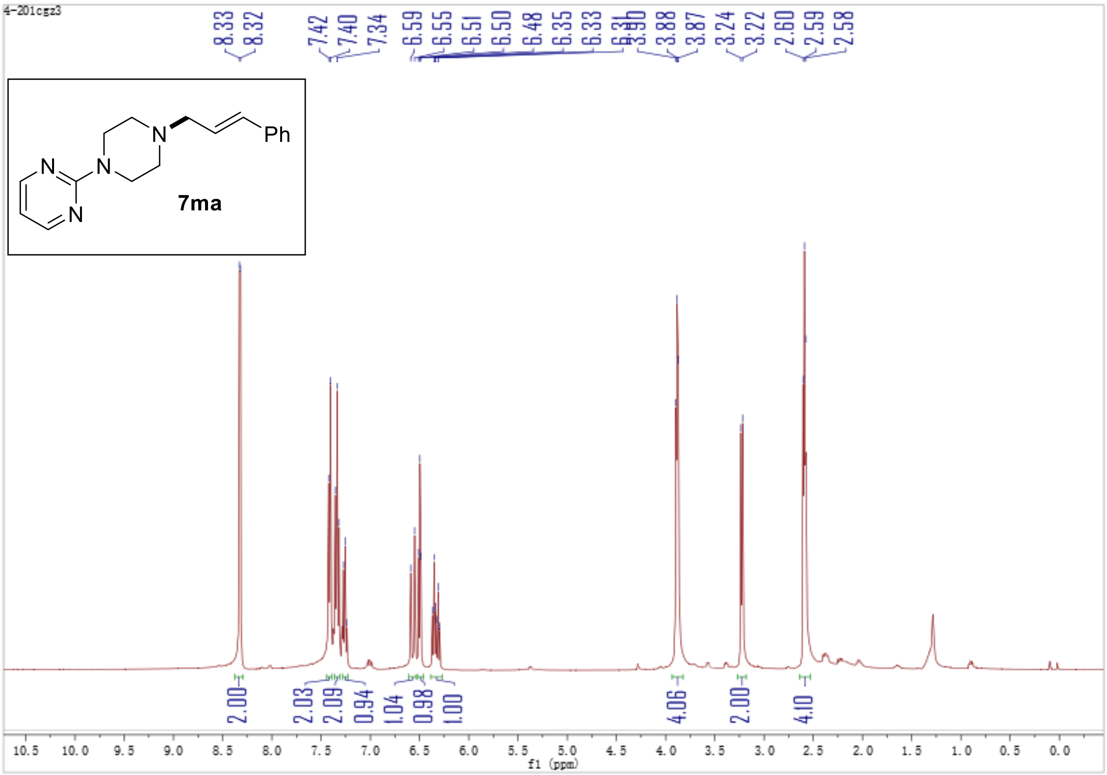

^{13}C NMR spectrum of compound **7ma** ($CDCl_3$, 101 MHz)

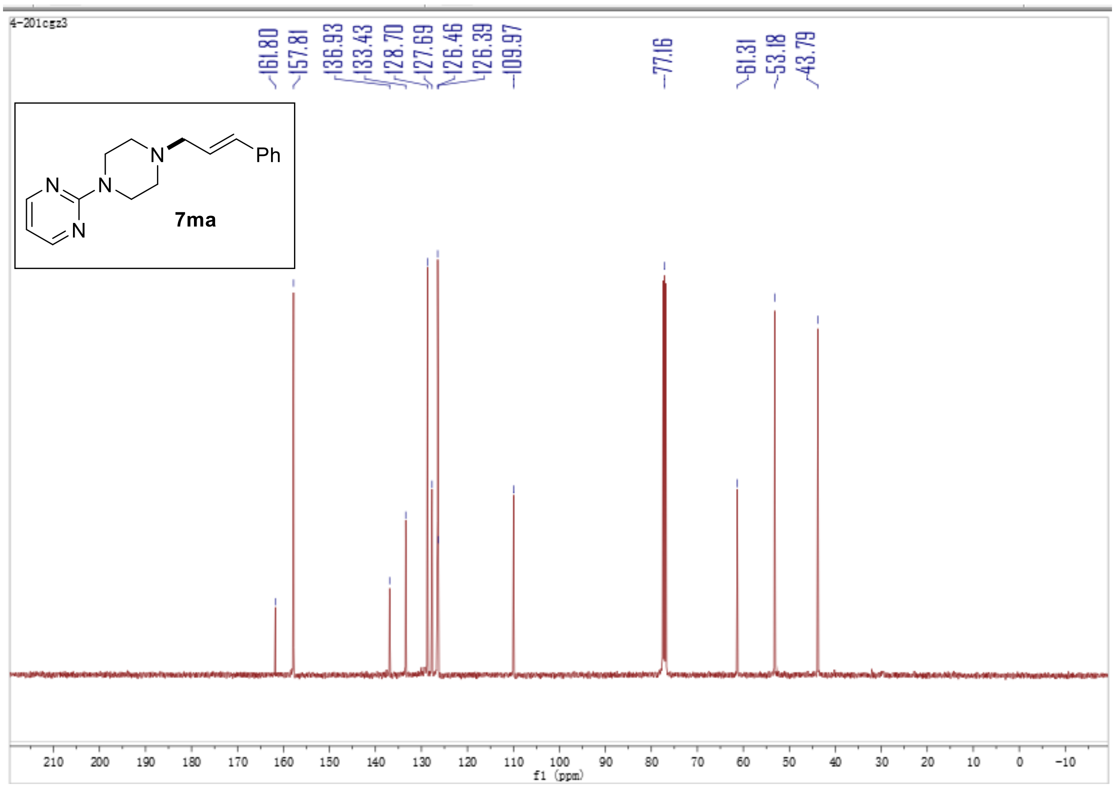

^{1}**H NMR** spectrum of compound **7na** ($CDCl_3$, 400 MHz)

Links CATALOG DETAILS NMR

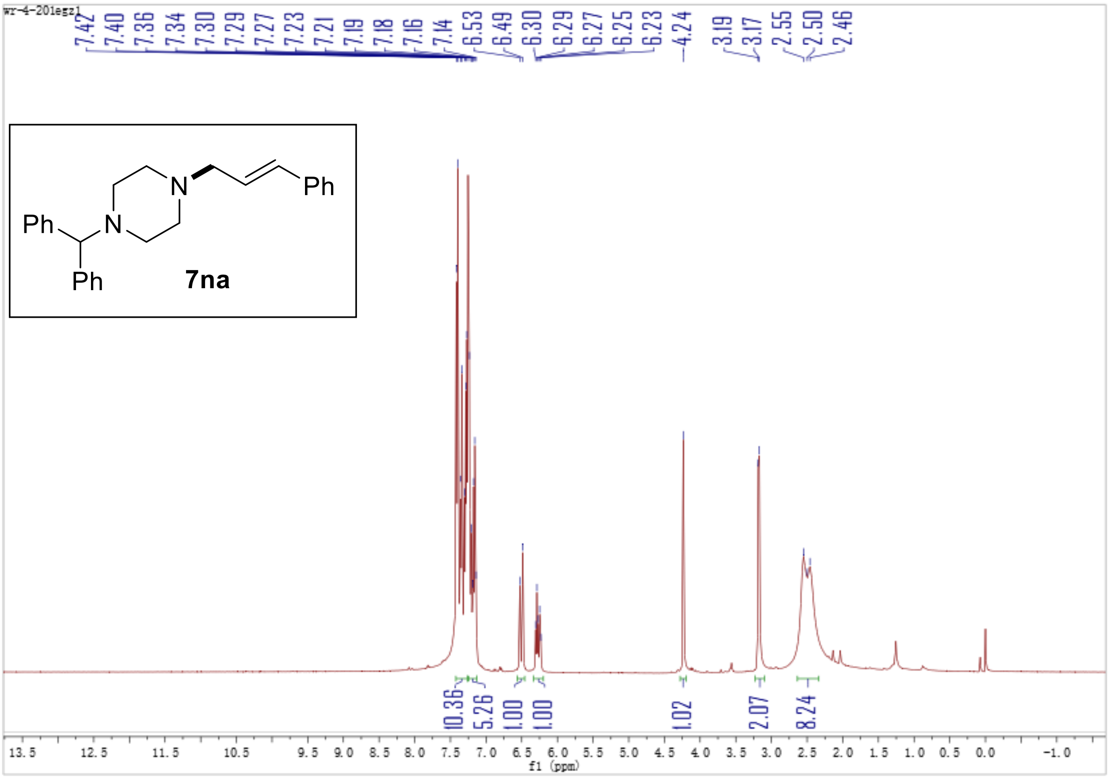

^{13}**C NMR** spectrum of compound **7na** ($CDCl_3$, 101 MHz)

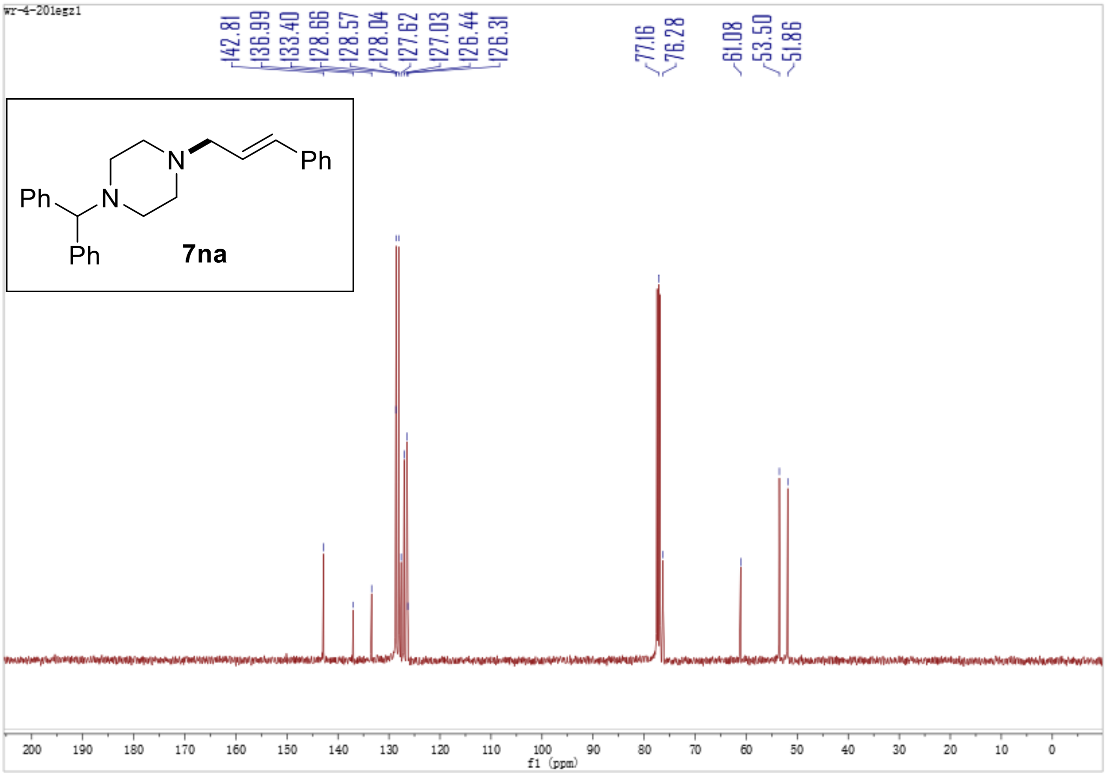

^{1}H NMR spectrum of compound **7oa** ($CDCl_3$, 400 MHz)

Links CATALOG DETAILS NMR

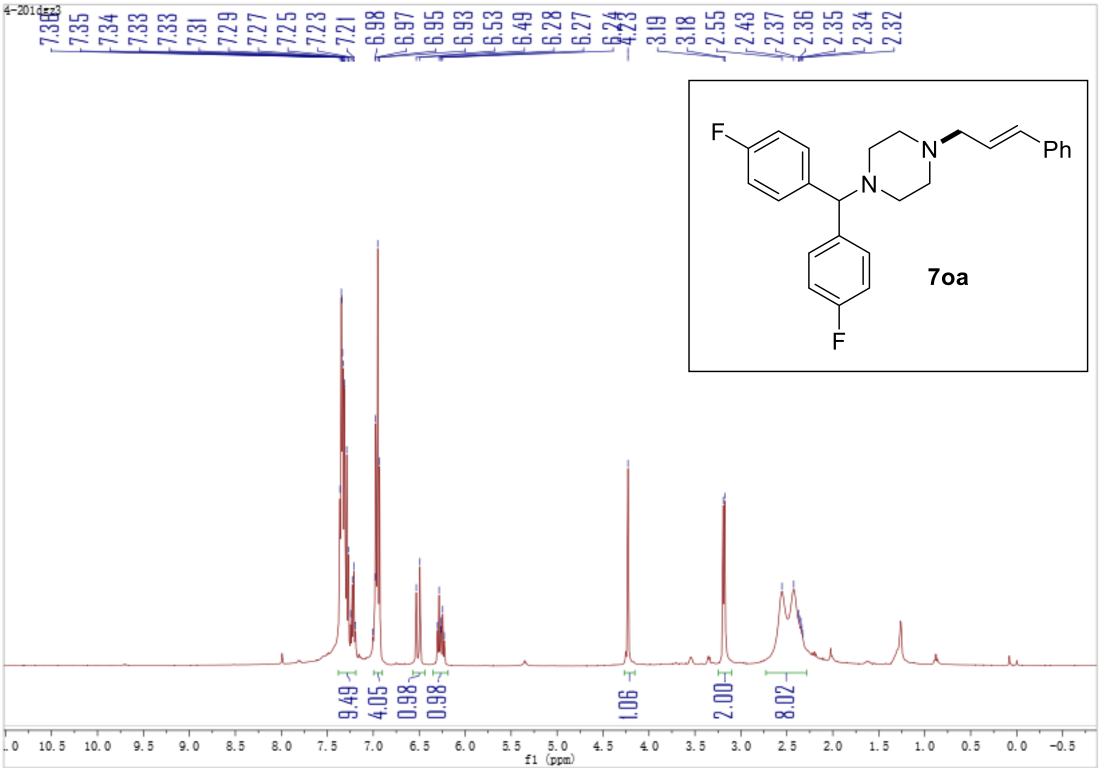

^{13}C NMR spectrum of compound **7oa** ($CDCl_3$, 101 MHz)

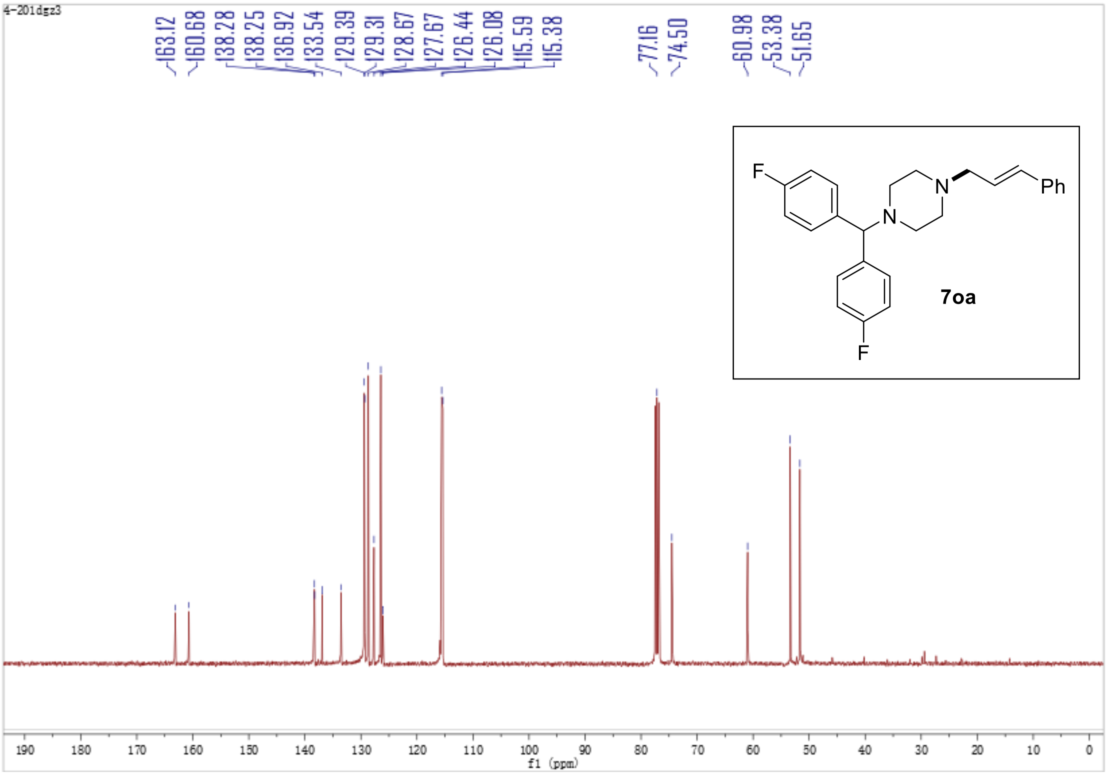

^{19}F NMR spectrum of compound **7oa** ($CDCl_3$,376 MHz)

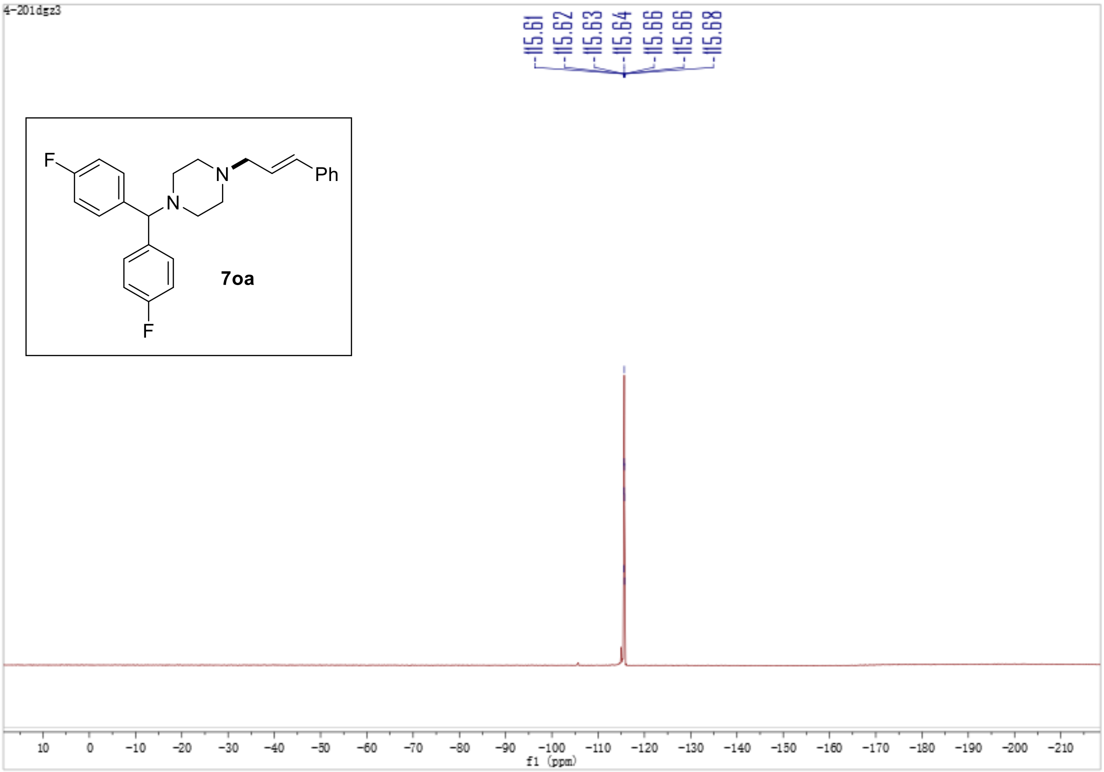

[1]**H NMR** spectrum of compound **8aa** ($CDCl_3$, 400 MHz)

Links CATALOG DETAILS NMR

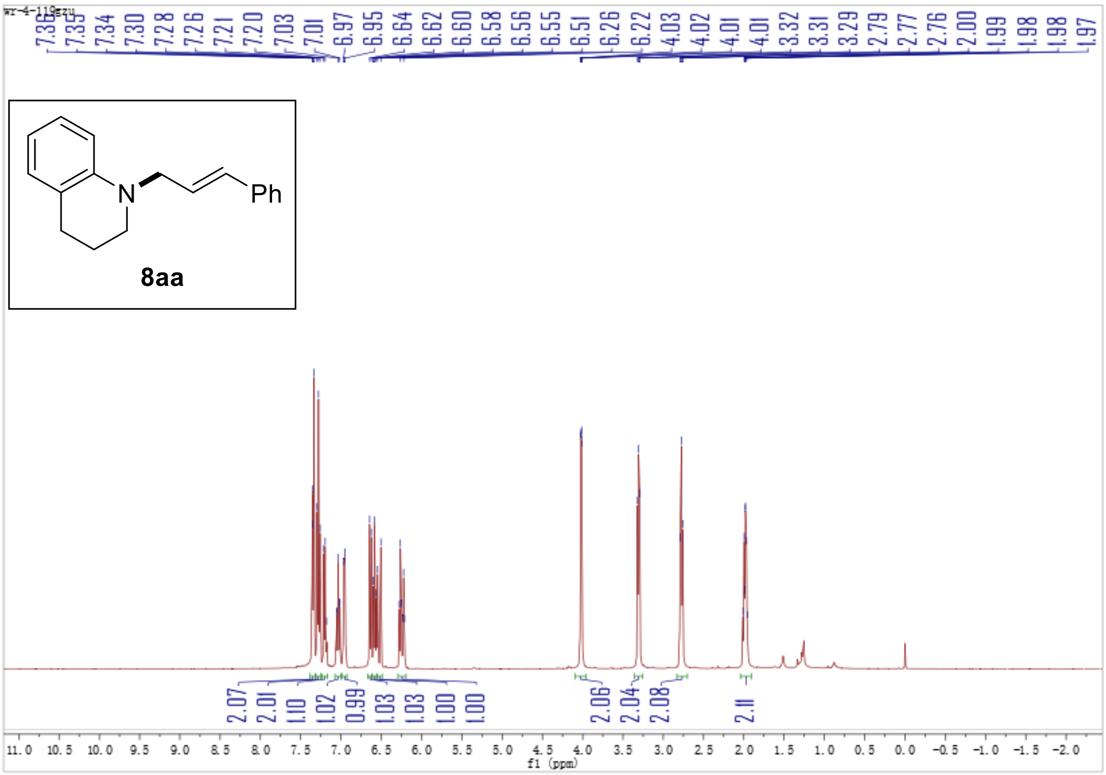

[13]**C NMR** spectrum of compound **8aa** ($CDCl_3$, 101 MHz)

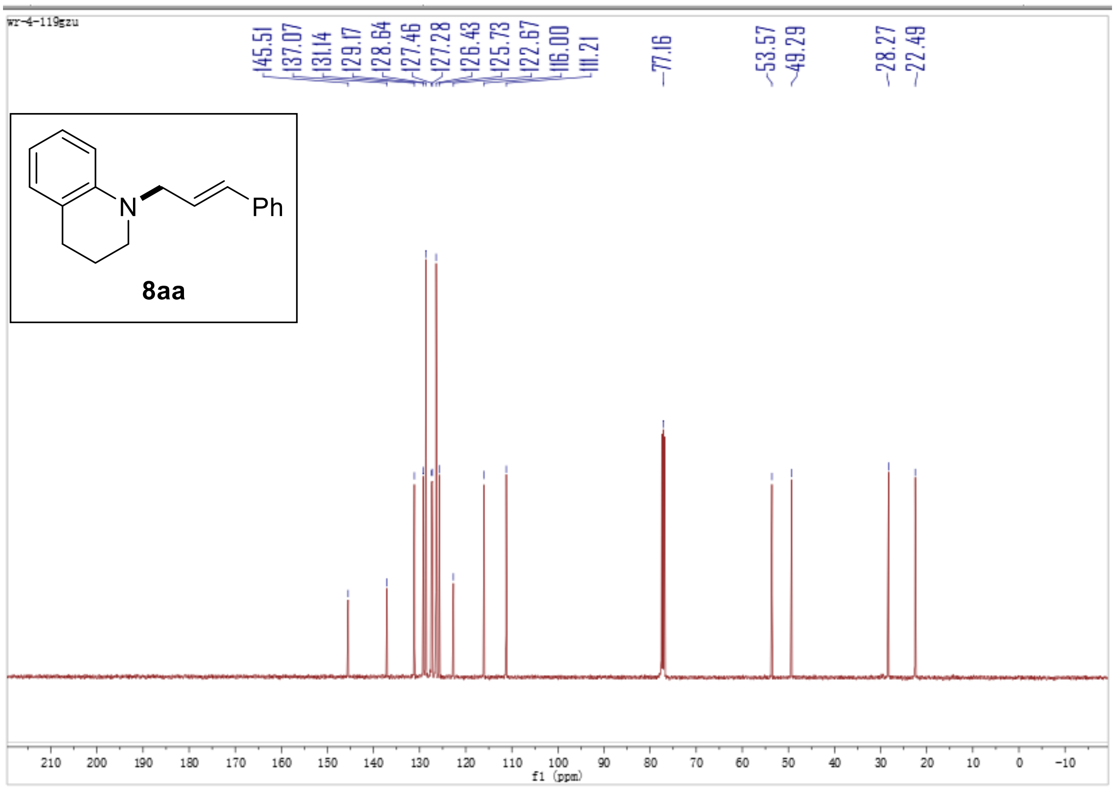

^{1}H NMR spectrum of compound **7hd** ($CDCl_3$, 400 MHz)

Links CATALOG DETAILS NMR

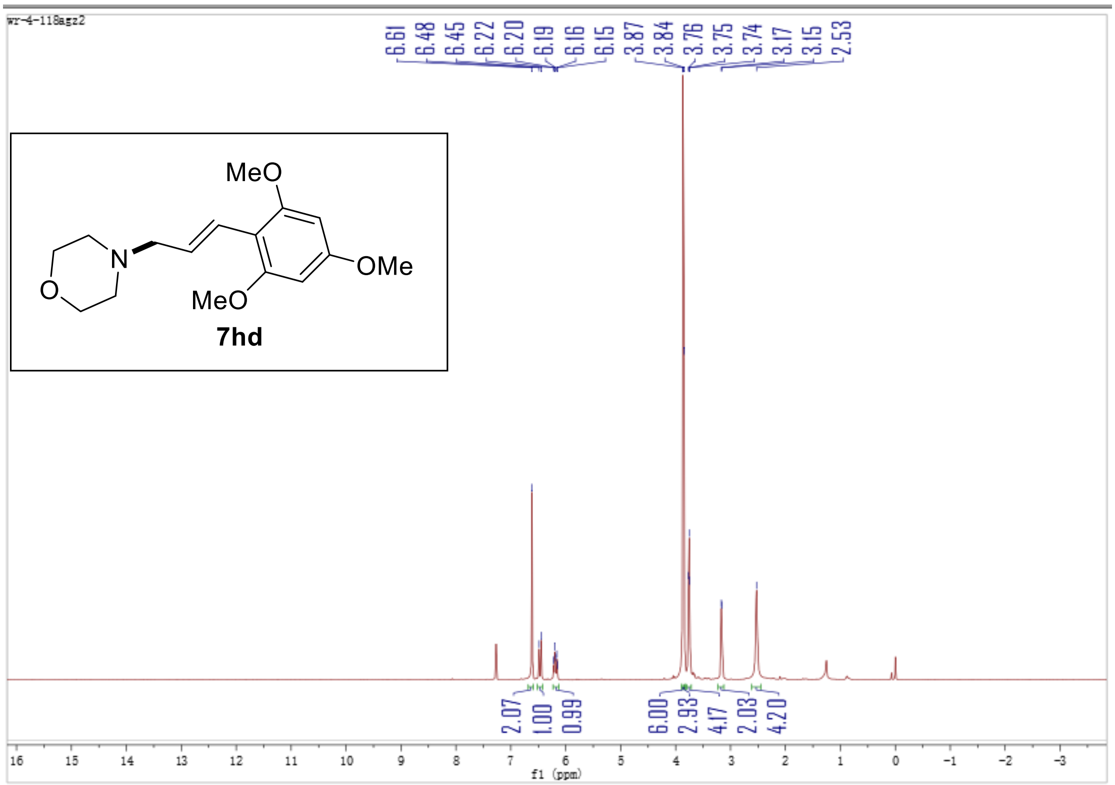

^{13}C NMR spectrum of compound **7hd** ($CDCl_3$, 101 MHz)

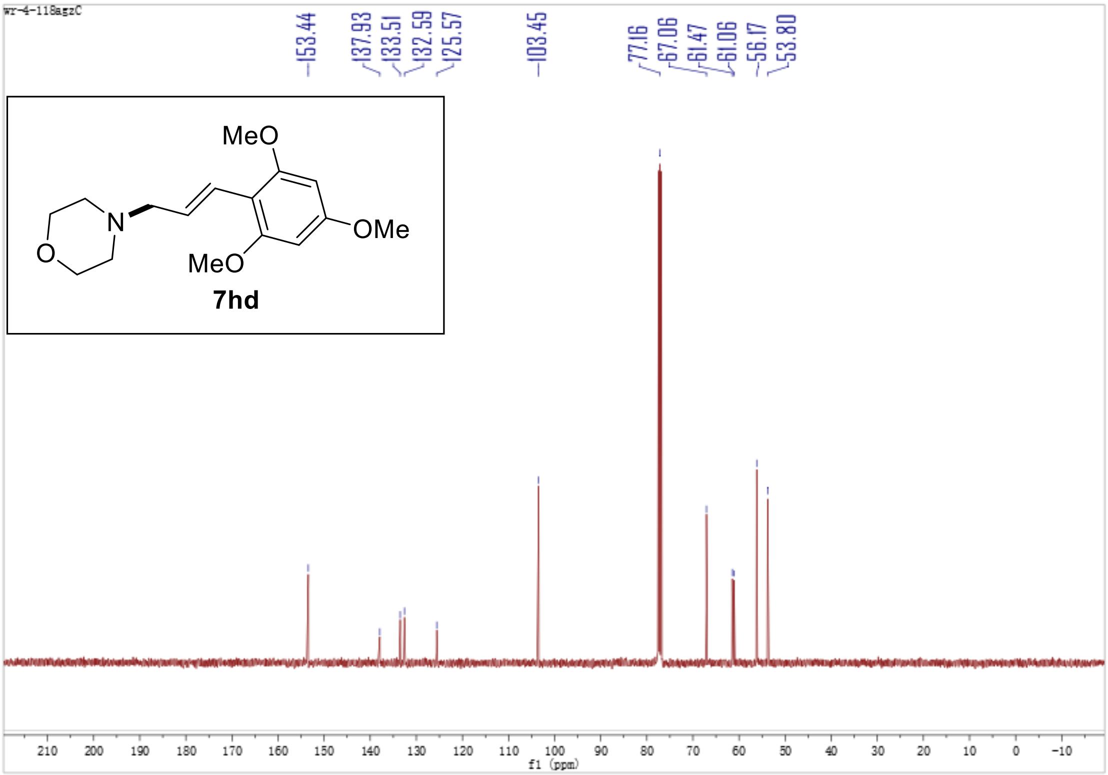

^{1}H NMR spectrum of compound **7he** ($CDCl_3$, 400 MHz)

Links CATALOG DETAILS NMR

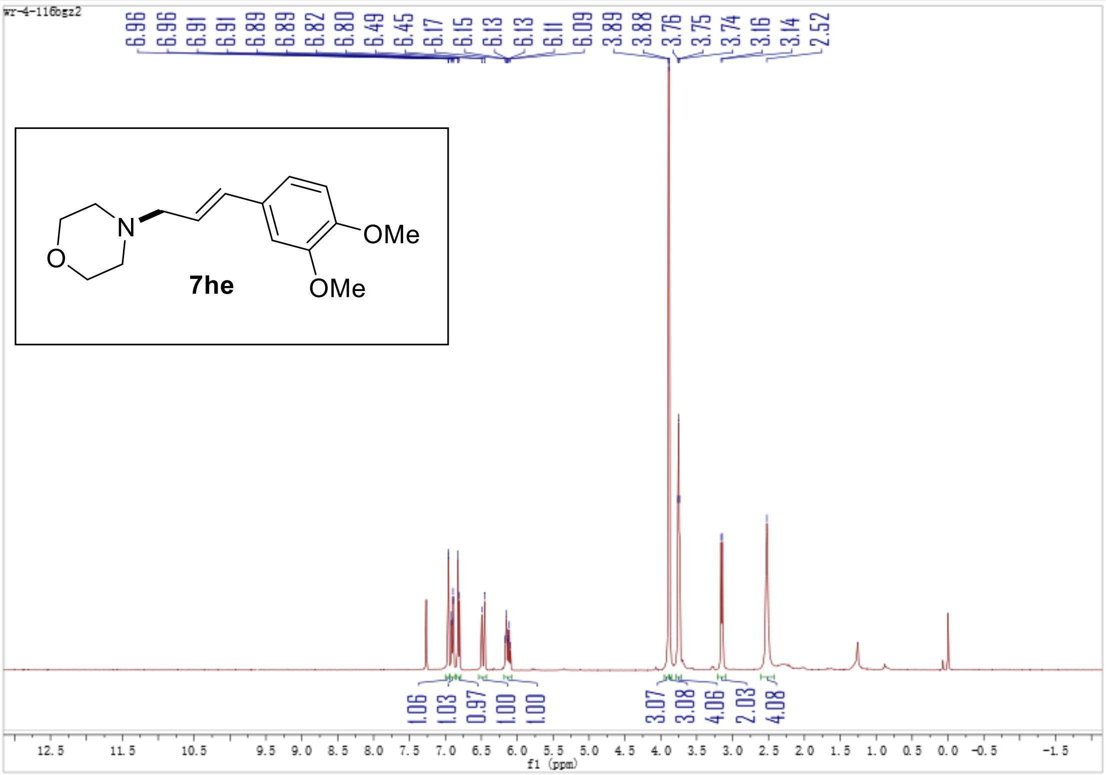

^{13}C NMR spectrum of compound **7he** ($CDCl_3$, 101 MHz)

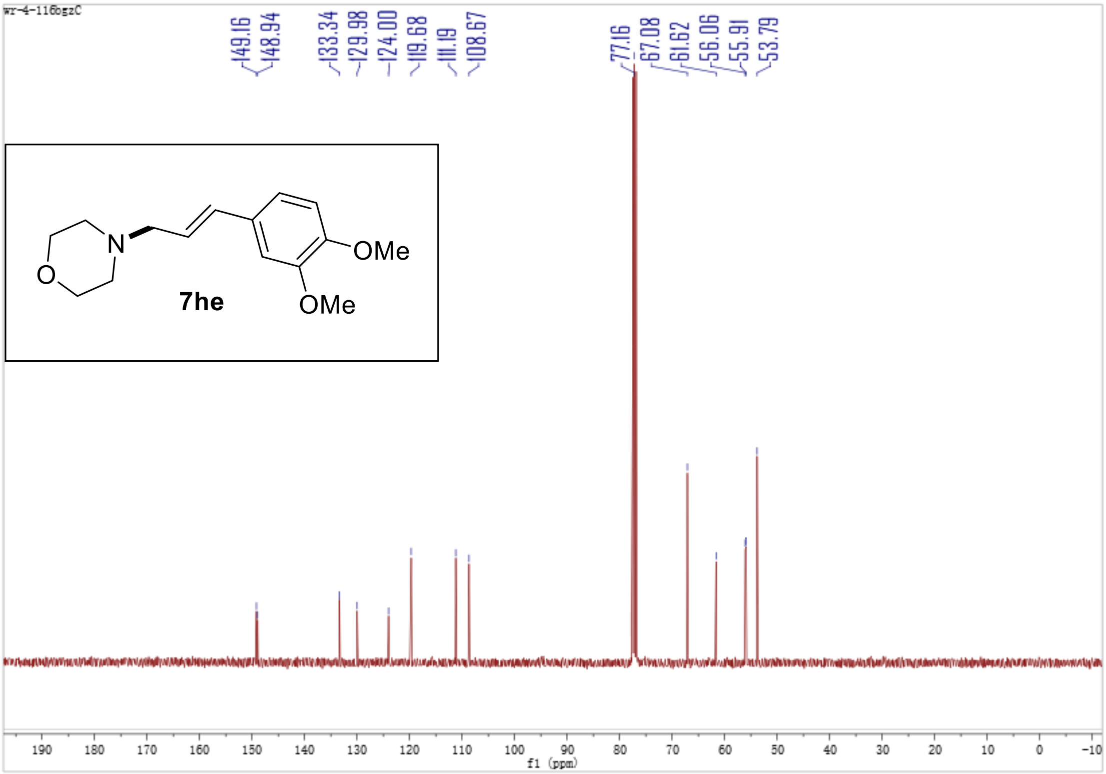

[1]**H NMR** spectrum of compound **7hg** ($CDCl_3$, 400 MHz)

Links CATALOG DETAILS NMR

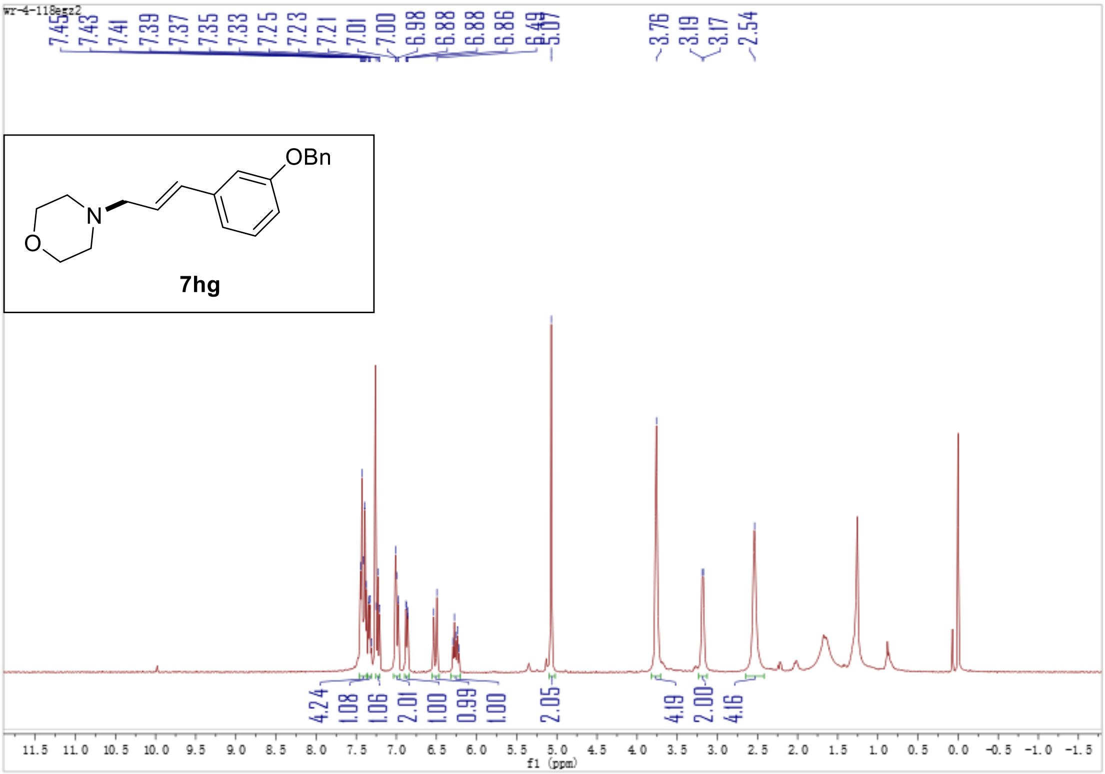

[13]**C NMR** spectrum of compound **7hg** ($CDCl_3$, 101 MHz)

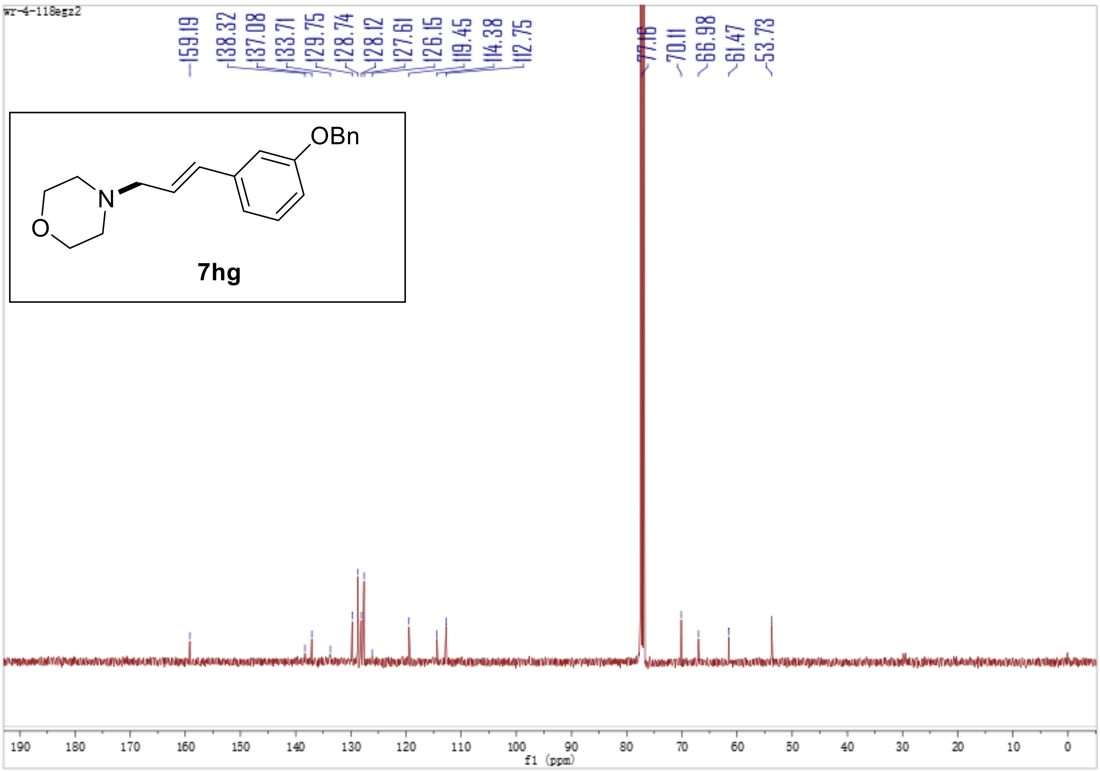

^{1}H NMR spectrum of compound **7hh** ($CDCl_3$, 400 MHz)

Links CATALOG DETAILS NMR

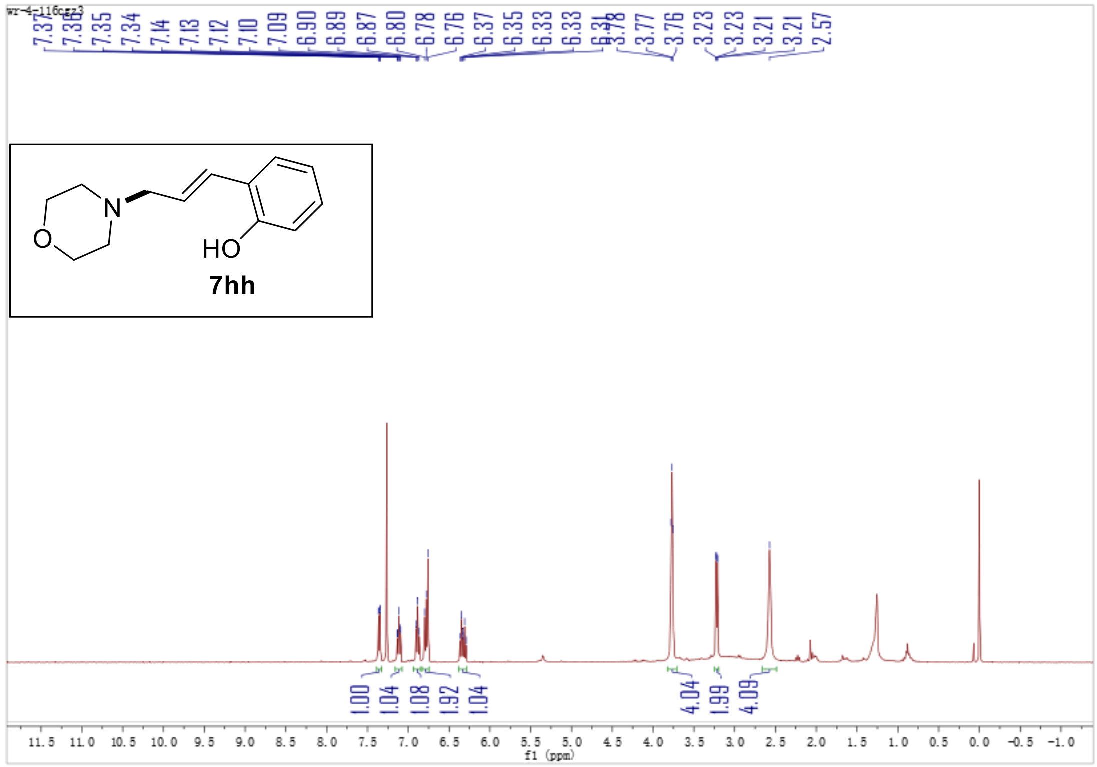

^{13}C NMR spectrum of compound **7hh** ($CDCl_3$, 101 MHz)

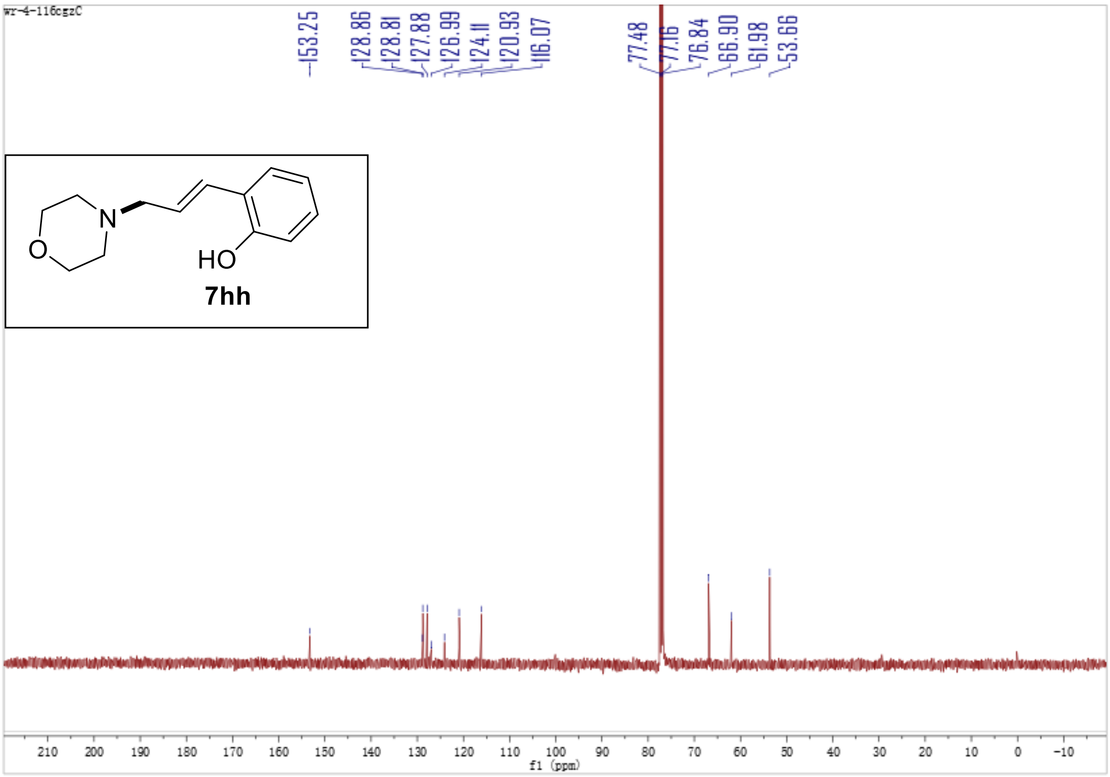

[1]**H NMR** spectrum of compound **7hi** ($CDCl_3$, 400 MHz)

Links CATALOG DETAILS NMR

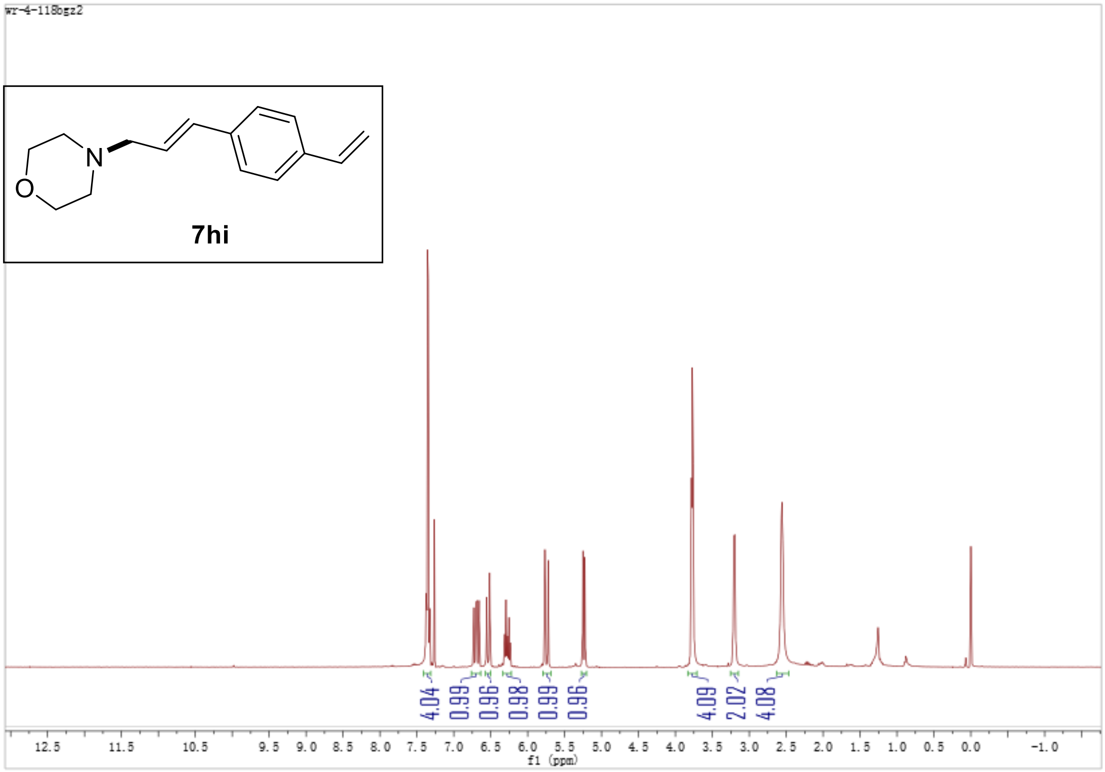

[13]**C NMR** spectrum of compound **7hi** ($CDCl_3$, 101 MHz)

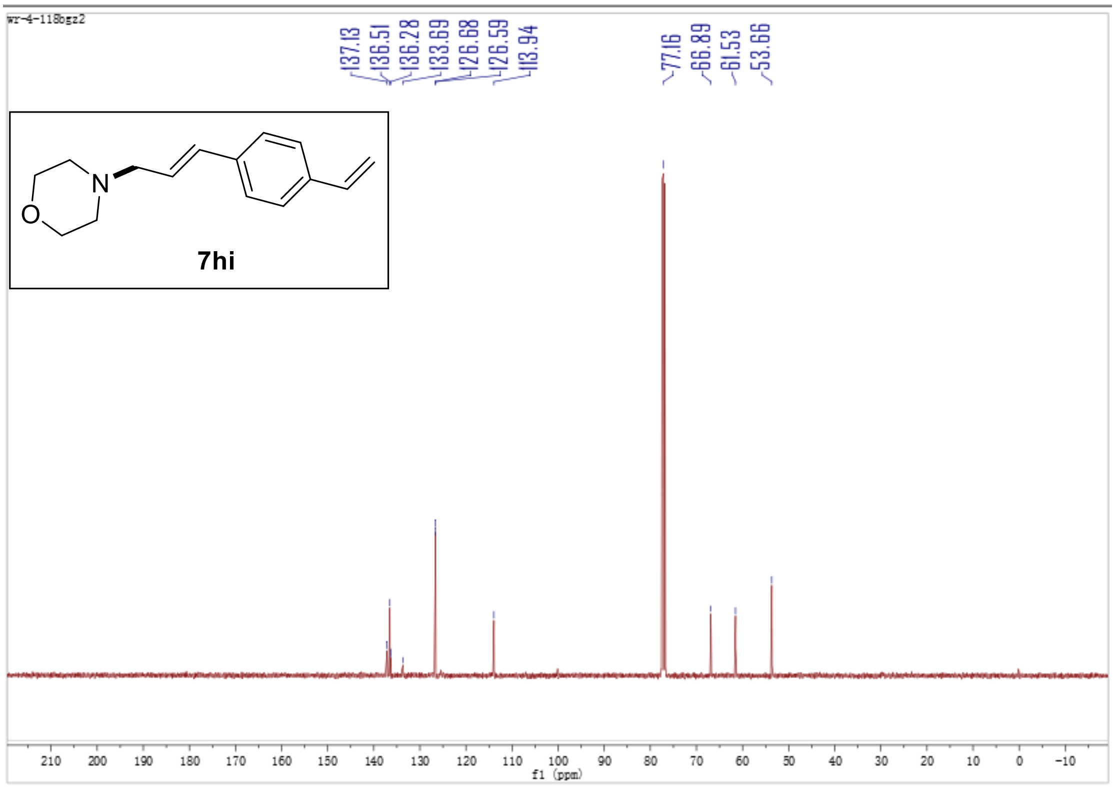

^{1}H NMR spectrum of compound **7hm** ($CDCl_3$, 400 MHz)

Links CATALOG DETAILS NMR

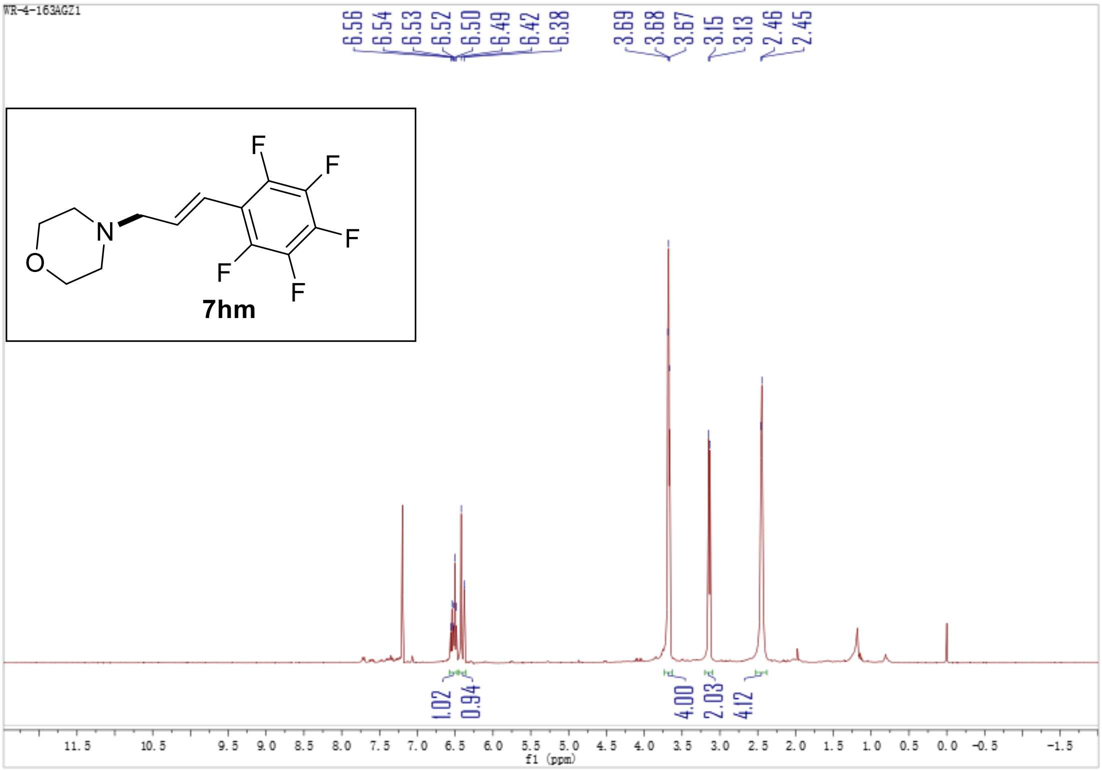

^{13}C NMR spectrum of compound **7hm** ($CDCl_3$, 101 MHz)

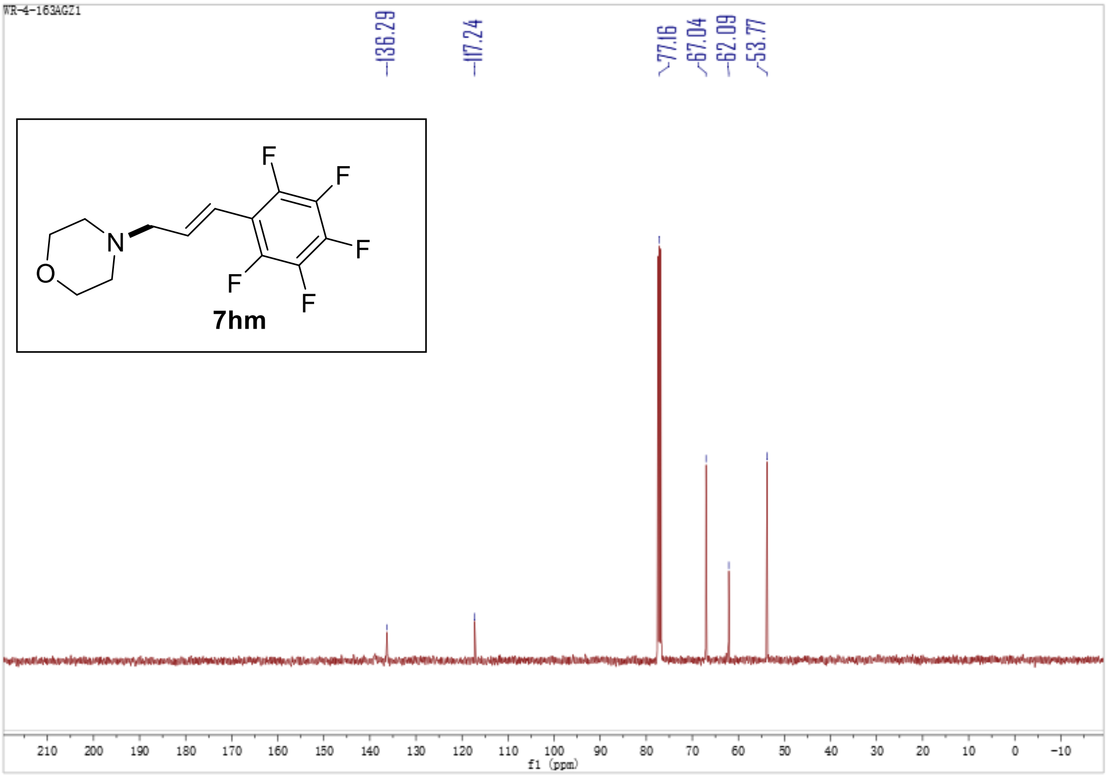

^{19}**F NMR** spectrum of compound **7hm** ($CDCl_3$, 376 MHz)

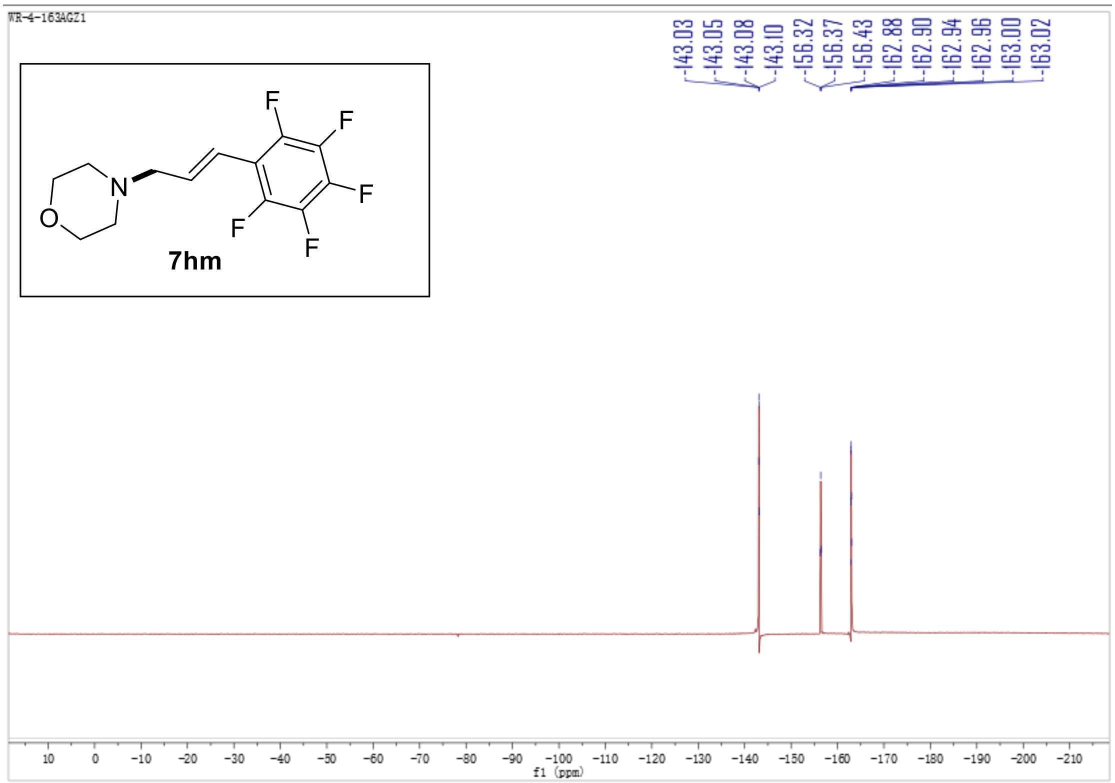

[1]**H NMR** spectrum of compound **7ht** ($CDCl_3$, 400 MHz)

Links CATALOG DETAILS NMR

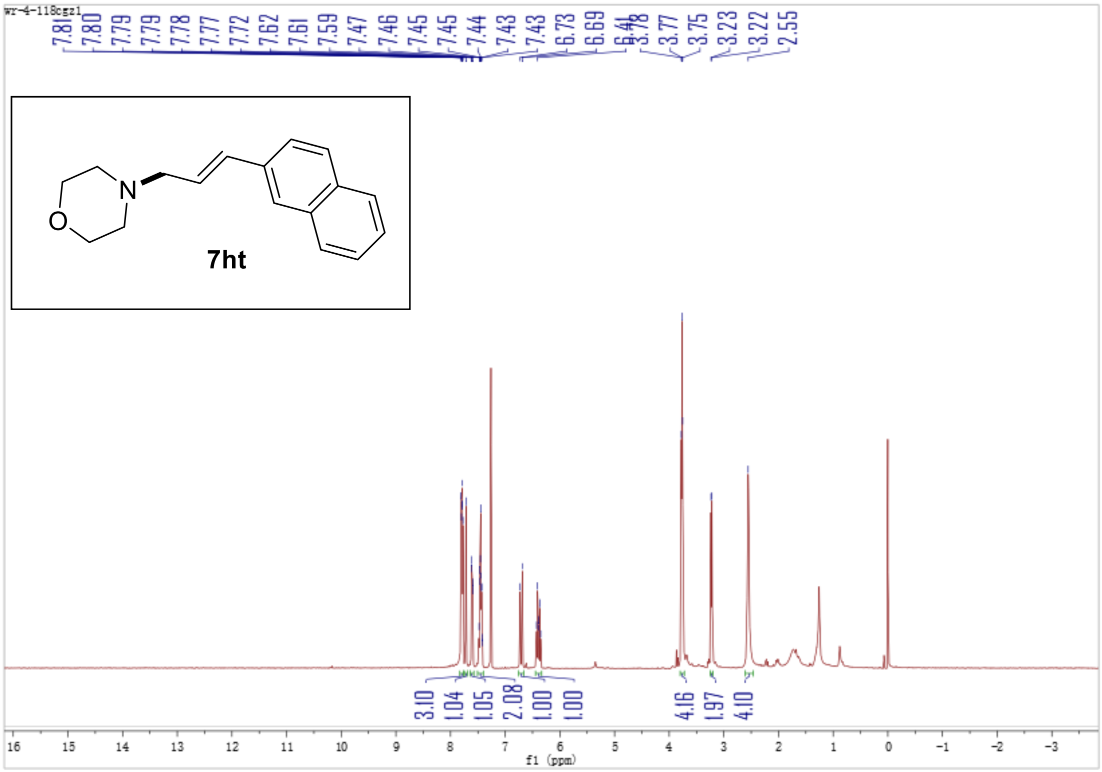

[13]**C NMR** spectrum of compound **7ht** ($CDCl_3$, 101 MHz)

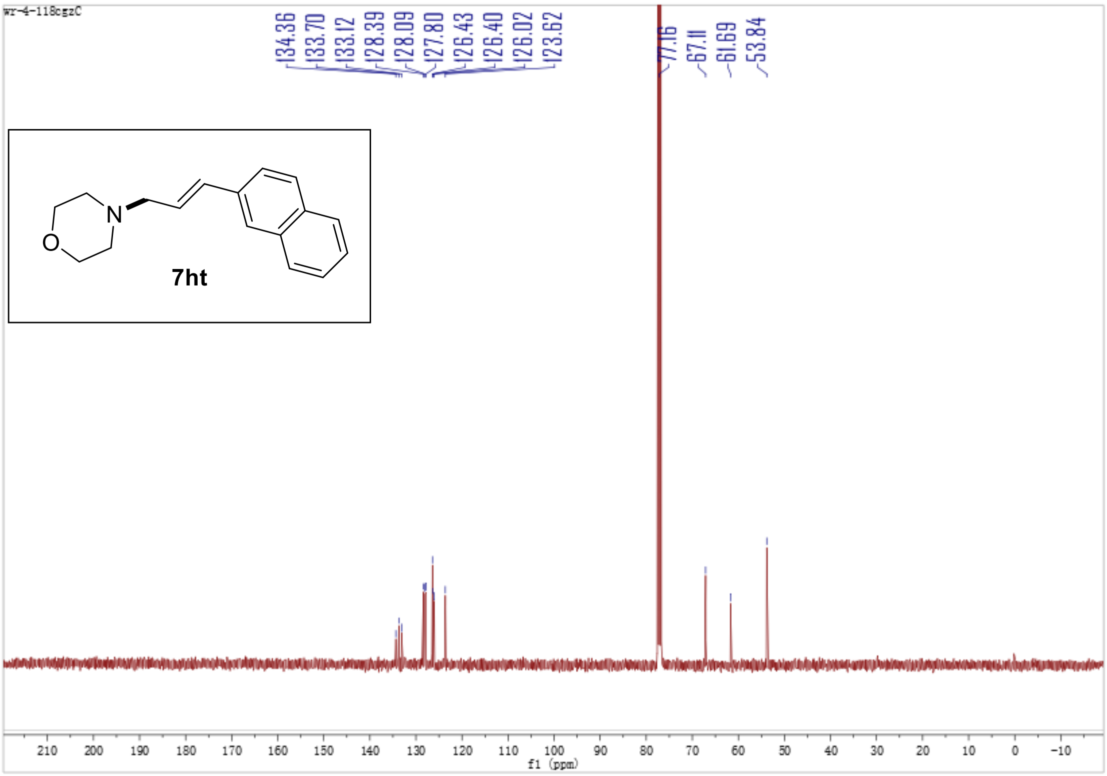

[1]**H NMR** spectrum of compound **7hw** ($CDCl_3$, 400 MHz)

Links CATALOG DETAILS NMR

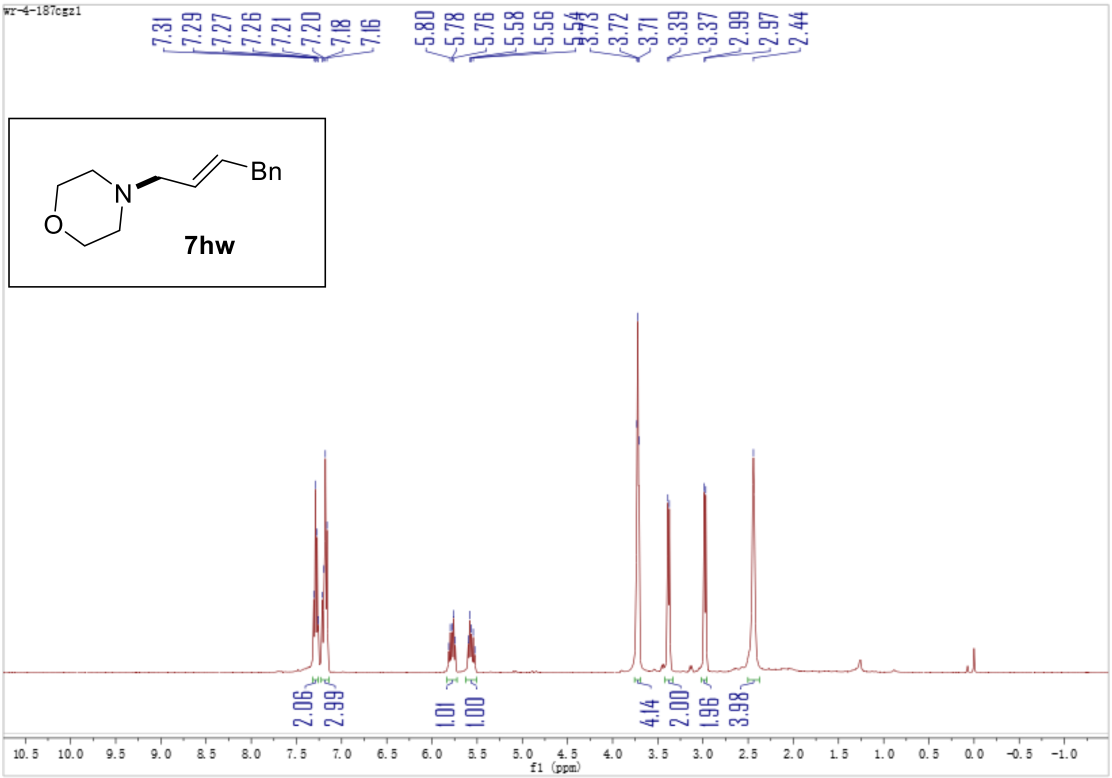

[13]**C NMR** spectrum of compound **7hw** ($CDCl_3$, 101 MHz)

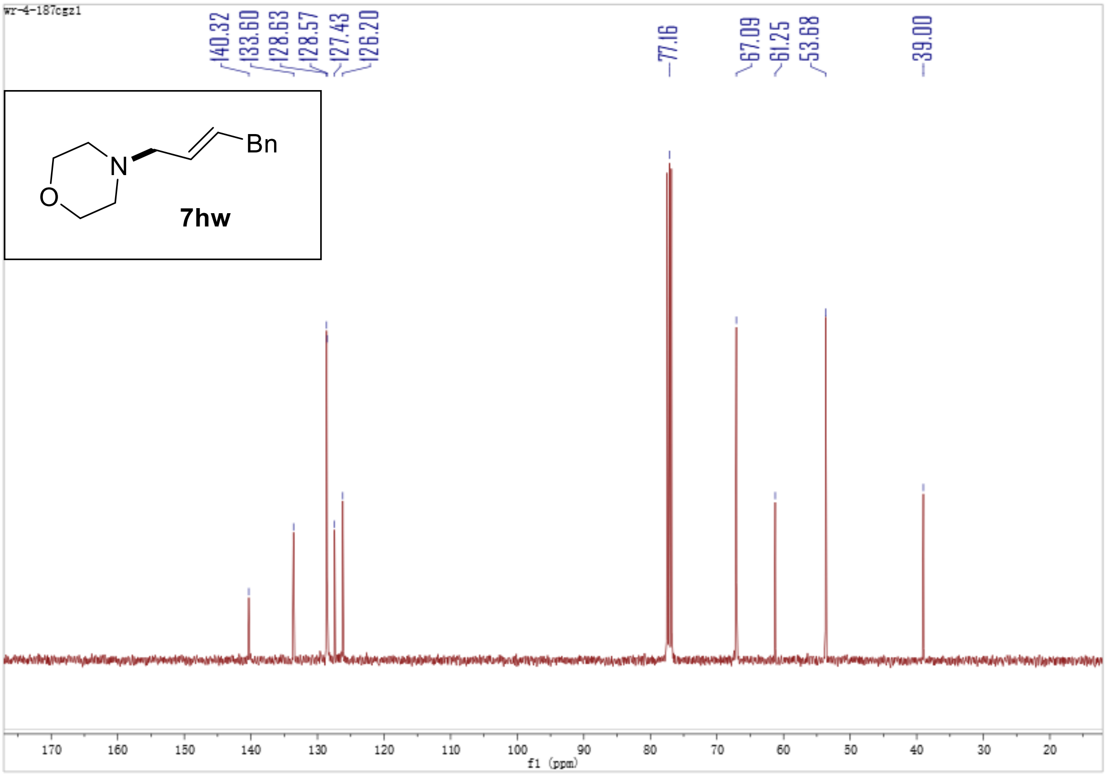

^{1}H NMR spectrum of compound **7pe** ($CDCl_3$, 400 MHz)

Links CATALOG DETAILS NMR

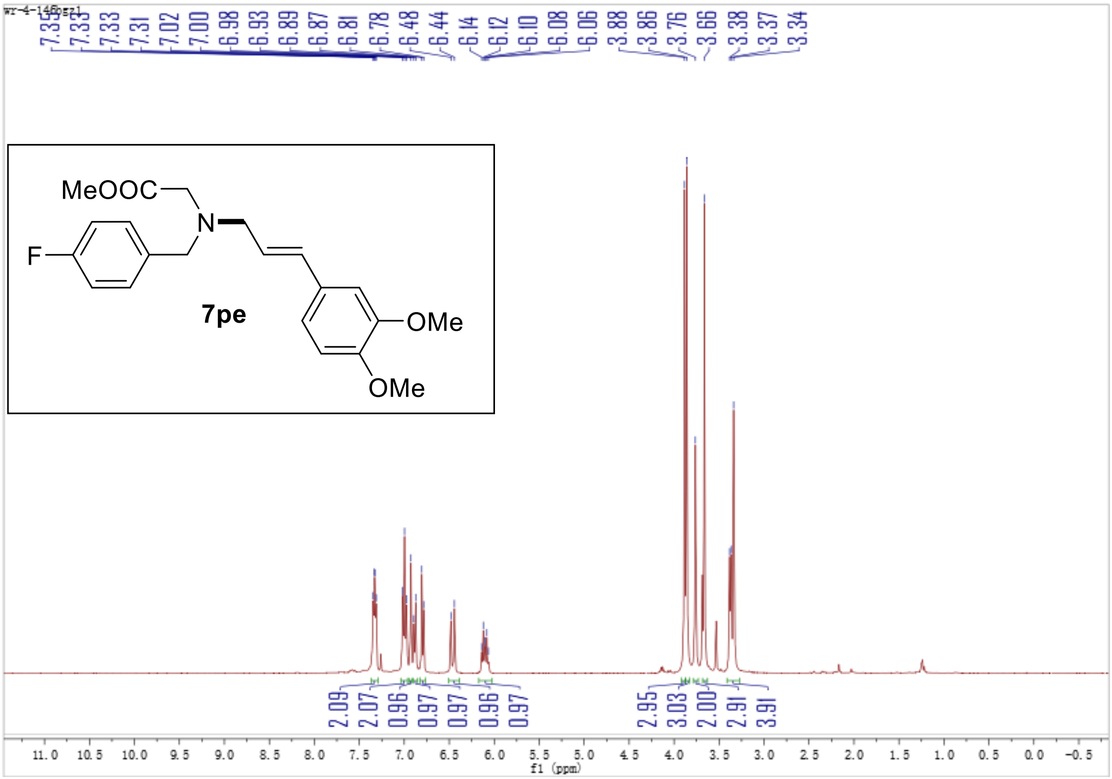

^{13}C NMR spectrum of compound **7pe** ($CDCl_3$, 101 MHz)

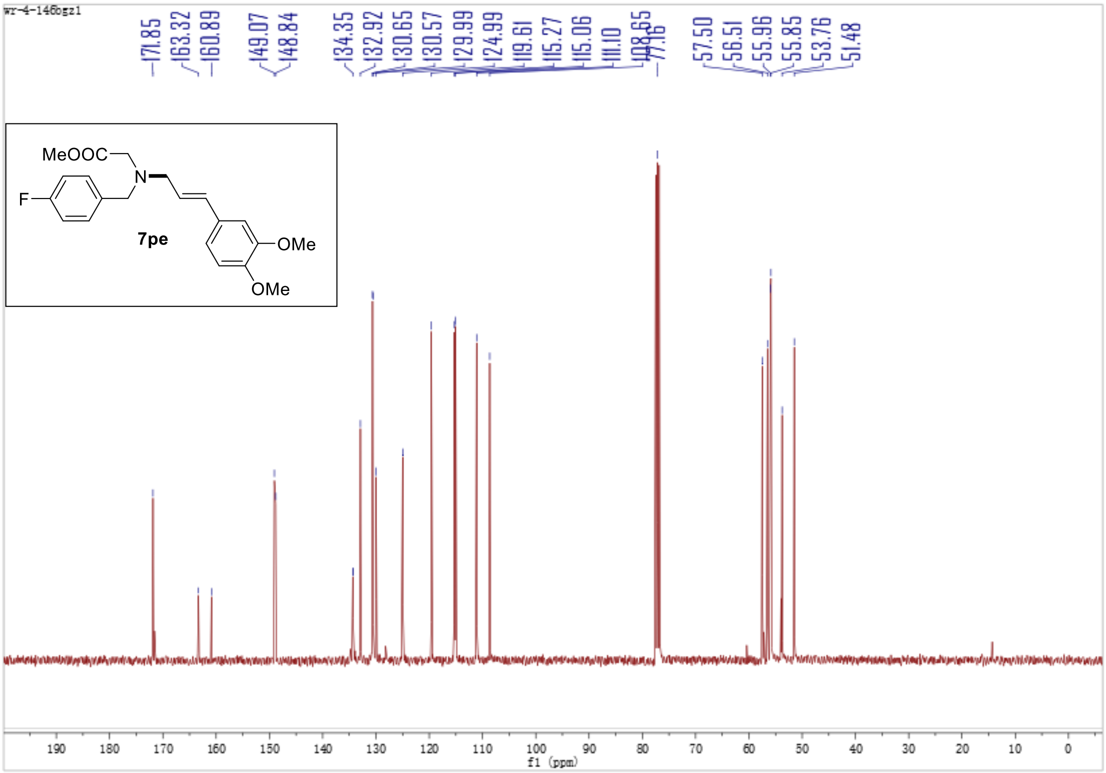

[1]**H NMR** spectrum of compound **8ba-mono** ($CDCl_3$, 400 MHz)

Links CATALOG DETAILS NMR

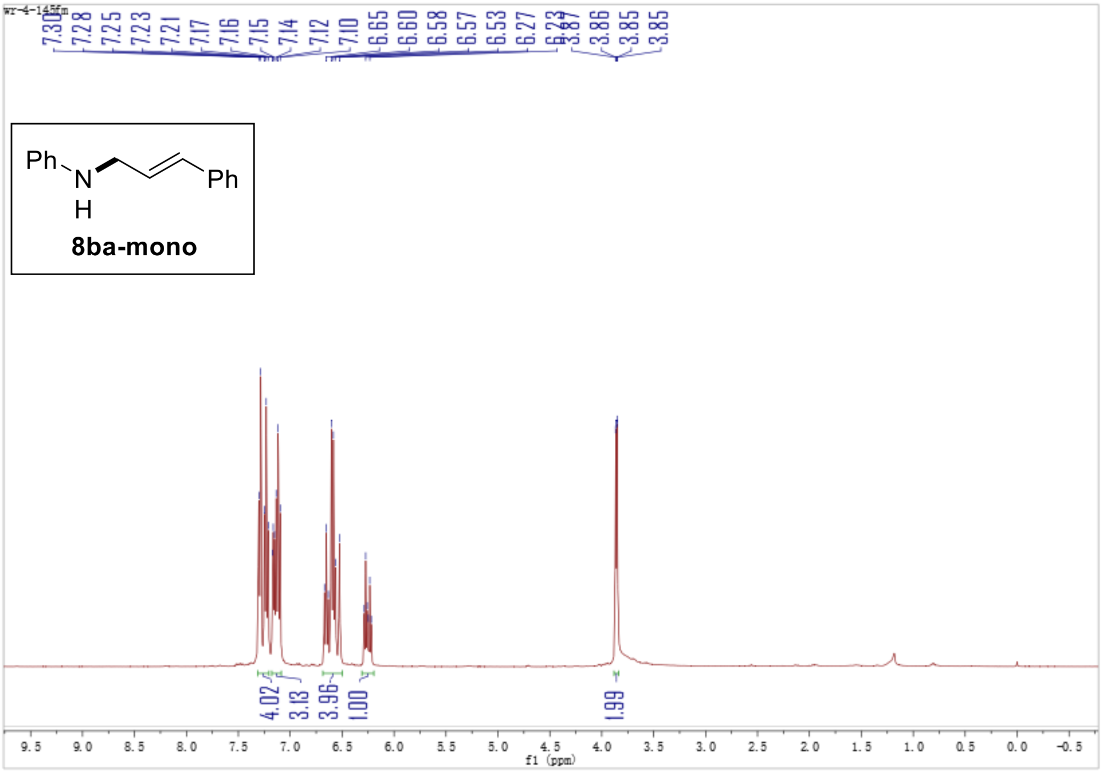

[13]**C NMR** spectrum of compound **8ba-mono** ($CDCl_3$, 101 MHz)

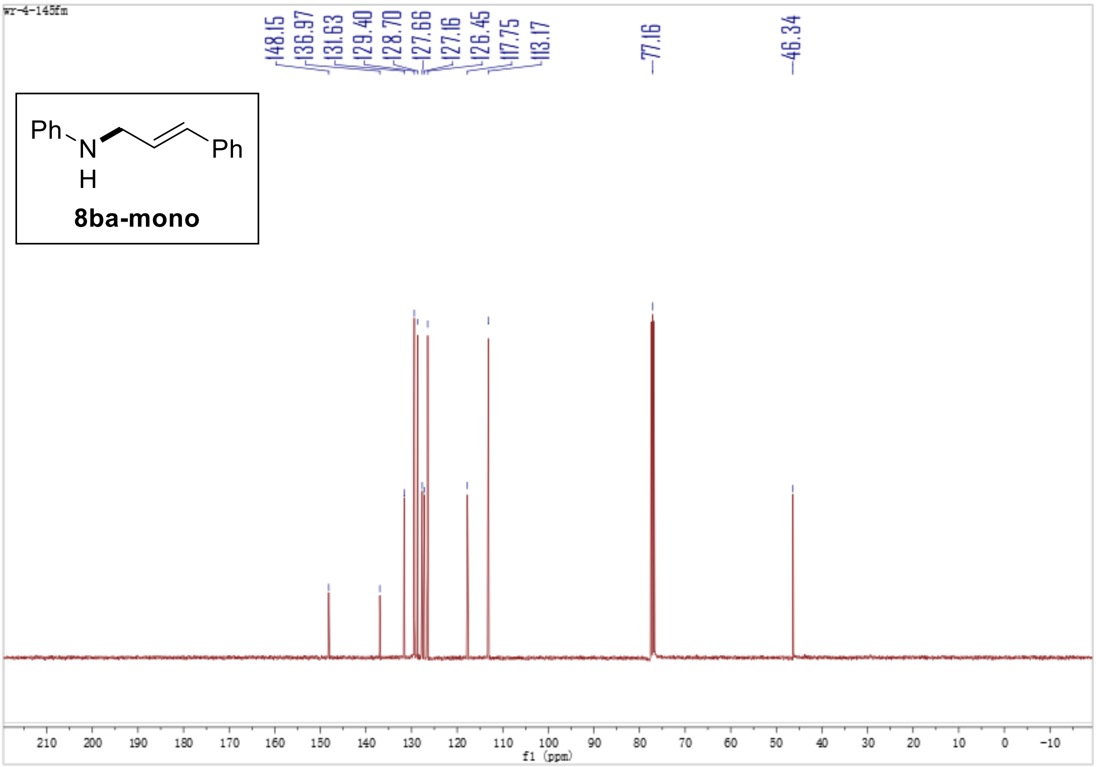

^{1}H NMR spectrum of compound **8ca-mono** ($CDCl_3$, 400 MHz)

Links CATALOG DETAILS NMR

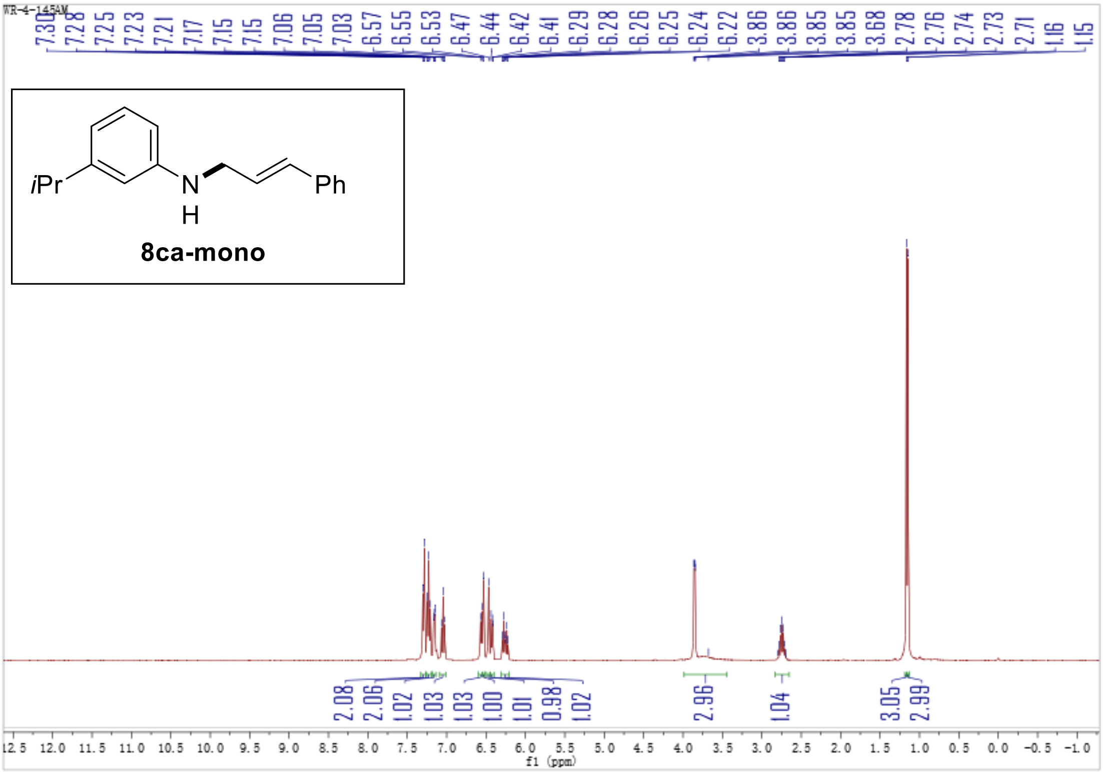

^{13}C NMR spectrum of compound **8ca-mono** ($CDCl_3$, 101 MHz)

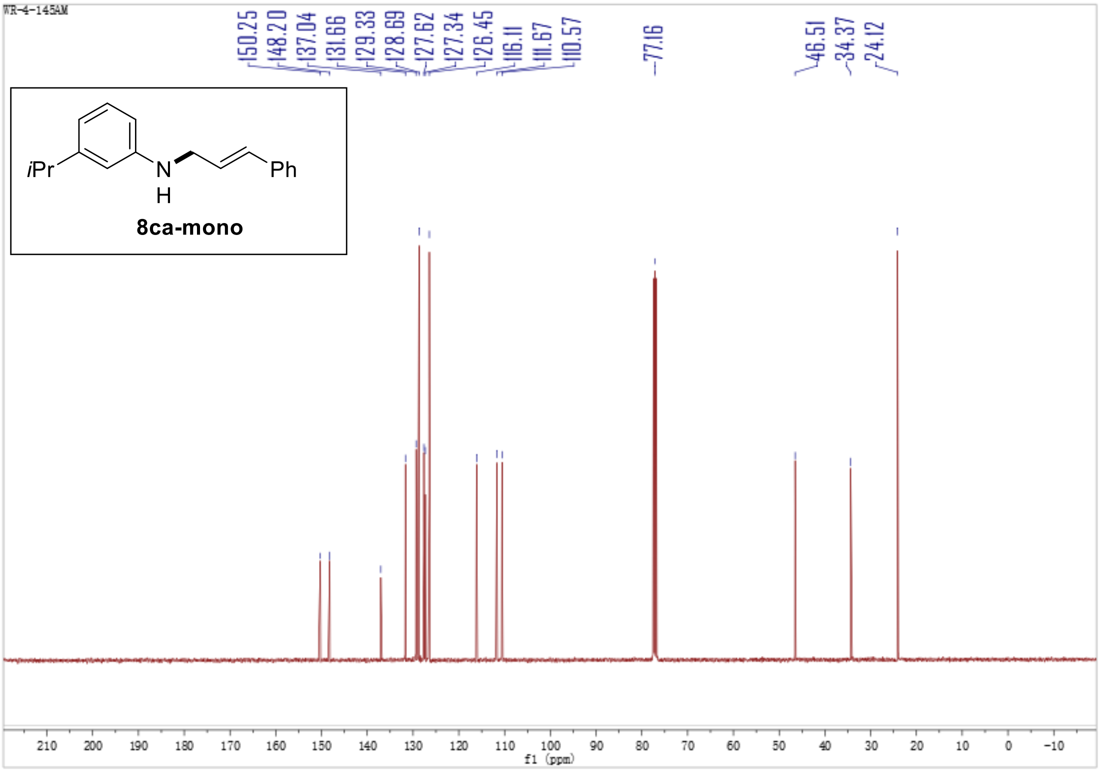

^{1}H NMR spectrum of compound **8da-mono** ($CDCl_3$, 400 MHz)

Links CATALOG DETAILS NMR

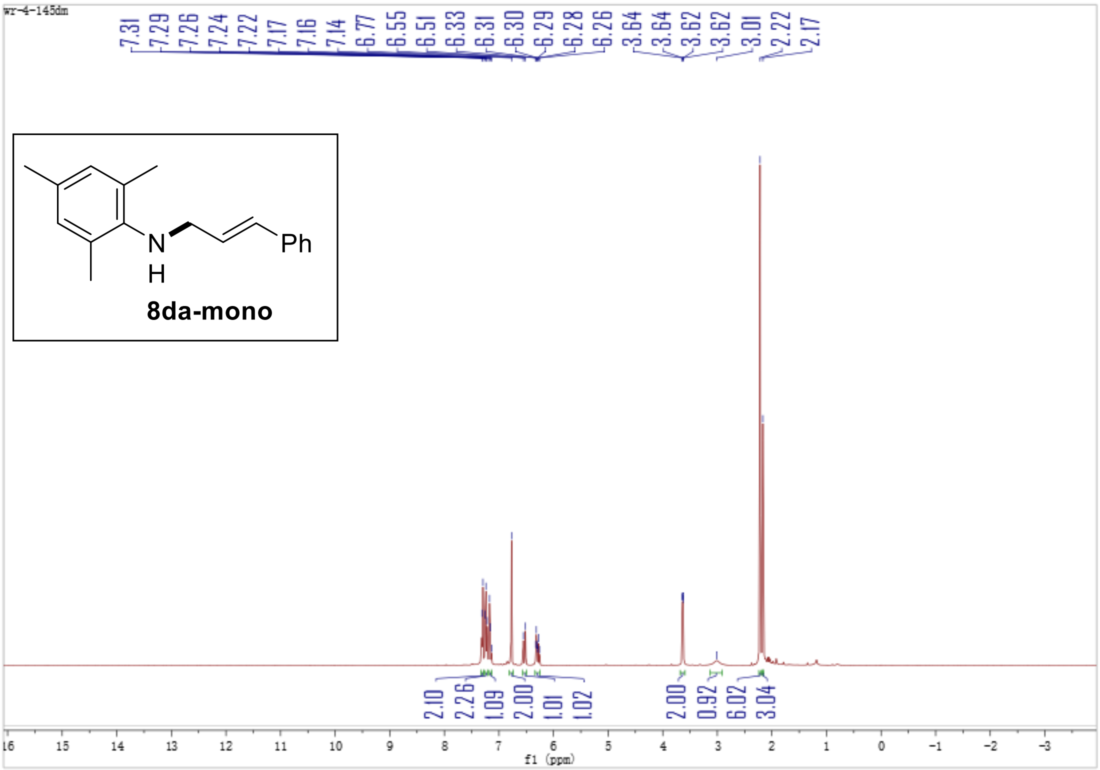

^{13}C NMR spectrum of compound **8da-mono** ($CDCl_3$, 101 MHz)

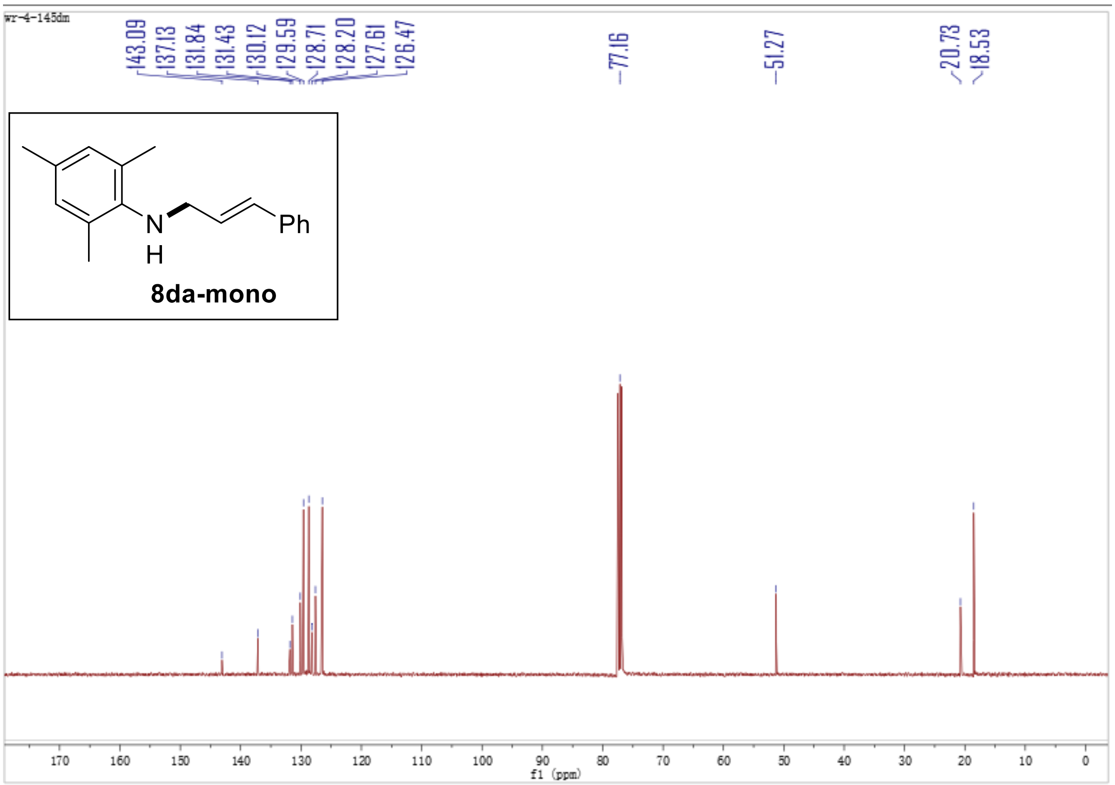

^{1}H NMR spectrum of compound **8ea-mono** ($CDCl_3$, 400 MHz)

Links CATALOG DETAILS NMR

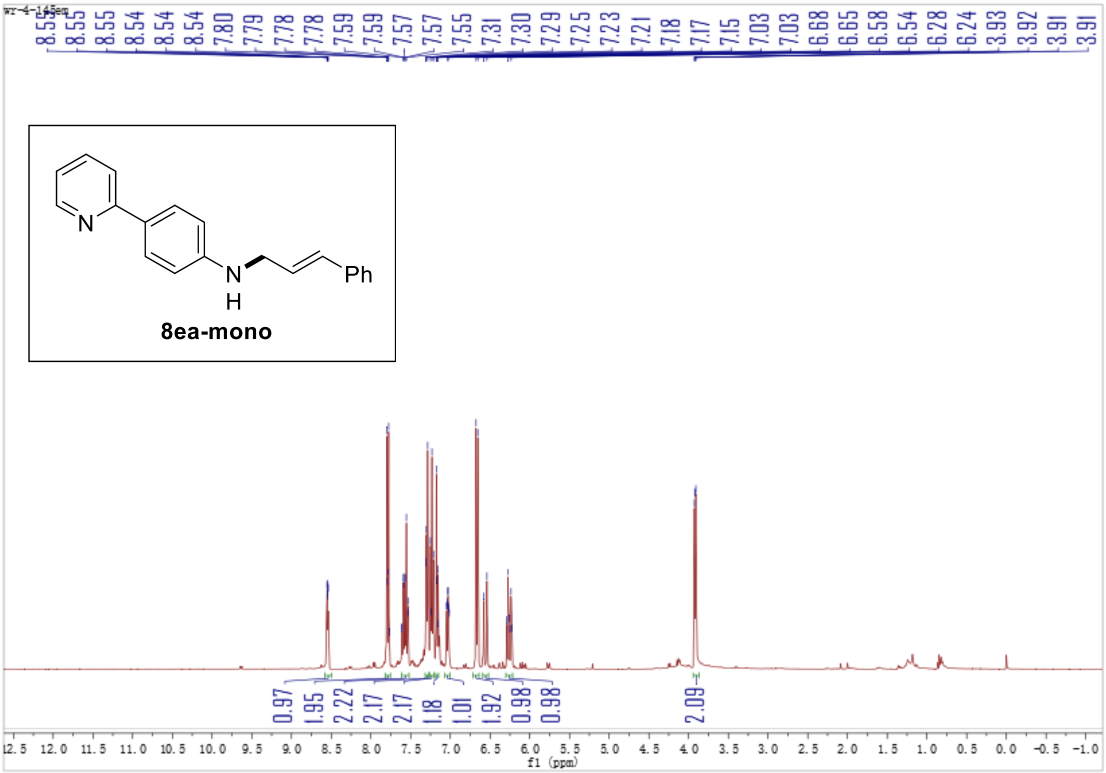

^{13}C NMR spectrum of compound **8ea-mono** ($CDCl_3$, 101 MHz)

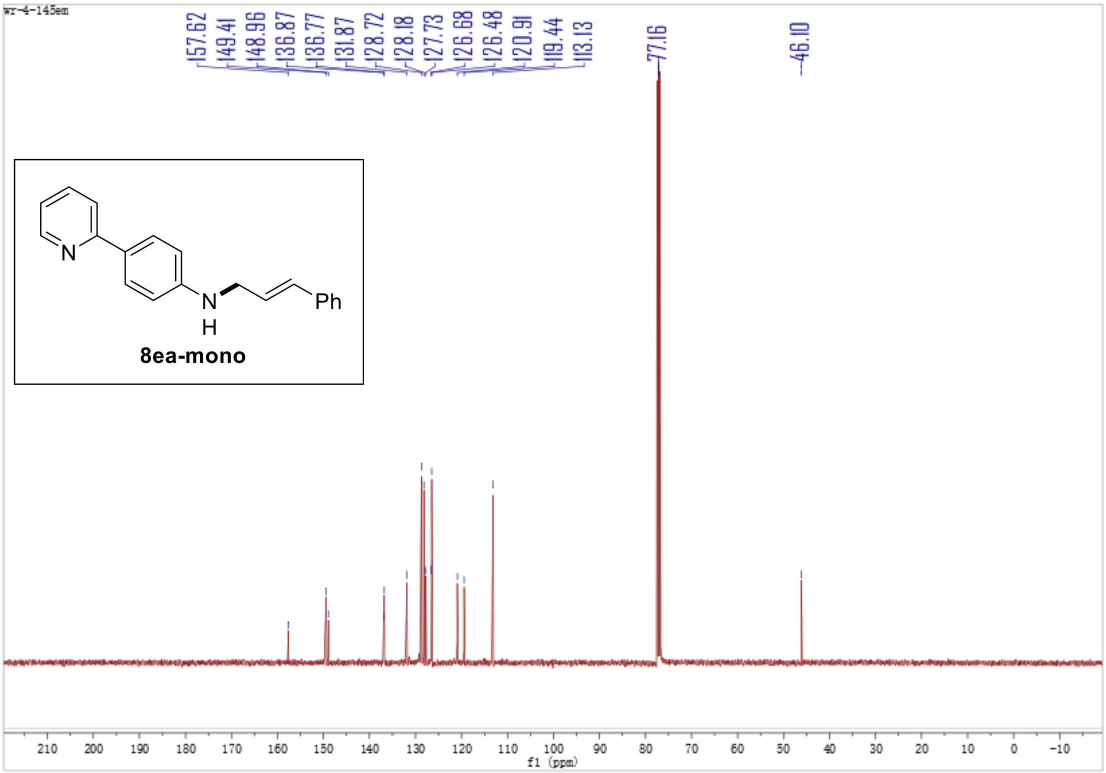

^{1}H NMR spectrum of compound **8fa-mono** ($CDCl_3$, 400 MHz)

Links CATALOG DETAILS NMR

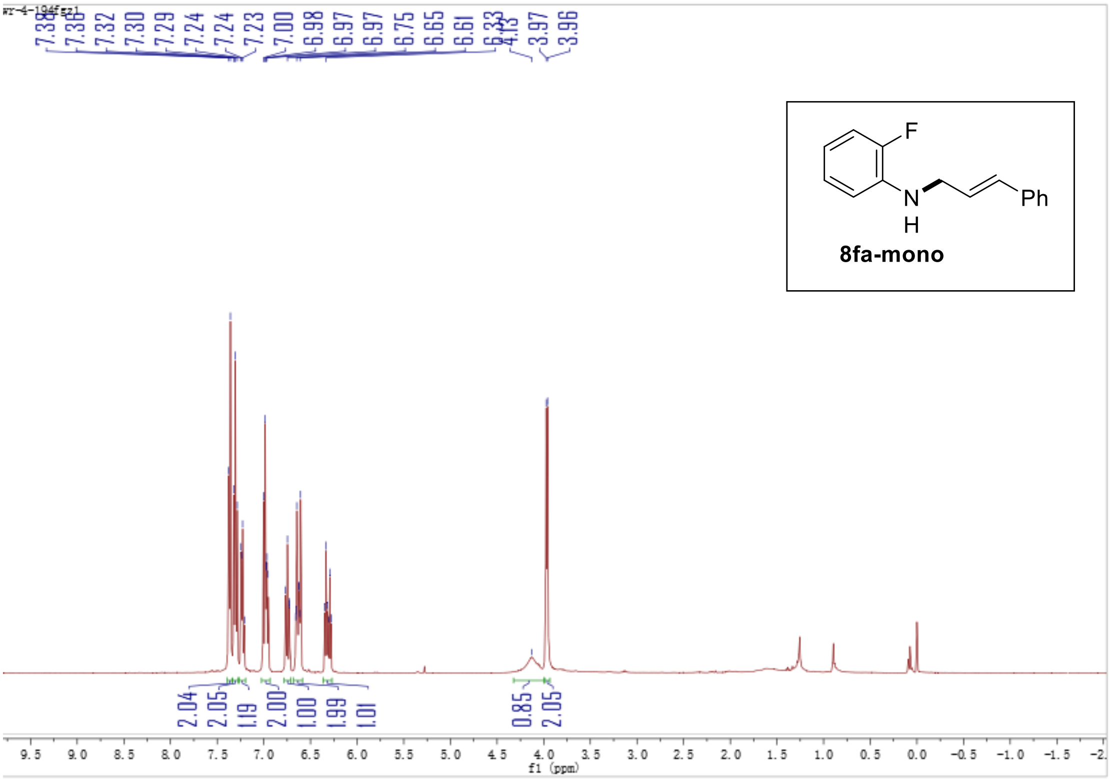

^{13}C NMR spectrum of compound **8fa-mono** ($CDCl_3$, 101 MHz)

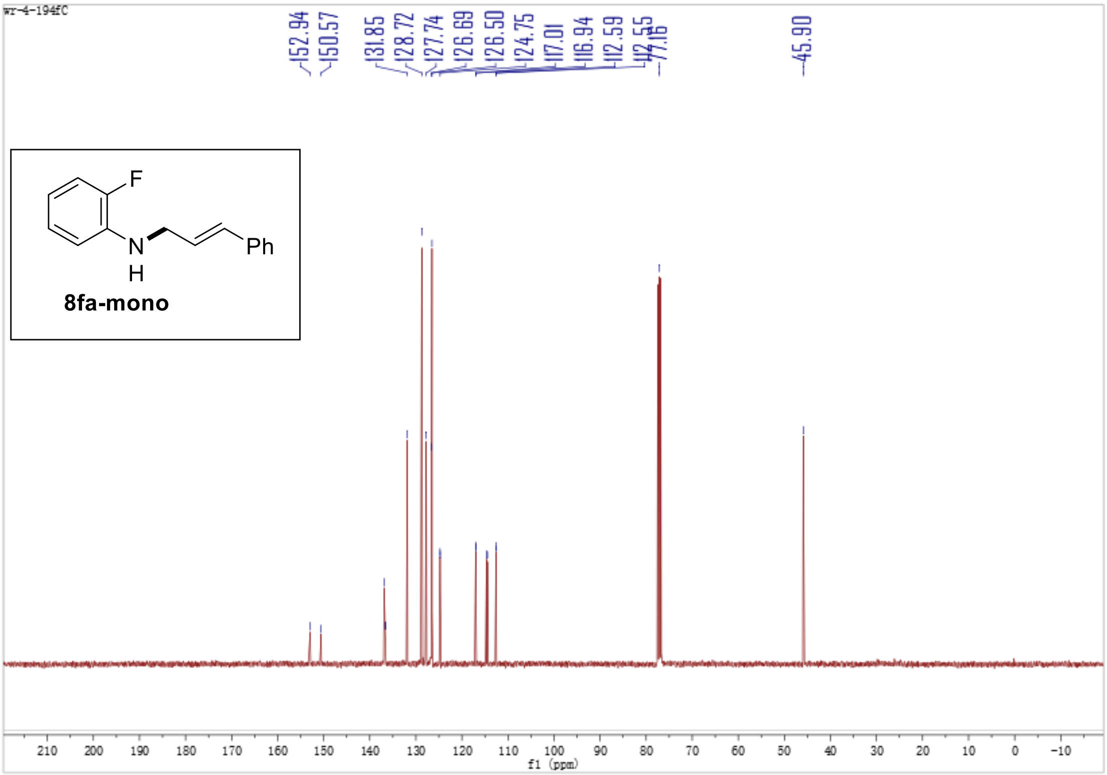

[19]**F NMR** spectrum of compound **8fa-mono** ($CDCl_3$, 376 MHz)

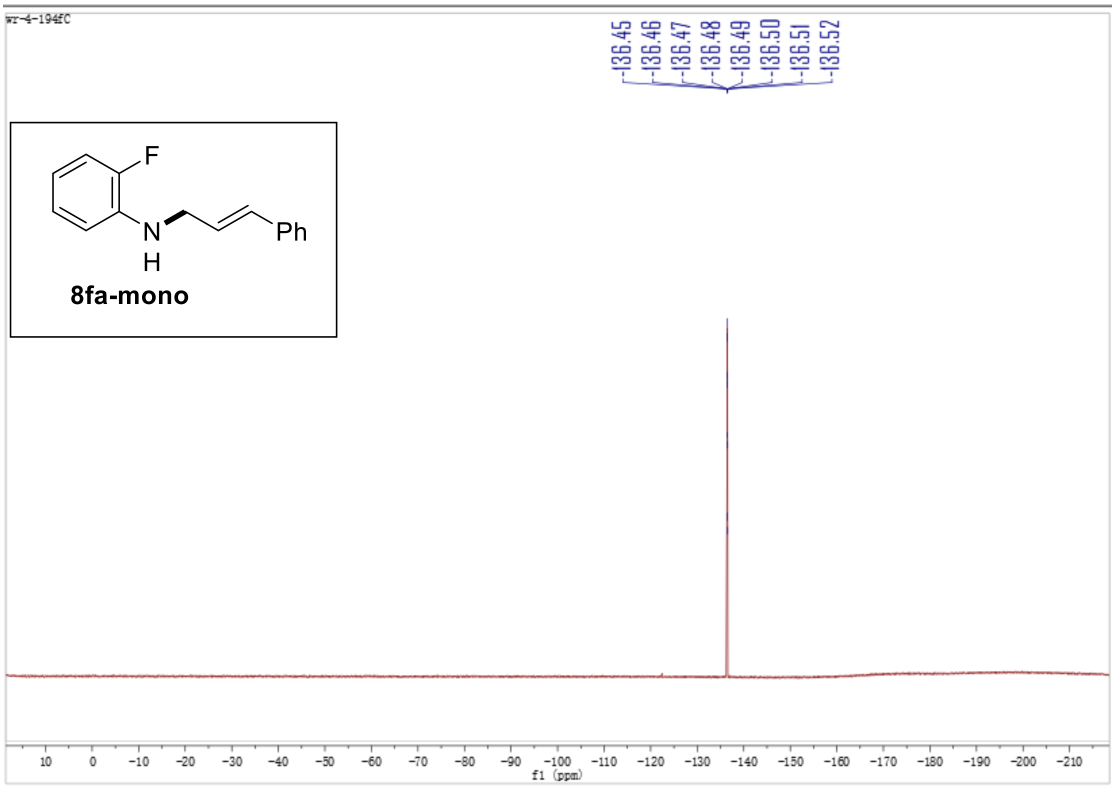

[1]**H NMR** spectrum of compound **8ga-mono** ($CDCl_3$, 400 MHz)

Links CATALOG DETAILS NMR

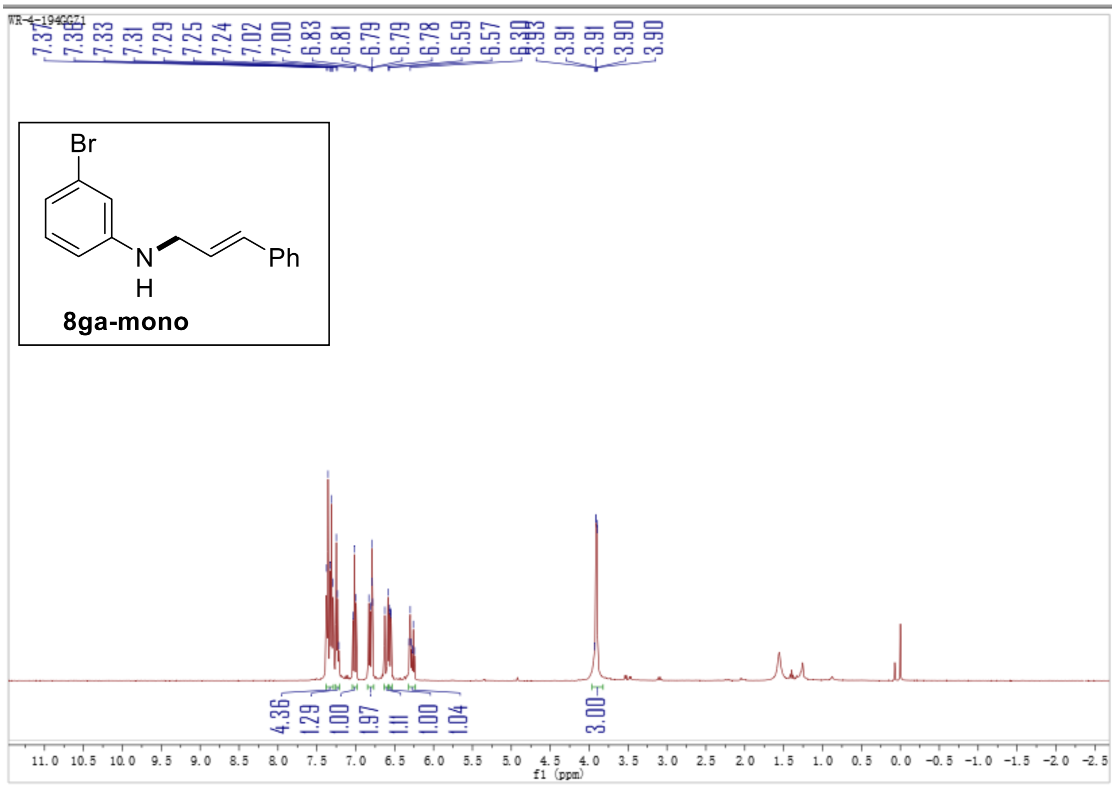

[13]**C NMR** spectrum of compound **8ga-mono** ($CDCl_3$, 101 MHz)

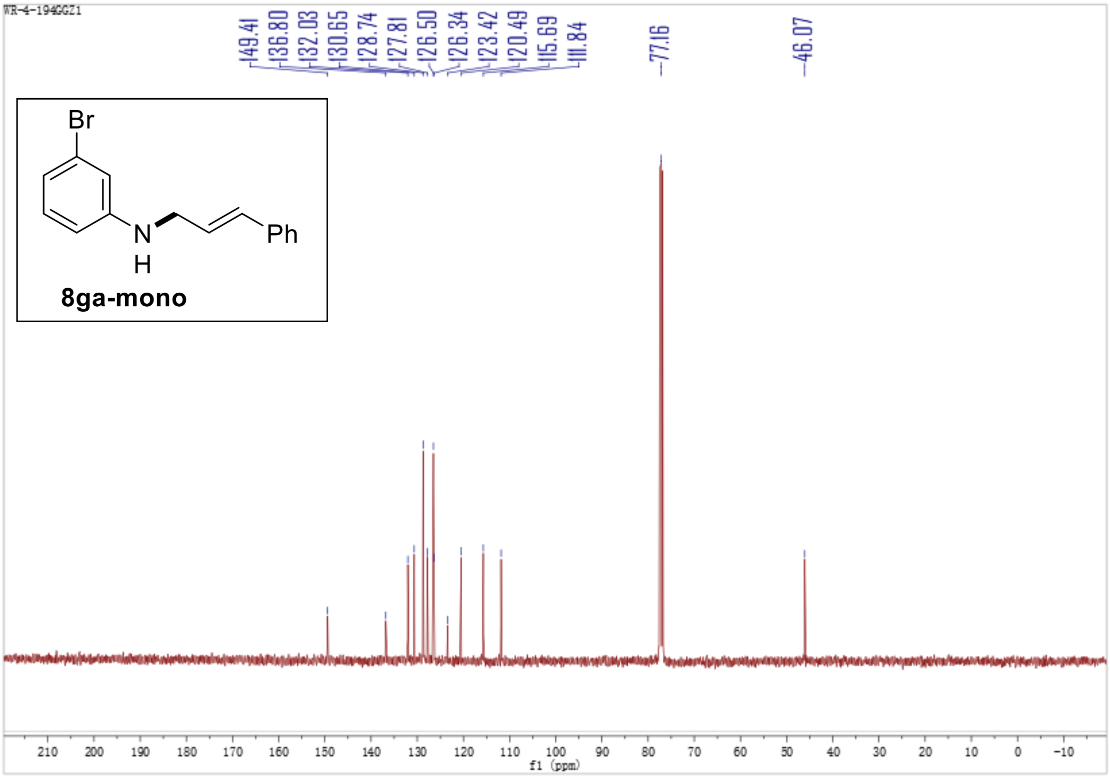

^{1}H NMR spectrum of compound **8ha-mono** ($CDCl_3$, 400 MHz)

Links CATALOG DETAILS NMR

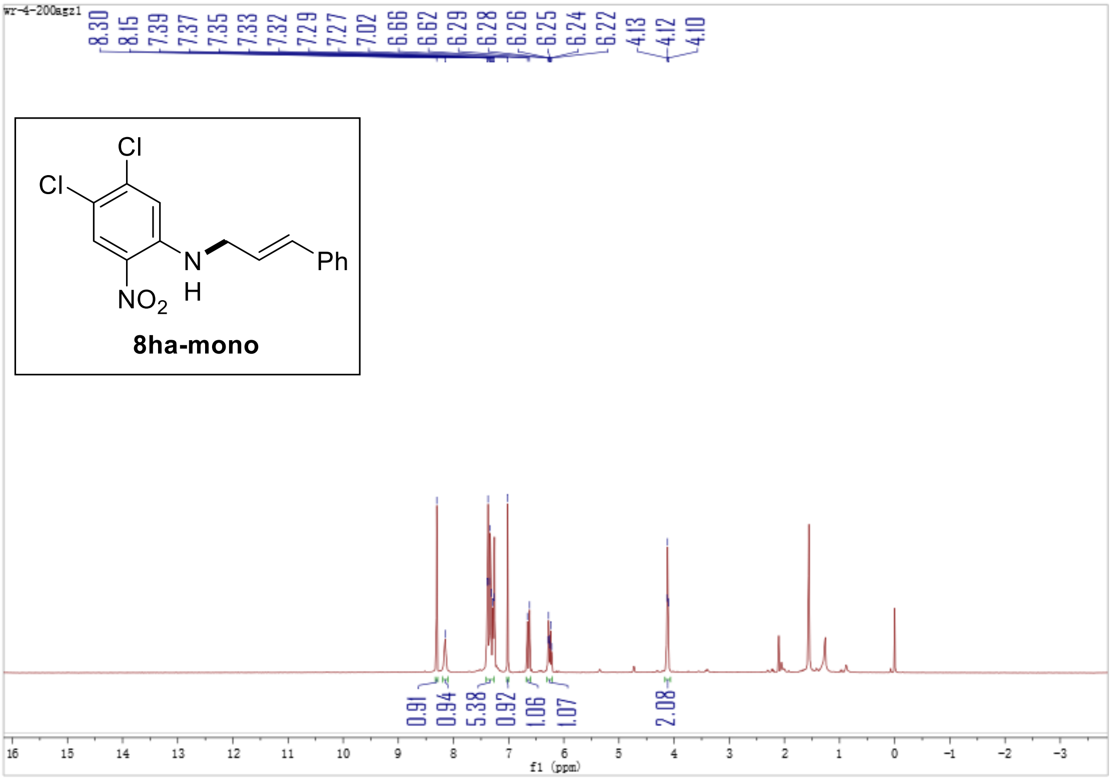

^{13}C NMR spectrum of compound **8ha-mono** ($CDCl_3$, 101 MHz)

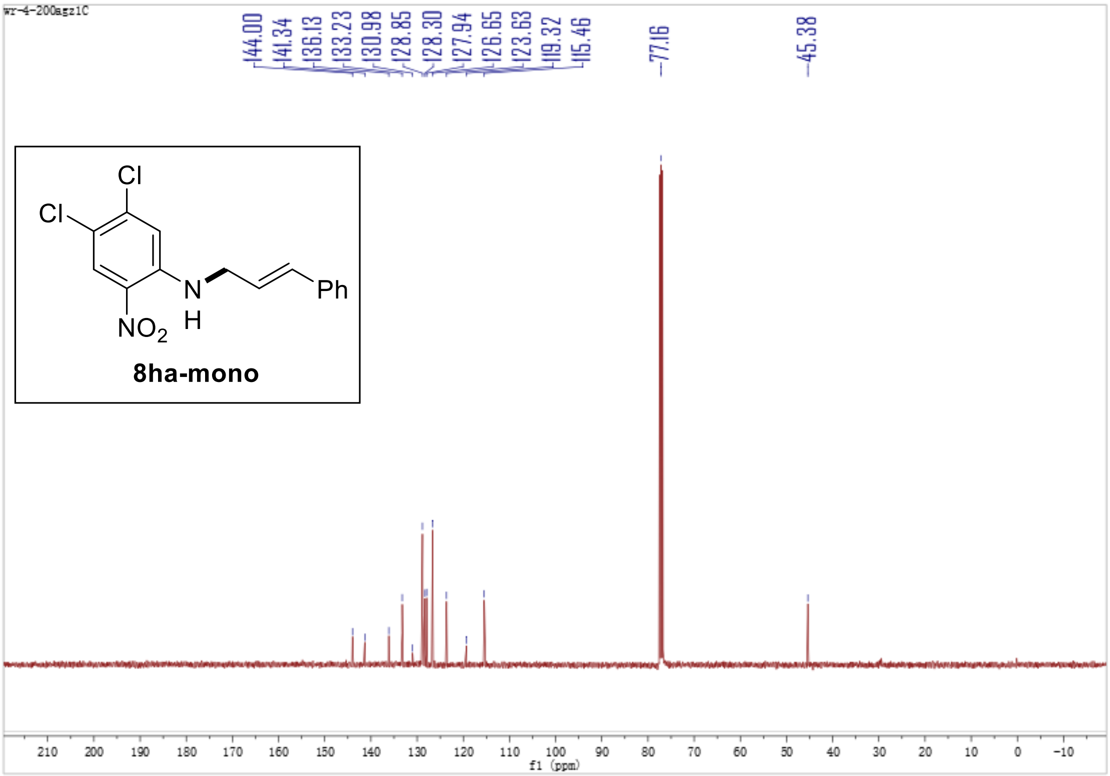

[1]**H NMR** spectrum of compound **8ia-mono** ($CDCl_3$, 400 MHz)

Links CATALOG DETAILS NMR

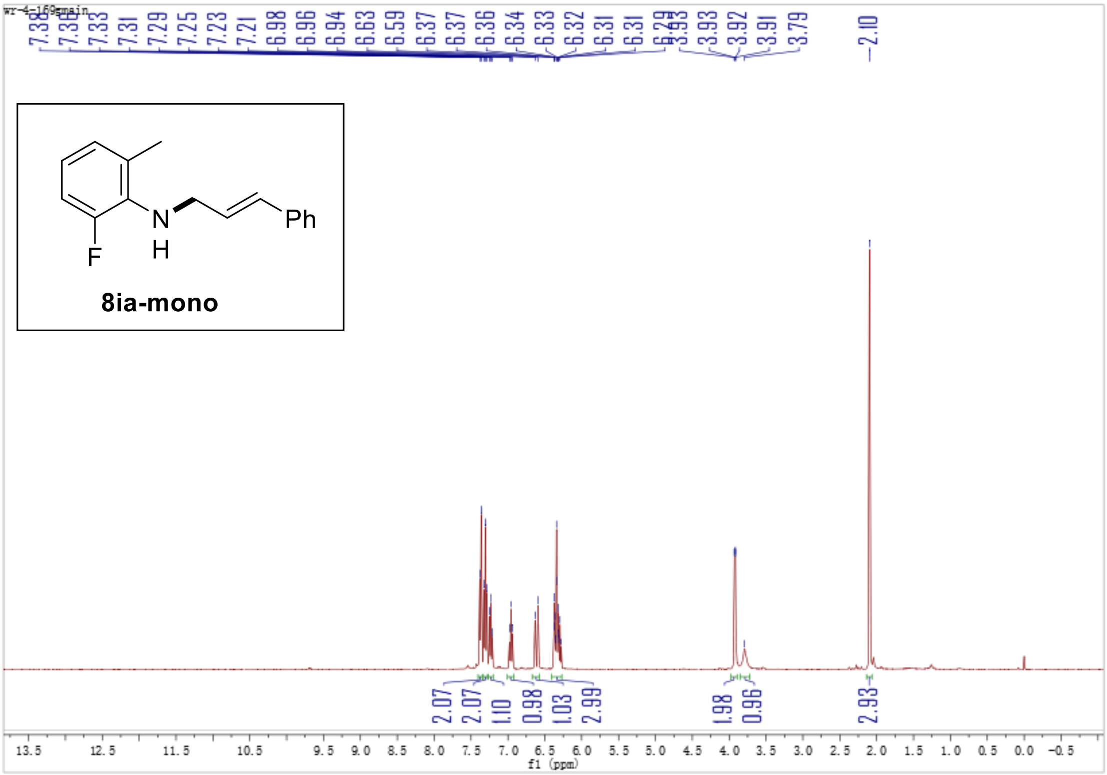

[13]**C NMR** spectrum of compound **8ia-mono** ($CDCl_3$, 101 MHz)

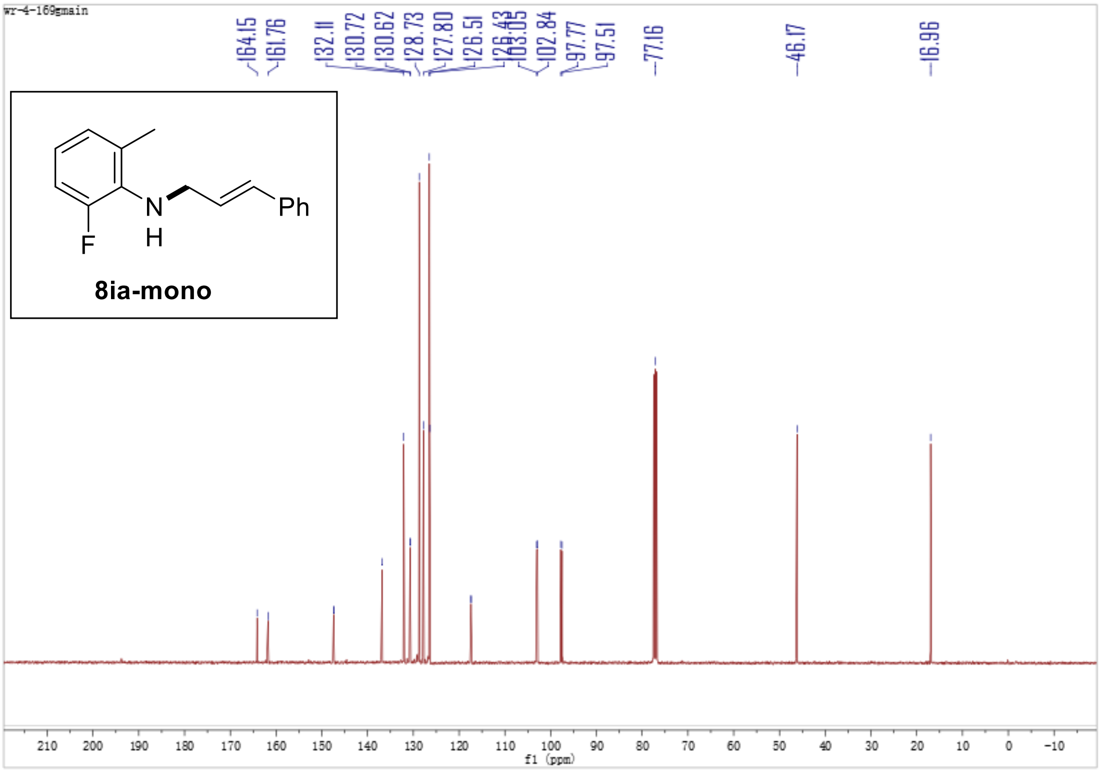

^{1}H NMR spectrum of compound **8ja-mono** ($CDCl_3$, 400 MHz)

Links CATALOG DETAILS NMR

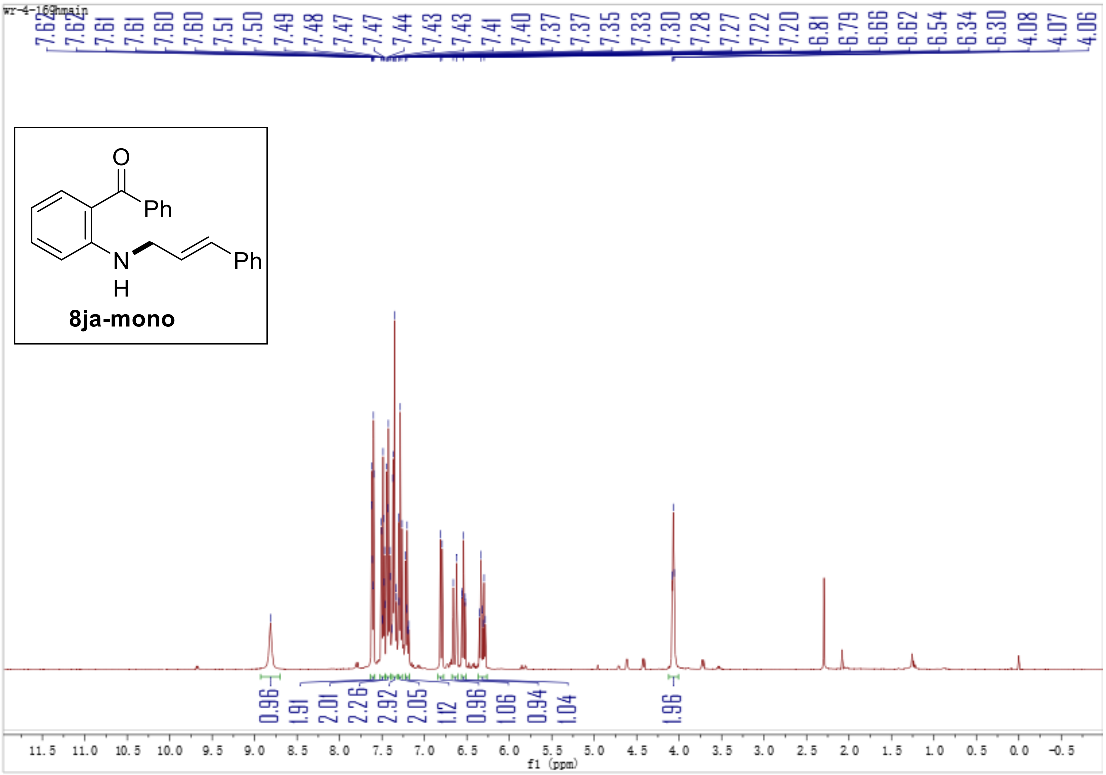

^{13}C NMR spectrum of compound **8ja-mono** ($CDCl_3$, 101 MHz)

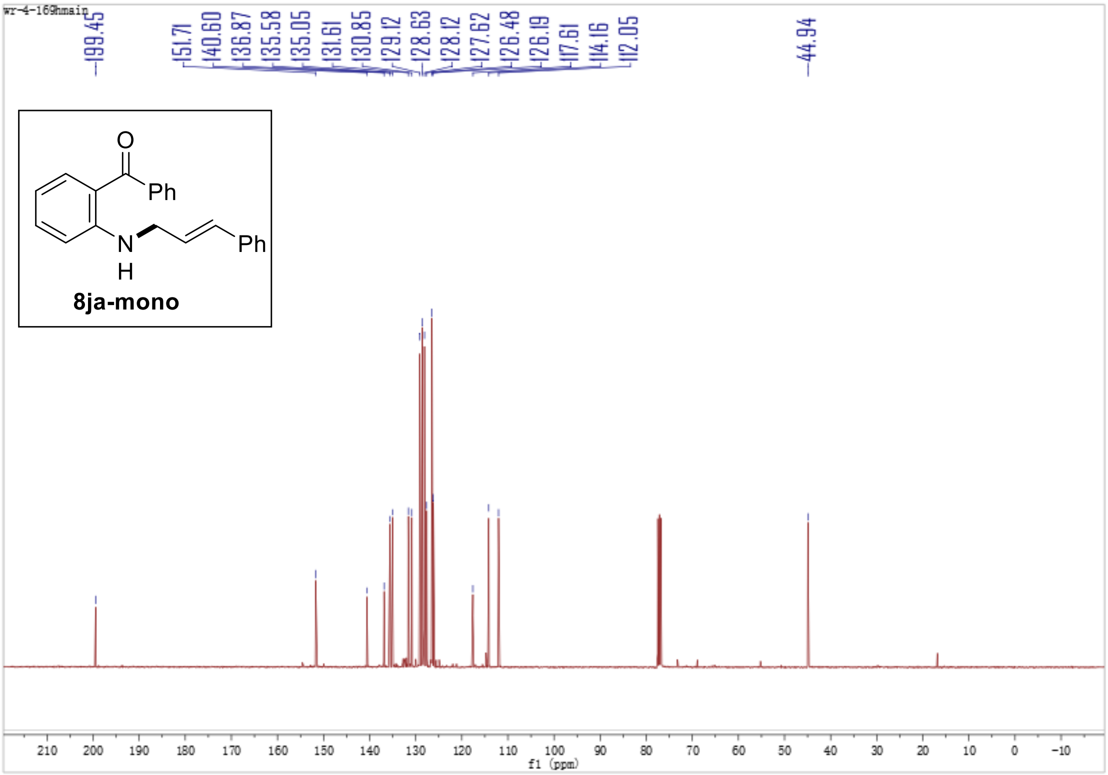

^{1}**H NMR** spectrum of compound **8ba-di** ($CDCl_3$, 400 MHz)

Links CATALOG DETAILS NMR

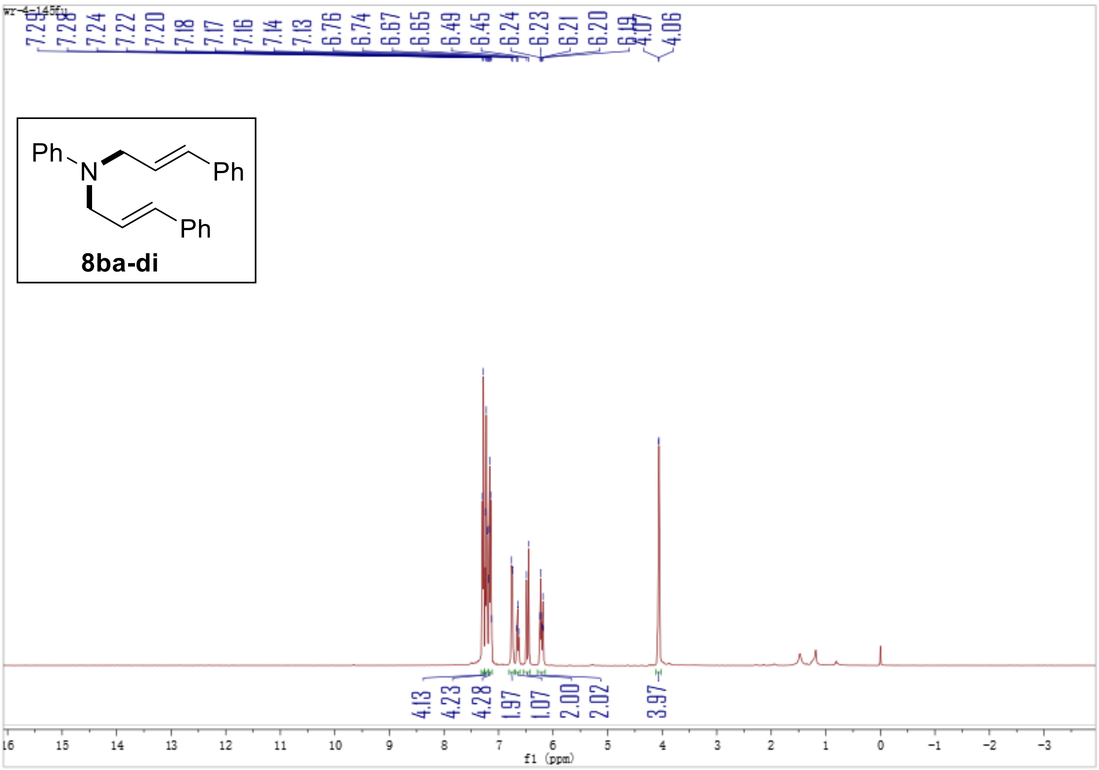

^{13}**C NMR** spectrum of compound **8ba-di** ($CDCl_3$, 101 MHz)

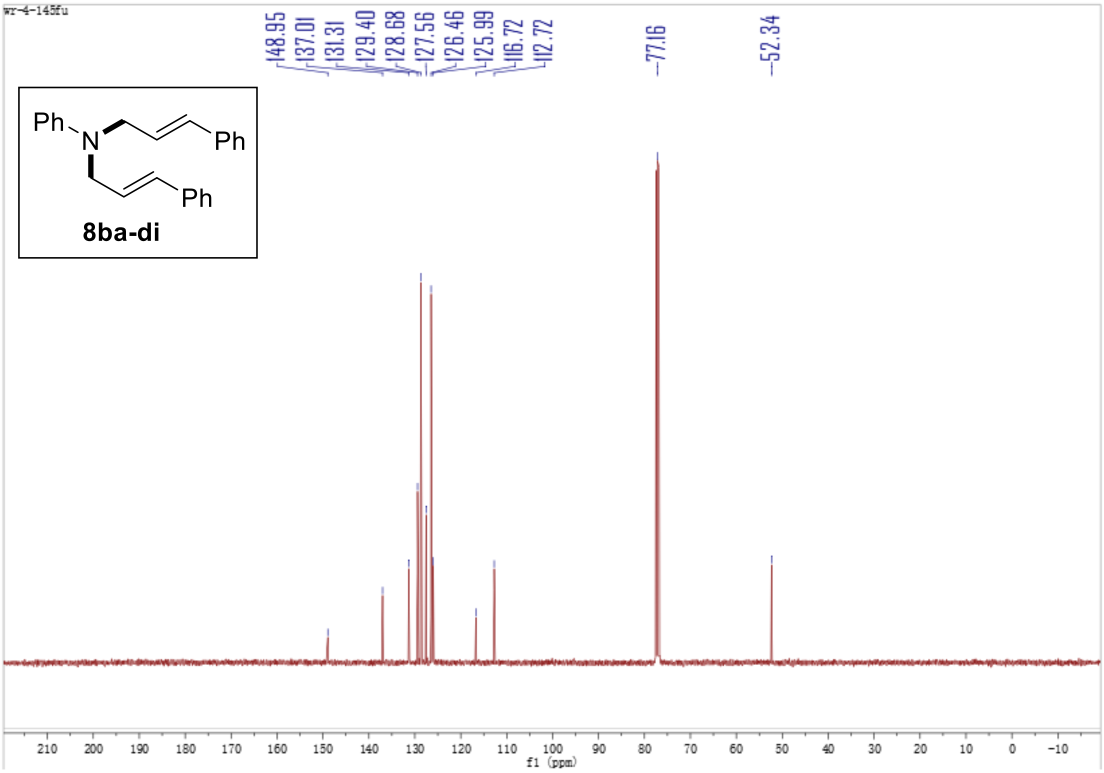

^{1}H **NMR** spectrum of compound **8ca-di** ($CDCl_3$, 400 MHz)

Links CATALOG DETAILS NMR

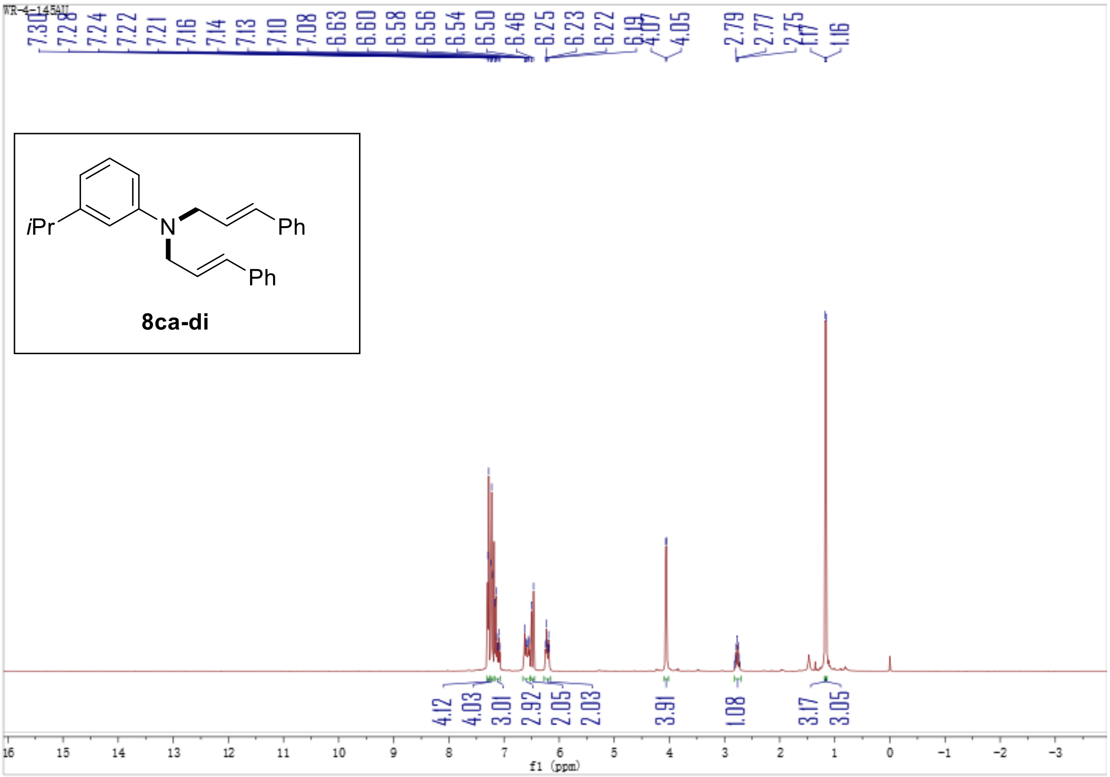

^{13}C **NMR** spectrum of compound **8ca-di** ($CDCl_3$, 101 MHz)

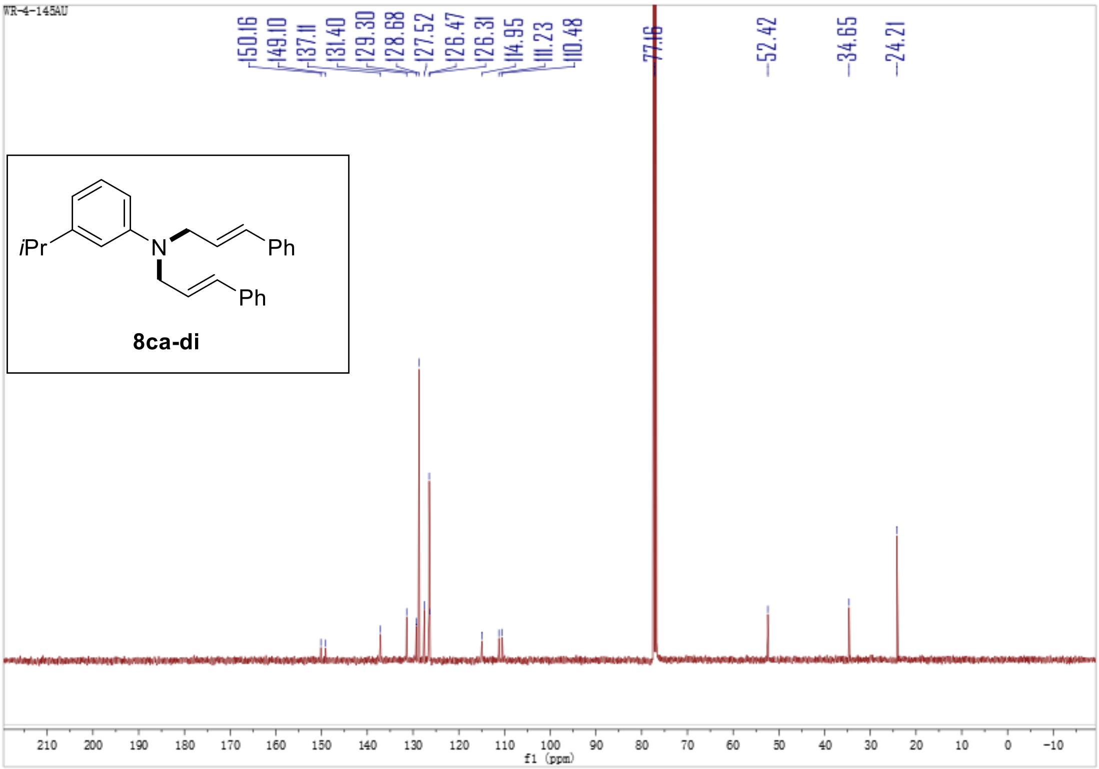

^{1}**H NMR** spectrum of compound **8da-di** ($CDCl_3$, 400 MHz)

Links CATALOG DETAILS NMR

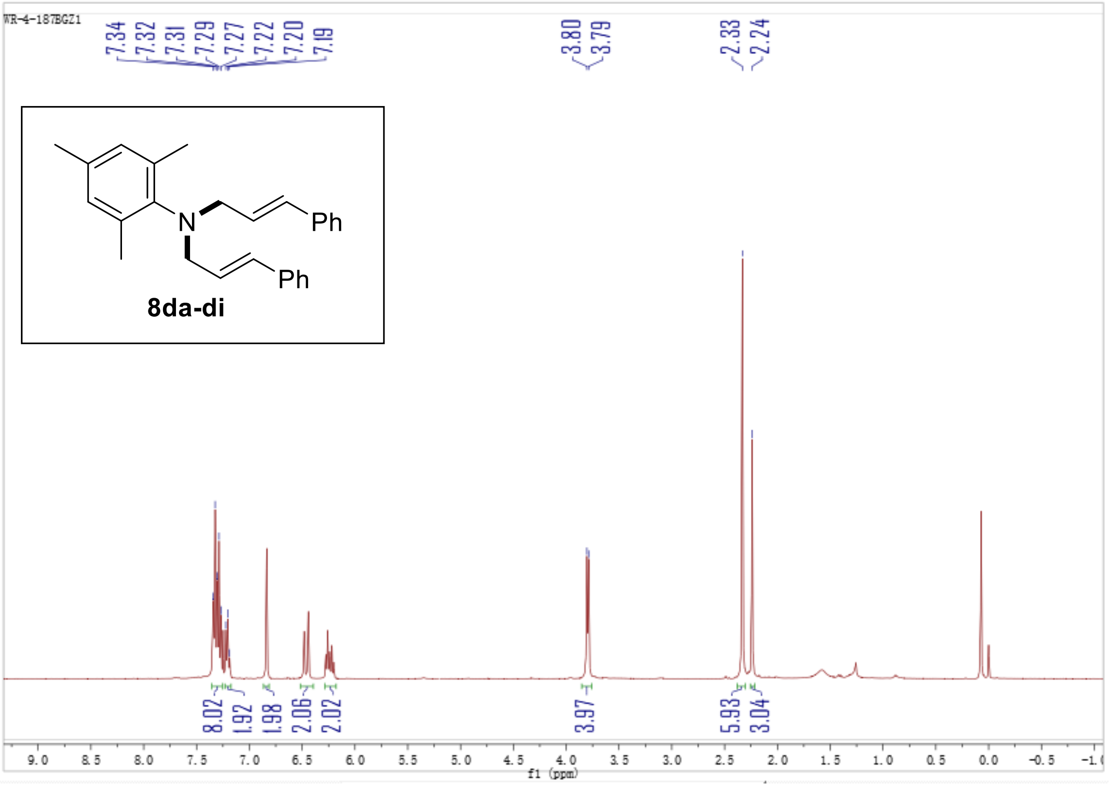

^{13}**C NMR** spectrum of compound **8da-di** ($CDCl_3$, 101 MHz)

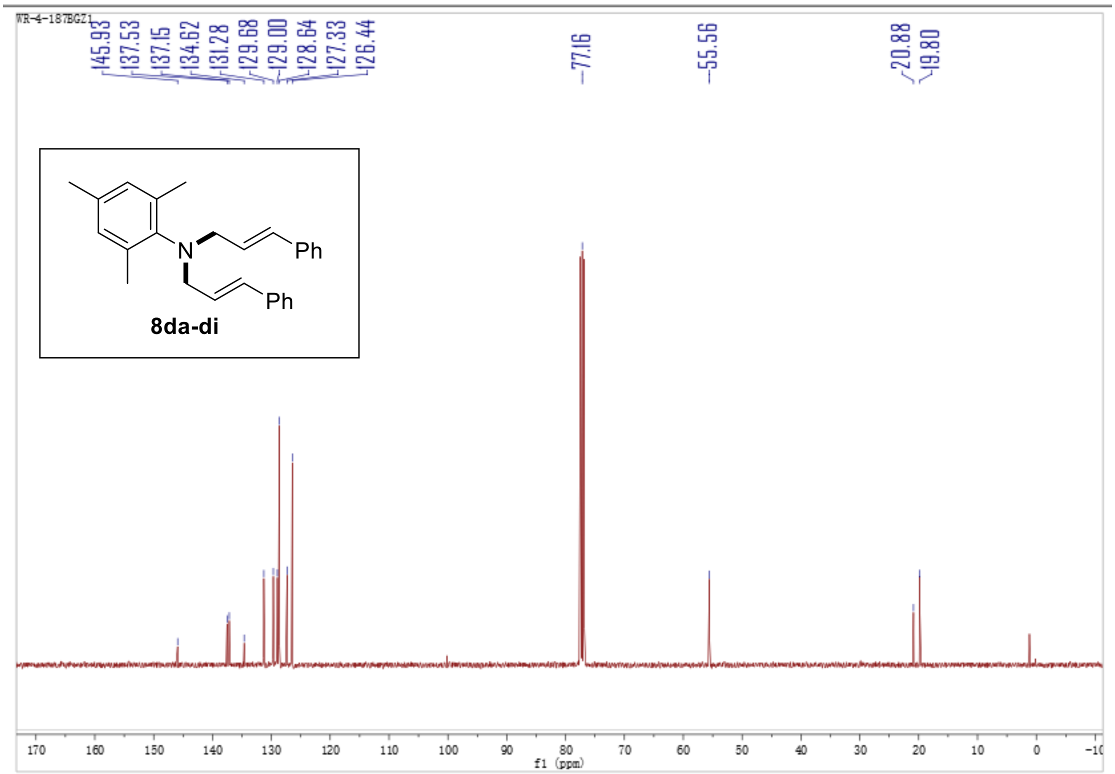

[1]**H NMR** spectrum of compound **8ea-di** ($CDCl_3$, 400 MHz)

Links CATALOG DETAILS NMR

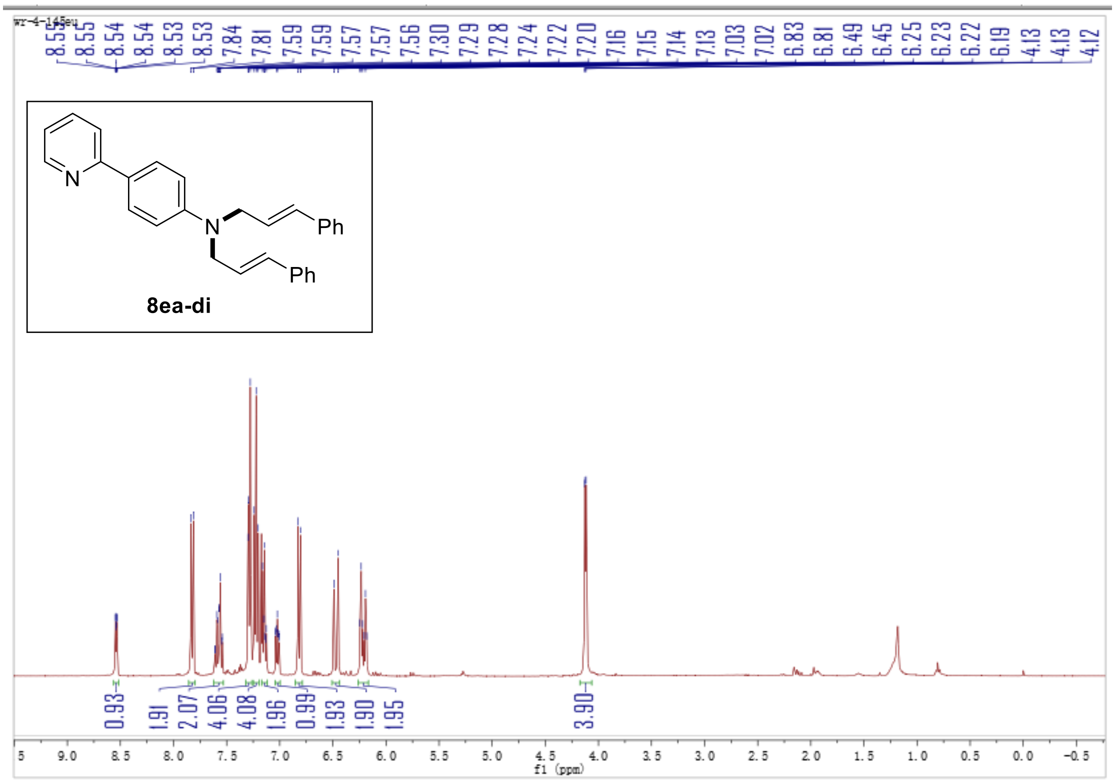

[13]**C NMR** spectrum of compound **8ea-di** ($CDCl_3$, 101 MHz)

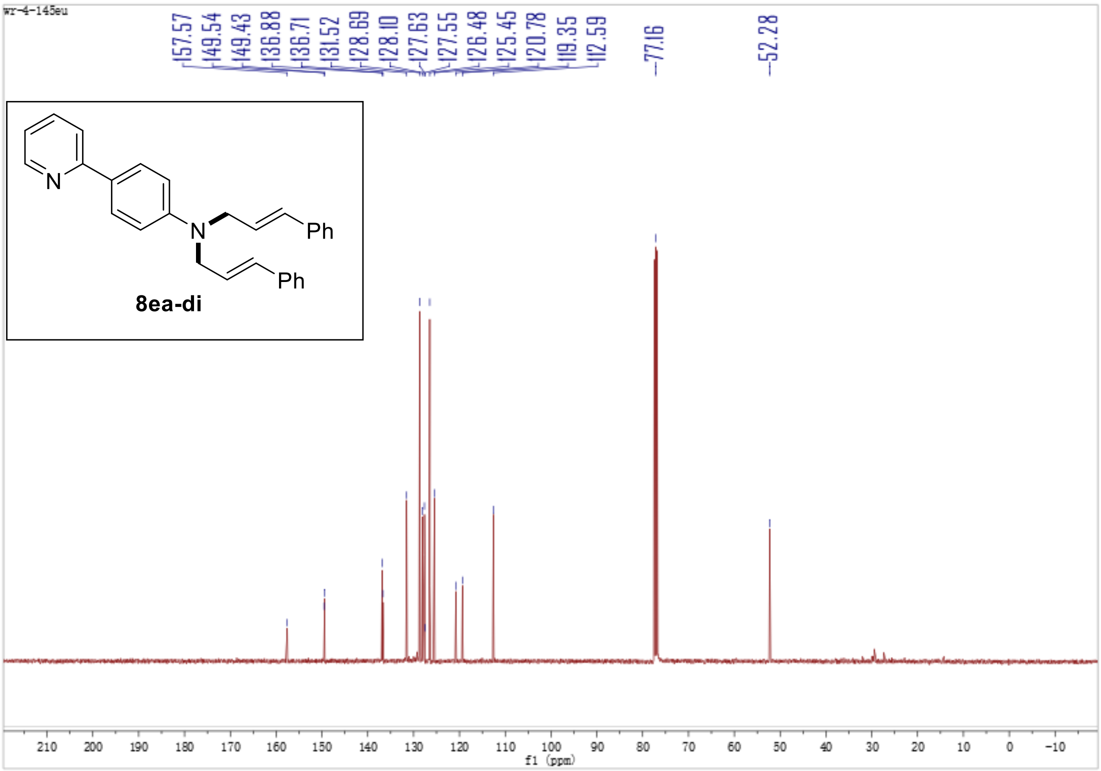

^{1}H NMR spectrum of compound **8la-di** ($CDCl_3$, 400 MHz)

Links CATALOG DETAILS NMR

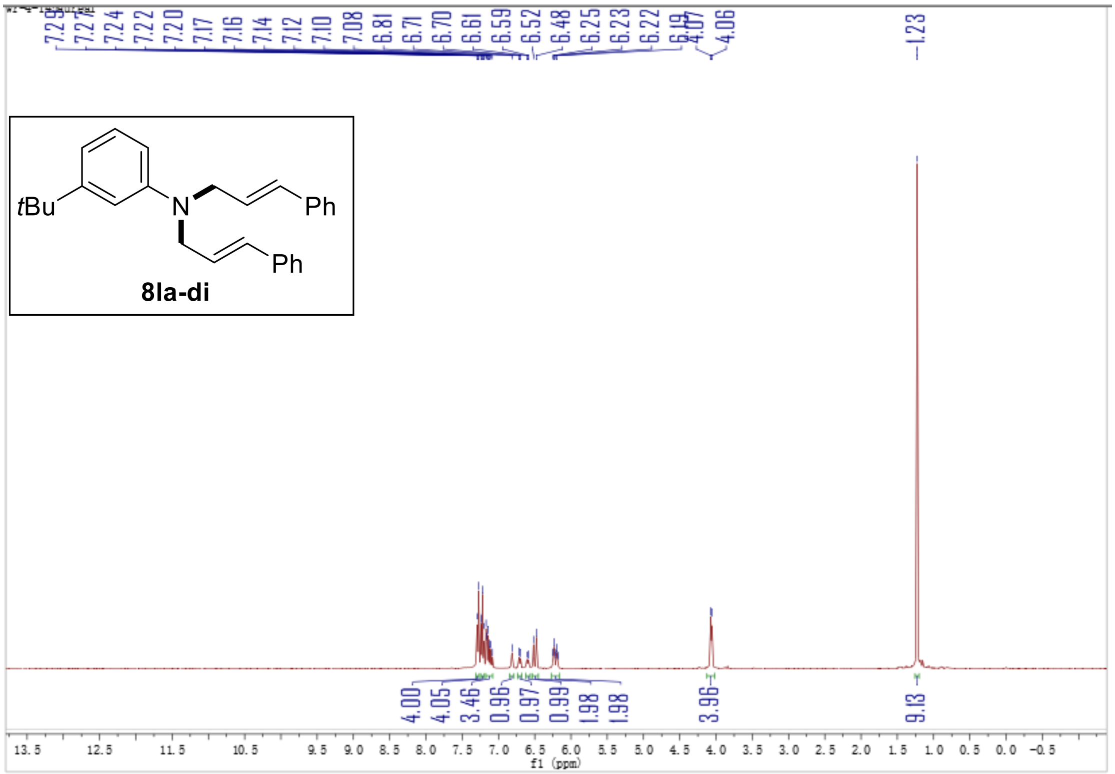

^{13}C NMR spectrum of compound **8la-di** ($CDCl_3$, 101 MHz)

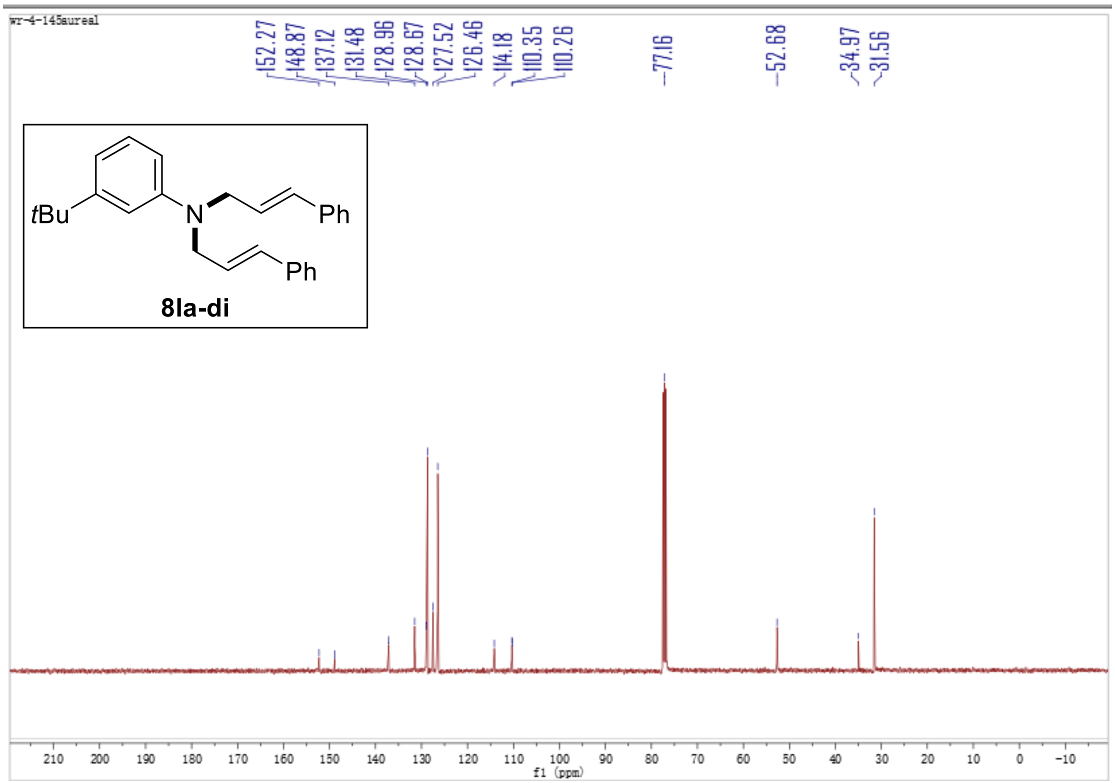

^{1}H NMR spectrum of compound **8ma-di** ($CDCl_3$, 400 MHz)

Links CATALOG DETAILS NMR

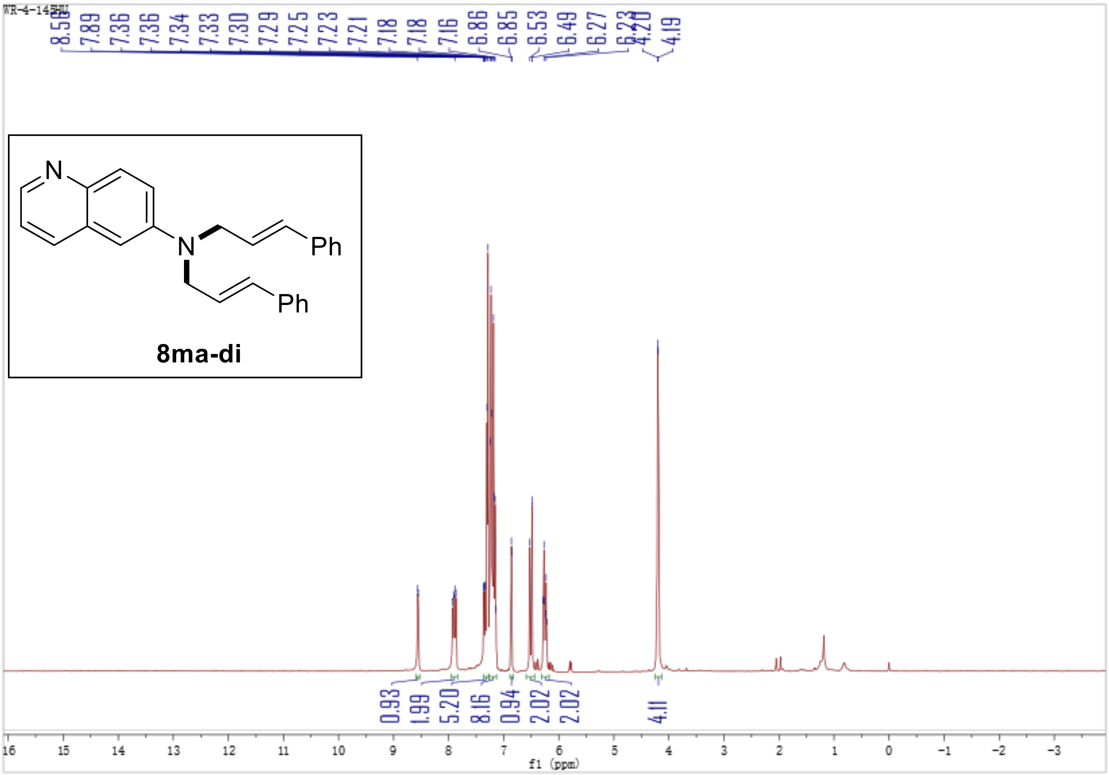

^{13}C NMR spectrum of compound **8ma-di** ($CDCl_3$, 101 MHz)

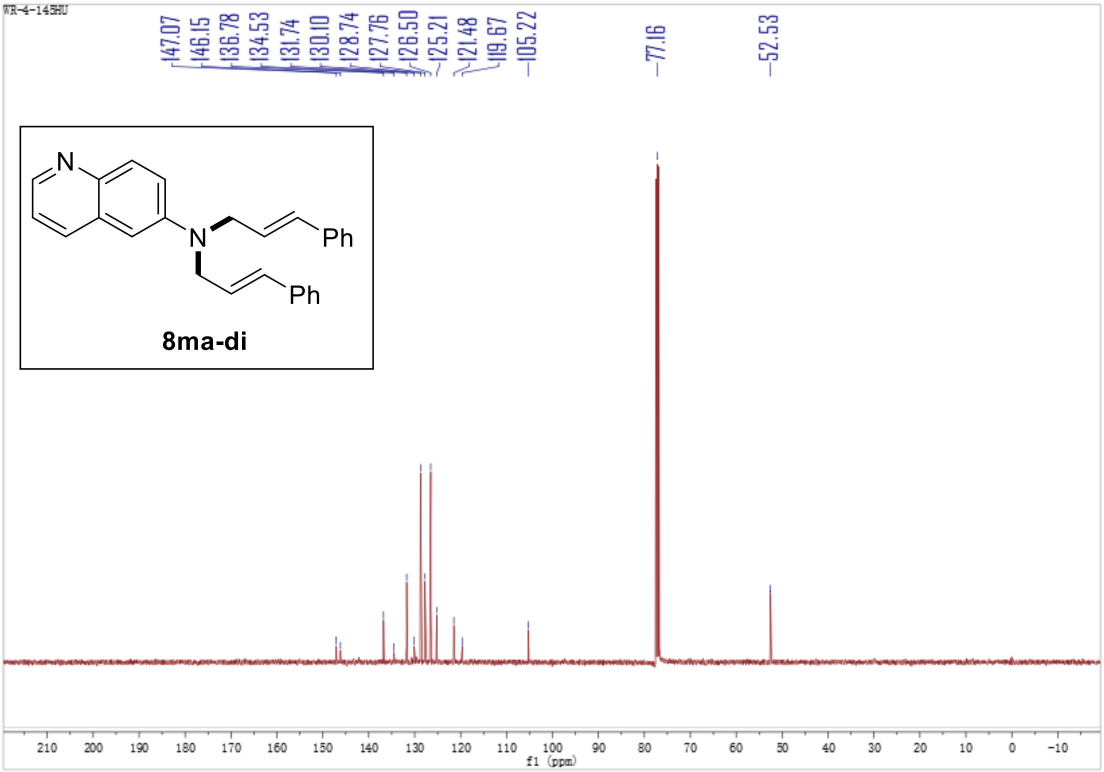

^{1}**H NMR** spectrum of compound **8dax** ($CDCl_3$, 400 MHz)

Links CATALOG DETAILS NMR

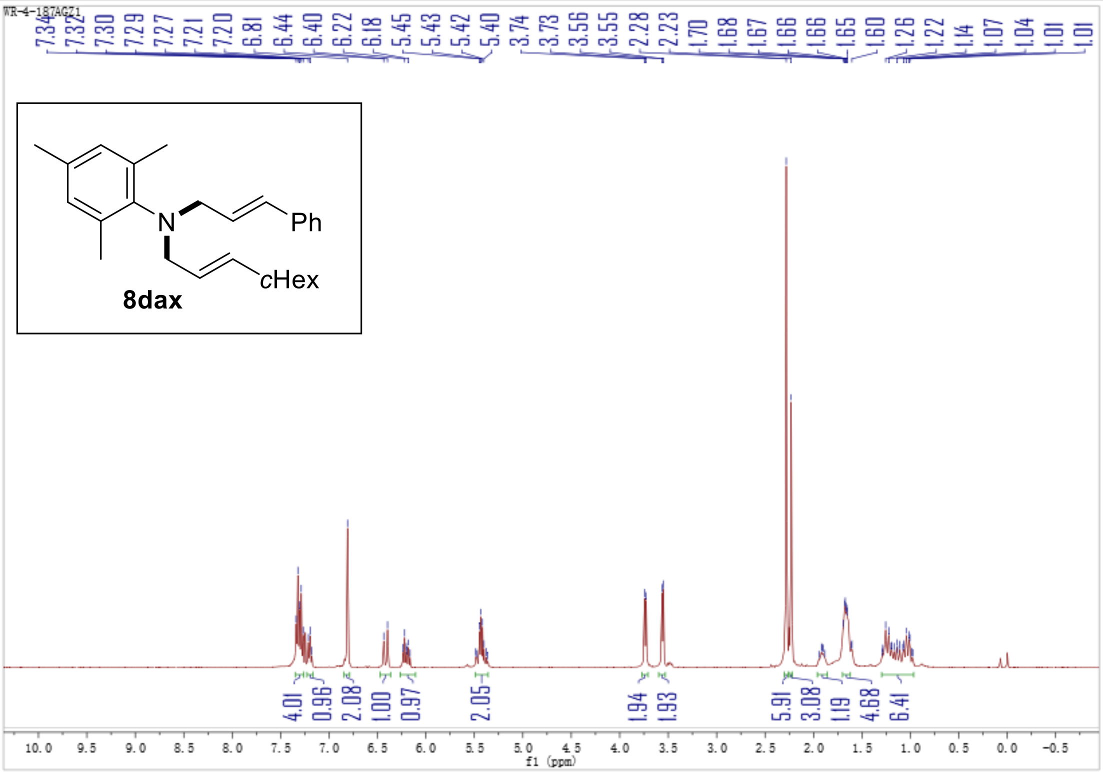

^{13}**C NMR** spectrum of compound **8dax** ($CDCl_3$, 101 MHz)

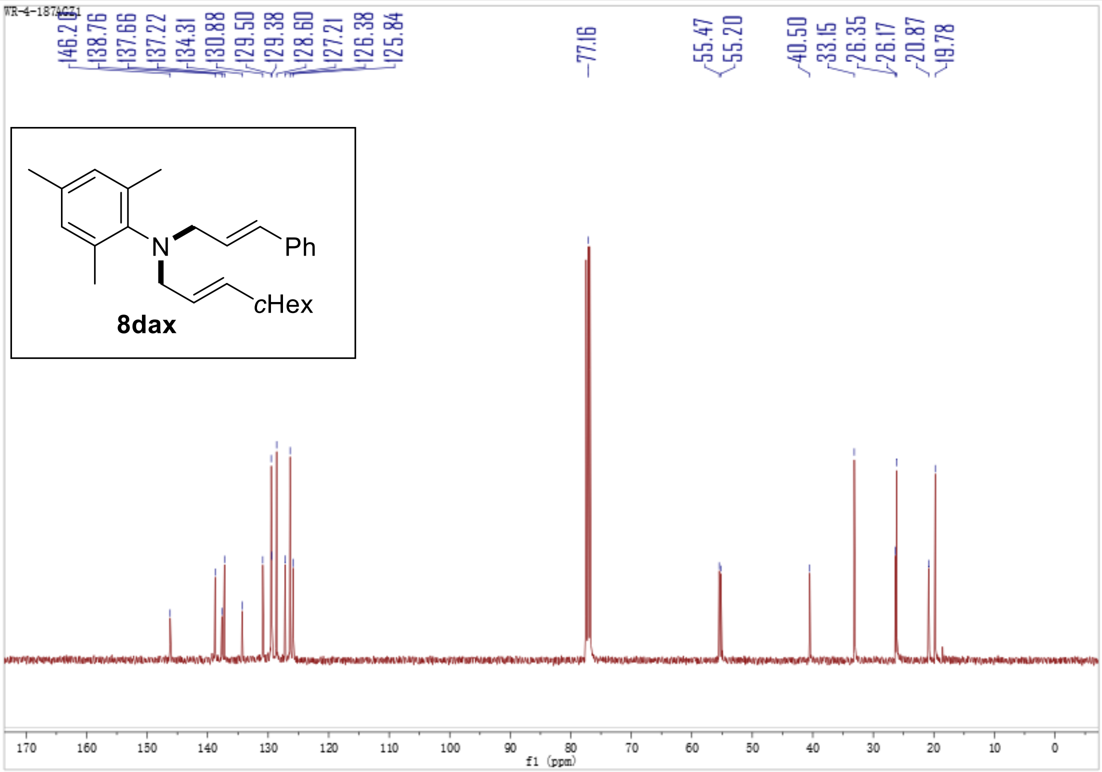

^{1}H NMR spectrum of compound **8dam** ($CDCl_3$, 400 MHz)

Links CATALOG DETAILS NMR

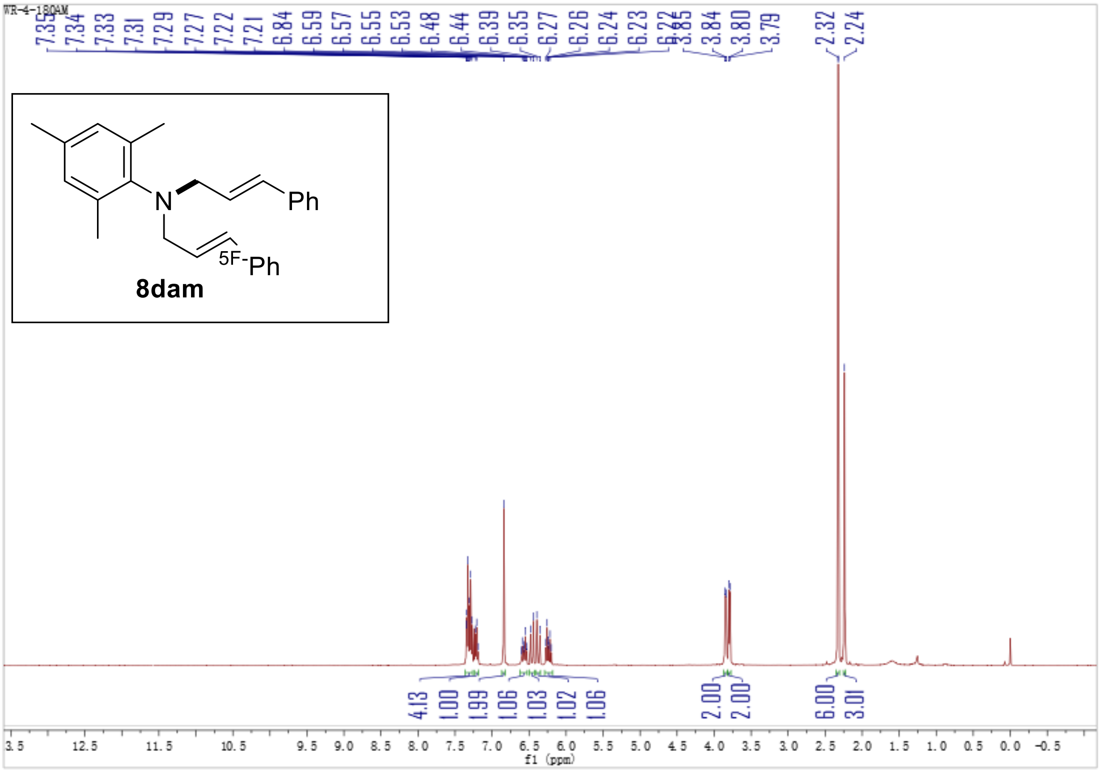

^{13}C NMR spectrum of compound **8dam** ($CDCl_3$, 101 MHz)

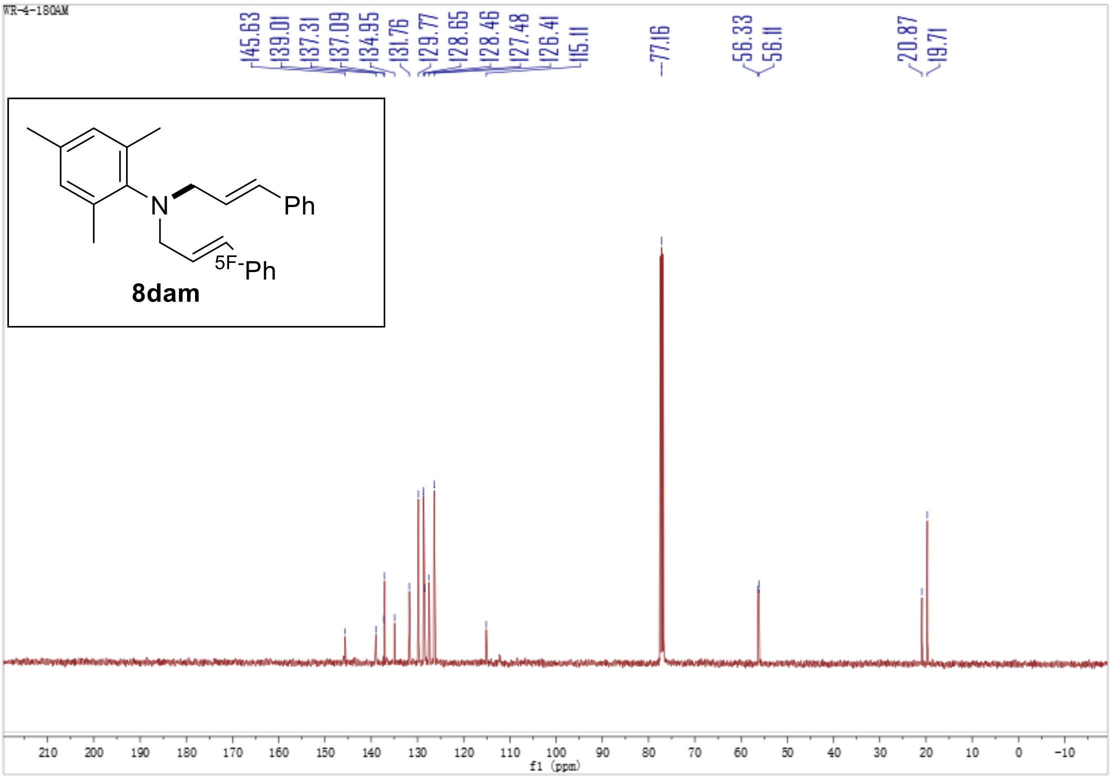

^{19}F NMR spectrum of compound **8dam** ($CDCl_3$, 374 MHz)

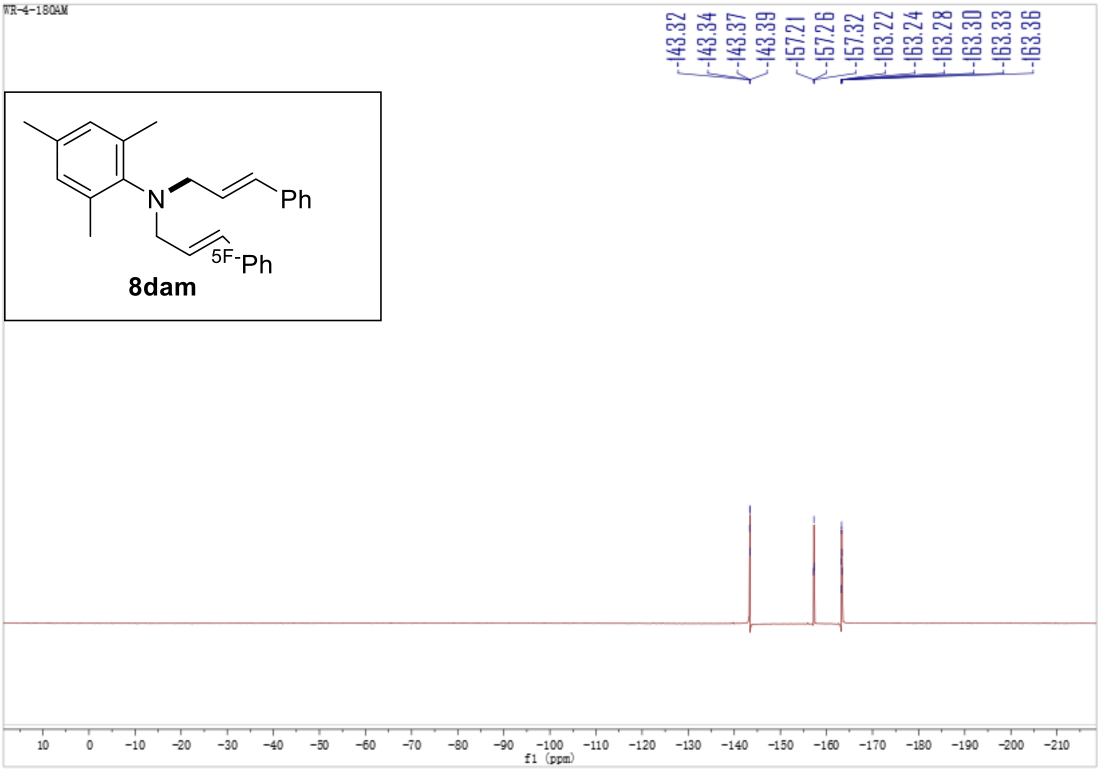

^{1}H NMR spectrum of compound **9aa** ($CDCl_3$, 400 MHz)

Links CATALOG DETAILS NMR

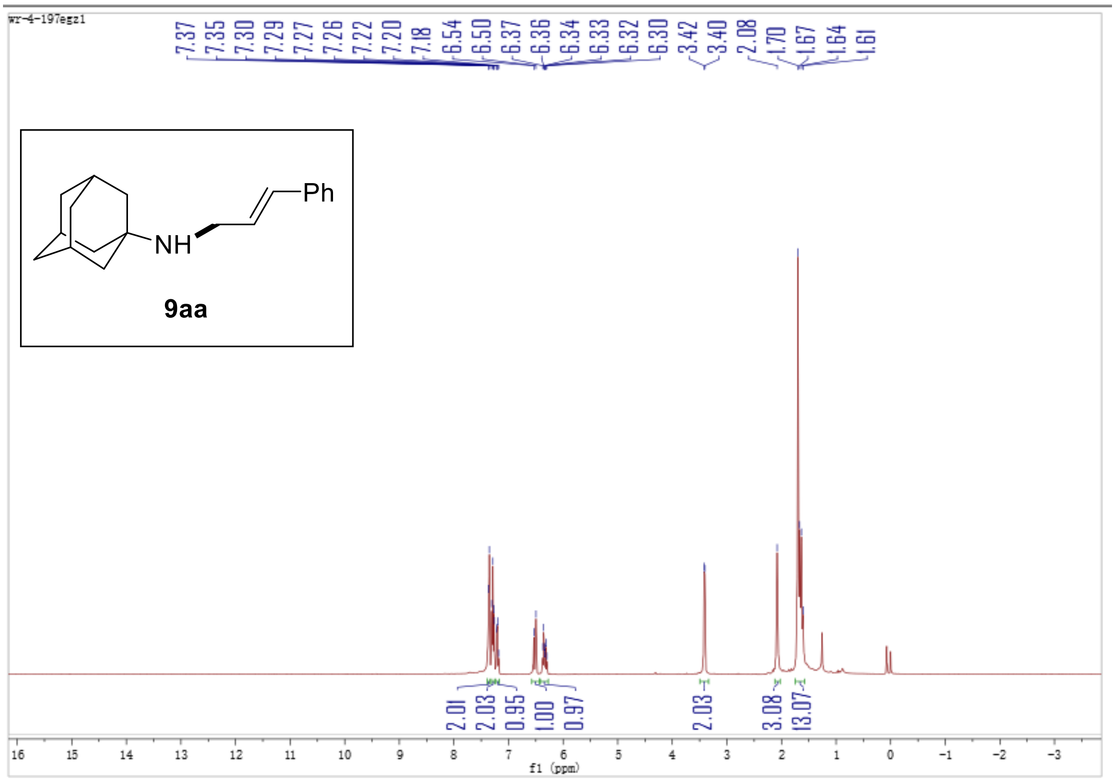

^{13}C NMR spectrum of compound **9aa** ($CDCl_3$, 101 MHz)

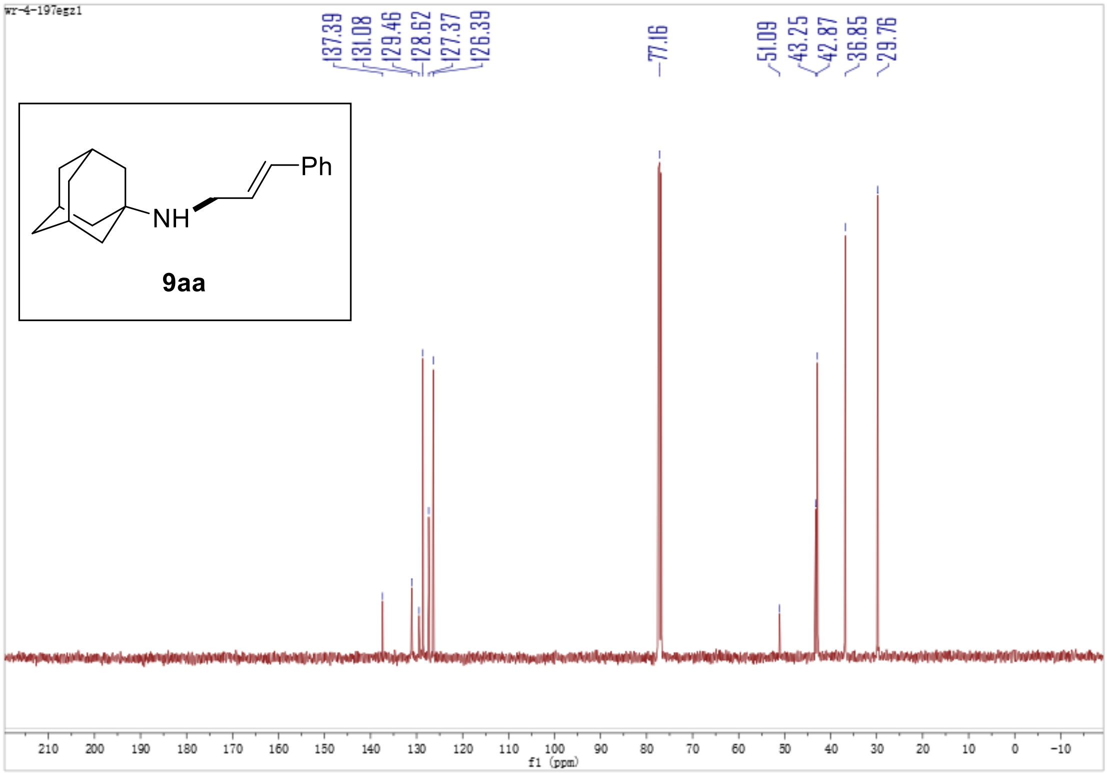

^{1}H NMR spectrum of compound **9ba** ($CDCl_3$, 400 MHz)

Links CATALOG DETAILS NMR

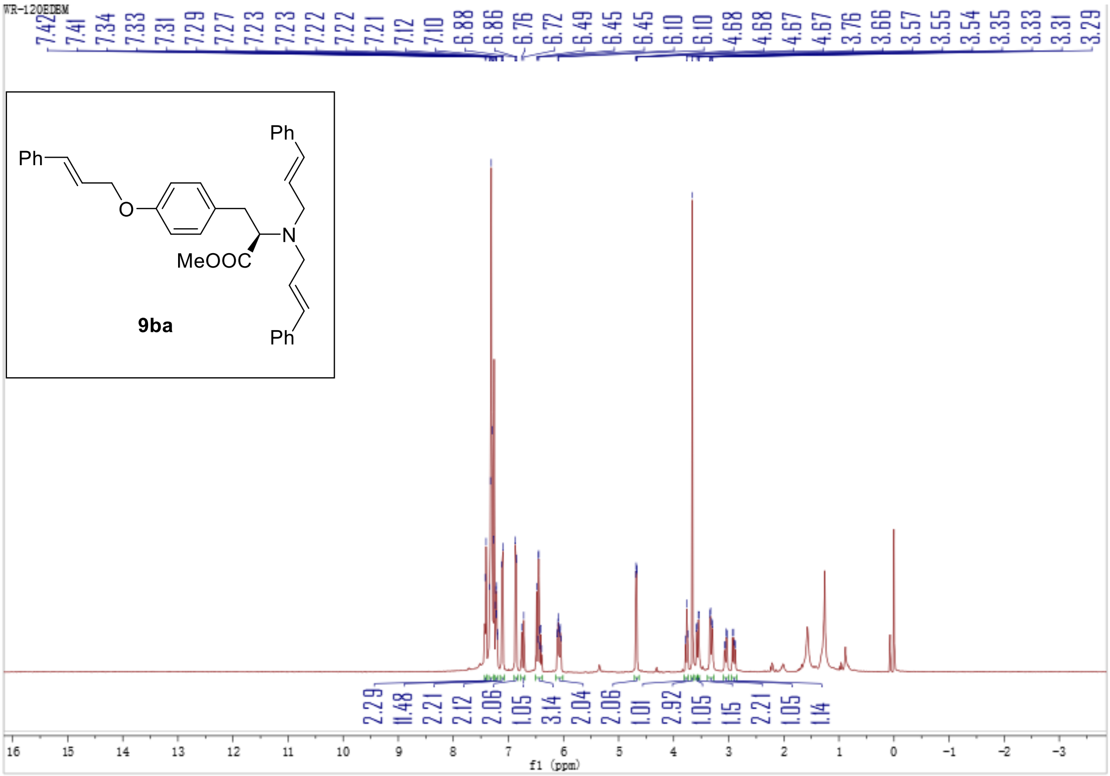

^{13}C NMR spectrum of compound **9ba** ($CDCl_3$, 101 MHz)

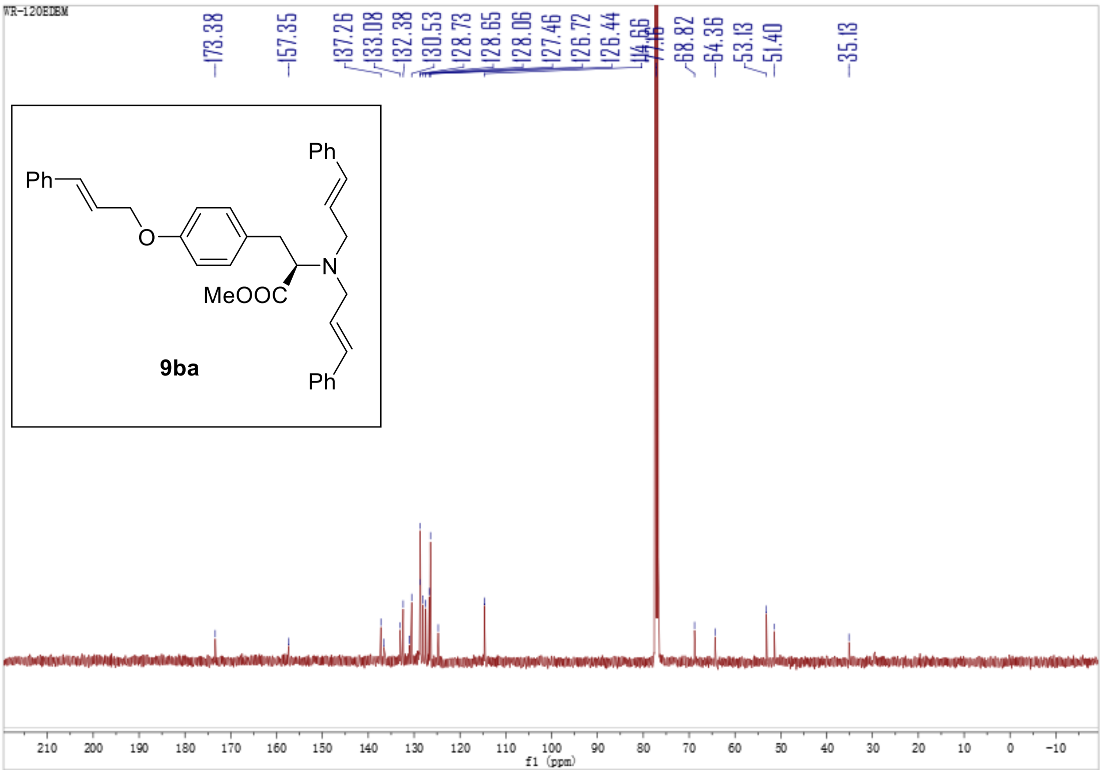

7. HPLC Spectra

Ethyl (*S*,*E*)-2-acetyl-2-methyl-5-phenylpent-4-enoate **(3aa)**

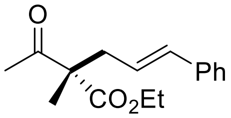

3aa

Links DETAILS NMR HPLC CATALOG

HPLC spectra of racemic **3aa**.

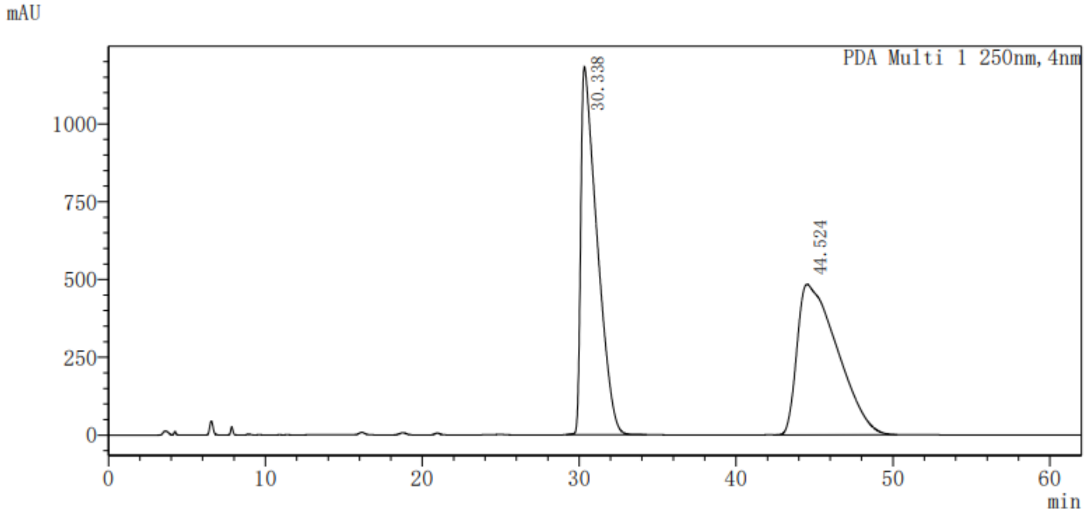

<峰表>

PDA Ch1 250nm

峰号	保留时间	面积	高度	面积%
1	30.338	85356792	1181955	49.835
2	44.524	85923314	483693	50.165
总计		171280106	1665648	100.000

HPLC spectra of chiral **3aa**.

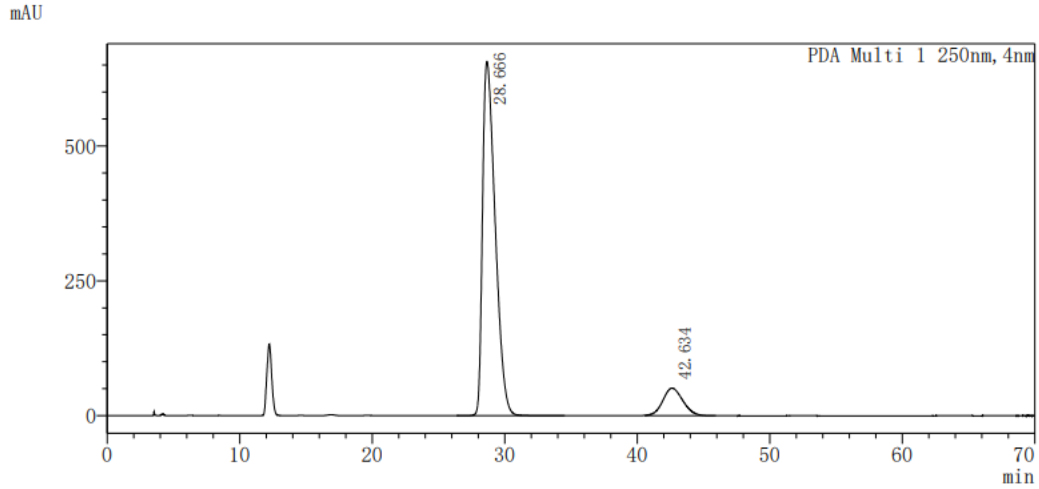

<峰表>

PDA Ch1 250nm

峰号	保留时间	面积	高度	面积%
1	28.666	43604767	656689	89.098
2	42.634	5335394	51014	10.902
总计		48940161	707703	100.000

Ethyl (S)-1-cinnamyl-2-oxocyclohexane-1-carboxylate **(3ha)**

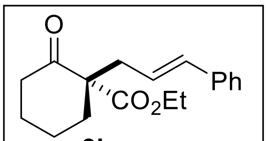

3ha

Links DETAILS NMR HPLC CATALOG E-Fact

HPLC spectra of racemic **3ha**.

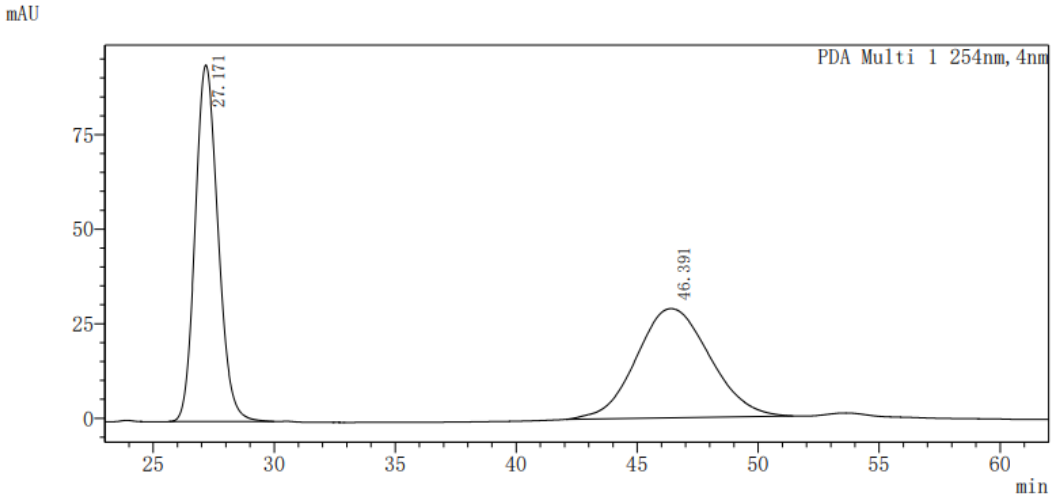

<峰表>

PDA Ch1 254nm

峰号	保留时间	面积	高度	面积%
1	27.171	6177912	94274	50.368
2	46.391	6087617	28862	49.632
总计		12265529	123136	100.000

HPLC spectra of chiral **3ha**.

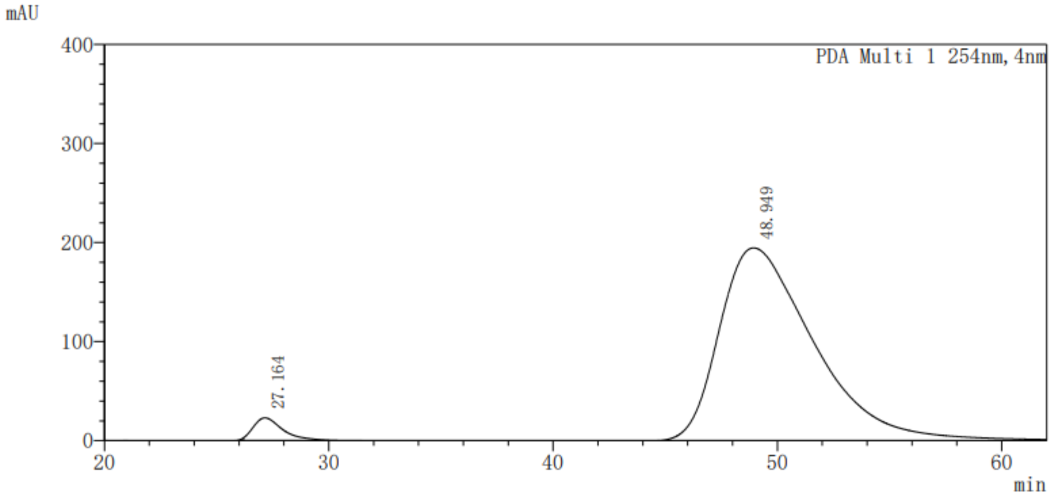

<峰表>

PDA Ch1 254nm

峰号	保留时间	面积	高度	面积%
1	27.164	2114802	23015	3.565
2	48.949	57211210	195054	96.435
总计		59326012	218069	100.000

Ethyl (*S*,*Z*)-2-oxo-1-(3-phenylallyl)cyclohexane-1-carboxylate **(3ha-*Z*)**

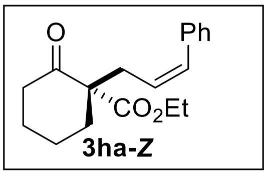

Links DETAILS NMR HPLC CATALOG

HPLC spectra of racemic **3ha-*Z***.

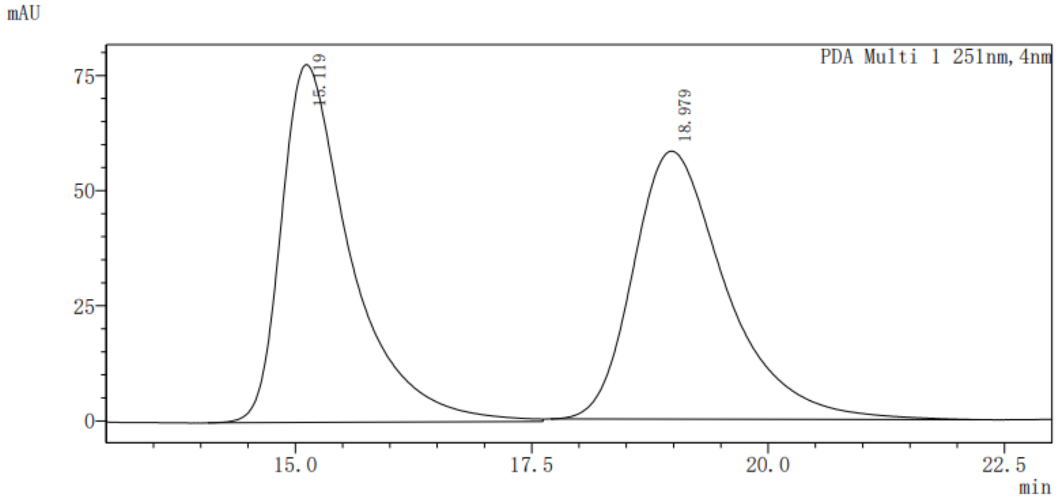

<峰表>

PDA Ch1 251nm

峰号	保留时间	面积	高度	面积%
1	15.119	3990060	77702	49.990
2	18.979	3991618	58176	50.010
总计		7981678	135878	100.000

HPLC spectra of chiral **3ha-*Z***.

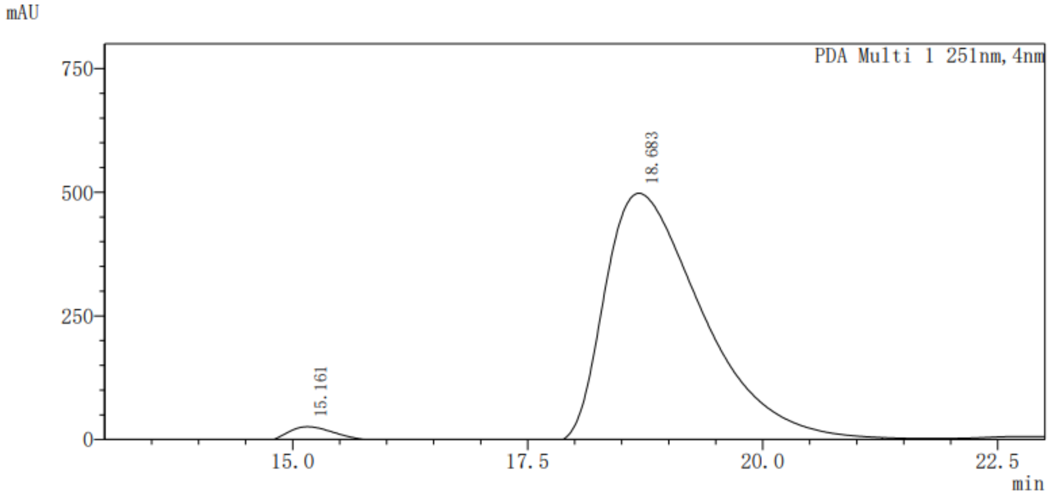

<峰表>

PDA Ch1 251nm

峰号	保留时间	面积	高度	面积%
1	15.161	1890088	38089	4.725
2	18.683	38108314	508933	95.275
总计		39998402	547022	100.000

8. References

[1] Anson CW, Ghosh S, Hammes-Schiffer S et al. Co(salophen)-catalyzed aerobic oxidation of *p*-hydroquinone:mechanism and implications for aerobic oxidation catalysis. *J Am Chem Soc* 2016; **138**: 4186-4193.

[2] 2,6-DMHQ was stirred with deuterated water overnight, and after extraction, NMR monitoring confirmed complete deuteration.

[3] Wang R, Zhang L, Luo S. Aerobic asymmetric allylic C−H alkylation by synergistic chiral primary amine-palladium-hydroquinone catalysis. *Chem Eur J* 2024; **30**: e202304316.

[4] Roger AS. The *E* factor 25 years on: the rise of green chemistry and sustainability. *Green Chem* 2017; **19**: 18-43.

[5] Ali S Z, Budaitis BG, Fontaine DFA et al. Allylic C–H amination cross-coupling furnishes tertiary amines by electrophilic metal catalysis. *Science* 2022; **376**: 276-283.
